# The Final Stereogenic Unit of [2]Rotaxanes: Type 2 Geometric Isomers

**DOI:** 10.1021/jacs.3c14594

**Published:** 2024-03-18

**Authors:** Andrea Savoini, Peter R. Gallagher, Abed Saady, Stephen M. Goldup

**Affiliations:** † School of Chemistry, University of Southampton, University Road, Southampton SO17 1BJ, U.K.; ‡ School of Chemistry, University of Birmingham, Edgbaston, Birmingham B15 2TT, U.K.

## Abstract

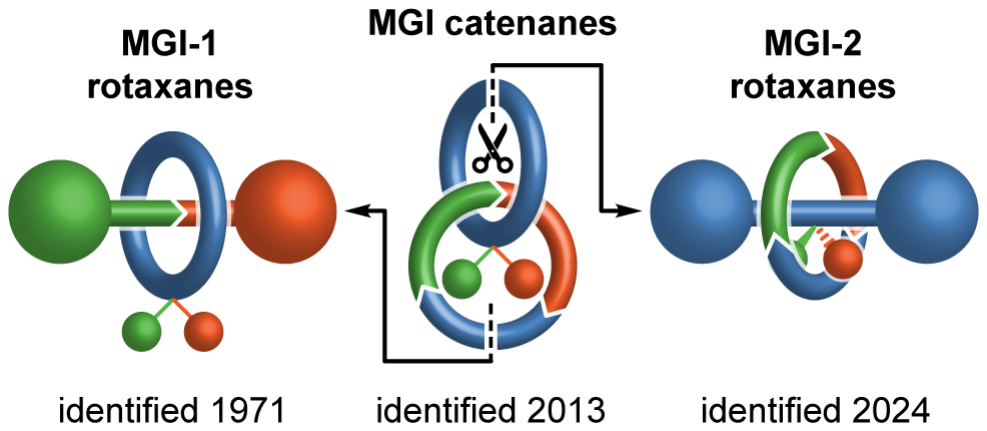

Mechanical stereochemistry arises when the interlocking of stereochemically trivial covalent subcomponents results in a stereochemically complex object. Although this general concept was identified in 1961, the stereochemical description of these molecules is still under development to the extent that new forms of mechanical stereochemistry are still being identified. Here, we present a simple analysis of rotaxane and catenane stereochemistry that allowed us to identify the final missing simple mechanical stereogenic unit, an overlooked form of rotaxane geometric isomerism, and demonstrate its stereoselective synthesis.

## Introduction

In 1961,^1^ Wasserman and Frisch recognized that interlocking two non-stereogenic rings can result in a chiral catenane where the enantiomers are related by inverting the relative orientations of the two rings.^2^ A decade later,^3^ Schill identified a similar phenomenon when a ring encircles an axle in a rotaxane, and that geometric isomerism is also possible in such systems. Since these first reports, the pantheon of mechanical stereogenic units in simple [2]catenanes and [2]rotaxanes has expanded beyond those envisaged by Wassermann and Frisch, and Schill; in 2013,^4^ Gaeta and Neri recognized that catenanes can also express mechanical geometric isomerism and more recently, we identified a previously overlooked class of mechanically chiral rotaxanes^5a^ and reanalyzed the planar chiral stereochemistry of catenanes to show that, although they were hitherto simply described as “topologically chiral”, this is not an essential characteristic of this stereogenic unit.^6^

The recent discovery of new conditional^7^ mechanical stereogenic units contrasts with covalent organic stereochemistry where, although new pathways of isomerization^8^ and previously overlooked expressions of atropisomerism^9^ have recently been reported, the archetypal stereogenic units (centers, axes, planes, helices, and multiple bonds)^10^ are long-established. This raises an obvious question; are there any mechanical stereogenic units of [2]catenanes and [2]rotaxanes still lying undetected? Here, we provide a simple stereochemical analysis that shows the answer is yes. Working from first-principles we identify a previously overlooked rotaxane geometric stereogenic unit but also demonstrate that this is the final one to be found; our pantheon is now complete (Figure 2). Using concepts developed for the synthesis of chiral rotaxanes, we demonstrate the first stereoselective synthesis of these new type 2 rotaxane mechanical geometric isomers.

## Results and Discussion

### Examining the Achiral Building Blocks of [2]Catenanes Confirms that the Set of Known Stereogenic Units is Complete

**Figure 1:**
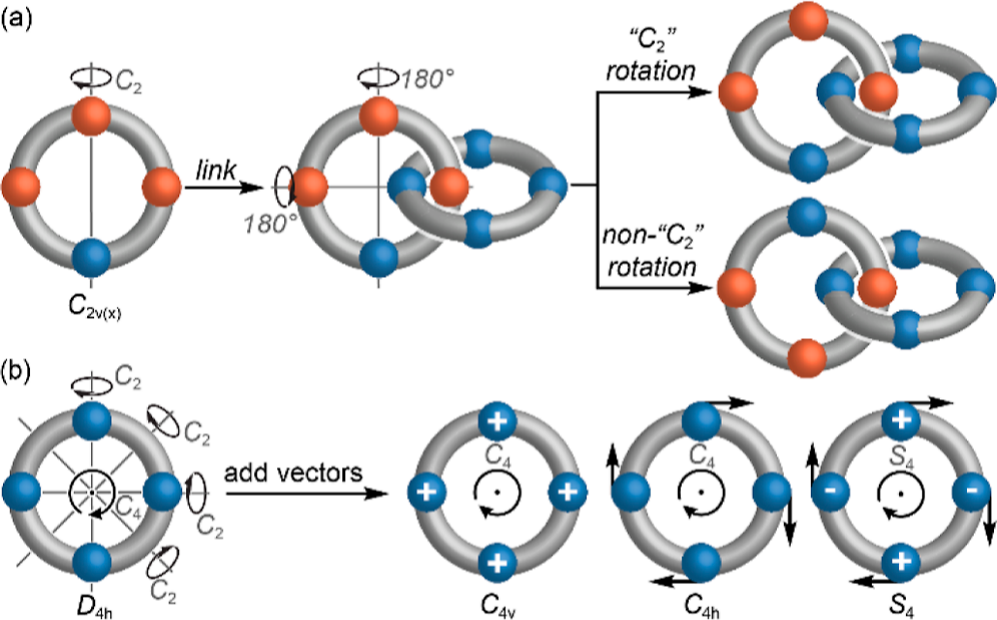
(a) Schematic demonstration that the *C*_2(x)_(11) symmetry operation of a non-interlocked ring corresponds to the notional process of inverting the relative ring orientations in a [2]catenane; hence, any ring for which *C*_2(x)_ is a symmetry operation cannot give rise to conditional mechanical stereoisomers. (b) Conversion of a *D*_4*h*_ symmetric structure to rings of *C*_4v_, *C*_4h_, and *S*_4_ symmetry, which we propose to be representative of the complete set of oriented (*C*_*n*h_ and *S*_2*n*_) and facially dissymmetric (*C*_*n*v_) building blocks of catenane stereochemistry, by the addition of simple vectors (± refer to vectors projecting up/down, respectively, perpendicular to the plane of the ring).

We first recognize that the highest symmetry ring point group, *D*_∞h_, contains the achiral *D*_*n*d_, *C*_*n*h_, *C*_*n*v_, and *S*_2*n*_ subgroups and that therefore rings of these symmetries are the complete set of building blocks of catenane mechanical stereochemistry (see Supporting Information Section 1 for further discussion). Second, we recognize that any ring that has a *C*_2_-axis in the macrocycle plane [*C*_2(x)_]^11^ cannot give rise to a conditional mechanical stereogenic unit because this symmetry operation of the separated rings corresponds to the notional process of switching their relative orientations in the corresponding [2]catenane (Figure 1a). Although this observation appears obvious, to our knowledge, this is the first time it has been stated explicitly.^12^ Thus, we can discard rings of *D*_*n*d_, and *C*_2v(x)_ and *C*_2h(x)_ symmetry.^11,13,14^

The visually tractable *D*_4h_ point group contains the *C*_4v_, *C*_4h_, and *S*_4_ subgroups, representative of *C*_*n*v_, *C*_*n*h_, and *S*_2*n*_, and so we modified a *D*_4h_ ring to generate these structures by adding four equally spaced, equivalent vectors perpendicular and/or tangential to the ring plane in different relative orientations to highlight the key features of these achiral macrocycles (Figure 1b). Taking this approach, we find that to ensure that *C*_2(x)_ is not a symmetry operation of the ring, it must either be oriented (*C*_*n*h_ or *S*_2*n*_; characterized by vectors tangential to the ring circumference that define its direction), or facially dissymmetric (*C*_*n*v_; characterized by vectors perpendicular to the ring plane that differentiate its faces).

**Figure 2:**
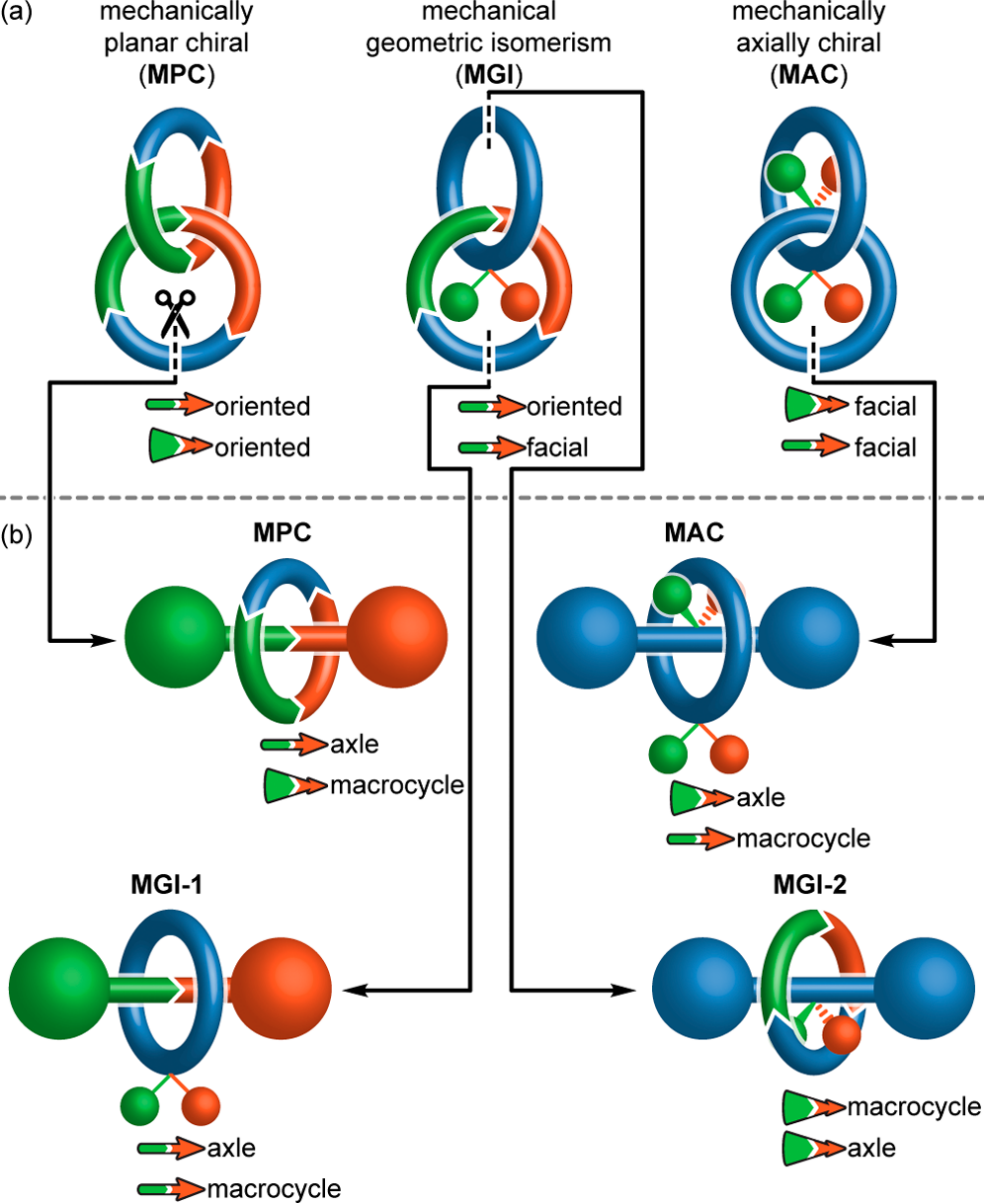
(a) Complete set of catenane mechanical stereogenic units that can be constructed from the archetypal rings identified (Figure 1) and their relationship with the (b) mechanical stereogenic units of rotaxanes via a notional ring-opening-and-stoppering operation, including the newly identified “type 2” rotaxane mechanical geometric unit. The vectors shown characterize their stereochemistry and their relationship to the components that gives rise to them defines the stereogenic unit.

The requirement for the rings of a [2]catenane to be oriented or facially dissymmetric for mechanical stereochemistry to arise is not a new observation; combining two oriented *C*_*n*h_ rings or two facially dissymmetric *C*_*n*v_ rings gives rise to the chiral catenanes originally identified by Wasserman and Frisch,^1^ illustrated here using rings of *C*_1h_ and *C*_1v_ symmetry,^15^ respectively (Figure 2a). The vectors associated with the orientation or facial dissymmetry of the individual rings can never become coplanar in the resultant catenanes, and thus, the stereochemistry of such structures can be defined using the resulting oriented skew lines.^16^ The skew lines lie parallel to the associated ring when two oriented rings are combined but perpendicular to the rings when two facially dissymmetric rings are combined, which provides robust definitions of the canonical mechanically planar chiral (MPC) and mechanically axially chiral (MAC) stereogenic units of [2]catenanes, respectively. Thus, the only surprising result from our analysis is that *S*_2*n*_ symmetric rings are oriented and thus give rise to a mechanical stereogenic unit, which to the best of our knowledge has not previously been noted. However, we suggest that combining two *S*_2*n*_ macrocycles (or a combination of *S*_2*n*_ and *C*_*n*h_ rings) gives rise to the MPC stereogenic unit, as defined by the orientation of the skew lines associated with the rings, rather than a new form of mechanical stereochemistry.^17^

Finally, combining one facially dissymmetric *C*_*n*v_ ring and one oriented *C*_*n*h_ (or *S*_2*n*_) ring results in an achiral structure because the associated skew lines can be made coplanar in the interlocked structure. However, two mechanical geometric isomers (MGI) are possible because the vectors can be arranged syn (*Z*_m_) or anti (*E*_m_) (Figure 2a).^18^

### Analyzing the Achiral Building Blocks of Rotaxanes Reveals the Final Mechanical Stereogenic Unit

The same analysis can be used to identify the axle point group symmetries that can give rise to mechanical stereochemistry in a rotaxane and thus the complete set of rotaxane stereogenic units (see Supporting Information Section 2). However, the same result is reached more intuitively by identifying that rotaxanes and catenanes are interconverted by a notional ring-opening-and-stoppering operation (Figure 2b), which, as previously noted, leads to the conclusion that MPC catenanes and rotaxanes are directly related,^6^ as are the MAC pair.^5a^ Once again, these rotaxane stereogenic units can be differentiated by considering the relative orientation of the skew lines that characterize their configuration; the MPC stereogenic unit of rotaxanes is defined as arising when the vector associated with the axle lies along its axis, whereas the MAC stereogenic unit arises when the vector associated with the axle is perpendicular to its axis. These axle vectors lie perpendicular to the vector associated with the ring when interlocked with oriented or facially dissymmetric rings, respectively.

It is when we turn to the MGI stereogenic unit of catenanes that we find a surprise. Because the two rings are distinct, there are two possible products of the opening-and-stoppering sequence, one of which is the canonical MGI rotaxane stereogenic unit identified by Schill, and the other is a previously overlooked form of rotaxane geometric isomerism. The former is characterized by the coplanar vectors associated with the two components lying parallel to the axle, whereas in the latter, these vectors lie perpendicular to the axle. We propose that the labels “type 1” and “type 2” are used to distinguish between the canonical and noncanonical geometric isomers of rotaxanes (MGI-1 and MGI-2, respectively), with the numeral assigned by the order in which they were identified.

### Catenane and Rotaxane Stereochemistry—Conclusions

Our simple, first-principles approach has allowed us to unambiguously identify and define all the possible conditional stereogenic units of rotaxanes and catenanes and confirm that, now that a previously overlooked MGI-2 rotaxane stereochemistry has been found, the pantheon of unique stereogenic units is complete. Based on this analysis, methods exist to stereoselectively synthesize all conditional mechanical stereogenic units of [2]catenanes and [2]rotaxanes apart from MGI-2 rotaxanes; although until 2014,^19^ chiral stationary phase high-performance liquid chromatography (HPLC) was required to produce enantioenriched samples of mechanically chiral molecules^20^ since this time, methodologies^21^ for the stereoselective synthesis of MPC^6,13,22,23^ and MAC^5^ catenanes and rotaxanes have been disclosed. Similarly, the first stereoselective synthesis of MGI-1 rotaxanes was reported in 2005^24^ using calixarene rings, and since then many examples based on cone-shaped macrocycles,^25^ and more recently simple prochiral^26^ rings,^5b,27^ have been reported. The corresponding MGI catenanes are less well studied but yield to similar strategies to the corresponding rotaxanes.^4,5b,28^

### Retrosynthetic Analysis of the “New” MGI-2 Stereogenic Unit

**Figure 3:**
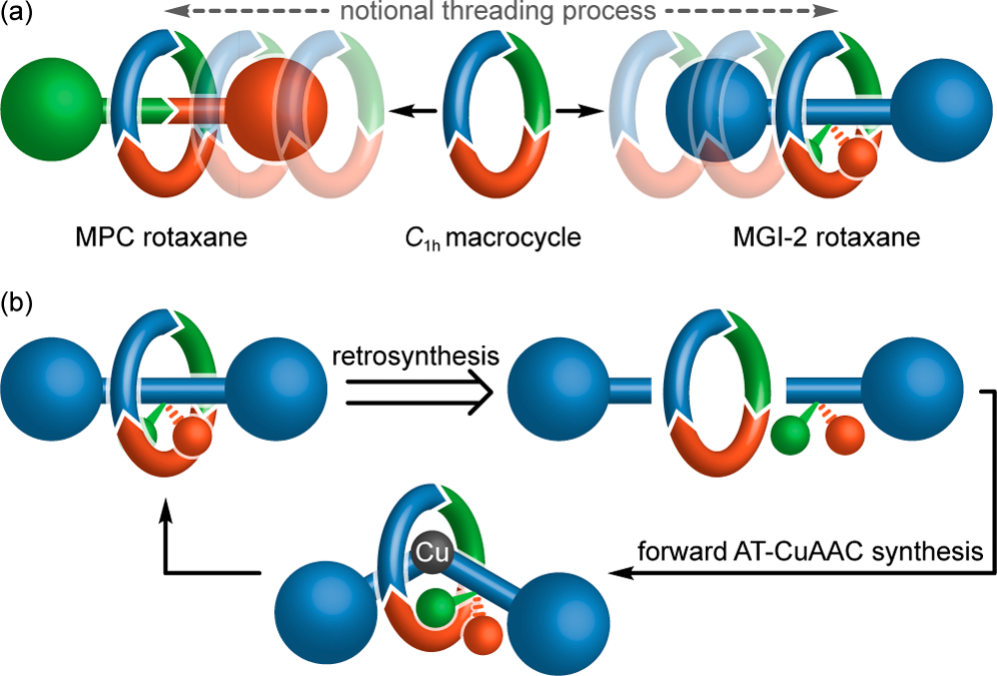
(a) Comparison of the MGI-2 and MPC stereogenic units highlighting the common challenge of selectively threading of an oriented ring onto an oriented or facially dissymmetric axle, respectively. (b) Retrosynthesis of the MGI-2 stereogenic unit using a direct AT-CuAAC approach. The forward reaction proceeds via two possible diastereomeric intermediates (one shown). Although one of the half-axle units is chiral, this is symmetrized in the forward reaction, and the same achiral, diastereomeric mixture is produced whether the starting material is enantiopure or racemic.

Having identified the MGI-2 stereogenic unit, we considered what strategies could be used for its selective synthesis. Notionally, the challenge in the synthesis of MGI-2 rotaxanes is the same as that of MPC rotaxanes—how to thread an oriented ring onto an axle with control over their relative orientation (Figure 3a). We previously achieved this for MPC rotaxanes^22a,22e^ using an active template^29^ Cu-mediated alkyne–azide cycloaddition (AT-CuAAC^30,31^) approach, in which the intermediates leading to the different enantiomers are diastereomeric due to a covalent chiral auxiliary. This analysis suggests that a similar approach is possible in the case of MGI-2 rotaxanes (Figure 3b). Although it may seem counterintuitive to synthesize the achiral MGI-2 stereogenic unit using chiral starting materials, it should be noted that almost regardless of where the prochiral axle is subdivided,^32^ a chiral starting material is produced. However, this is symmetrized during mechanical bond formation, so no additional auxiliary removal step is required. Furthermore, a racemic mixture of starting materials would lead to the same MGI-2 product mixture using this direct approach.

### Attempted Direct Synthesis of MGI-2 Rotaxanes **5**

**Scheme 1:**
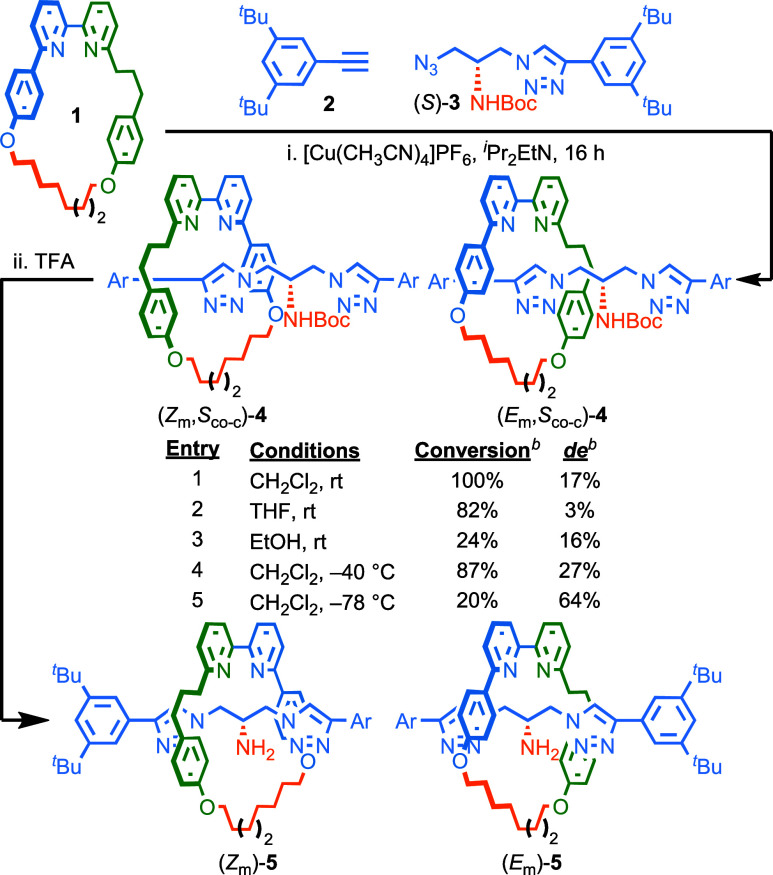
Poorly Selective Direct AT-CuAAC Synthesis of Type **2** Rotaxane Geometric Isomers **5** via Chiral Diastereomers **4** Reagents and conditions: (i) **1** (1 equiv), **2** (1.1 equiv), (*S*)-**3** (1.1 equiv), [Cu(CH_3_CN)_4_]PF_6_ (0.97 equiv), ^*i*^Pr_2_EtN (2 equiv). (ii) TFA, CH_2_Cl_2_, rt, 1 h. ^*b*^Determined by ^1^H NMR analysis of the crude reaction product. Ar = 3,5-di-^*t*^Bu-C_6_H_3_.

Thus, we initially attempted the synthesis of a rotaxane expressing the MGI-2 stereogenic unit using a stepwise AT-CuAAC approach. Reaction of oriented macrocycle **1**,^33^ alkyne **2**, and serine-based azide (*S*)-**3** under our AT-CuAAC conditions^22a^ in CH_2_Cl_2_ gave rotaxane **4** as a mixture of diastereomers (17% *de*,^34^Scheme 1, entry 1) that differ in their MGI-2 configuration but have the same co-conformational covalent configuration, which is fixed due to the bulky NHBoc unit that prevents the macrocycle from shuttling between the two triazole compartments.

The same reaction in THF (entry 2) or EtOH (entry 3) gave lower selectivity (3 and 16% *de*, respectively), whereas lower temperatures (entries 4 and 5) gave increased selectivity at the expense of reduced conversion. Unfortunately, the (*Z*_m_,*S*_co-c_)-**4** and (*E*_m_,*S*_co-c_)-**4** diastereomers proved hard to separate; the best we could achieve was a 59% *de* sample starting from a 17% *de* sample after several rounds of chromatography. We were also unable to separate rotaxanes **5**, which express only the MGI-2 stereogenic unit, obtained by removal of the Boc group from the mixture of rotaxanes **4**.

The disappointing stereoselectivity in the formation of rotaxanes **4** is perhaps unsurprising; we have previously identified that AT-CuAAC auxiliary approaches to MPC rotaxanes, which are analogous to the direct approach to the achiral MGI-2 stereogenic units presented here, only proceed efficiently when a sterically hindered α-chiral azide half-axle is used.^22a,22e^ This is hard to realize practically in the case of the MGI-2 stereogenic unit as it would nominally require iterative CuAAC couplings of a 1,1-bis-azide synthon. Thus, we returned to our comparison of the MPC and MGI-2 stereogenic units and recognized that our chiral interlocking auxiliary strategy,^19^ which reliably loads macrocycle **1** onto the axle of almost any rotaxane in a specific orientation that is determined by the absolute stereochemistry of the amino acid-derived azide used, corresponds to the desired notional oriented threading process (Figure 3a).

### Stereoselective Synthesis of MGI-2 Rotaxanes **11** Using an Interlocking Auxiliary Approach

**Scheme 2:**
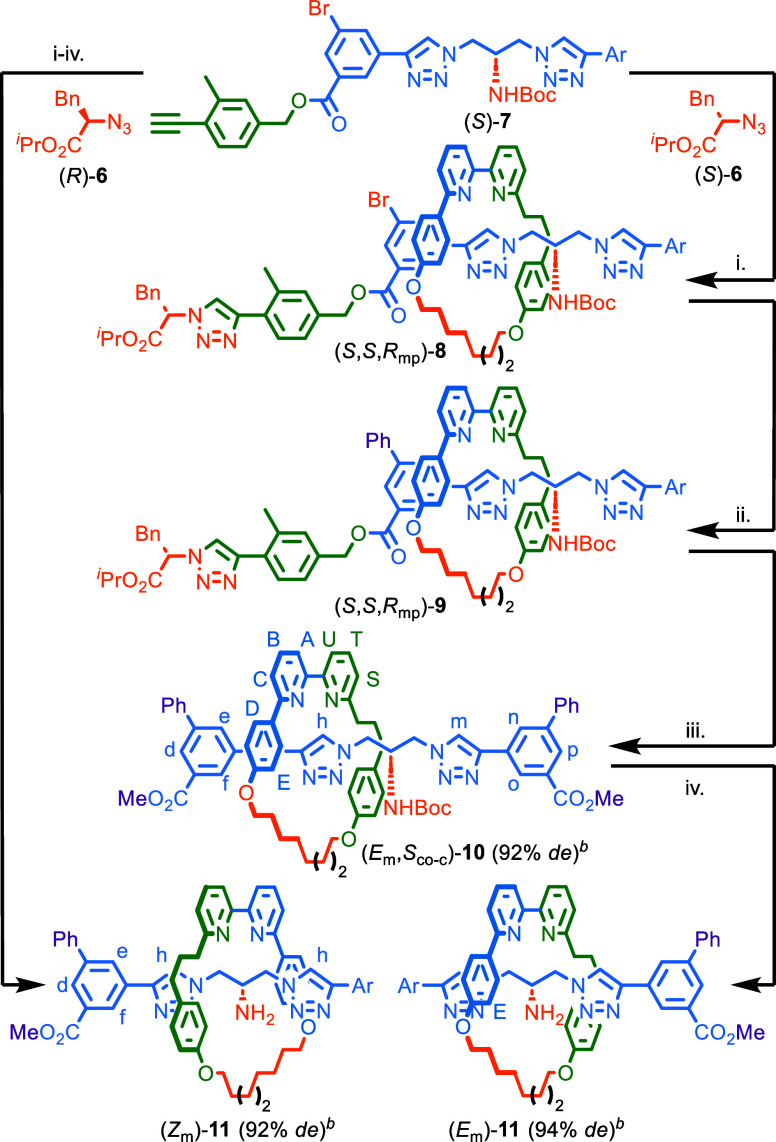
Chiral Interlocking Auxiliary Synthesis of MGI-2 Rotaxanes **10** and **11** Reagents and conditions: (i) **1** (1 equiv),**6** (1.1 equiv), (*S*)-**7** (1.1 equiv), [Cu(CH_3_CN)_4_]PF_6_ (0.99 equiv), ^*i*^Pr_2_Et (2 equiv), CH_2_Cl_2_, rt, 16 h. (ii) PhB(OH)_2_, Pd(PPh_3_)_4_, K_2_CO_3_, acetone-^*i*^PrOH-H_2_O (2:1:1), 60 °C, 3 h. (iii) K_2_CO_3_, CH_2_Cl_2_-MeOH, rt, 3 h. (iv) TFA, CH_2_Cl_2_, rt, 1 h. *^b^*Determined by ^1^H NMR analysis. Ar = 3-CO_2_Me-5-Ph-C_6_H_3_.

Coupling of azide (*S*)-**6** with *o*-Me acetylene half-axle (*S*)-**7** in the presence of macrocycle **1** gave rotaxane (*S*,*S*,*R*_mp_)-**8** (Scheme 2), in which the macrocycle preferentially encircles the less hindered triazole unit, in excellent stereoselectivity (94% *de*). Subsequent Suzuki coupling produced rotaxane (*S*,*S*,*R*_mp_)-**9** as the major co-conformational isomer. Transesterification with MeOH gave rotaxane (*E*_m_,*S*_co-c_)-**10**, which contains an MGI-2 and a co-conformational stereogenic unit, in excellent stereopurity (92% *de*).^35^ Removal of the Boc group provided rotaxane (*E*_m_)-**11** that expresses only MGI-2 stereochemistry, again in high stereopurity (94% *de*). The same synthesis but starting from (*R*)-**6** and (*S*)-**7** gave (*Z*_m_,*S*_co-c_)-**10** (94% *de*), which was then converted to (*Z*_m_)-**11** (92% *de*).

We note that the absolute MGI-2 configuration of the product of this interlocking auxiliary approach depends not on the enantiomer of chiral auxiliary **6** used but instead on the diastereomer of the axle produced in the first coupling step; the reaction of the (*S*)-**6**/(*S*)-**7** (Scheme 1) or (*R*)-**6**/(*R*)-**7** (not shown) pairs to give (*S*,*S*,*R*_mp_)-**8** or (*R*,*R*,*S*_mp_)-**8**, respectively, would both ultimately produce (*E*_m_)-**11**. However, unlike in the case of a direct AT-CuAAC synthesis (Figure 3b and Scheme 1), a racemic mixture of starting materials would always lead to an equal mixture of MGI-2 isomers by using this approach.

### Analysis of Rotaxanes **10** and **11**

**Figure 4:**
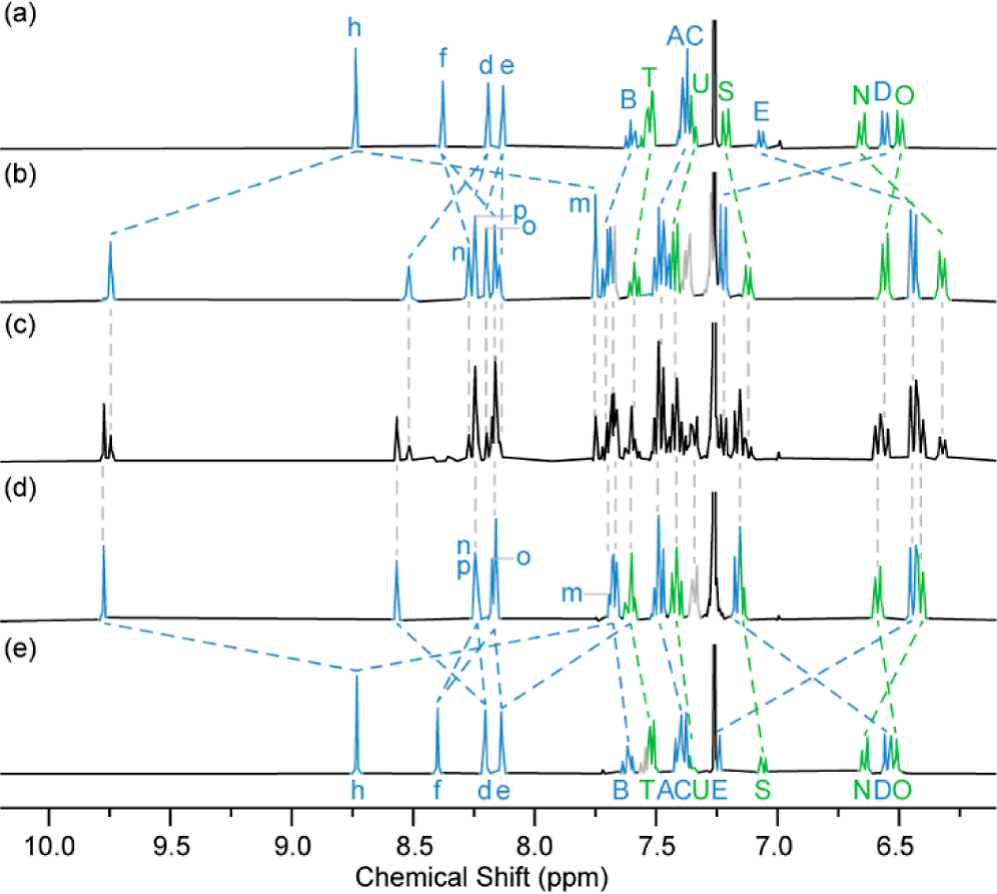
Partial ^1^H NMR (400 MHz, CDCl_3_, 298 K) spectra of (a) (*Z*_m_)-**11** (92% *de*), (b) (*Z*_m_,*S*_co-c_)-**10** (94% *de*), (c) **10** (16% *de*, obtained by a direct AT-CuAAC coupling, see Supporting Information Section 4), and (d) (*E*_m_,*S*_co-c_)-**10** (92% *de*) (e) (*E*_m_)-**11** (94% *de*). Peak assignment and colors are the same as shown in Scheme 2.

Rotaxanes (*E*_m_,*S*_co-c_)-**10** and (*Z*_m_,*S*_co-c_)-**10** have distinct ^1^H NMR spectra (Figure 4b,d respectively) that each correspond to one of the inseparable isomers obtained using a direct AT-CuAAC approach to the same molecules (*c*.*f*., **4**, see Supporting Information Section 4) (Figure 4c). The ^1^H NMR spectra of the two geometric isomers of rotaxanes **11** (Figure 4a,e) are also distinct from one another, but they suggest molecules of much higher symmetry than rotaxanes **10**. This is not because the macrocycle preferentially encircles the amine unit; the high chemical shift of triazole protons H_*h*_ in rotaxanes **11** is consistent with the macrocycle exchanging between the two triazole containing compartments where it engages in a C–H···N H-bond.^36^ Instead, and in contrast with MAC rotaxanes,^5^ based on a similar prochiral axle, the two co-conformers of rotaxanes **11** are enantiomeric and so the H_*h*_ pair are enantiotopic and isochronous.

**Scheme 3:**
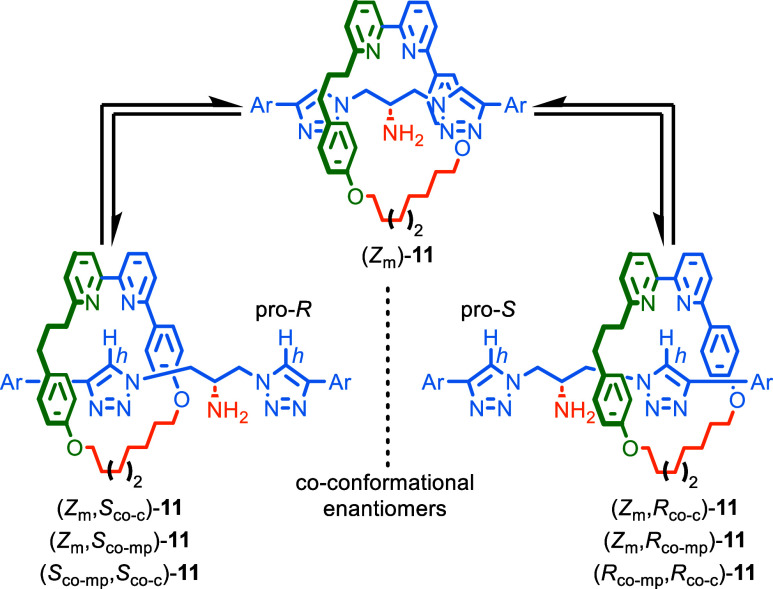
Co-Conformational Exchange between the Enantiomeric Co-Conformations of Rotaxane (*Z*_m_)-**11** Highlighting the Different Stereochemical Labels that Can Be Applied to Fully Assign Their Absolute Stereochemistry Ar = 3-CO_2_Me-5-Ph-C_6_H_3_.

Interestingly, the absolute stereochemistry of the co-conformations of rotaxane **11** (Scheme 3), and that of static diastereomers **4** and **10**, can be fully described using two of three possible stereolabels, of which we strongly prefer the co-conformational covalent and MGI-2 description as this captures the desymmetrization of the axle component upon shuttling and the sole fixed stereogenic unit of the molecule. The co-conformational MPC/MGI-2 description fails to capture the former, and the co-conformational covalent/co-conformational MPC description obscures the fixed MGI-2 unit, with both stereolabels inverting under co-conformational exchange (see Supporting Information Section 7 for an extended discussion).

## Conclusions

In conclusion, we have presented a simple stereochemical analysis to identify the complete set of [2]catenane and [2]rotaxane mechanical stereoisomers and, in doing so, recognized a new form of rotaxane geometric isomerism. Furthermore, retrosynthetic analysis of the noncanonical type 2 geometric stereogenic unit allowed us to make the link to the mechanical planar chiral stereogenic unit of rotaxanes, which led ultimately to the first stereoselective synthesis of such molecules.

Now that all of the mechanical stereogenic units of simple [2]catenanes and [2]rotaxanes have been delineated and concepts developed to allow their stereoselective synthesis,^4−6,21−25,28^ it is reasonable to propose that, 62 years after such systems were first discussed,^1^ we have finally reached the end of the beginning of the study of mechanical stereochemistry. Such molecules have already been used as the basis of molecular machines,^14d^ enantioselective sensors^37^ and catalysts,^38^ and chiroptical switches,^39^ work which will only accelerate as methods to synthesize them improve. Moreover, we suggest it is time now to set our sights beyond these simple structures and develop methodologies for the systematic synthesis of structures whose stereochemistry arises due to the presence of additional crossing points^40^ or larger numbers of interlocked components^41^ so that the potential benefits of such architectures can also be explored.

## Data Availability

Data (characterization data for reported compounds) is available from the University of Birmingham UBIRA eData repository at https://doi.org/10.25500/edata.bham.00001074.

## Supporting Information Available

Procedures and full characterization data (NMR, MS, CD, SCXRD, HPLC as appropriate) for all novel compounds (PDF)
