# Supplementary material for: The Final Stereogenic Unit of [2]Rotaxanes: Type 2 Geometric Isomers
Source: J Am Chem Soc. 2024 Mar 18;146(12):8472–9. doi: 10.1021/jacs.3c14594 (PMC10979452; doi:10.1021/jacs.3c14594)
Supplement: Supplementary file 1 — ja3c14594_si_001.pdf [file ja3c14594_si_001.pdf]

Electronic Supporting Information

The Final Stereogenic Unit of [2]Rotaxanes: Type 2 Geometric Isomers

Andrea Savoini,[1,†] Peter R. Gallagher,[1,†] Abed Saady,[1,2,†] and Stephen M. Goldup[1,2,*]

[1] Department of Chemistry, University of Southampton, Highfield, Southampton, SO17 1BJ
[2] School of Chemistry, University of Birmingham, University Rd W, Birmingham B15 2TT
[†]These authors contributed equally
*s.m.goldup@bham.ac.uk

1. Stereochemical analysis to identify the fundamental catenane stereogenic units **3**
1.1 The achiral ring point groups – the building blocks of mechanically chiral catenanes 3
1.2 The stereogenic units of [2]catenanes 5
2. Stereochemical analysis to identify the fundamental rotaxane stereogenic units **8**
1.3 The achiral axles 8
1.4 The stereogenic units of rotaxanes 10
3. General Experimental Information **13**
4. Synthesis of rotaxane 5 and associated compounds (Scheme 1) **15**
1.5 NHBoc Rotaxanes 4 15
1.6 Amine Rotaxanes 5 20
5. Synthesis of rotaxane 11 and associated compounds (Scheme 2) **25**
1.7 Rotaxanes (E_{m},S_{co-c})-10 and (Z_{m},S_{co-c})-10 from (*S*)-S6 40
1.8 Alkyne (*S*)-13 53
1.9 Rotaxane (E_{m},S_{co-c})-10 57
1.10 Rotaxane (E_{m})-11 62
1.11 Rotaxane (Z_{m},S_{co-c})-10 67
1.12 Rotaxane (Z_{m})-11 72
6. Absolute Stereochemistry of Type 2 Mechanical Geometric Isomers **76**
7. Rotaxanes 4, 5, 10 and 11 – three possible sets of stereodescriptors **78**
8. References **79**

1. Stereochemical analysis to identify the fundamental catenane stereogenic units

1.1 The achiral ring point groups – the building blocks of mechanically chiral catenanes

Conditional mechanical stereochemistry arises in [2]catenanes when two achiral rings are interlocked such that the resulting structure expresses stereochemistry, even in its highest symmetry representation, that is invariant with co-conformational motion. If the stereochemistry of the catenane varies with co-conformation, a co-conformational stereogenic unit is present, which can be either covalent, if the position of one ring desymmetrizes the other such that a pro-stereogenic unit becomes stereogenic, or mechanical if the position of one ring desymmetrises the other such that a mechanical stereogenic unit arises.

The highest possible symmetry of a macrocycle is $D_{\infty h}$, which is easily represented by a simple ring (Figure S1). The key symmetry properties of this point group are a principal rotation axis perpendicular to the ring plane, a horizontal mirror plane perpendicular to the principal axis, and an infinite number of C_2 axes in the plane of the ring and an infinite number of reflection planes perpendicular to the ring plane. Interlocking two $D_{\infty h}$ rings does not result in a fixed mechanical stereogenic unit; the highest symmetry representation of the corresponding catenane has achiral D_{2d} point group symmetry. It should be noted that such structures do express co-conformational stereochemistry; when the two rings are not perpendicular to one another, a co-conformational helical stereogenic unit is present.

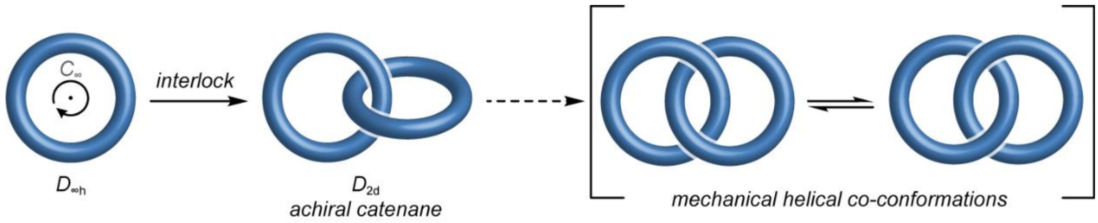

Figure S1: Schematic representation of a $D_{\infty h}$ ring and the corresponding catenane highlighting the lack of conditional mechanical stereochemistry but the potential for co-conformational stereochemistry.

As discussed in the manuscript, the $D_{\infty h}$ point group contains the D_{nd}, C_{nh}, C_{nv} and S_{2n} subgroups, which therefor represent the achiral point group symmetries possible for macrocycles and are thus the candidate building blocks of catenane mechanical stereochemistry. Given that mechanical stereoisomers are related by changing the relative orientation of the two rings, which corresponds to notional process of rotating one ring by 180° through the other about an axis in the plane of the ring, we can then eliminate D_{nd}, $C_{2h(x)}$ and $C_{2v(x)}$ as this process corresponds to a symmetry operation of such rings, leaving the structure unchanged.

This leaves us with $C_{nh(z)}$, $C_{nv(z)}$ and S_{2n} symmetric rings as the candidate building blocks of catenane stereochemistry. To make this problem visually tractable, we considered C_{4h}, C_{4v} and S_4, which can be derived from a D_{4h} symmetric structure by addition of sets of 4 equivalent vectors either tangential or perpendicular to the ring.

From D_{4h} to C_{4h}. The C_{4h} point group is derived from D_{4h} if the structure is modified such that the reflection planes perpendicular to the ring are removed. This can be achieved simply by adding four equivalent, equidistant vectors evenly distributed around the rotation axis in the plane of the ring, which for convenience we represent as tangents at the circumference of the macrocycle, which ensures that $C_{2(x)}$ symmetry is lost. This is a general property of macrocycles in the C_{nh} point group – they can all be described as "oriented". By oriented we mean

that as the ring is rotated about the principal axis an observer would see all the vectors pointing in the same direction (left or right) as they pass in front of them. We note that C_{1h} is more properly named as $C_{s(xy)}$ (z-axis is defined as perpendicular to the ring) with the orientation defining the mirror plane as in the plane of the ring. We prefer to use $C_{1h(z)}$ as it emphasises the family (C_{nh}) to which these macrocycles belong.

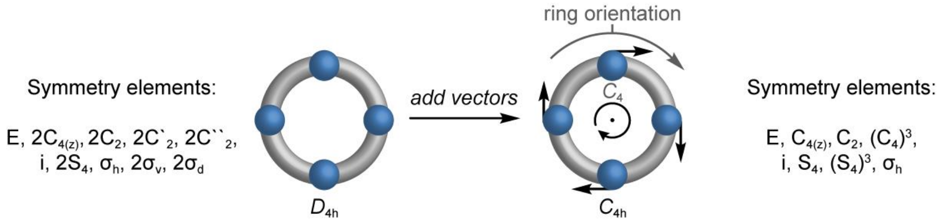

Figure S2: Schematic representation of the descent in symmetry from D_{4h} to C_{4h}.

Chemically, the C_{nh} symmetry can be achieved if the ring has a distinguishable sequence of atoms that, for C_{4h} symmetry, repeats four times, equally spaced around the principal axis. More commonly the rings used in rotaxanes and catenanes have C_{1h} symmetry.

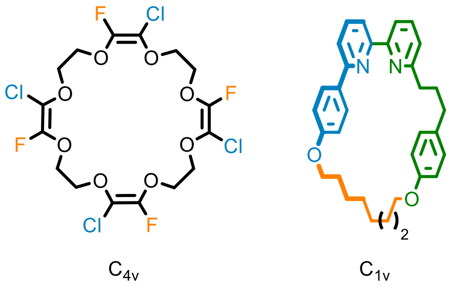

Figure S3: Chemical structure that corresponds to C_{4h} and C_{1h} symmetry.

From D_{4h} to C_{4v}. The C_{4v} point group is derived from D_{4h} by desymmetrizing the two faces of the ring without removing the reflection planes perpendicular to the ring or the principal rotation axis. This can be achieved simply by adding four equivalent vectors perpendicular to the plane of the ring and distributed evenly around the ring circumference. The same approach can be taken starting from any D_{nh} ring and thus, all macrocycles in the C_{nv} (principal axis perpendicular to the ring plane) point group can be described as "facially dissymmetric". Practically, facial dissymmetry means that as the ring is rotated about the principal axis an observer would see all of the vectors pointing in the same direction (up or down) as they pass in front of them. We note that C_{1v} is more properly named as $C_{s(xz)}$ (z-axis is defined as perpendicular to the ring) with the orientation defining the mirror plane as perpendicular to the ring. We prefer to use $C_{1v(z)}$ as it emphasises the family (C_{nv}) to which these macrocycles belong.

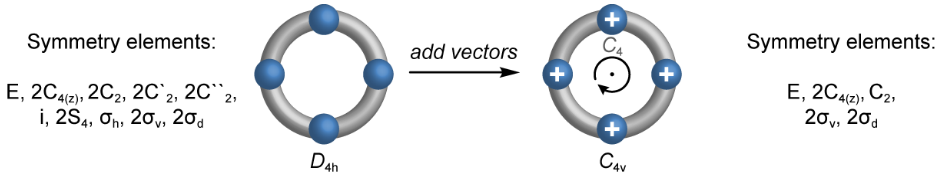

Figure S4: Schematic representation of the descent in symmetry from D_{4h} to C_{4v}.

Chemically, C_{nv} symmetry can be achieved if the ring contains prochiral centres paired either side of a σ_v reflection plane, which in the case of C_{4v} symmetry requires four such centres. More commonly the rings used in rotaxanes and catenanes have C_{1v} symmetry.

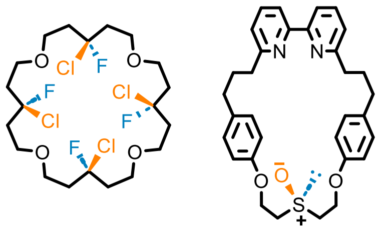

Figure S5: Chemical structure that corresponds to C_{4v} and C_{1v} symmetry

From D_{4h} to S_4 (Figure S6). The S_4 point group is derived from D_{4h} by desymmetrizing the structure both in the plane of the ring and facially such that the principal rotation axis is reduced from 4-fold to 2-fold and all reflection symmetry is lifted, but such that an improper rotation axis is maintained. Examining the symmetry properties of the S_4 structure, we see that if the ring is rotated about its principal axis an observer would see the vectors perpendicular to the ring alternating in direction (up then down, then up…) but the vectors parallel to the ring would be seen as pointing in the same direction (left or right) as they pass in front of and so rings of this symmetry can be described as oriented.

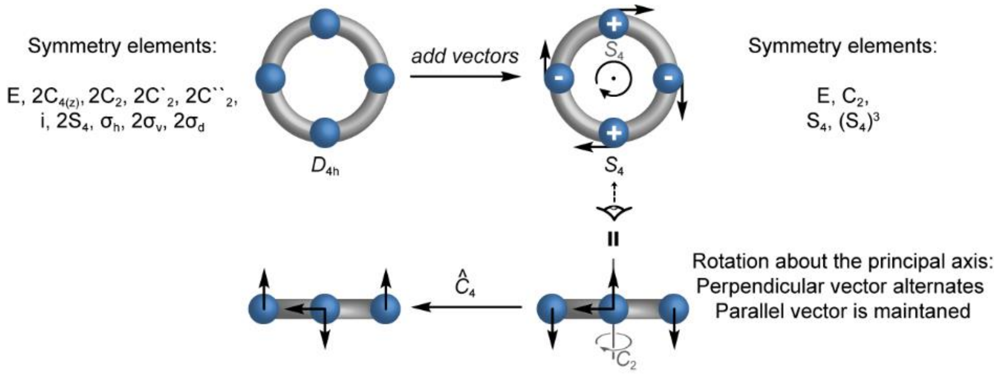

Figure S6: Schematic representation of the descent in symmetry from D_{4h} to S_4.

Chemically, S_{2n} symmetry can be achieved if the ring has 2n equivalent stereogenic centres arranged around the S_{2n} axis alternating in their configuration. For example, S_4 symmetry requires four such centres whereas S_2 symmetry (more properly C_i symmetry) requires two such centres. We could not identify any rotaxanes or catenanes composed of S_{2n} symmetric rings.

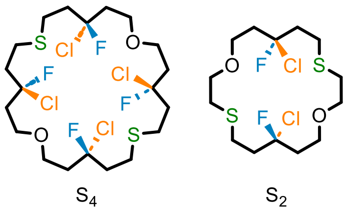

Figure S7: Chemical structure that corresponds to S_4 and S_2 symmetry

1.2 The stereogenic units of [2]catenanes

Having developed representative models of the achiral macrocycle point groups we considered the stereochemical outcome of interlocking to determine if the known set of mechanical stereogenic units is complete.

Interlocking two facially dissymmetric (C_{nv}) rings – the mechanical axial stereogenic unit: When two C_{nv} rings are interlocked, the resulting structure has no improper symmetry operations because the mechanical bond prevents the rings from becoming coplanar and thus there is no relative orientation in which the σ_v reflection plane of one

ring is a symmetry operation of the corresponding catenane. The facial dissymmetry of the rings can be used to characterize this stereogenic unit by observing that the vectors that differentiate the faces can never become co-planar and thus correspond to oriented skew lines. The mechanically axially chiral stereogenic unit is thus defined as that which arises when these skew lines are perpendicular to the rings.

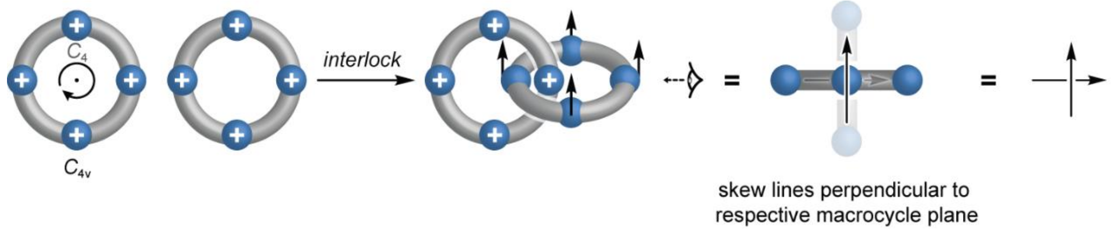

Figure S8: Schematic representation of the interlocking of two C_{4v} symmetric rings, which gives rise to a mechanically axially chiral catenane whose stereochemistry is characterized by the oriented skew lines of the rings lying perpendicular to one another and also the associated ring.

Interlocking two oriented rings (C_{nh} or S_{2n} symmetry) – the mechanically planar chiral stereogenic unit: When two C_{nh} rings are interlocked, the resulting structure has no improper symmetry operations because the mechanical bond prevents the rings from becoming coplanar and thus the is no relative orientation in which the σ_h reflection plane of one ring is a symmetry operation of the corresponding catenane. The orientation of the rings can be used to characterize this stereogenic unit by observing that the vectors that define the orientations can never become co-planar and thus correspond to oriented skew lines. The mechanically planar chiral stereogenic unit is thus defined as when these skew lines are parallel to the rings.

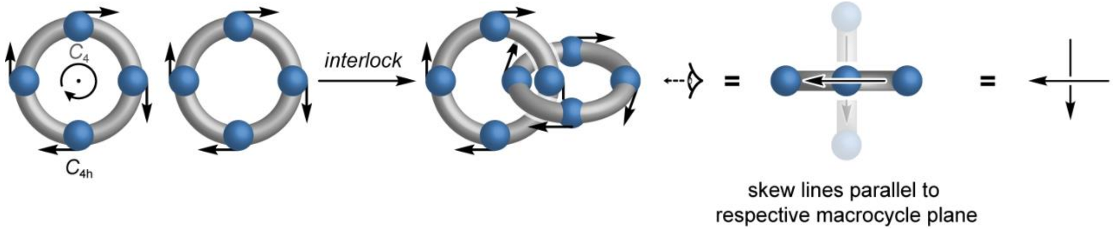

Figure S9: Schematic representation of the interlocking of two C_{4h} symmetric rings, which gives rise to a mechanically planar chiral catenane whose stereochemistry is characterized by the oriented skew lines of the rings lying perpendicular to one another and coplanar associated ring.

Similarly, when two S_{2n} rings are interlocked, the resulting structure has no improper symmetry operations because the improper S_n axis of one ring cannot be an operation of the corresponding catenane. The orientation of the rings can be used to characterize this stereogenic unit by observing that the vectors that define the ring orientations can never become co-planar and thus correspond to oriented skew lines. Because these skew lines are coplanar with the rings, we propose that interlocking S_{2n} rings results in a special case of the mechanically planar chiral stereogenic unit, rather than a new form of mechanical stereochemistry. We note that if one ring is rotated relative to the other, the skew line pair in the plane of the rings that define the mechanical planar stereogenic unit is unaltered but the skew lines that arise from the vector pair perpendicular to the rings inverts, and thus such structures display a dynamic co-conformational stereogenic unit as well.

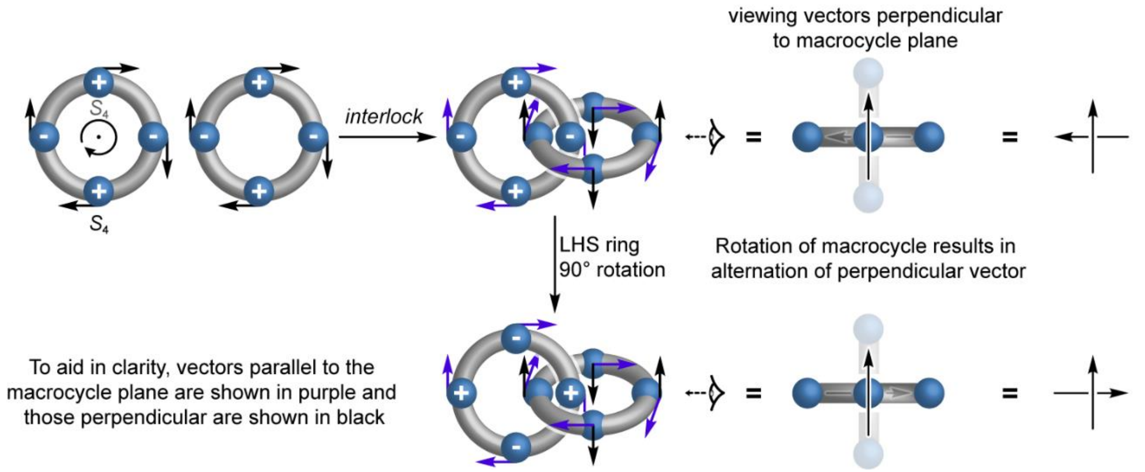

Figure S10: Schematic representation of the interlocking of two S_4 symmetric rings, which gives rise to a mechanically planar chiral catenane whose stereochemistry is characterized by the oriented skew lines of the rings lying perpendicular to one another and coplanar associated ring. Such structures also display co-conformational stereochemistry, which is characterized by the vectors perpendicular to the rings alternating in orientation with respect to the observer as they are rotated relative to one another.

Interlocking one oriented (C_{nh} or S_{2n}) and one facially dissymmetric (C_{nv}) ring – the geometric stereogenic unit of catenanes: When one oriented and one facially dissymmetric ring are interlocked the resulting structure expresses a geometric stereogenic unit because the vector(s) associated with the oriented ring in the plane of the macrocycle can be made co-planar with the vector(s) associated with facially dissymmetric macrocycle perpendicular to this ring. We note that the S_{2n}/C_{nv} pair also expresses a co-conformational mechanically axially chiral stereogenic unit, making the stereochemistry of these structure extremely rich.

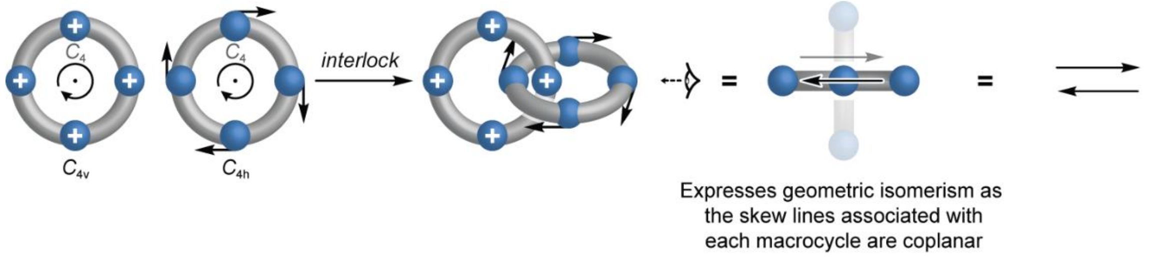

Figure S11: Schematic representation of the interlocking of one C_{4v} and one C_{4h} symmetric ring, which gives rise to a mechanical geometric stereogenic unit that is characterized by the oriented skew lines of the rings lying coplanar to one another in either a parallel or antiparallel fashion.

Summary. The above analysis has allowed us to confirm that the set of known conditional mechanical stereogenic units of [2]catenanes is complete. The only new result from the discussion above is the observation that the achiral S_{2n} symmetric rings are oriented and so can be considered as components of mechanically planar chiral or mechanical geometric isomers.

2. Stereochemical analysis to identify the fundamental rotaxane stereogenic units

1.3 The achiral axles

As with macrocycles, the highest symmetry axle has $D_{\infty h}$ point group symmetry which thus contains the D_{nd}, C_{nh}, C_{nv} and S_{2n} subgroups that we can consider to be our fundamental building blocks of rotaxane mechanical stereochemistry. Once again, given that rotaxane mechanical stereoisomers are related by changing the relative orientation of the ring and axle, which corresponds to notional process of rotating either the ring or axle by 180° through the other about an axis in the plane of the ring or perpendicular to the axle, we can eliminate D_{nd}, $C_{2h(x)}$ and $C_{2v(x)}$ symmetric rings and axles as this process corresponds to one of their symmetry operations, leaving the structure unchanged. This leaves us with the C_{nh}, C_{nv} and S_{2n} symmetric rings discussed above and C_{nh}, C_{nv} and S_{2n} symmetric axles as the candidate building blocks of rotaxane stereochemistry.

From $D_{\infty h}$ to C_{nv} (principal axis along the axle): The symmetry of the $D_{\infty h}$ axle can be reduced to $C_{\infty v}$ simply by adding a vector parallel to the long axis of the axle, which removes all $C_{2(x)}$ axes and horizontal mirror planes. Lower C_{1h} symmetry is achieved simply by allowing the axle to deviate from linearity. We note that C_{1h} is more properly named as $C_{s(xy)}$ (z-axis is defined as perpendicular to the ring) with the orientation defining the mirror plane as in the plane of the ring. We prefer to use C_{1h} as it emphasises the family (C_{nh}) to which these axles belong.

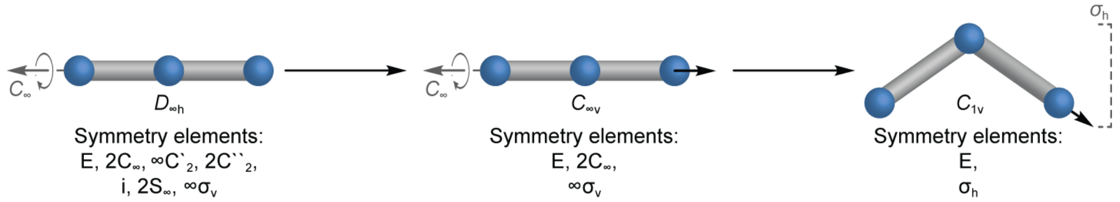

Figure S12: Schematic representation of the descent in symmetry from $D_{\infty h}$ to $C_{\infty v}$ and C_{1v}

Chemically, C_{nv} symmetry can be achieved if the two ends of the axle are different, which effectively ensures that the axle must have a defined sequence of atoms.

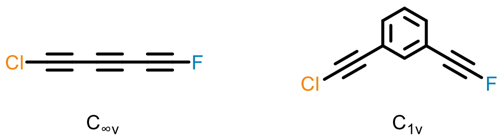

Figure S13: Chemical structure of axles that corresponds to $C_{\infty v}$ and C_{1v} symmetry

From $D_{\infty h}$ to C_{1h} (principal axis along the axle): Converting our $D_{\infty h}$ axle model to C_{nh} symmetry requires that we desymmetrize the structure such that a single reflection plane remains perpendicular to the axle and all $C_{2(x)}$ axes are removed. Although any value of n is technically possible, practically and of greatest chemical relevance, the easiest example to construct is C_{1h} (more properly $C_{s(xy)}$) by adding two non-colinear, non-equivalent vectors to the center of the axle. Alternatively, the same vectors can be added in the same orientation to both ends of the axle or a single vector added to the center of a non-linear axle. All of these achieve C_{1h} symmetry.

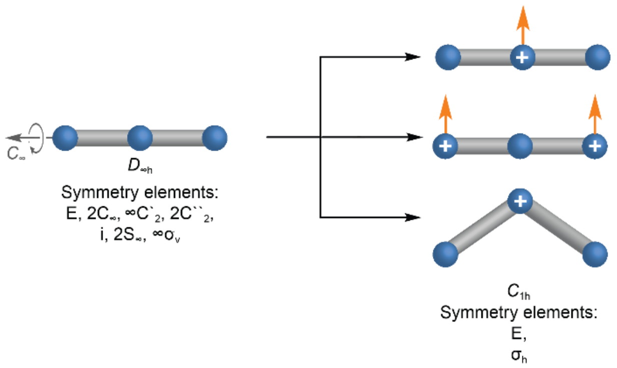

Figure S14: Schematic representation of the descent in symmetry from $D_{\infty h}$ to C_{1h}. The vectors in the plane of the page (orange) and perpendicular (+/-) are inequivalent and perpendicular to one another.

Chemically, the first example corresponds to an axle with a single prochiral centre in the middle of the axle, whereas the second corresponds to two equivalent stereogenic centers at either end of the axle such that the highest symmetry structure is meso. The non-linear axle model corresponds to a pro planar chiral axle.

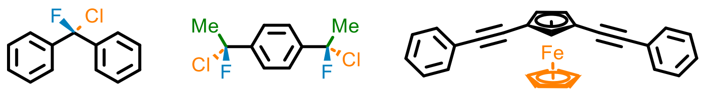

Figure S15: Chemical structure of axles that corresponds to C_{1h}

From $D_{\infty h}$ to S_{2n} (principal axis along the axle): Converting our $D_{\infty h}$ axle model to C_{nh} symmetry requires that we remove all reflection symmetry and $C_{2(x)}$ axes while maintaining a rotation and rotation-reflection parallel to the axle. Although any value of n is technically possible, practically and of greatest chemical relevance, the easiest example to construct is S_2, which is more properly referred to as C_i, by adding two non-colinear, non-equivalent vectors to either end of the axle such that they are related by a center of inversion.

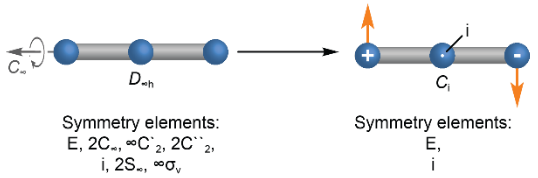

Figure S16: Schematic representation of the descent in symmetry from $D_{\infty h}$ to S_2 (more properly C_i). The vectors in the plane of the page (orange) and perpendicular (+/-) are inequivalent and perpendicular to one another.

Chemically this can be achieved by including equivalent stereogenic centers at either end of the axle, as in the case of the meso C_{1v} structure above but with a central unit of axle designed to lift the reflection symmetry (we note that the meso axle shown can also adopt a C_i conformation).

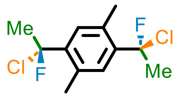

Figure S17: Chemical structure of an axle that corresponds to C_i symmetry

1.4 The stereogenic units of rotaxanes

"Orientation" and "facial dissymmetry" of axles: The achiral rings that we identified as the building blocks of catenane stereochemistry were described as either oriented (C_{nh} or S_{2n}) or facially dissymmetric (C_{nv}). Axles with C_{nv} symmetry can also be readily seen to be oriented – the vector used to desymmetrize the $D_{\infty h}$ model clearly defines the orientation of the axle.

Facial dissymmetry is harder to discern in an axle but is still possible to define for C_{1v} and C_i symmetric structures if we apply arbitrary rules that determine the orientation in which an axle should be viewed. For example, if we define that the C_{1v} structure based on a bent axle always be viewed with the ends of the axle pointing down, we see that the vector perpendicular to axle plane can either be pointing towards or away from the observer and that these views are interconverted by the C_2 operation that we have previously identified as equivalent to interchanging the relative orientation of the components in a [2]rotaxane.

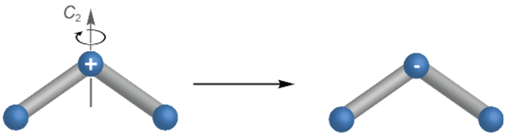

Figure S18: Schematic to demonstrate that a C_{1v} axle based on a non-linear structure can be described as facially dissymmetric if we define the axle to always be viewed with the apex pointing up.

Similarly, the C_{1v} structure based on two inequivalent vectors on the center of the axle can be defined as being viewed with the orange vector pointing up, upon which we see that the orthogonal vector can either point towards or away from the viewer. A similar rule can be applied to the meso C_{1v} structure.

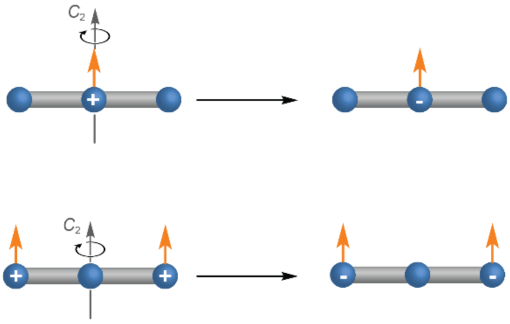

Figure S19: Schematic to demonstrate that C_{1v} axles based inequivalent perpendicular vectors either at the middle (prochiral) or ends (meso) can be defined as facially dissymmetric if we define that the orange vectors point up.

Finally, the C_i structure can be also defined as facially dissymmetric but in this case, we must define orientation to view the axle as that in which the left-most orange vector points upwards in the plane of the page with the axle face determined by the direction of the vector perpendicular to the page. Thus, applying a C_2 rotation about an axis perpendicular to the axle (which corresponds to switching the relative orientation of axle and macrocycle in a rotaxane) followed by a C_2 rotation along the axle axis (which corresponds to a pirouetting motion in a rotaxane) confirms that the leftmost perpendicular vector is inverted.

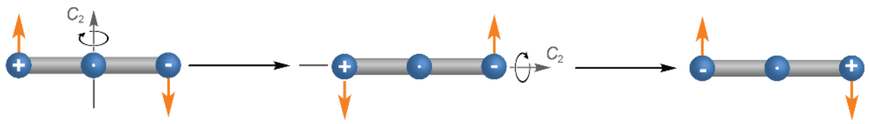

Figure S20: Note on direction that axle is viewed from

Interlocking an oriented (C_{nh} or S_{2n}) ring with an oriented (C_{nv}) axle – the mechanically planar chiral stereogenic unit: When an oriented ring encircles a C_{nv} axle, the resulting structure has no improper symmetry operations because the mechanical bond prevents the ring and axle from becoming coplanar and thus the is no relative orientation in which the σ_h reflection plane of the ring (in the C_{nh} case) is a symmetry operation of the corresponding rotaxane. The orientation of the ring and axle can be used to characterize this stereogenic unit by observing that the vectors that define the orientations of the components can never become co-planar and thus correspond to oriented skew lines. The mechanically planar chiral stereogenic unit of rotaxanes is thus defined as arising when these skew lines are parallel to the ring circumference and axle axis respectively.

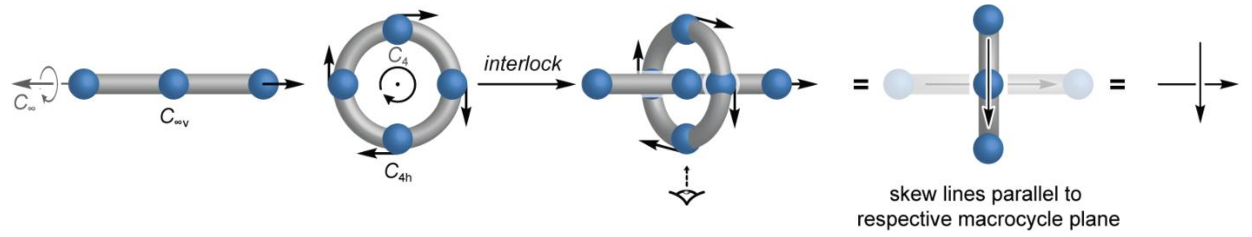

Figure S21: Schematic representation of the interlocking of a C_{4h} ring and a $C_{\infty v}$, which gives rise to a mechanical planar chiral stereogenic unit that is characterized by the associated oriented skew lines of each component lying along the circumference of the ring and along the axle axis respectively

Interlocking a facially dissymmetric (C_{nv}) ring with a facially dissymmetric (C_{nh} or S_{2n}) axle – the mechanically axially chiral stereogenic unit: When a C_{nv} ring encircles a C_{nh} or S_{2n} axle, the resulting structure has no improper symmetry operations because the mechanical bond prevents the ring and axle from becoming coplanar and thus there is no relative orientation in which the σ_v reflection planes of the ring or σ_h plane of the axle (in the C_{nh} case) are a symmetry operation of the corresponding rotaxane. The facial dissymmetry of the ring and axle (see above discussion) can be used to characterize this stereogenic unit by observing that the vectors that define the ring and axle faces can never become co-planar and thus correspond to oriented skew lines. The mechanically axially chiral stereogenic unit of rotaxanes is thus defined as when these skew lines are perpendicular to the ring and axle respectively.

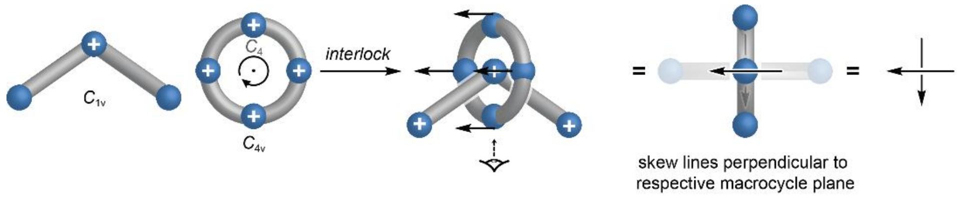

Figure S22: Schematic representation of the interlocking of a C_{4h} ring and a C_{1h} axle, which gives rise to a mechanical axial chiral stereogenic unit that is characterized by the associated oriented skew lines of each component lying perpendicular to the ring and axle respectively

Interlocking a facially dissymmetric (C_{nv}) ring with an oriented (C_{nv}) axle – the type 1 mechanical geometric stereogenic unit: When a C_{nv} ring encircles a C_{nv} axle, the resulting structure is achiral because it is possible for the σ_v reflection planes of the ring and axle to be made coincident. However, the co-planar vectors associated with the components can either be arranged parallel or antiparallel, giving rise to a type 1 mechanical geometric stereogenic unit, which we propose is characterized by the vectors associated with the components lying parallel to the axle.

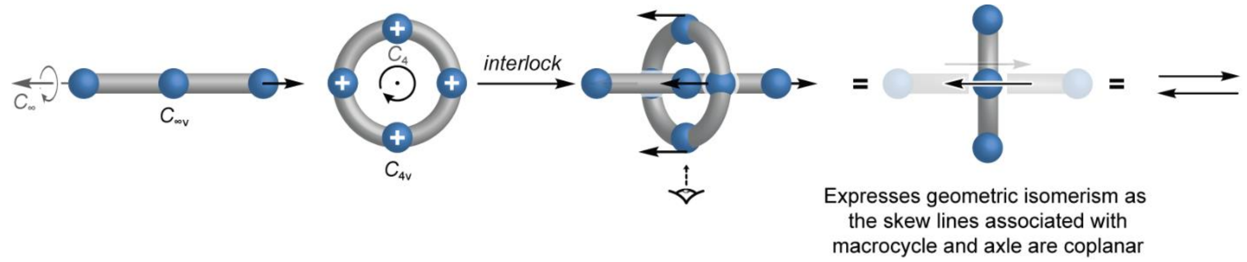

Figure S23: Schematic representation of the interlocking of a C_{4v} ring and a $C_{\infty v}$ axle, which gives rise to a type 1 mechanical geometric stereogenic unit that is characterized by the associated oriented skew lines of both components lying parallel to the axle axis

Interlocking an oriented (C_{nh} or S_{2n}) ring with a facially dissymmetric (C_{nh} or S_{2n}) axle – the type 2 mechanical geometric stereogenic unit: When a facially dissymmetric axle is encircled by an oriented ring, the resulting structure is achiral because it is possible for the σ_h reflection planes of the ring and axle (C_{nh} combination) to be made coincident. However, the co-planar vectors associated with the components can either be arranged parallel or antiparallel, giving rise to a type 2 mechanical geometric stereogenic unit, which we propose is characterized by the vectors associated with the components lying perpendicular to the axle.

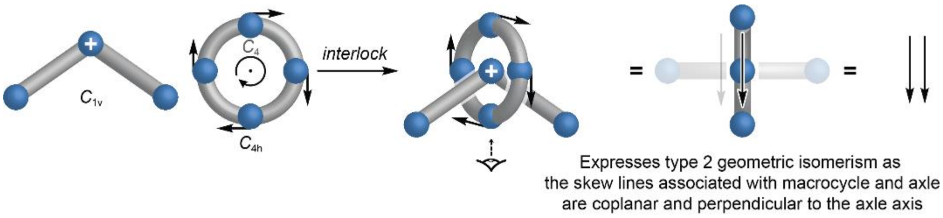

Figure S24: Schematic representation of the interlocking of a C_{4h} ring and a C_{1h} axle, which gives rise to a type 2 mechanical geometric stereogenic unit that is characterized by the associated oriented skew lines of both components lying perpendicular to the axle axis

Summary. The above analysis has allowed us to identify the missing type 2 mechanical geometric stereogenic unit of rotaxanes and also that this is the last stereogenic unit to be found. In addition, we have identified that S_{2n} symmetric axles, of which the most chemically relevant is S_2 symmetry (more properly C_i), are facially dissymmetric and so can give rise to mechanical axial or type 2 mechanical geometric stereochemistry.

3. General Experimental Information

Unless otherwise stated, all reagents were purchased from commercial sources (Acros Organics, Alfa Aesar, Fisher Scientific, FluoroChem, Sigma Aldrich and VWR) and used without further purification. $[Cu(CH_3CN)_4]PF_6$ was prepared as described by Pigorsch and Köckerling.[1] Anhydrous solvents were purchased from Acros Organics. Petrol refers to the fraction of petroleum ether boiling in the range 40-60 °C. IPA refers to isopropanol. THF refers to tetrahydrofuran. EDTA-NH_3 solution refers to an aqueous solution of NH_3 (17% w/w) saturated with sodium-ethylenediaminetetraacetate. $CDCl_3$ (without stabilising agent) was distilled over $CaCl_2$ and K_2CO_3 prior to use. Unless otherwise stated, all reaction mixtures were performed in oven dried glassware under an inert N_2 atmosphere with purchased anhydrous solvents. Unless otherwise stated experiments carried out in sealed vessels were performed in CEM microwave vials, with crimped aluminium caps, with PTFE septa. Young's tap vessels and Schlenk techniques were used where specified.

Flash column chromatography was performed using Biotage Isolera-4 or Isolera-1 automated chromatography system. SiO_2 cartridges were purchased commercially Biotage (SNAP or ZIP (50 µm), or Sfär (60 µm) irregular silica, default flow rates). Neutralised SiO_2 refers to ZIP cartridges which were eluted with petrol-NEt_3 (99 : 1, 5 column volumes), followed by petrol (5 column volumes). Analytical TLC was performed on pre-coated silica gel plates on aluminum (0.25 mm thick, 60F254, Merck, Germany) and observed under UV light (254 nm) or visualised with $KMnO_4$ stain.

All melting points were determined using a Griffin apparatus. NMR spectra were recorded on Bruker AV400 or AV500 instrument, at a constant temperature of 298 K. Chemical shifts are reported in parts per million from low to high field and referenced to residual solvent. Coupling constants (*J*) are reported in Hertz (Hz). Standard abbreviations indicating multiplicity were used as follows: m = multiplet, quint = quintet, q = quartet, t = triplet, d = doublet, s = singlet, app. = apparent, br = broad, sept = septet. Signal assignment was carried out using 2D NMR methods (COSY, NOESY, HSQC or HMBC) where necessary. In some cases, complex multiplets with multiple contributing proton signals, exact assignment was not possible. In interlocked compounds, all proton signals corresponding to axle components are in lower case, and all proton signals corresponding to the macrocycle components are in upper case.

Many of the signals analysed to determine diastereopurity were close in ppm and/or broad, which limited the potential to use Q-NMR methodology (https://nmrweb.chem.ox.ac.uk/Data/Sites/70/userfiles/pdfs/quantitative-nmr.pdf). For this reason, we systematically applied the "peak integration" function implemented in MestReNova (v11.0.4, Mestrelab Research S. L.) combined with the GSD peak modelling function (4 rounds of refinement, optimised for broad peaks), which has been shown to be comparable in accuracy to sum integration even when peaks are overlapping (qGSD - quantitative Global Spectral Deconvolution - Mestrelab Resources). Prior to integration, the default polynomial baseline correction was applied. Where possible, the values obtained were improved by comparison of multiple signals. The corresponding values obtained by sum integration are provided for comparison. Residual intensity is included for all peak integrations. Integral curves are included for sum integrations. Full details are included in the captions of the corresponding spectra.

Low resolution mass spectrometry was carried out by the mass spectrometry services at University of Southampton (Waters TQD mass spectrometer equipped with a triple quadrupole analyser with UHPLC injection [BEH C18 column; CH_3CN -H_2O gradient {0.2% formic acid}]). High resolution mass spectrometry was either carried out by the mass spectrometry services at the University of Southampton (MaXis, Bruker Daltonics, with a Time of Flight (TOF) analyser; samples were introduced to the mass spectrometer via a Dionex Ultimate 3000 autosampler and uHPLC pump in a gradient of 20% CH_3CN in *n*-hexane to 100% acetonitrile (0.2% formic acid) over 5-10 min at 0.6 mL/min; column: Acquity UPLC BEH C18 (Waters) 1.7 micron 50 × 2.1mm) or services at University of Birmingham (Waters Synapt G2-S mass spectrometer fitted with a TOF detector).

Circular dichroism spectra were either acquired on an Applied Photo-physics Chirascan spectropolarimeter, recorded using Applied Photophysics software Ver. 4.2.0 or a Jasco J-1500 spectropolarimeter in dried spectroscopic grade $CHCl_3$ in a quartz cell of 1 cm path length, at a temperature of 293 K.

Stereochemical purity was determined by Chiral Stationary Phase HPLC on a Waters Acquity Arc Instrument at 303 K, with *n*-hexane-^{i}PrOH or *n*-hexane-EtOH isocratic eluents. A RegisCell (tris-(3,5-dimethylphenyl) carbamoyl cellulose stationary phase) column was used (5 micron, column dimensions 25 cm x 4.6 mm).

The following compounds were synthetized according to literature procedures: **1**,[2] **2**,[3] (*S*)-**3**[4], (*S*)-**1**[4], **S7**,[2] **S10**,[5] **6** ((*S*)-**6** and (*R*)-**6**)).[6]

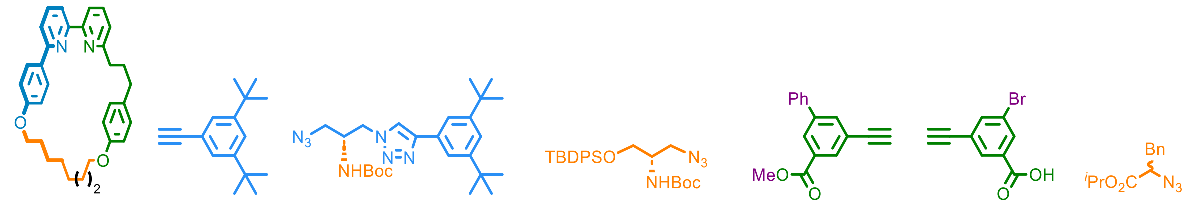

1 **2** (*S*)-**3** (*S*)-**S1** **S7** **S10** **6**

4. Synthesis of rotaxane 5 and associated compounds (Scheme 1)

1.5 NHBoc Rotaxanes 4

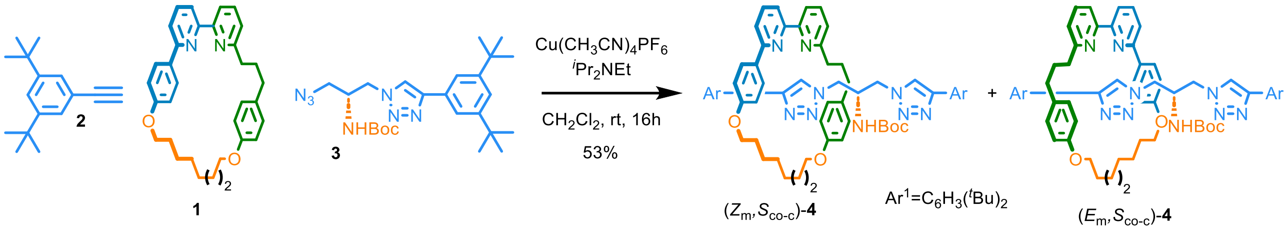

In a CEM vial were added **2** (9.4 mg, 43.9 µmol), (*S*)-**3** (20.0 mg, 43.9 µmol), **1** (19.7 mg, 40.2 µmol) and [$Cu(CH_3CN)_4PF_6$] (14.5 mg, 38.9 µmol). The vial was sealed and purged with N_2, then CH_2Cl_2 was added (2.0 mL), followed by iPr2NEt (14 µL, 80 µmol). The solution was stirred at rt for 16 h. Then, MeOH (2.0 mL) and KCN as a solid (13 mg, 0.20 mmol) were added and the resulting mixture was stirred vigorously for 1 h. The crude mixture was diluted with CH_2Cl_2 (5 mL) and washed with H_2O in two portions (10 mL and 5 mL). The combined aqueous phase was then extracted with CH_2Cl_2 (3 x 5 mL) and the combined organic extracts were washed with brine (10 mL), dried ($MgSO_4$) and concentrated *in vacuo* to give a sample containing **4** as a mixture of diastereomers (58 : 42 *dr*, Figure S25). Two rounds of chromatography (1st: *n*-hexane-acetone 0→100%; 2nd: CH_2Cl_2-CH_3CN 0→100%;) gave **4** as a colourless oil (23.5 mg, 53%) as a partially enriched mixture of diastereomers (1.43 : 1 *dr*, Figure S26).

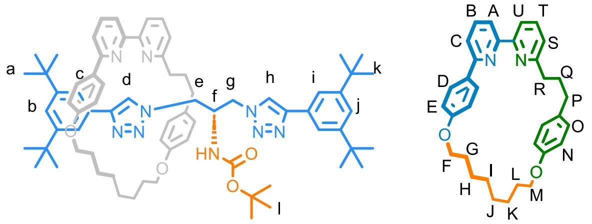

Major diastereomer

^{1}H NMR (500 MHz, CDCl$_3$) δ: 9.71 (s, 1H, H_d), 7.80-7.69 (m, 4H, H_B, H_c, H_T), 7.65-7.51 (m, 5H, H_A, H_C, H_h, H_i), 7.43 (d, *J* = 7.8, 1H, H_U), 7.41-7.38 (m, 1H, H_j), 7.32-7.27 (m, 4H, H_b, H_D, H_S), 6.67-6.56 (m, 2H, H_O), 6.50-6.41 (m, 4H, H_E, H_N), 5.54 (d, *J* = 7.5, 1H, NH), 4.39-4.20 (m, 1H, H_M), 4.14-3.97 (m, 2H, H_F), 3.97-3.58 (m, 5H, H_e, H_f, H_g, $H_{M'}$), 3.52 (dd, *J* = 14.6, 4.1, 1H, $H_{e'}$), 2.85-2.43 (m, 4H, H_P, H_R), 2.09-1.46 (m, 14H, H_G, H_H, H_I, H_J, H_K, H_L, H_Q, superimposed with residual H_2O), 1.38-1.36 (m, 18H, H_k), 1.20 (s, 18H, H_a), 1.17 (bs, 9H, H_l)

Minor diastereomer

^{1}H NMR (500 MHz, CDCl$_3$) δ: 9.63 (s, 1H, H_d), 7.80-7.69 (m, 4H, H_B, H_c, H_T), 7.65-7.51 (m, 4H, H_A, H_C, H_i), 7.49 (d, *J* = 8.0, 1H, H_U), 7.41-7.38 (m, 1H, H_j) , 7.34 (s, 1H, H_h), 7.32-7.27 (m, 2H, H_b, H_S), 7.20 (dt, *J* = 8.6, 1.9, 2H, H_D), 6.67-6.56 (m, 2H, H_O), 6.50-6.41 (m, 2H, H_E), 6.38 (d, *J* = 7.9, 2H, H_N), 5.50 (d, *J* = 8.0, 1H, NH), 4.39-4.20 (m, 1H, H_M), 4.14-3.97 (m, 4H, H_F, H_g), 3.97-3.58 (m, 4H, H_e, H_f, $H_{M'}$), 2.85-2.43 (m, 4H, H_P, H_R), 2.09-1.46 (m, 14H, H_G, H_H, H_I, H_J, H_K, H_L, H_Q, superimposed with residual H_2O), 1.38-1.36 (m, 18H, H_k), 1.23 (bs, 9H, H_l), 1.21 (s, 18H, H_a)

It was not possible to attribute each carbon peak to a single isomer unambiguously, so the complete list of observed peaks is reported below.

^{13}C NMR (126 MHz, CDCl$_3$): 163.4, 163.3, 159.2, 159.2, 158.9, 157.9, 157.7, 157.5, 157.2, 157.2, 157.2, 155.2, 155.0, 151.3, 151.3, 150.8, 150.8, 148.2, 148.1, 147.9, 147.6, 137.4, 137.4, 137.3, 132.2, 132.0, 131.5, 131.2, 131.2, 130.1, 130.1, 129.3, 129.1, 128.8, 124.6, 124.4, 122.8, 122.6, 122.3, 122.2, 121.3, 121.2, 120.8, 120.5, 120.4, 120.4, 120.2, 120.2, 120.1, 115.0, 114.8, 114.1, 114.0, 79.7, 79.4, 68.0, 67.9, 66.8, 66.7, 51.0, 50.6, 50.6, 50.2, 50.1, 42.0, 42.0, 41.9, 41.9, 37.8, 37.7, 37.4, 35.3, 35.1, 34.9, 32.1, 32.1, 31.6, 31.5, 29.8, 29.5, 29.4, 29.3, 28.7, 28.7, 28.6, 28.5, 28.5, 28.4, 28.3, 28.3, 26.1, 26.1, 25.7, 25.6.

HR-ESI-MS (+ve) *m/z* = 1162.7 [M+H]$^+$, for isotopic pattern see Figure S32.

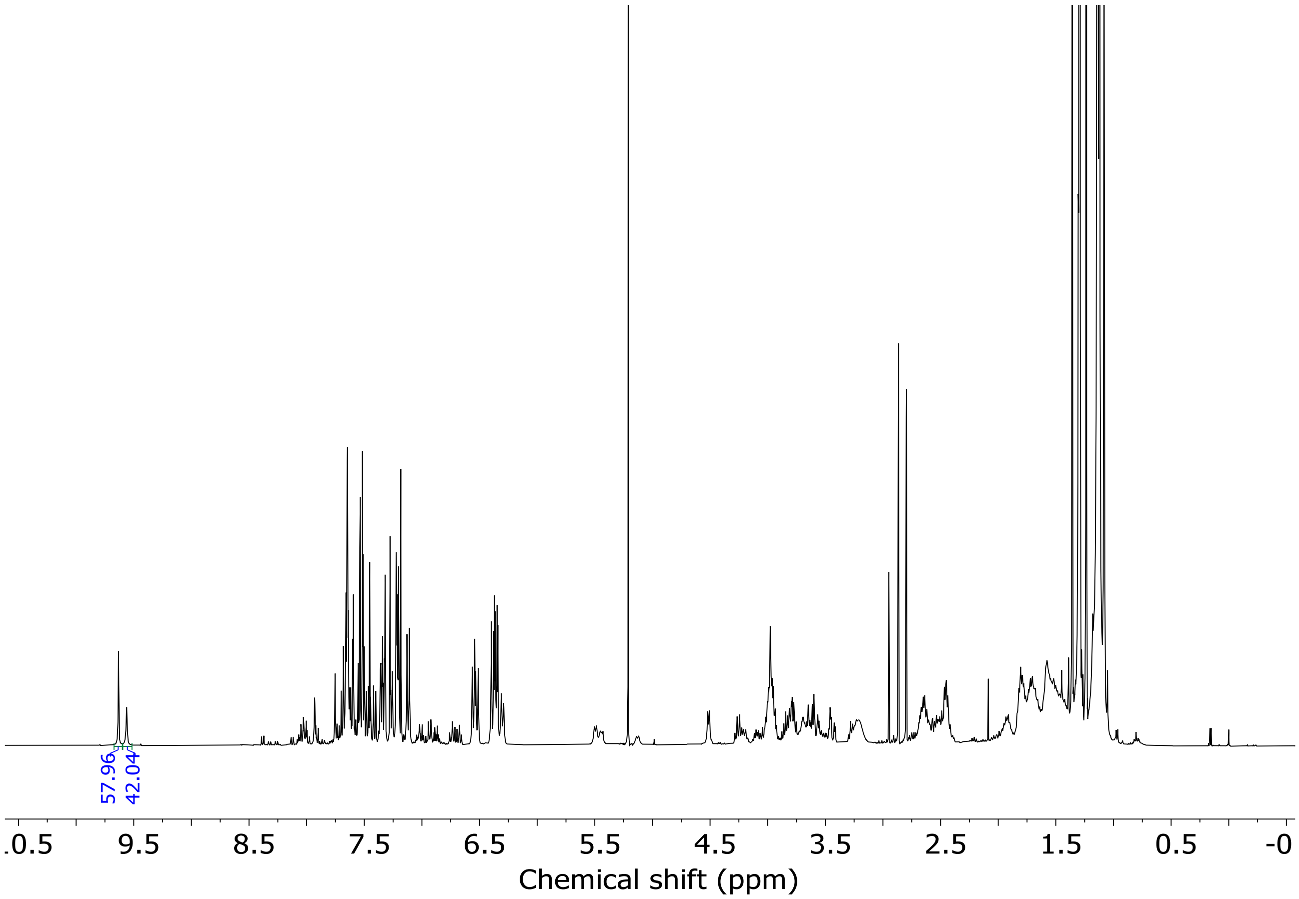

Figure S25: ^{1}H NMR (CDCl$_3$, 400 MHz) of rotaxanes **4** prior to chromatography (58 : 42 *dr*).

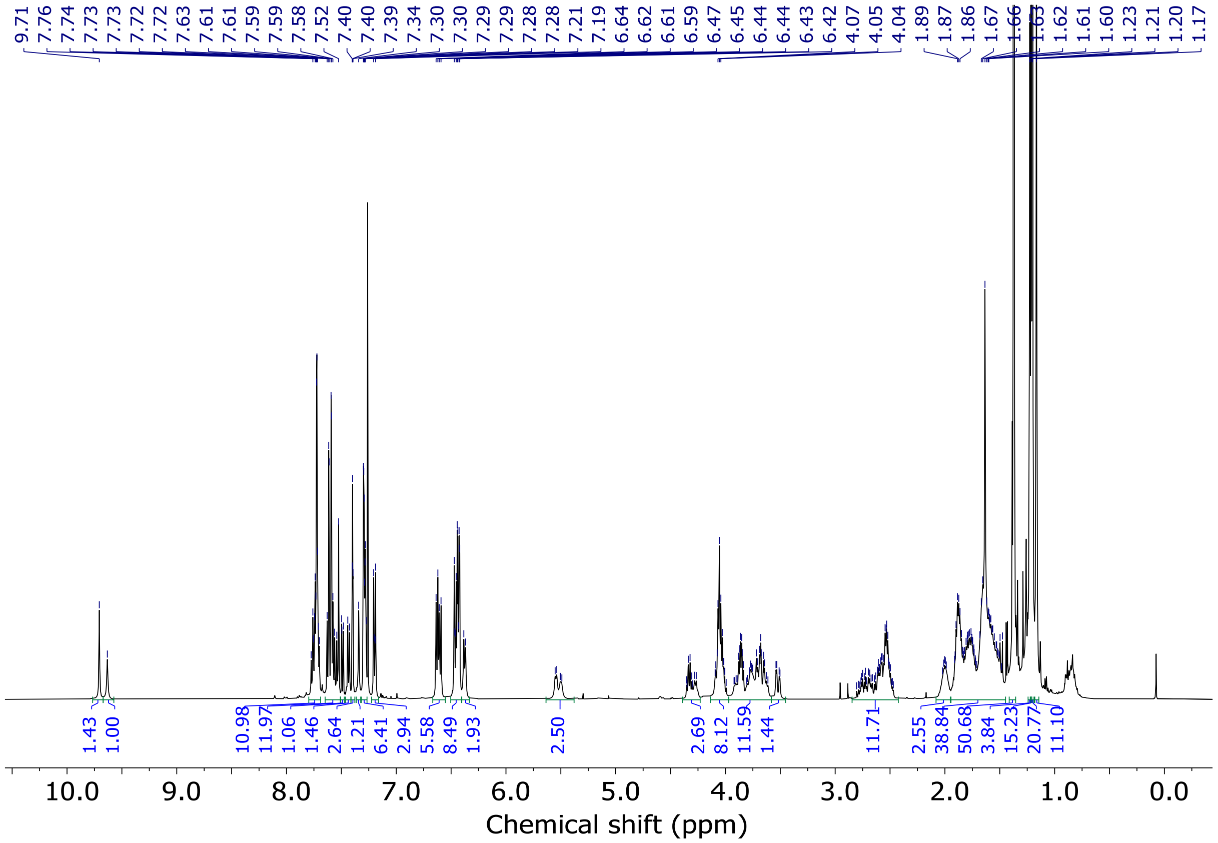

Figure S26: ^{1}H NMR ($CDCl_3$, 500 MHz) of (Z_m,$S_{co\text{-}c}$)-**4** and (E_m,$S_{co\text{-}c}$)-**4** (1.4 : 1 *dr*).

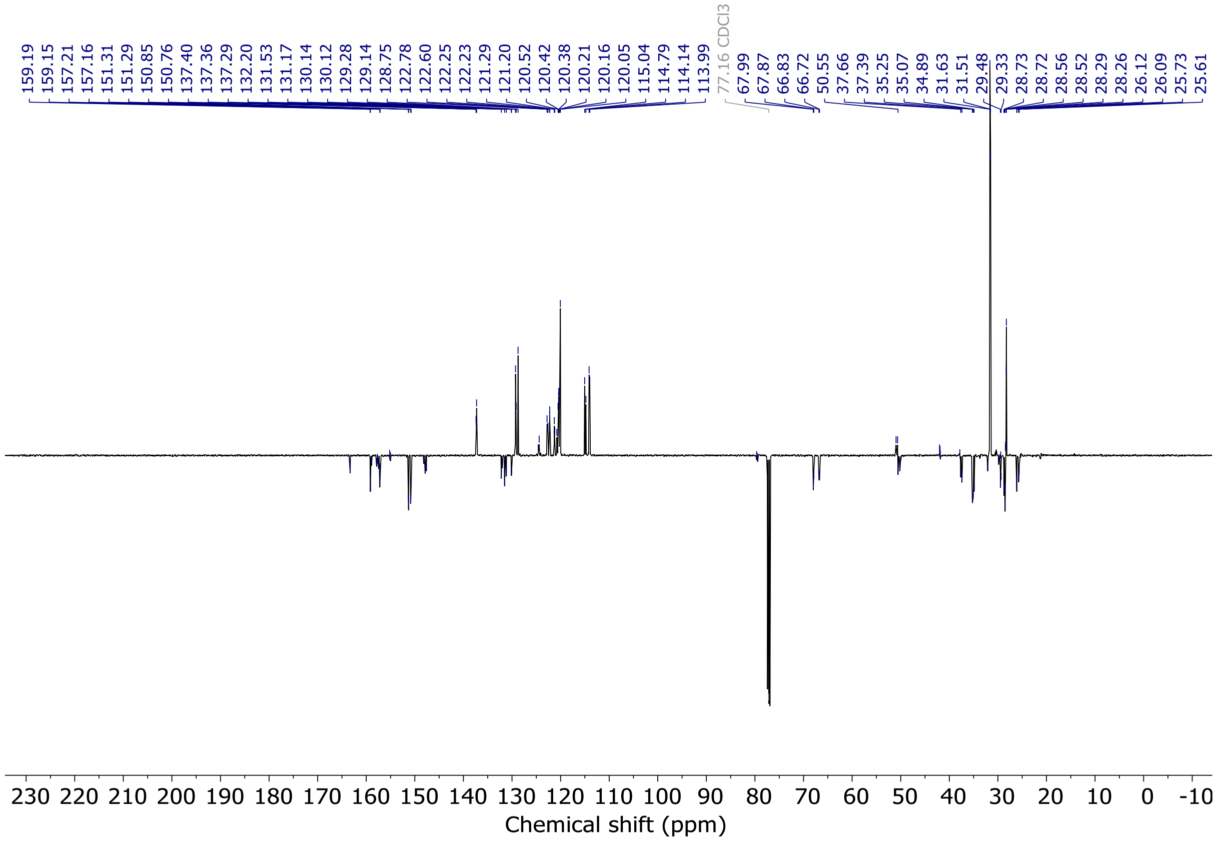

Figure S27: JMOD NMR ($CDCl_3$, 126 MHz) of (Z_m,$S_{co\text{-}c}$)-**4** and (E_m,$S_{co\text{-}c}$)-**4** (1.4 : 1 *dr*).

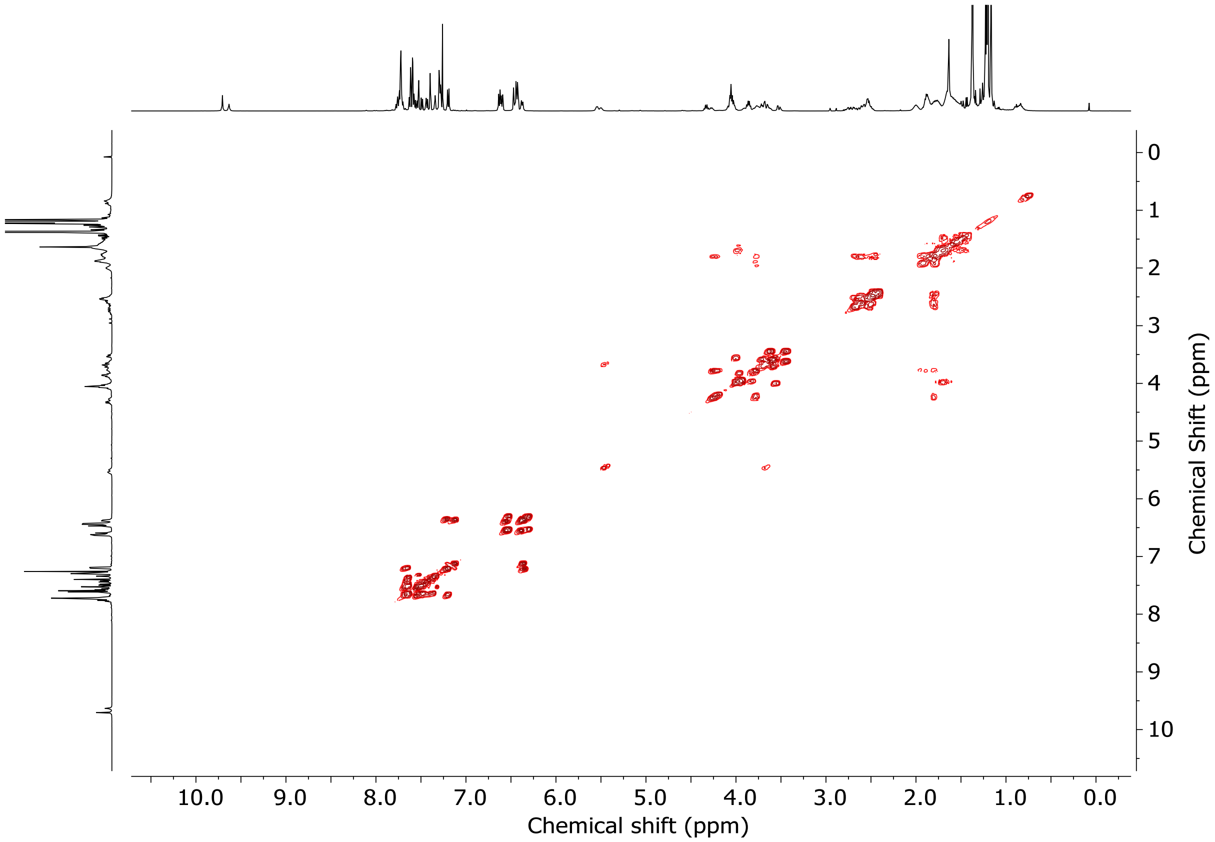

Figure S28: COSY NMR ($CDCl_3$) of (Z_m,S_{co-c})-**4** and (E_m,S_{co-c})-**4** (1.4 : 1 *dr*).

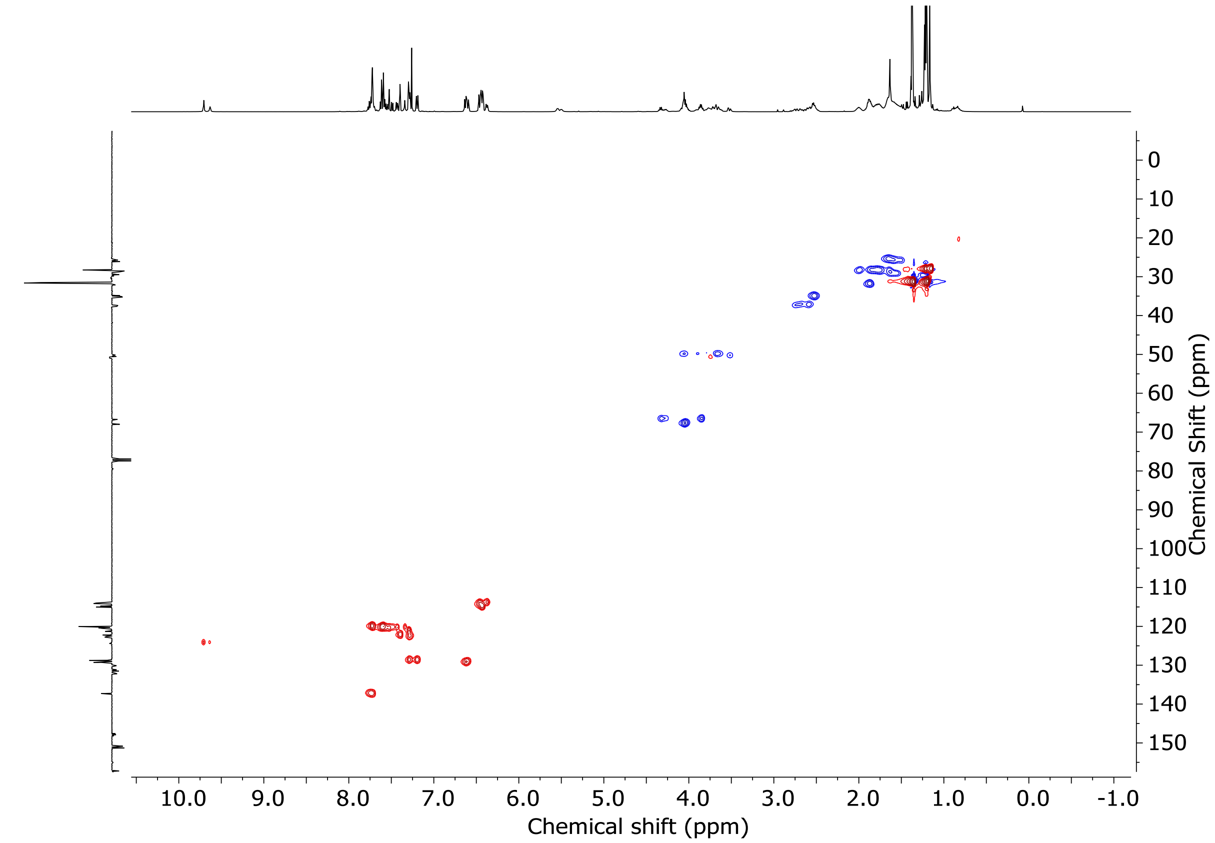

Figure S29: HSQC NMR ($CDCl_3$) of (Z_m,S_{co-c})-**4** and (E_m,S_{co-c})-**4** (1.4 : 1 *dr*).

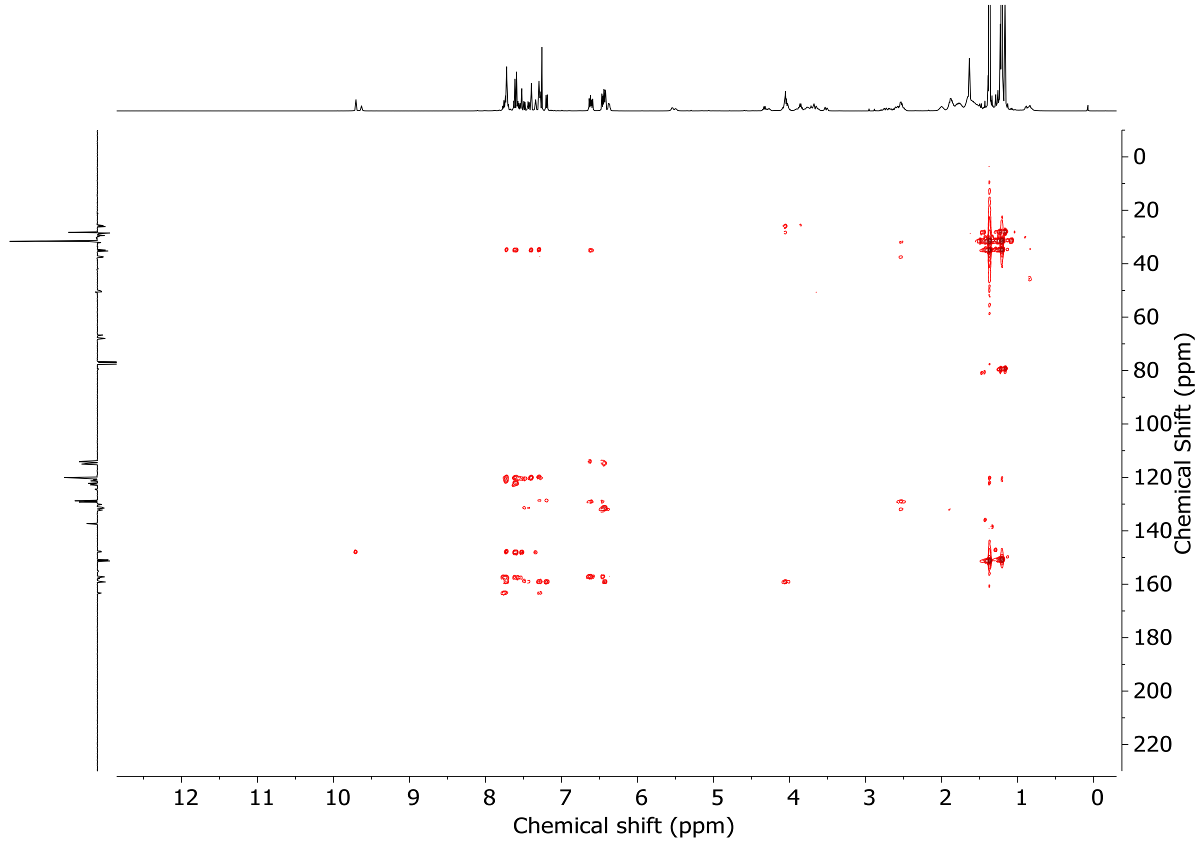

Figure S30: HMBC NMR ($CDCl_3$) of (Z_m,$S_{co\text{-}c}$)-**4** and (E_m,$S_{co\text{-}c}$)-**4** (1.4 : 1 *dr*).

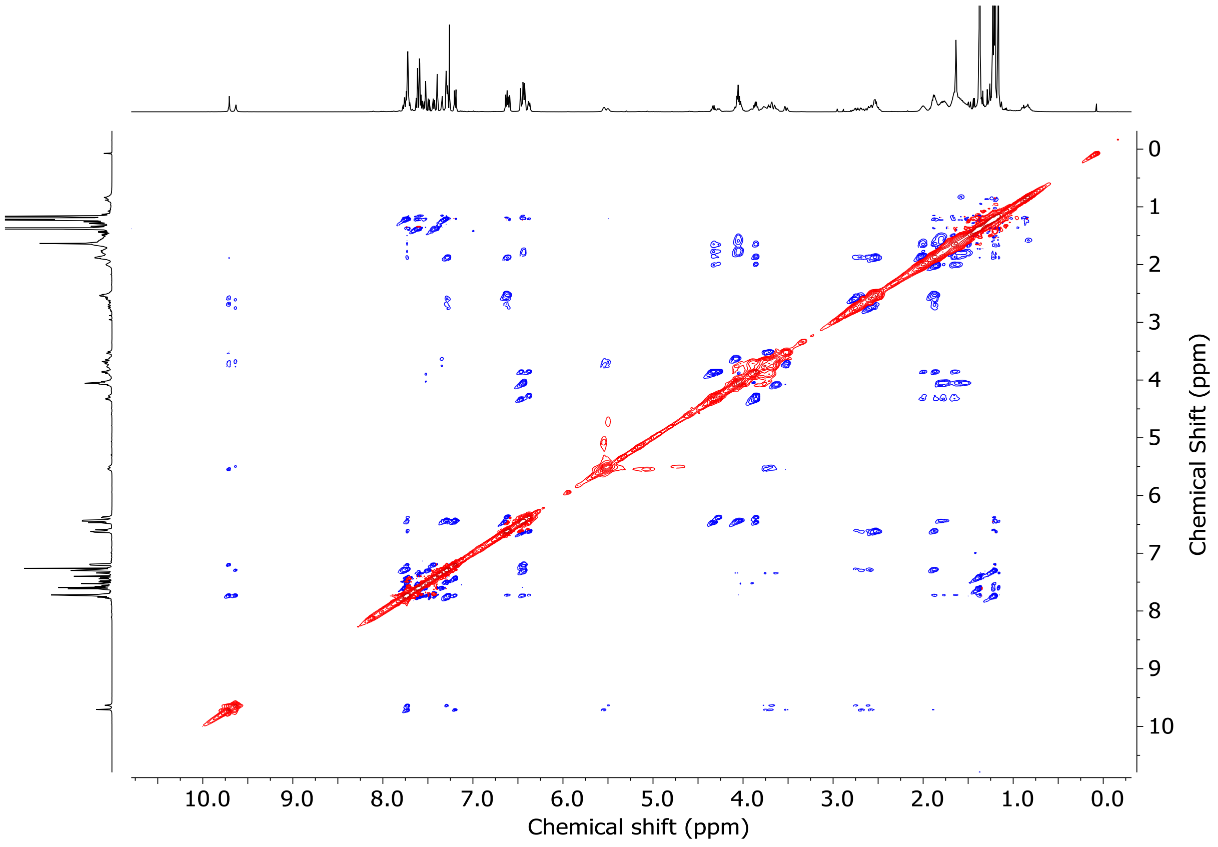

Figure S31: NOESY NMR ($CDCl_3$) of (Z_m,$S_{co\text{-}c}$)-**4** and (E_m,$S_{co\text{-}c}$)-**4** (1.4 : 1 *dr*).

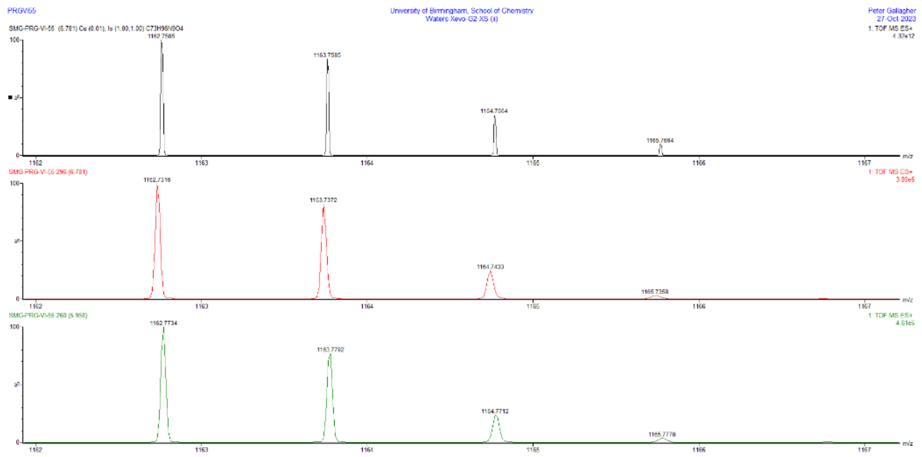

Figure S32: Calculated (top) and observed (middle, bottom) isotopic patterns for (Z_m,$S_{co\text{-}c}$)-**4** and (E_m,$S_{co\text{-}c}$)-**4**.

1.6 Amine Rotaxanes 5

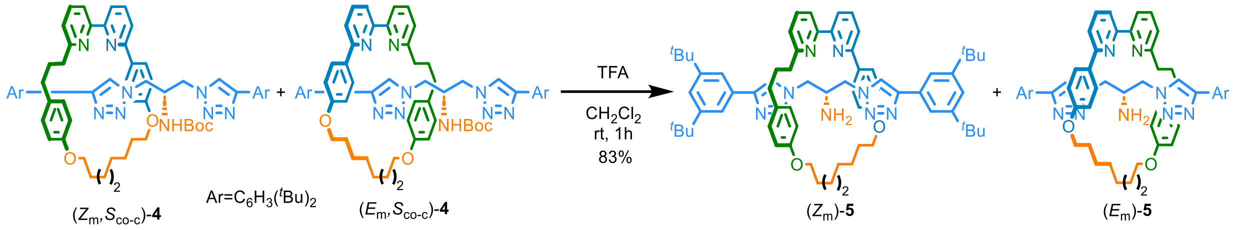

A vessel was charged with a mixture of rotaxanes **4** (16.4 mg, 0.014 mmol, 55:45 *dr*), TFA (25.0 µL, 0.21 mmol), and CH_2Cl_2 (1 mL). The reaction mixture was stirred at rt for 1 h. CH_2Cl_2 (10 mL) was added, washed with sat. $NaHCO_3$ solution (10 mL), brine (10 mL), and the combined organic extracts were dried ($MgSO_4$) and concentrated *in vacuo* to give rotaxanes **5** as a white foam (12.3 mg, 83%, 57:43 *dr*) that were characterized without further purification.

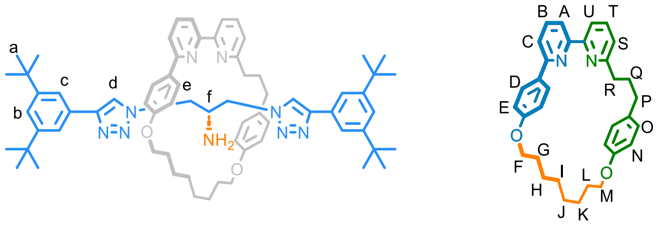

Major diastereomer

^{1}H NMR (500 MHz, CDCl$_3$) δ: 8.54 (s, 2H, H_d), 7.73 (app. T, J = 8.2, 1H, H_T), 7.70-7.63 (m, 5H, H_c, H_B), 7.60-7.55 (m, 1H, H_U), 7.52-7.48 (m, 1H, H_A or H_C), 7.45-7.39 (m, 1H, H_A or H_C), 7.37-7.34 (m, 2H, H_b), 7.30-7.19 (m, 3H, H_S and H_D or H_E, superimposed with residual $CHCl_3$), 6.71-6.63 (m, 2H, H_O), 6.58 (dt, J = 8.4, 2.5, 2H, H_N), 6.51-6.46 (m, 2H, H_D or H_E), 4.22-3.99 (m, 4H, H_F, H_M), 3.88-3.70 (m, 2H, H_e), 3.29 (dd, J = 14.0, 7.6, 2H, $H_{e'}$), 2.65-2.40 (m, 5H, H_f, H_P, H_R), 1.97-1.44 (m, 14H, H_G, H_H, H_I, H_J, H_K, H_L, H_Q, superimposed with residual H_2O), 1.29 (s, 36H, H_a).

Minor diastereomer

^{1}H NMR (500 MHz, CDCl$_3$) δ: 8.54 (s, 2H, H_d), 7.73 (app. T, J = 8.2, 1H, H_T), 7.70-7.63 (m, 5H, H_c, H_B), 7.60-7.55 (m, 1H, H_U), 7.52-7.48 (m, 1H, H_A or H_C), 7.45-7.39 (m, 1H, H_A or H_C), 7.37-7.34 (m, 2H, H_b), 7.30-7.19 (m, 3H, H_S and H_D or H_E, superimposed with residual $CHCl_3$), 6.71-6.63 (m, 2H, H_O), 6.58 (dt, J = 8.4, 2.5, 2H, H_N), 6.51-6.46 (m, 2H,

H_D or H_E), 4.22-3.99 (m, 4H, H_F, H_M), 3.88-3.70 (m, 2H, H_e), 3.29 (dd, *J* = 14.0, 7.6, 2H, $H_{e'}$), 2.65-2.40 (m, 5H, H_f, H_P, H_R), 1.97-1.44 (m, 14H, H_G, H_H, H_I, H_J, H_K, H_L, H_Q, superimposed with residual H_2O), 1.29 (s, 36H, H_a).

It was not possible to attribute each carbon peak to a single isomer unambiguously, so the complete list of observed peaks is reported below.

^{13}C NMR (126 MHz, $CDCl_3$) δ: 163.1, 159.1, 159.1, 159.0, 157.5, 157.1, 151.1, 151.0, 147.9, 147.9, 137.4, 137.3, 137.3, 137.2, 132.5, 132.3, 131.9, 131.8, 130.4, 129.4, 129.3, 129.0, 128.8, 122.7, 122.6, 122.2, 122.0, 121.8, 121.8, 120.6, 120.5, 120.5, 120.3, 120.2, 120.0, 119.9, 115.0, 115.0, 114.2, 114.1, 67.7, 67.5, 66.7, 66.5, 50.5, 50.4, 37.4, 37.3, 35.1, 35.1, 34.9, 32.7, 32.4, 31.4, 29.7, 29.7, 29.4, 29.4, 29.4, 28.8, 28.6, 28.6, 28.4, 28.4, 26.0, 25.7, 25.6

HR-ESI-MS (+ve) *m/z* = 1062.7061 $[M+H]^+$, for isotopic pattern see Figure S32.

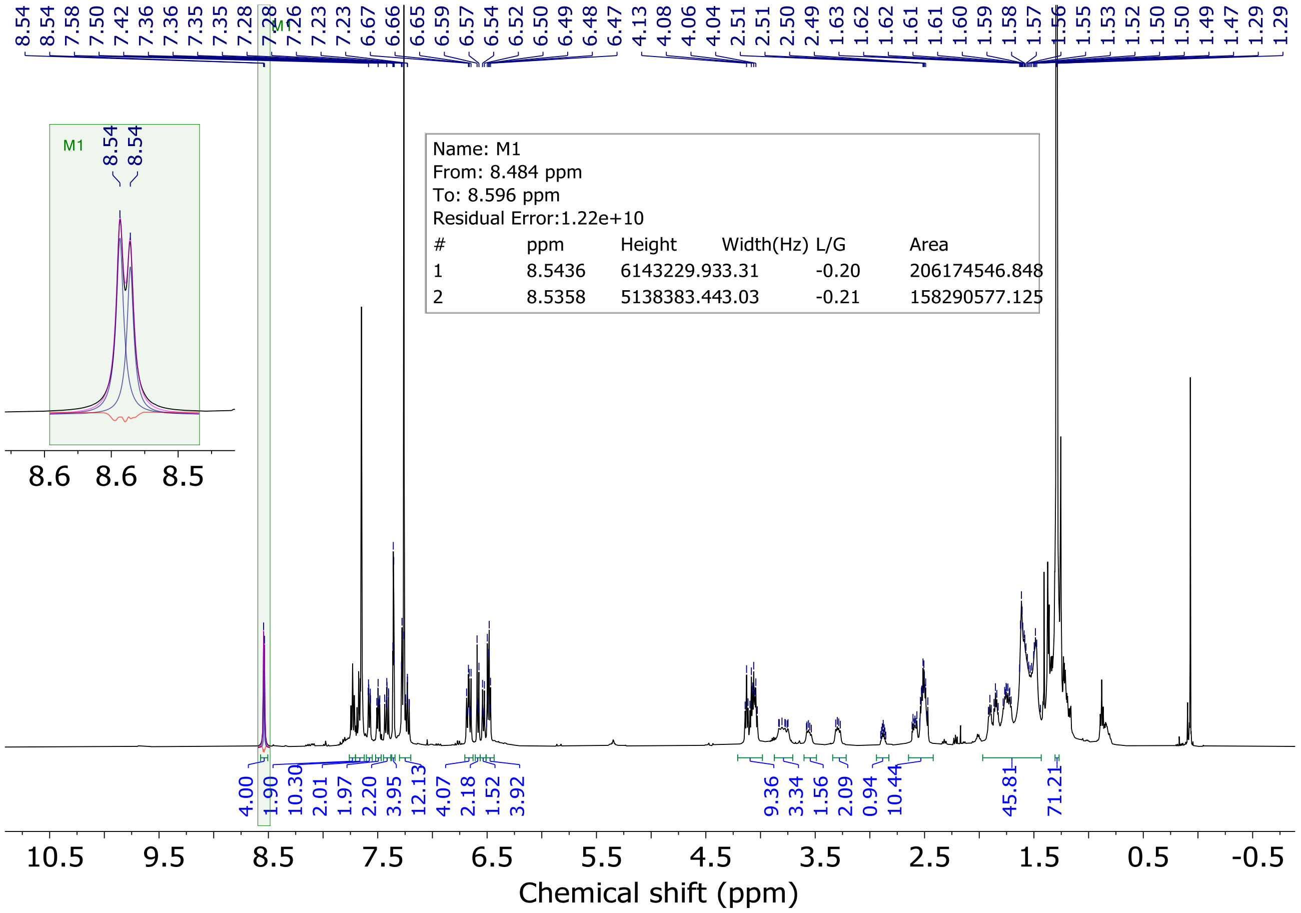

Figure S33: ^{1}H NMR ($CDCl_3$, 500 MHz) of (Z_m)-**5** and (E_m)-**5** (1.3 : 1 *dr*).

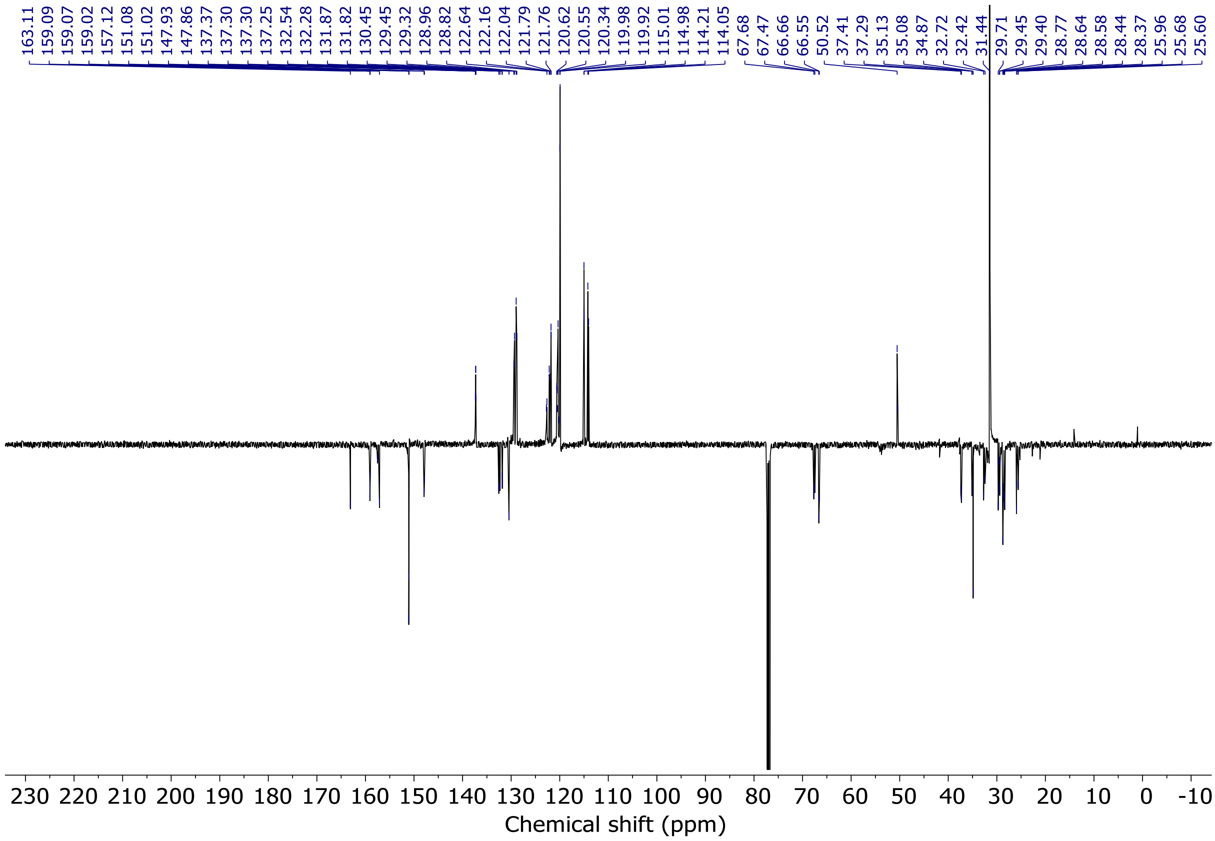

Figure S34: JMOD NMR ($CDCl_3$, 126 MHz) of (Z_m)-**5** and (E_m)-**5** (1.3 : 1 *dr*).

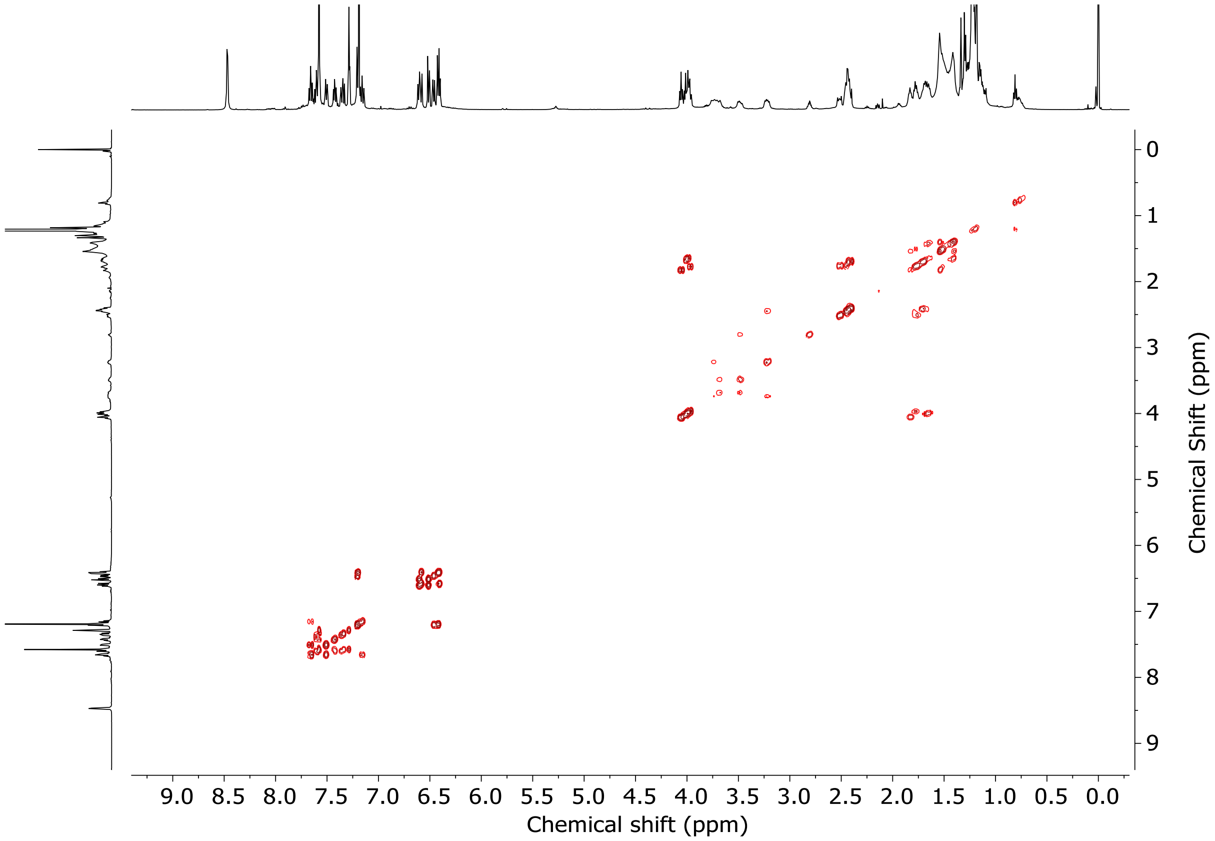

Figure S35: COSY NMR ($CDCl_3$) of (Z_m)-**5** and (E_m)-**5** (1.3 : 1 *dr*).

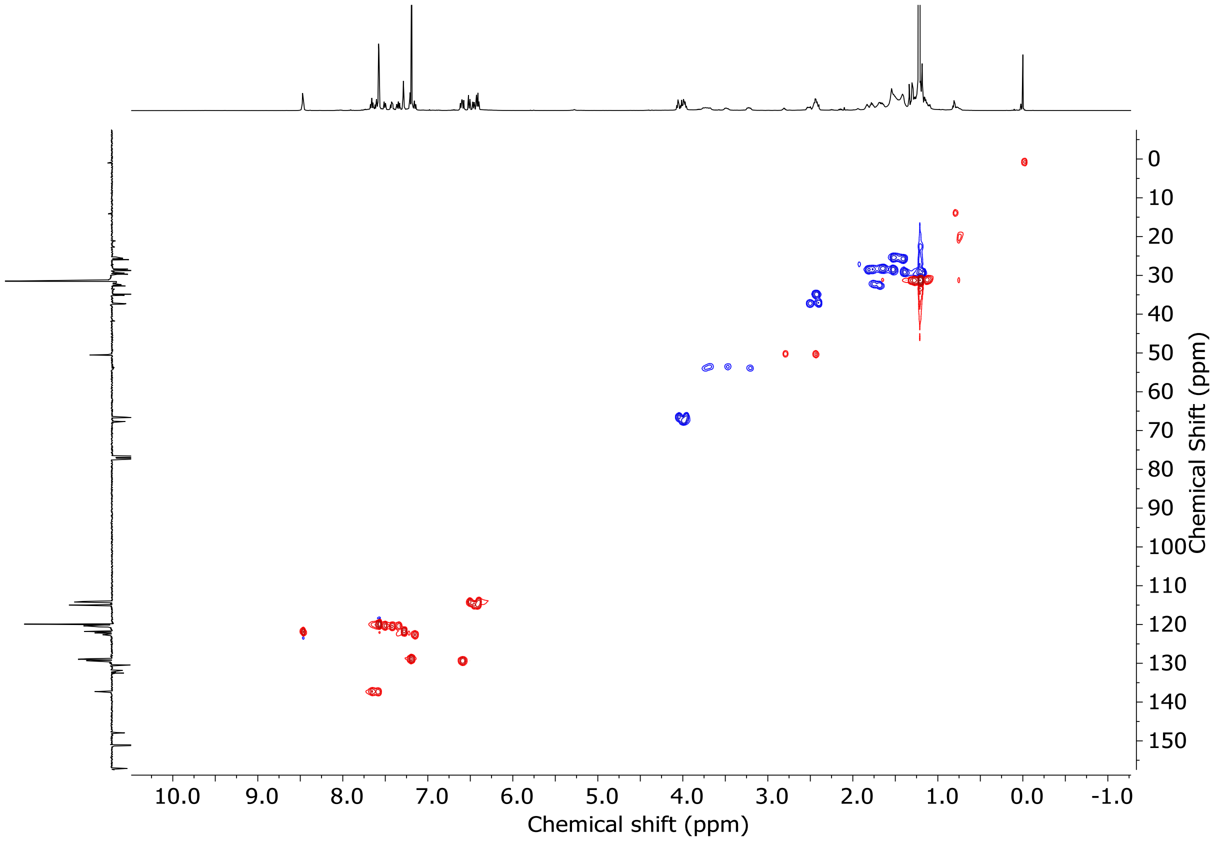

Figure S36: HSQC NMR ($CDCl_3$) of (Z_m)-**5** and (E_m)-**5** (1.3 : 1 *dr*).

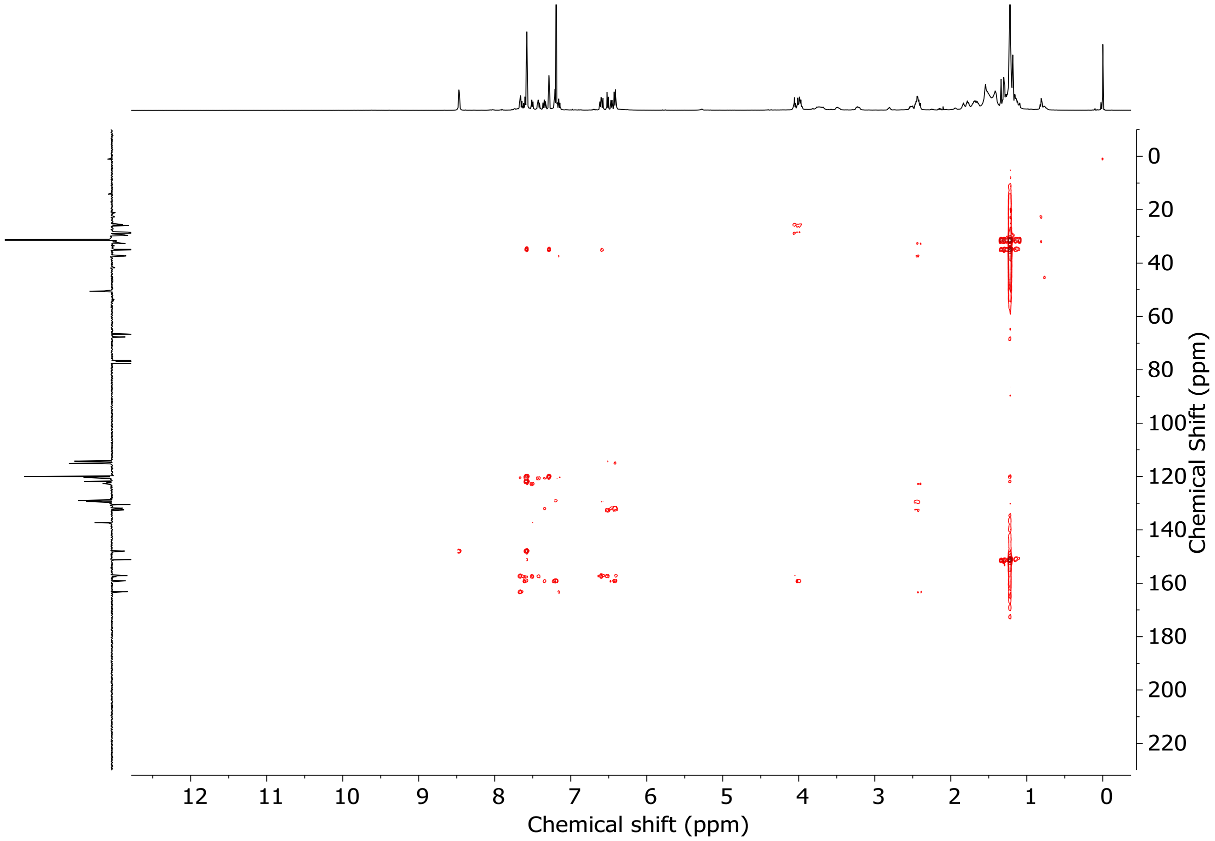

Figure S37: HMBC NMR ($CDCl_3$) of (Z_m)-**5** and (E_m)-**5** (1.3 : 1 *dr*).

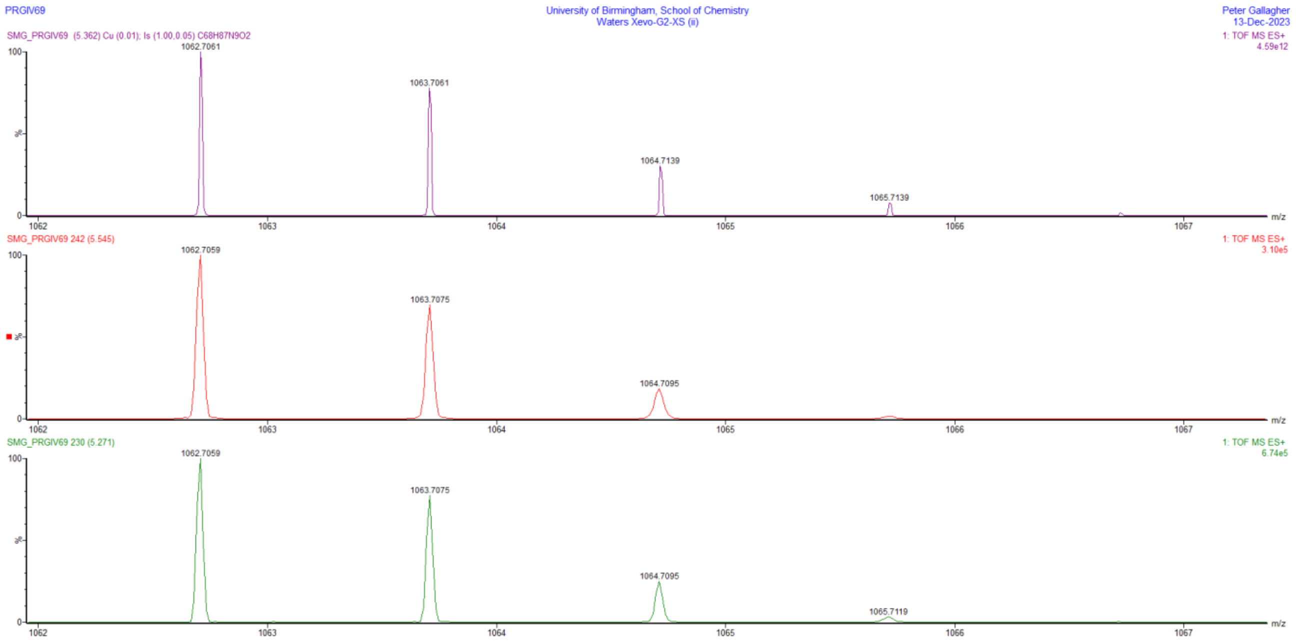

Figure S38: Calculated (top) and observed (middle, bottom) isotopic patterns for (Z_m)-**5** and (E_m)-**5**.

5. Synthesis of rotaxane 11 and associated compounds (Scheme 2)

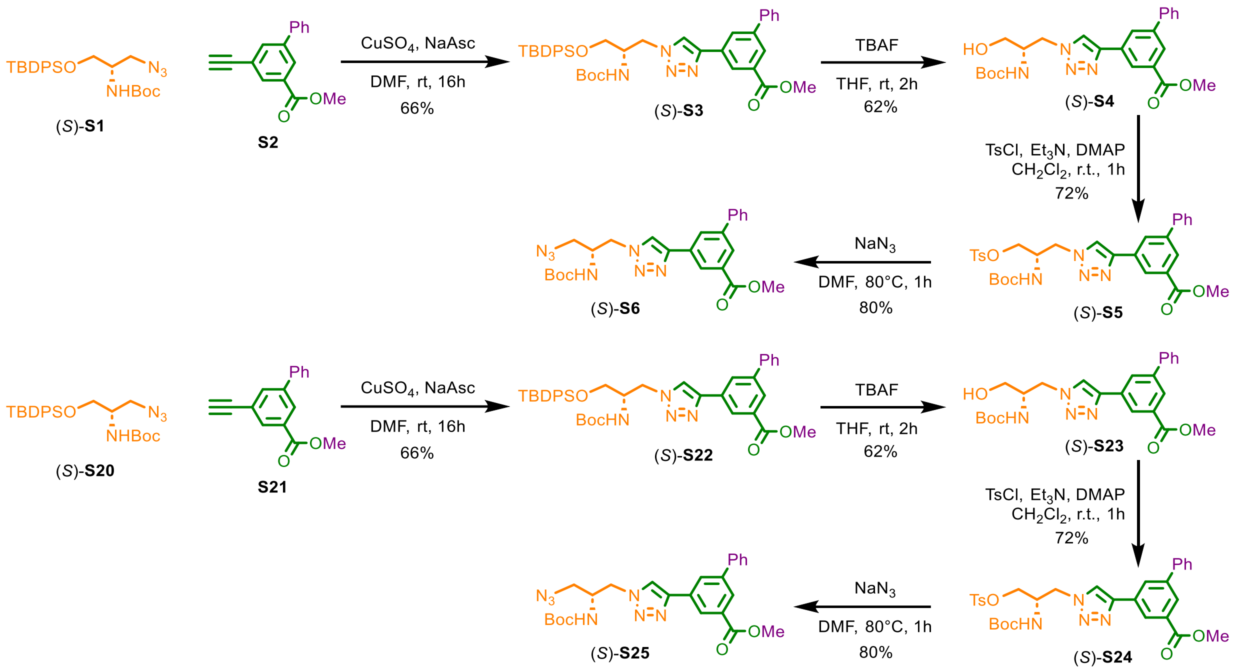

Scheme S1: Synthetic route to azide (*S*)-**S6.**

Silyl ether (*S*)-S3

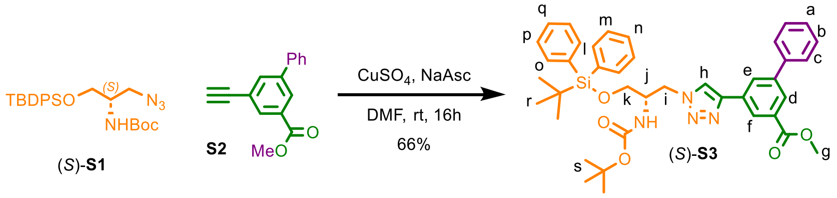

A suspension of (*S*)-**S1** (1.20g, 2.64 mmol), **S2** (623 mg, 2.64 mmol), $CuSO_4$ (42.2 mg, 0.26 mmol) and sodium ascorbate (78.4 mg, 0.39 mmol) in DMF (30 mL) is degassed by bubbling N_2 for 10 min. The solution turns brown, and it is stirred at rt for 16h. The reaction mixture is poured into a NH_3-EDTA (100 mL) then extracted with Et_2O (3 x 50 mL). The organic phases are washed with a 5% LiCl solution (50 mL), washed with brine (50 mL), dried ($MgSO_4$) and concentrated *in vacuo*. Chromatography (petrol-EtOAc 0→20%) gave (*S*)-**S3** as a white foam (1.2 g, 66%).

^{1}H NMR (400 MHz, $CDCl_3$) δ: 8.35 (t, *J* = 1.4, 1H, H_f), 8.31 (t, *J* = 1.8, 1H, H_e) 8.26 (t, *J* = 1.7, 1H, H_d) 7.84 (bs, 1H, H_h), 7.71-7.66 (m, 2H, H_b), 7.66-7.59 (m, 4H, H_p, H_m *or* H_o, H_l) 7.51-7.45 (m, 2H, H_c) 7.44-7.35 (m, 7H, H_a, H_n, H_o and H_l, H_o *or* H_p, H_m), 5.01 (d, *J* = 8.42, 1H, NH), 4.64 (d, *J* = 6.2, 2H, H_k, $H_{k'}$) 4.19 (app bs, 1H, H_j), 3.97 (s, 3H, H_g), 3.79-3.69 (m, 1H, H_i or $H_{i'}$), 3.67-3.53 (m, 1H, H_i or $H_{i'}$), 1.40 (bs, 9H, H_s), 1.11 (s, 9H, H_r)

^{13}C NMR (101 MHz, $CDCl_3$) δ: 166.8, 146.8, 142.2, 139.8, 139.8, 135.6, 135.6, 135.5, 132.7, 132.5, 131.4, 131.3, 130.1, 130.1, 128.9, 128.6, 128.0, 128.0, 127.9, 127.2, 125.5, 121.2, 79.7 (HMBC), 62.9, 52.3, 51.9, 50.7, 28.3, 27.0, 23.9, 19.3.

HR-ESI-MS (+ve) *m/z* = 691.3317 $[M+H]^+$ (calc. 691.3310 *m/z* for $C_{40}H_{46}N_4O_5Si$);

$[\alpha]_D^{23}$+1.2 (c 0.65, $CHCl_3$);

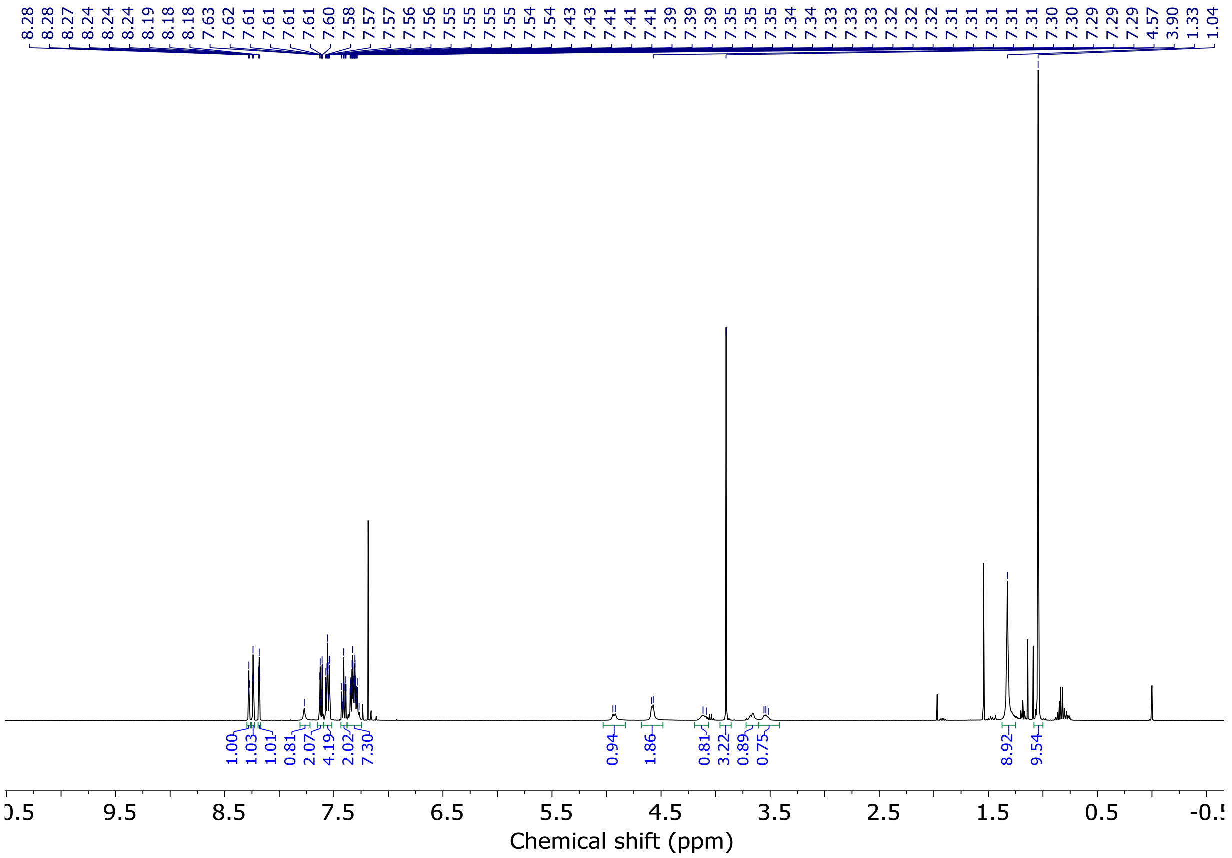

Figure S39: ^{1}H NMR ($CDCl_3$, 400 MHz) of (*S*)-**S3**.

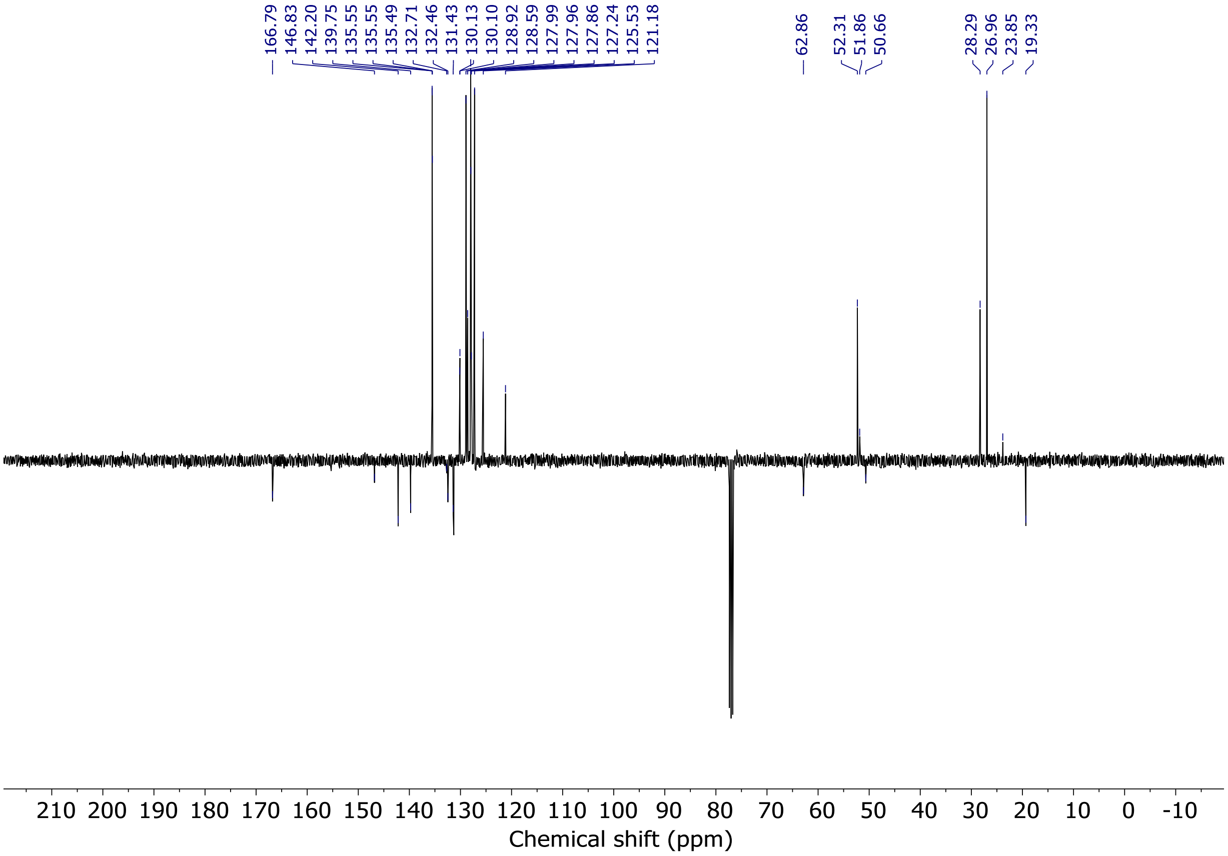

Figure S40: JMOD NMR ($CDCl_3$, 101 MHz) of (*S*)-**S3**.

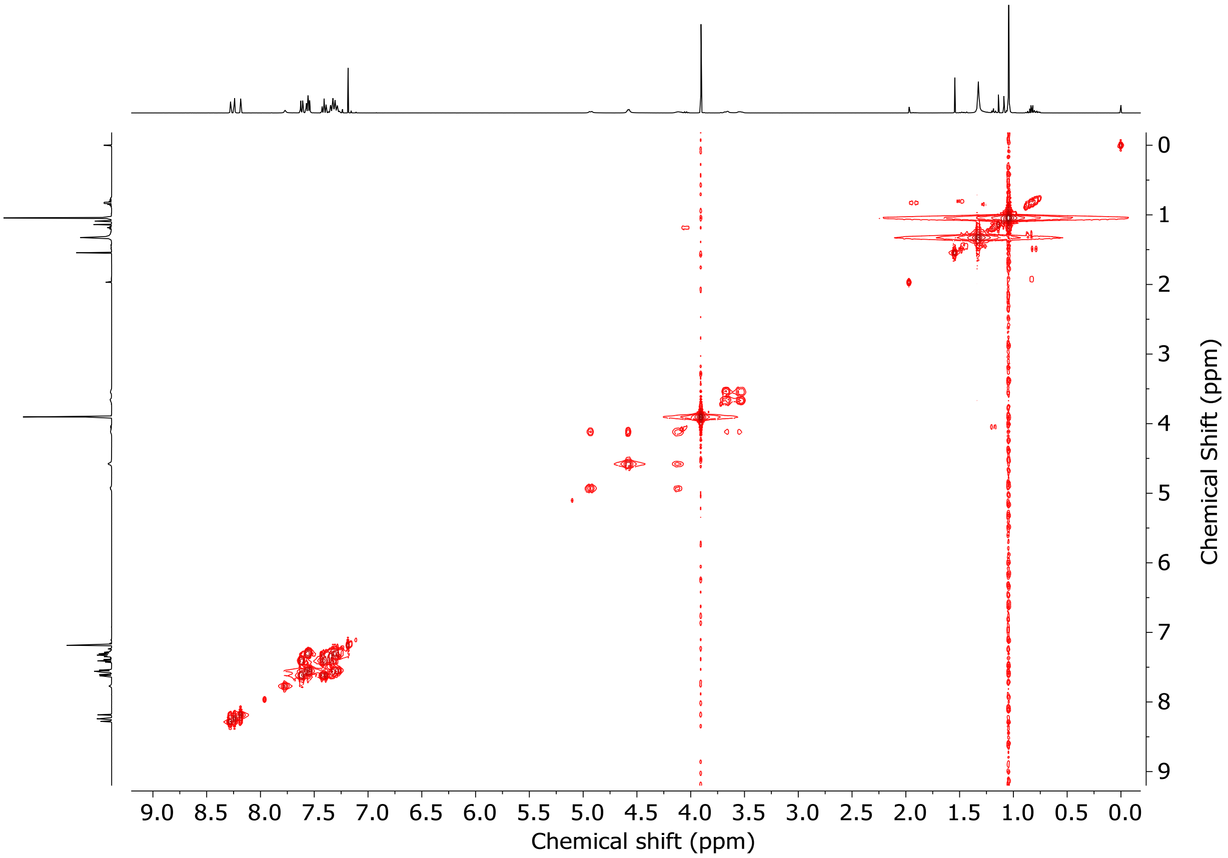

Figure S41: COSY NMR ($CDCl_3$) of (*S*)-**S3**.

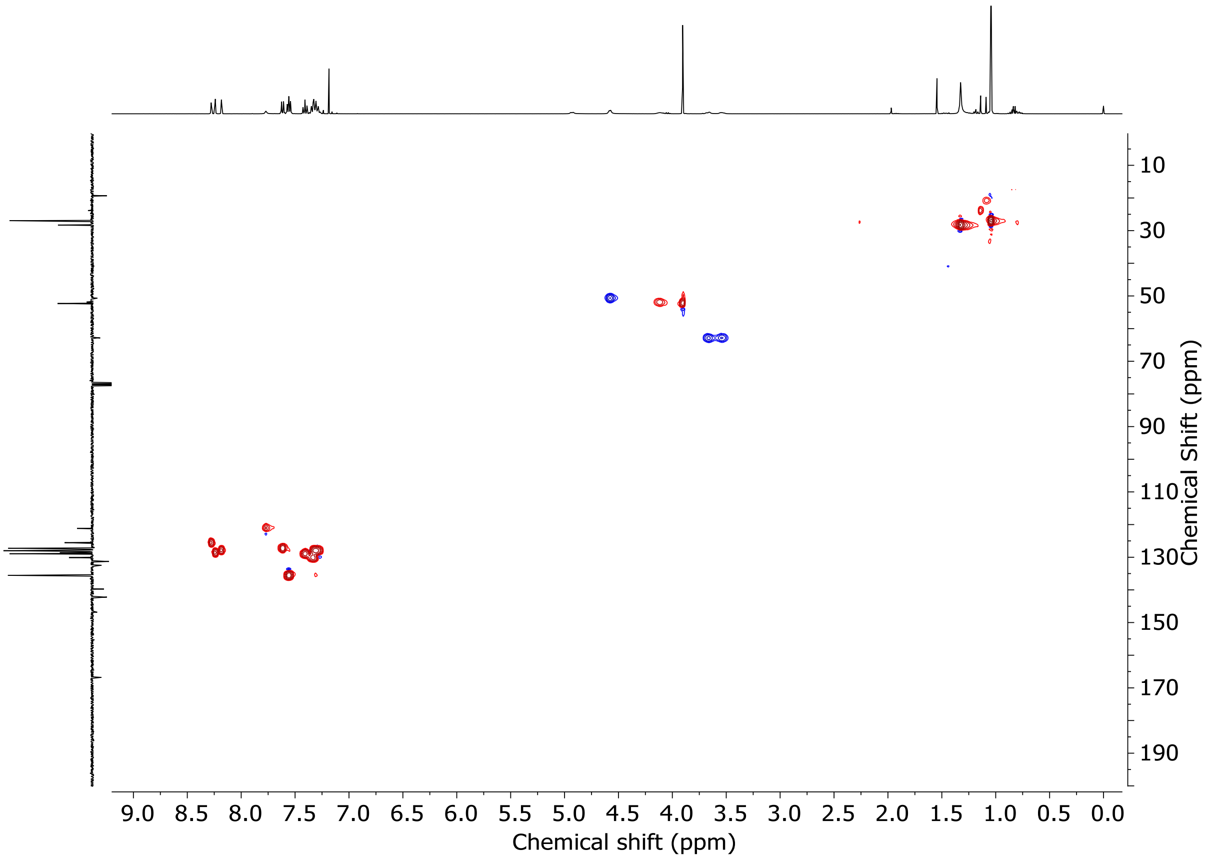

Figure S42: HSQC NMR ($CDCl_3$) of (*S*)-**S3**.

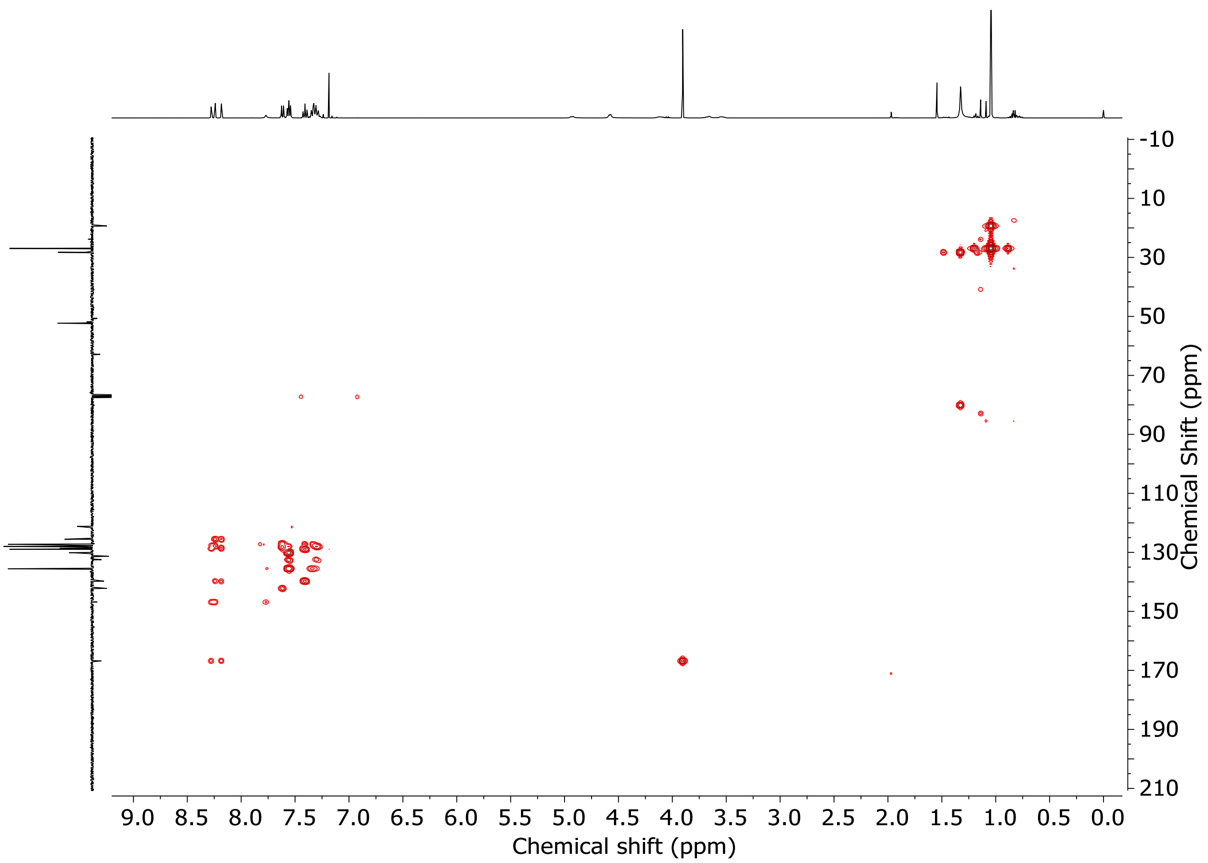

Figure S43: HMBC NMR ($CDCl_3$) of (*S*)-**S3**.

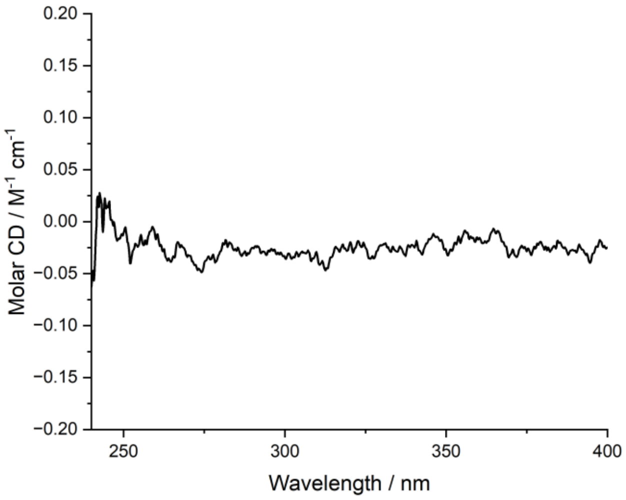

Figure S44: Circular dichroism spectra of (*S*)-**S3** (63.5 µM) at 293 K in $CHCl_3$. No measurable CD response was observed so the $[\boldsymbol{a}]_{\boldsymbol{D}}$ of (*S*)-**S3** was measured.

Alcohol (*S*)-S4

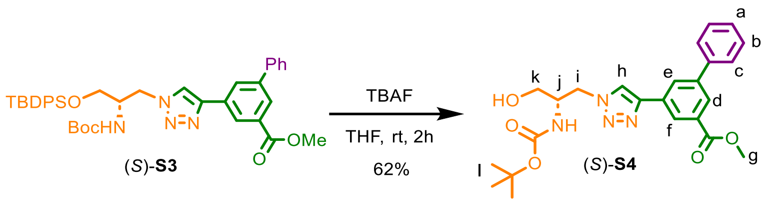

To a suspension of (*S*)-**S3** (1.20 g, 1.74 mmol) in THF (20 mL) was added a 1M solution of TBAF in THF (3.5 mL). The solution is stirred at rt for 2 h. The reaction mixture was partitioned between H_2O (20 mL) and EtOAc (50 mL), the phases separated, and the organic layer extracted with EtOAc (2 x 50 mL). The collected organic fractions were then washed with brine (50 mL), then dried ($MgSO_4$). The solvent was removed *in vacuo* and the crude was purified by column chromatography (CH_2Cl_2-CH_3CN 0→80%) to give (*S*)-**S4** as a white foam (486 mg, 62%).

^{1}H NMR (400 MHz, $CDCl_3$) δ: 8.38 (t, *J* = 1.60, 1H, H_f), 8.35 (t, *J* = 1.60, 1H, H_e), 8.26 (t, *J* = 1.60, 1H, H_d), 7.99 (s, 1H, H_h), 7.71-7.66 (m, 2H, H_c) 7.51-7.45 (m, 2H, H_b) 7.40 (app. tt, *J* = 7.4, 1.1, 1H, H_a) 5.18 (d, *J* = 7.6, 1H, NH), 4.77-4.62 (m, 2H, H_k, $H_{k'}$) 4.09 (app. sept., 1H, H_j), 3.97 (s, 3H, H_g), 3.77 (dd, *J* = 15.0, 4.8, 1H, H_i or $H_{i'}$), 3.62 (dd, *J* = 11.6, 4.8, 1H, H_i or $H_{i'}$), 1.44 (s, 9H, H_l)

^{13}C NMR (101 MHz, $CDCl_3$) δ: 166.8, 147.0 (HMBC) 142.3, 139.7, 131.4, 131.2, 128.9, 128.6, 128.0, 128.0, 127.2, 125.5, 121.6, 80.4 (HMBC), 61.8, 52.3, 52.0, 50.0 28.3.

HR-ESI-MS (+ve) *m/z* = 453.2133 $[M+H]^+$ (calc. 453.2132 *m/z* for $C_{24}H_{29}N_4O_5$);

$[\alpha]_D^{23}$ -0.8 (c 0.74, $CHCl_3$)

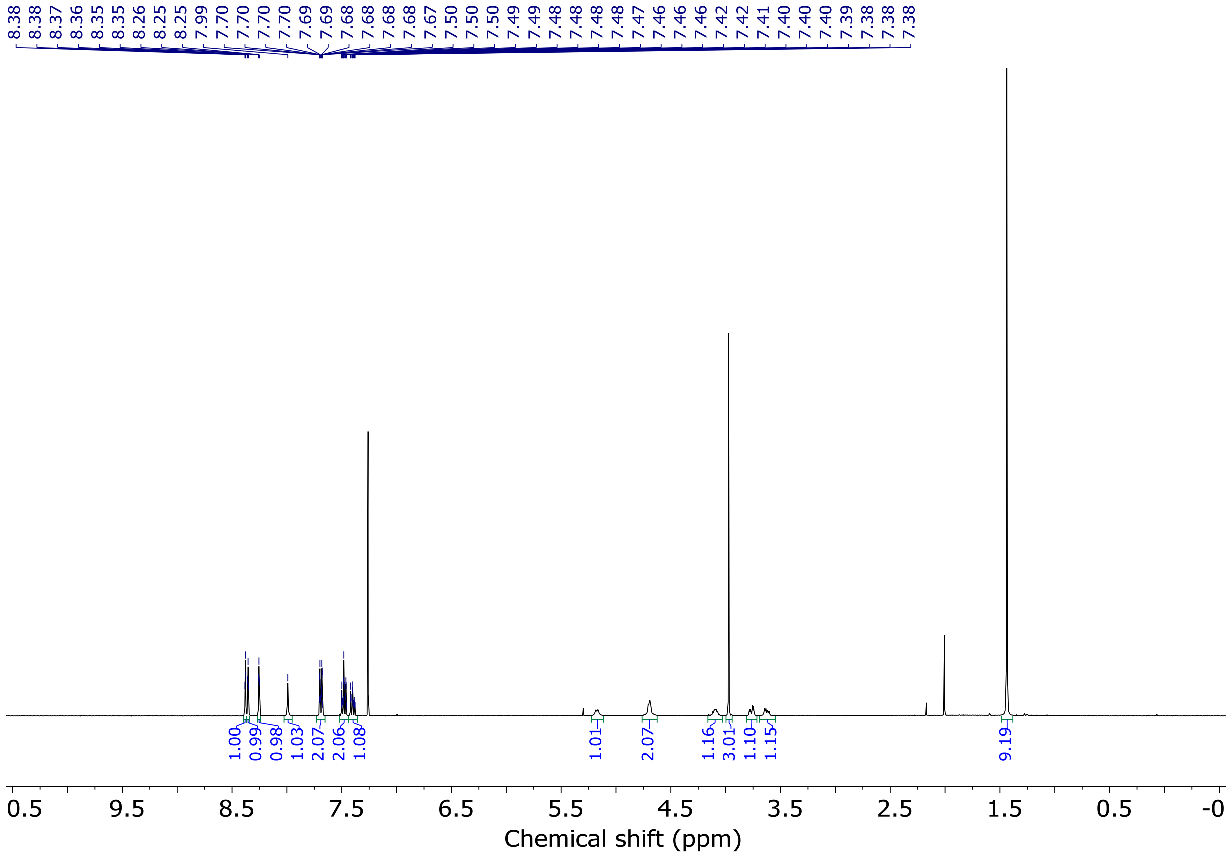

Figure S45: ^{1}H NMR ($CDCl_3$, 400 MHz) of (*S*)-**S4**.

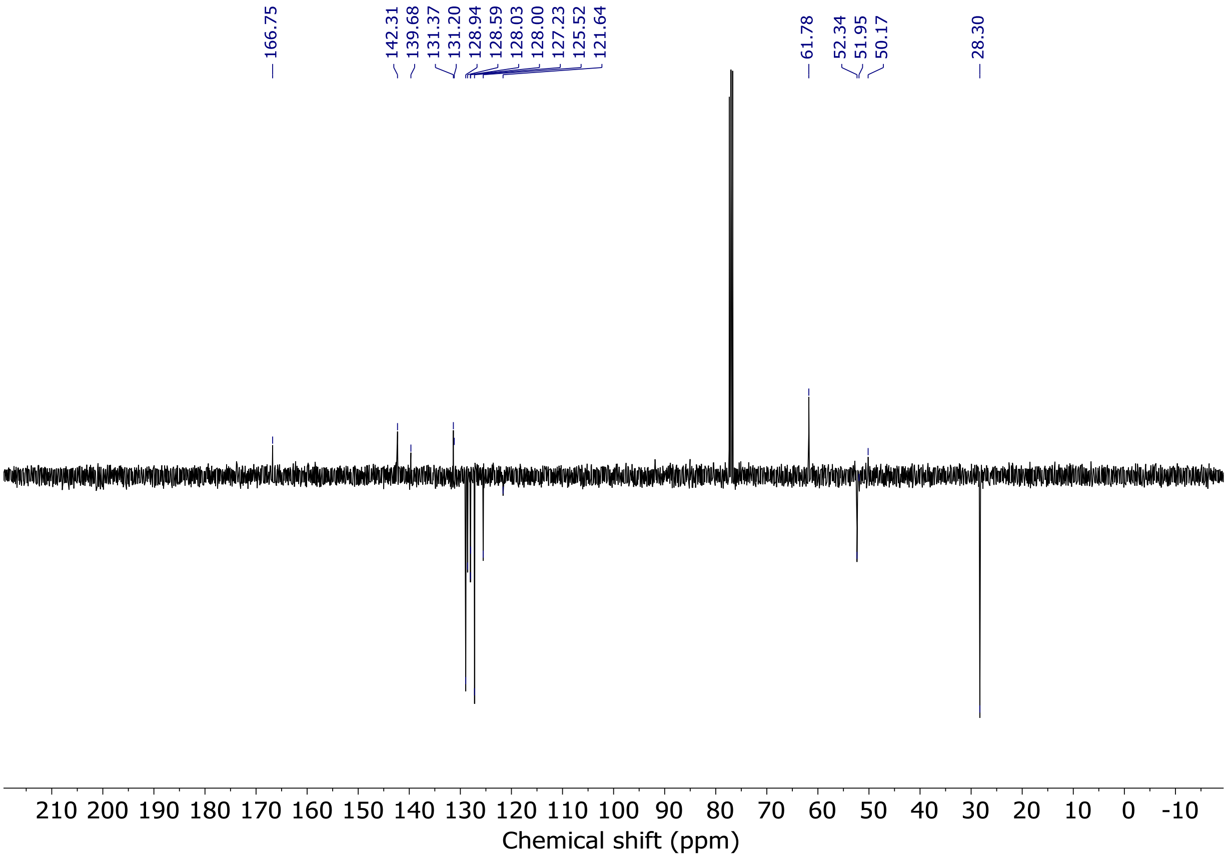

Figure S46: JMOD NMR ($CDCl_3$, 101 MHz) of (*S*)-**S4**.

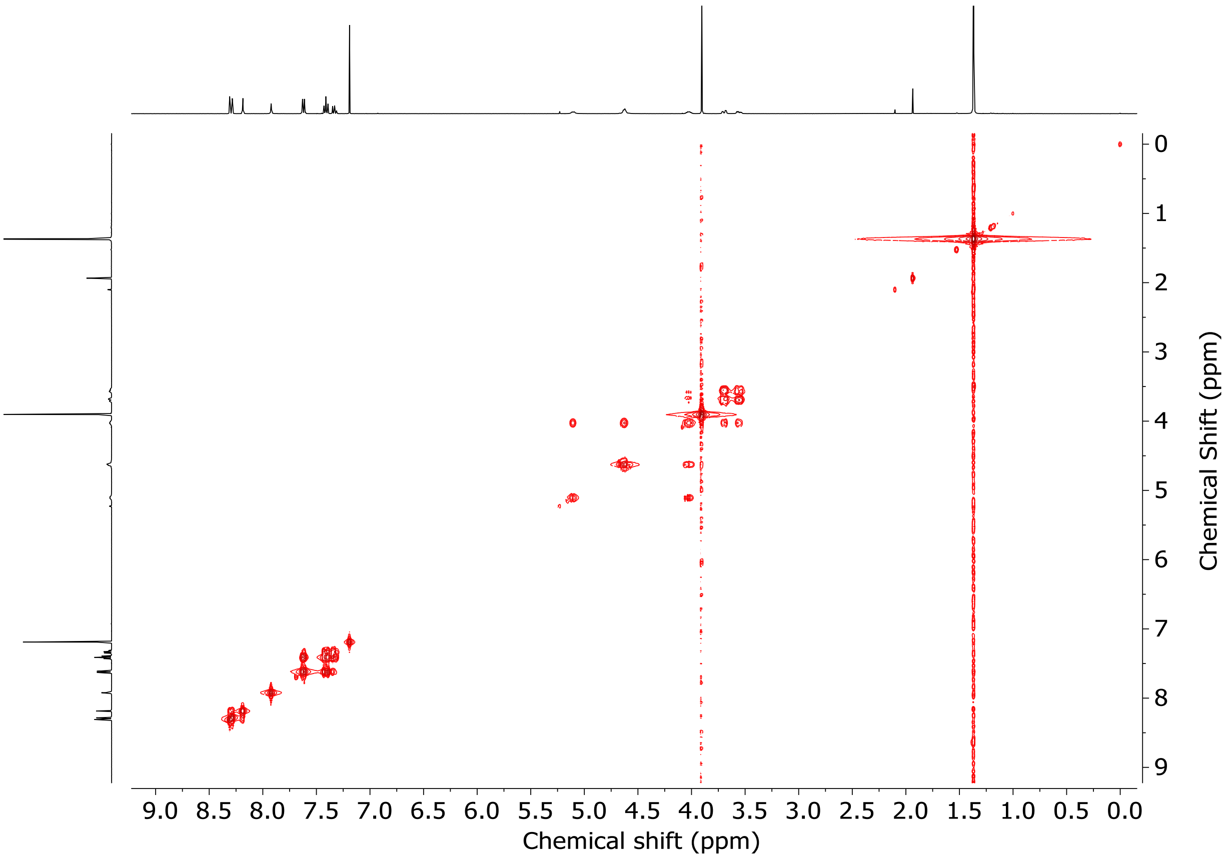

Figure S47: COSY NMR ($CDCl_3$) of (*S*)-**S4**.

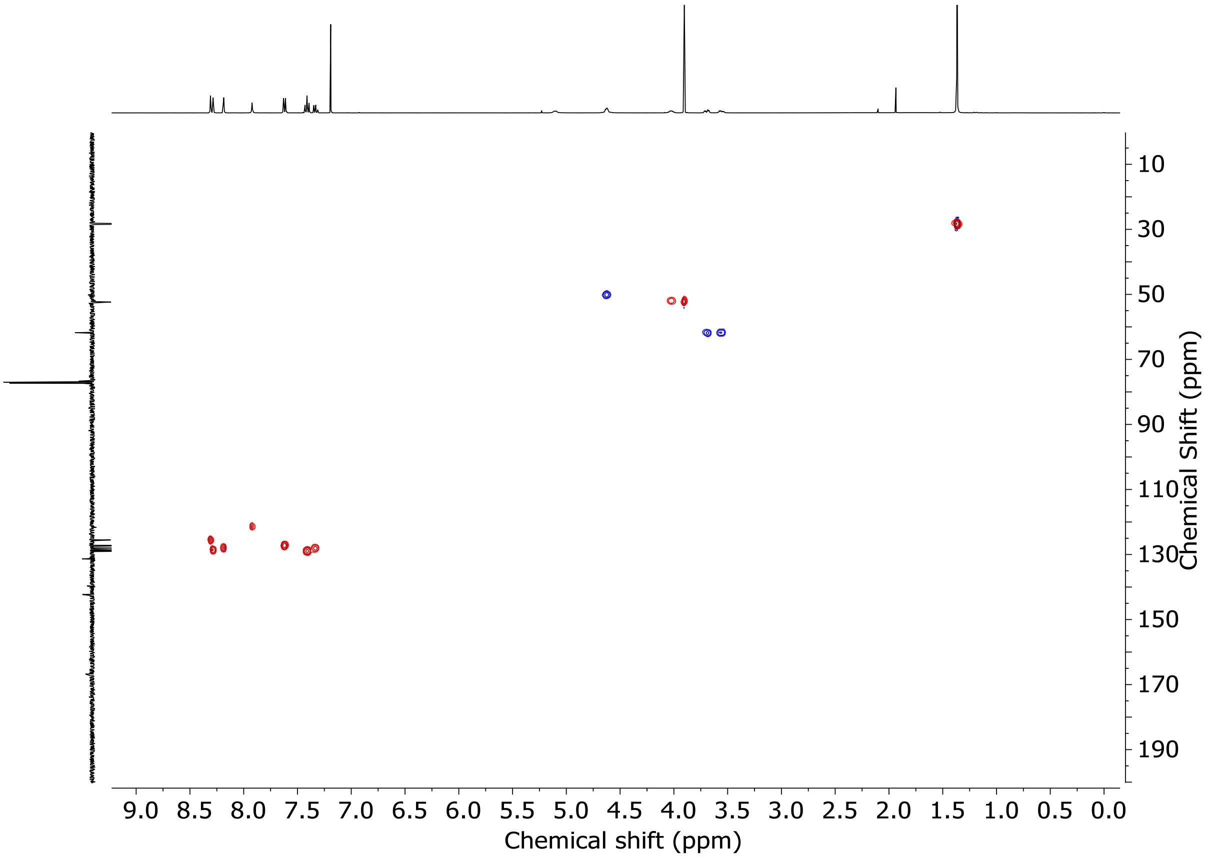

Figure S48: HSQC NMR ($CDCl_3$) of (*S*)-**S4**.

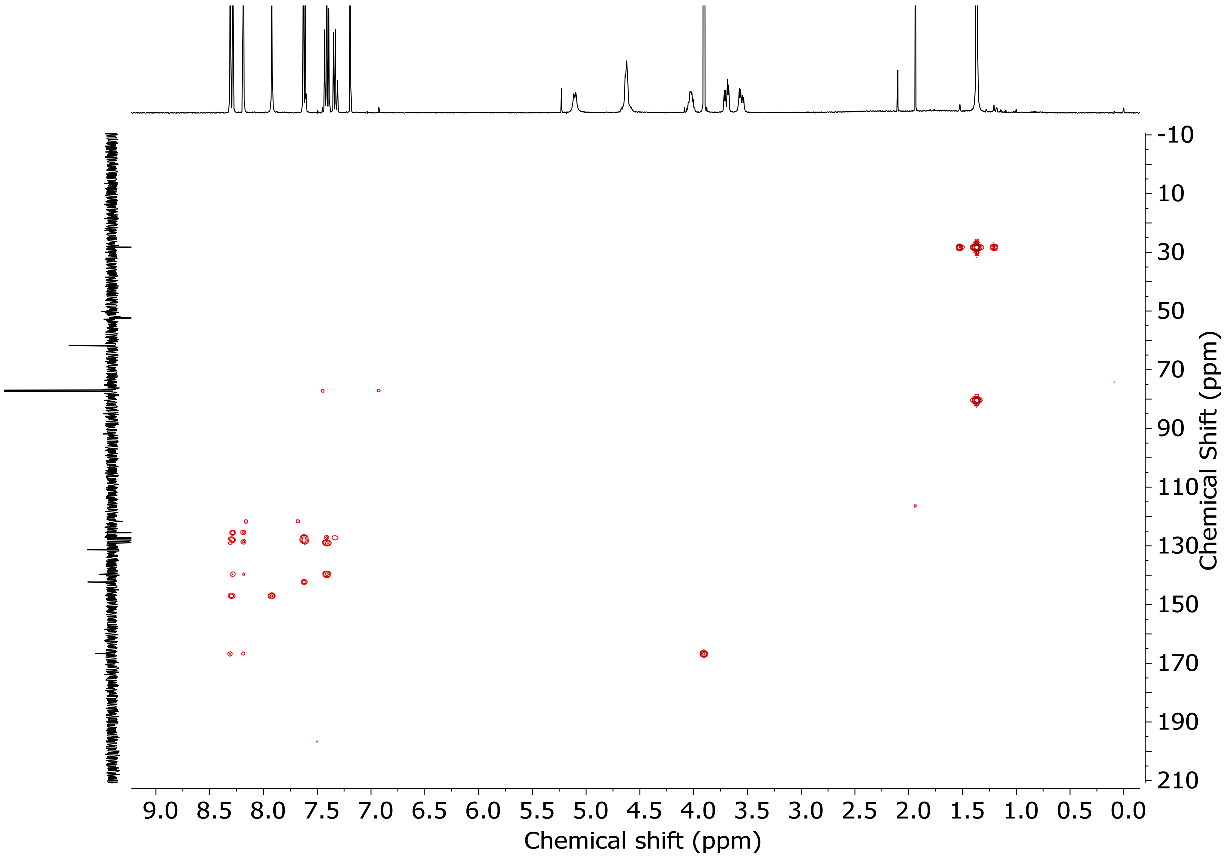

Figure S49: HMBC NMR ($CDCl_3$) of (*S*)-**S4**.

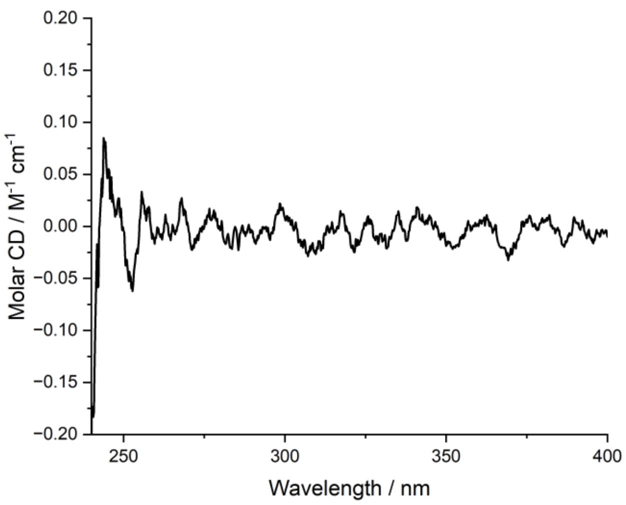

Figure S50: Circular dichroism spectra of (*S*)-**S4** (63.5 µM) at 293 K in $CHCl_3$. No measurable CD response was observed so the $[\alpha]_D$ of (*S*)-**S4** was measured.

Tosylate (*S*)-S5

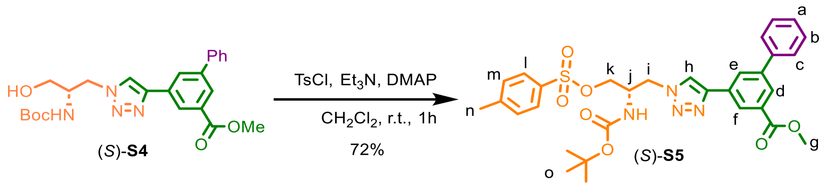

To a solution of (*S*)-**S4** (480 mg, 1.06 mmol), DMAP (13.0 mg, 0.11 mmol), and Et_3N (0.59 mL, 4.24 mmol) in CH_2Cl_2 (10 mL) was added TsCl (404.6 mg, 2.12 mmol). The solution turned yellow and was stirred at rt for 1h. H_2O (20 mL) was added, the phases separated, and the organic layer extracted with CH_2Cl_2 (2 x 50 mL). The collected organic fractions were then washed with brine (50 mL), then dried ($MgSO_4$). The solvent was removed *in vacuo* and the crude was purified by column chromatography (petrol-EtOAC 0→50%) to give (*S*)-**S5** as a white foam (460.4 mg, 72%). Decomposition of the product was observed upon standing in solution of CD_3CN (See Figure S52). The sample was too unstable to collect CD data.

^{1}H NMR (400 MHz, CD_3CN) δ: 8.42 (t, *J* = 1.5, 1H, H_f), 8.30 (t, *J* = 1.8, 1H, H_e), (t, *J* = 1.70, 1H, H_d), 8.20 (s, 1H, H_h), 7.81 (dt, *J* = 8.3, 2.0, 2H, H_l), 7.78-7.74 (m, 2H, H_c) 7.57-7.51 (m, 2H, H_b), 7.49-7.41 (m, 3H, H_a, H_m), 5.58 (d, *J* = 9.0, 1H, NH), 4.61-4.41 (m, 2H, H_i or H_k), 4.35-4.24 (m, 1H, H_j), 4.16-4.04 (m, 2H, H_i or H_k), 3.96 (s, 3H, H_g), 2.40 (s, 3H, H_n), 1.32 (bs, 9H, H_o)

^{13}C NMR (101 MHz, CD_3CN) δ: 167.0, 142.7, 140.1, 132.7, 132.5, 132.3, 130.7, 129.7, 128.8, 128.7, 128.5, 127.7, 126.5, 125.5, 123.0, 79.4 (HMBC), 69.6, 67.6, 53.6, 52.3, 50.6, 50.5, 28.0, 21.2

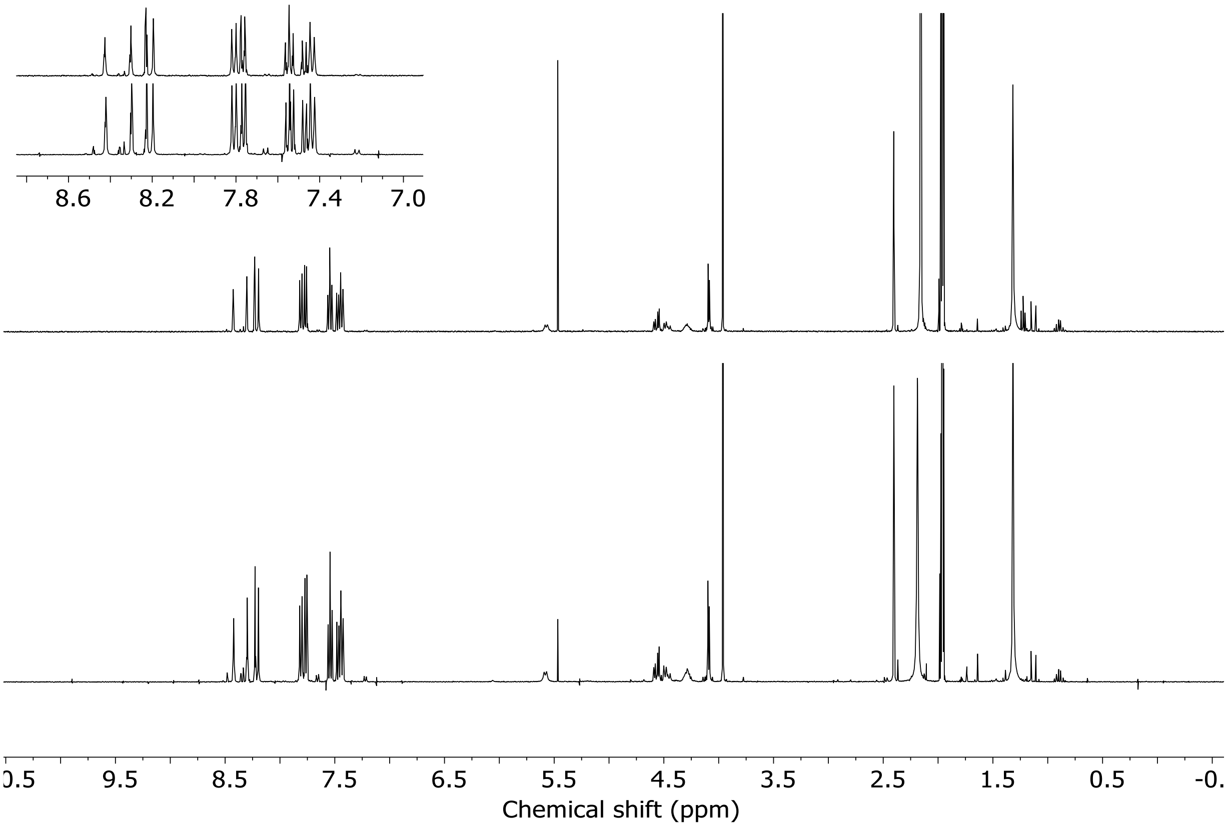

Figure S51: Stacked ^{1}H NMR spectrum of purified (*S*)-**S5** (top) and ^{1}H NMR spectrum of the same sample after 1 hour at room temperature to indicate degradation (bottom, 400 MHz, CD_3CN)

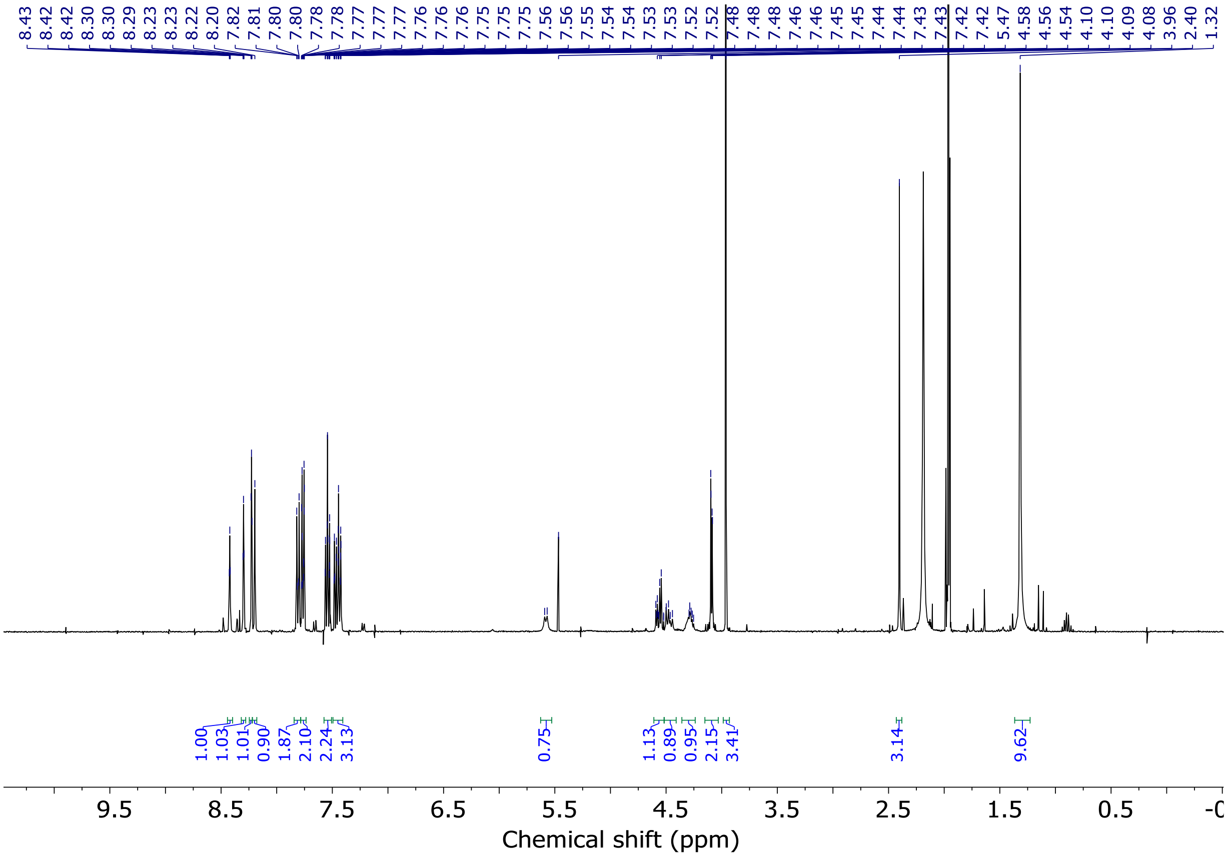

Figure S52: ^{1}H NMR (CD_3CN, 400 MHz) of (*S*)-**S5**.

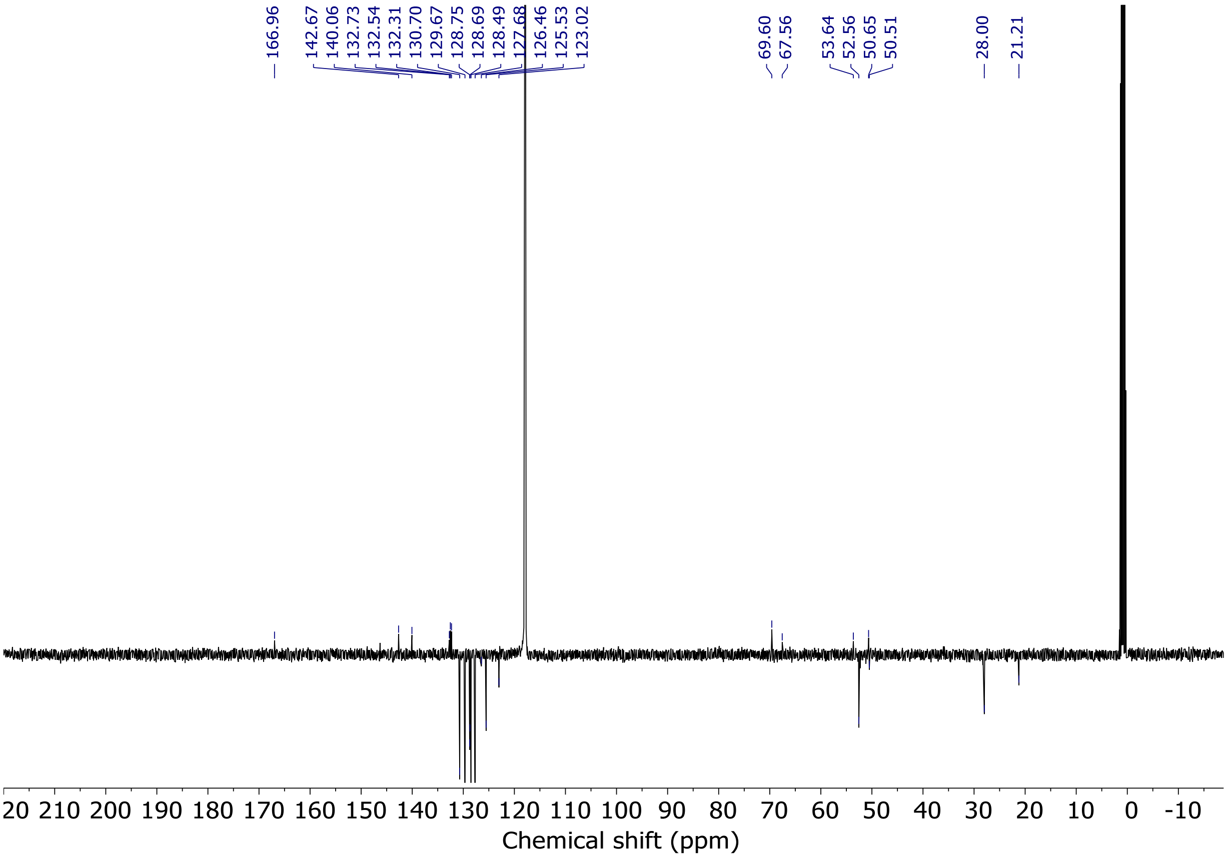

Figure S53: JMOD NMR (CD_3CN, 101 MHz) of (*S*)-**S5**.

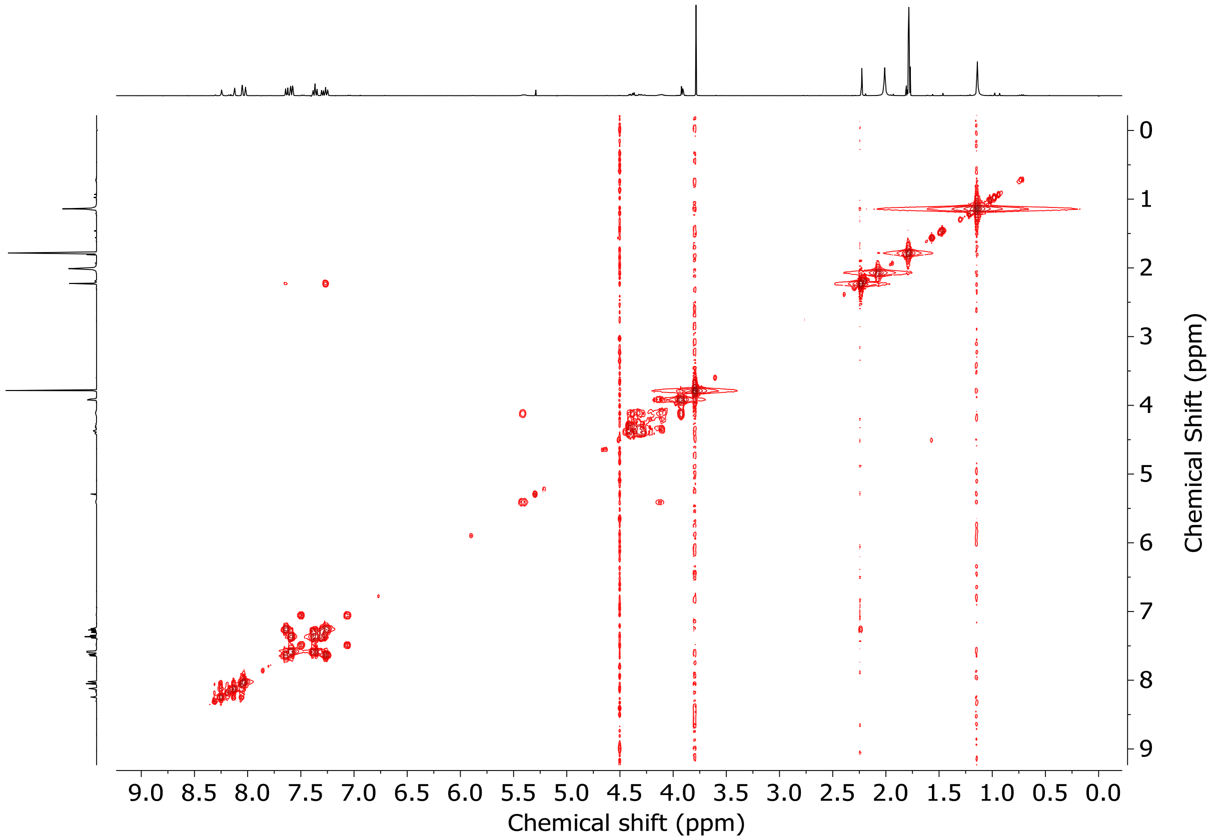

Figure S54: COSY NMR (CD_3CN) of (*S*)-**S5**.

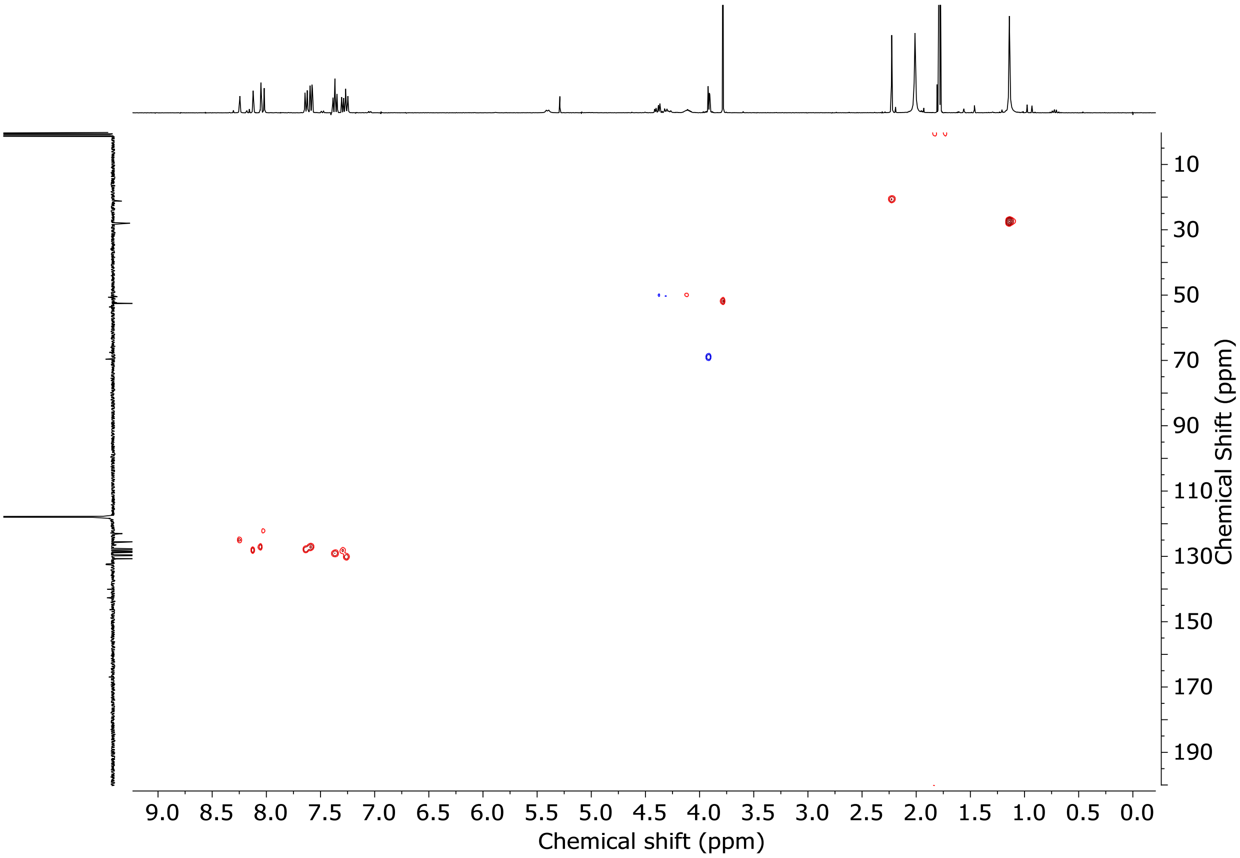

Figure S55: HSQC NMR (CD_3CN) of (*S*)-**S5**.

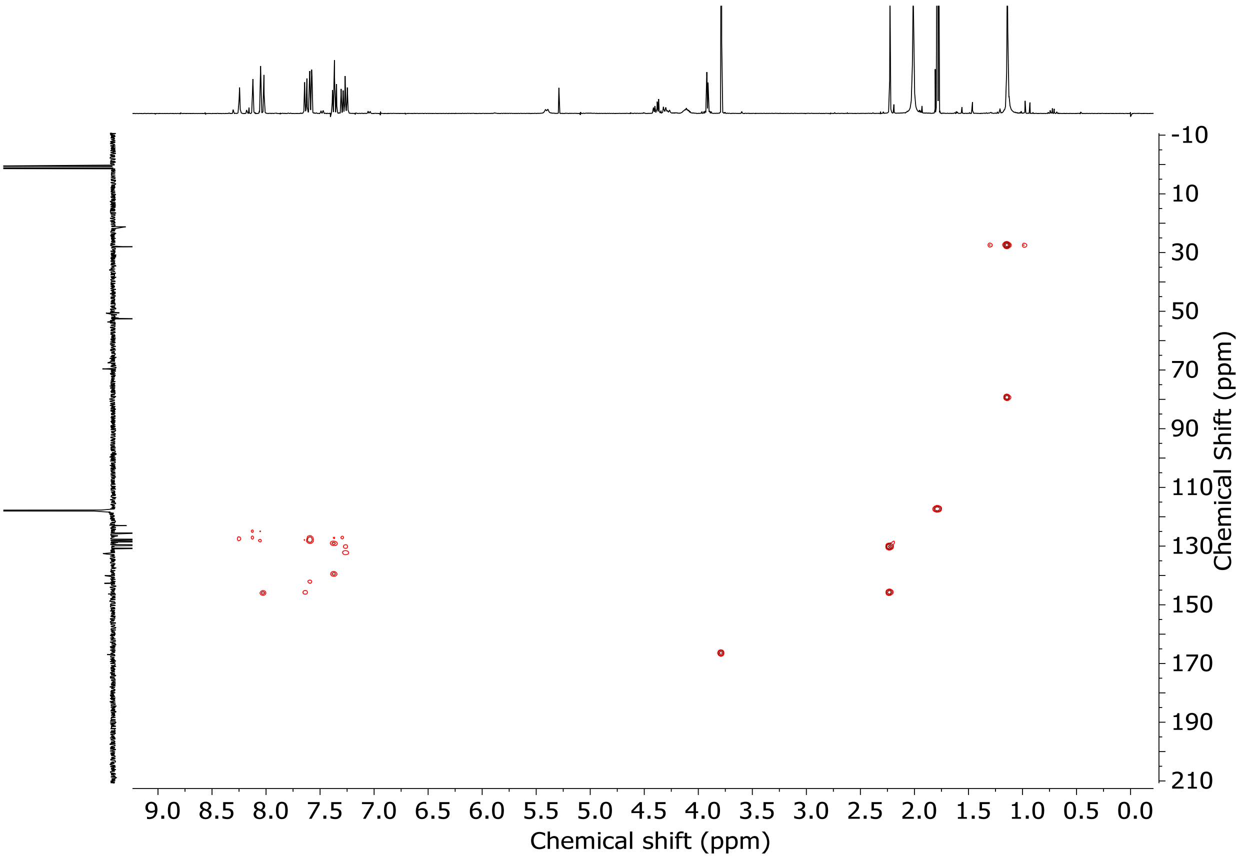

Figure S56: HMBC NMR (CD_3CN) of (*S*)-**S5**.

Azide (*S*)-S6

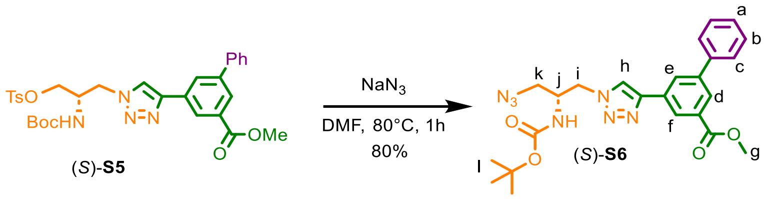

To a solution of (*S*)-**S5** (200 mg, 0.34 mmol) in DMF (4.0 mL) was carefully added NaN_3 (86 mg, 1.32 mmol). The suspension was heated at 80 °C for 1 h, then let cool down. 5% LiCl (10 mL) and Et_2O (15 mL) were added, the phases separated, and the organic layer extracted with Et_2O (2 x 15 mL). The collected organic fractions were then washed with brine (10 mL), then dried ($MgSO_4$). The solvent was removed *in vacuo* and the crude was purified by column chromatography (petrol-EtOAC 0→50%) to give (*S*)-**S6** as a white foam (129.3 mg, 80%).

^{1}H NMR (400 MHz, $CDCl_3$) δ: 8.38 (t, *J* = 1.6, 1H, H_f), 8.36 (t, *J* = 1.7, 1H, H_e), 8.26 (t, *J* = 1.6, 1H, H_f) 7.96 (s, 1H, H_h), 7.69 (dt, *J* = 7.5, 1.5, 2H, H_c), 7.48 (tt, *J* = 7.6, 1.4, 2H, H_b) 7.40 (tt, *J* = 7.4, 1.2, 1H, H_a), 5.13 (d, *J* = 8.8, 1H, NH), 4.61 (d, *J* = 5.7, 2H, H_i), 4.21 (app tq, *J* = 8.3, 5.6, 1H, H_j), 3.98 (s, 3H, H_g), 3.59 (dd, *J* = 12.6, 5.0, 1H, H_k), 3.39 (dd, 12.7, 6.5, 1H, $H_{k'}$), 1.43 (s, 9H, H_l)

^{13}C NMR (101 MHz, $CDCl_3$) δ: 166.7, 142.3, 139.7, 131.4, 131.1, 129.0, 128.6, 128.1, 128.1, 127.2, 125.5, 121.4, 80.7 (HMBC) 52.4, 51.3, 50.6, 50.1, 28.3.

HR-ESI-MS (+ve) *m/z* = 478.2201 $[M+H]^+$ (calc. 478.2197 *m/z* for $C_{24}H_{28}N_7O_4$);

$[\alpha]_D^{23}$+5.4 (c 0.55, $CHCl_3$)

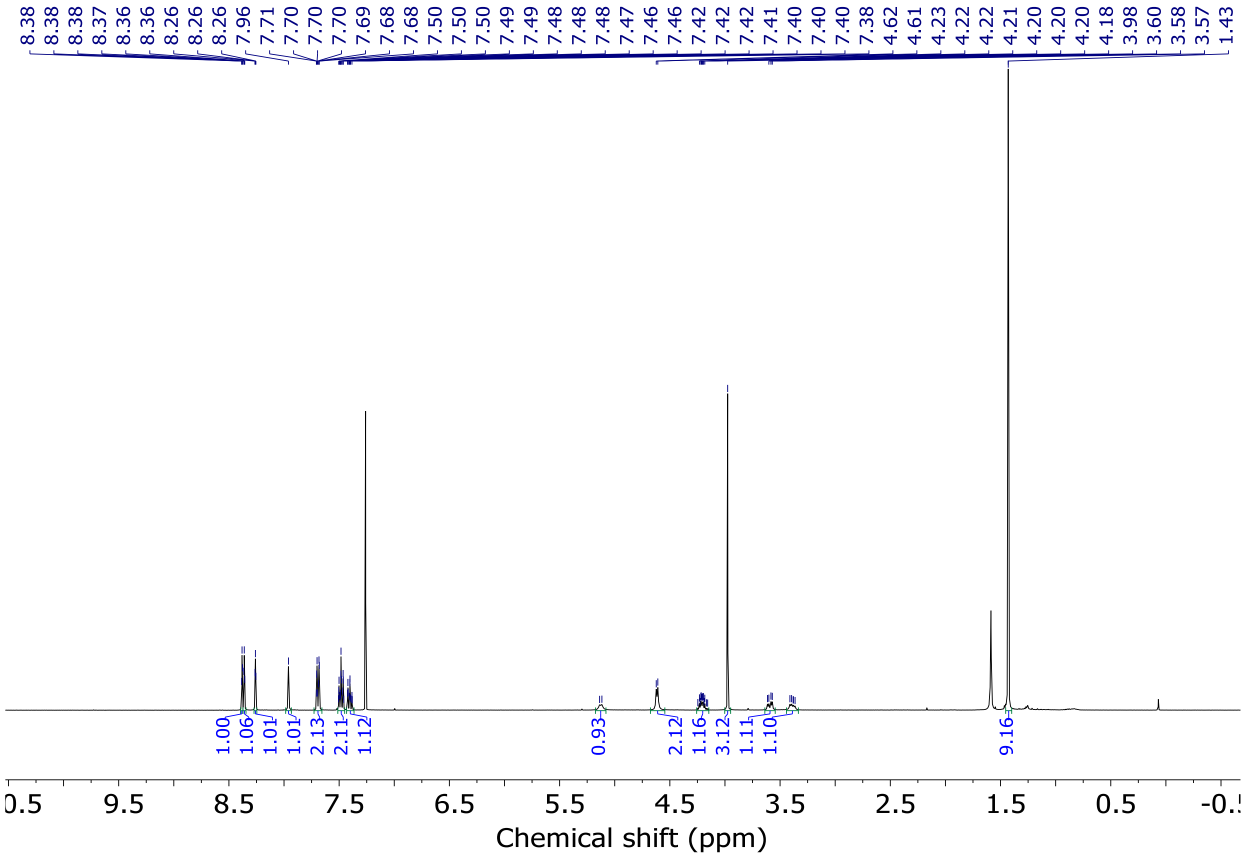

Figure S57: ^{1}H NMR ($CDCl_3$, 400 MHz) of (*S*)-**S6**.

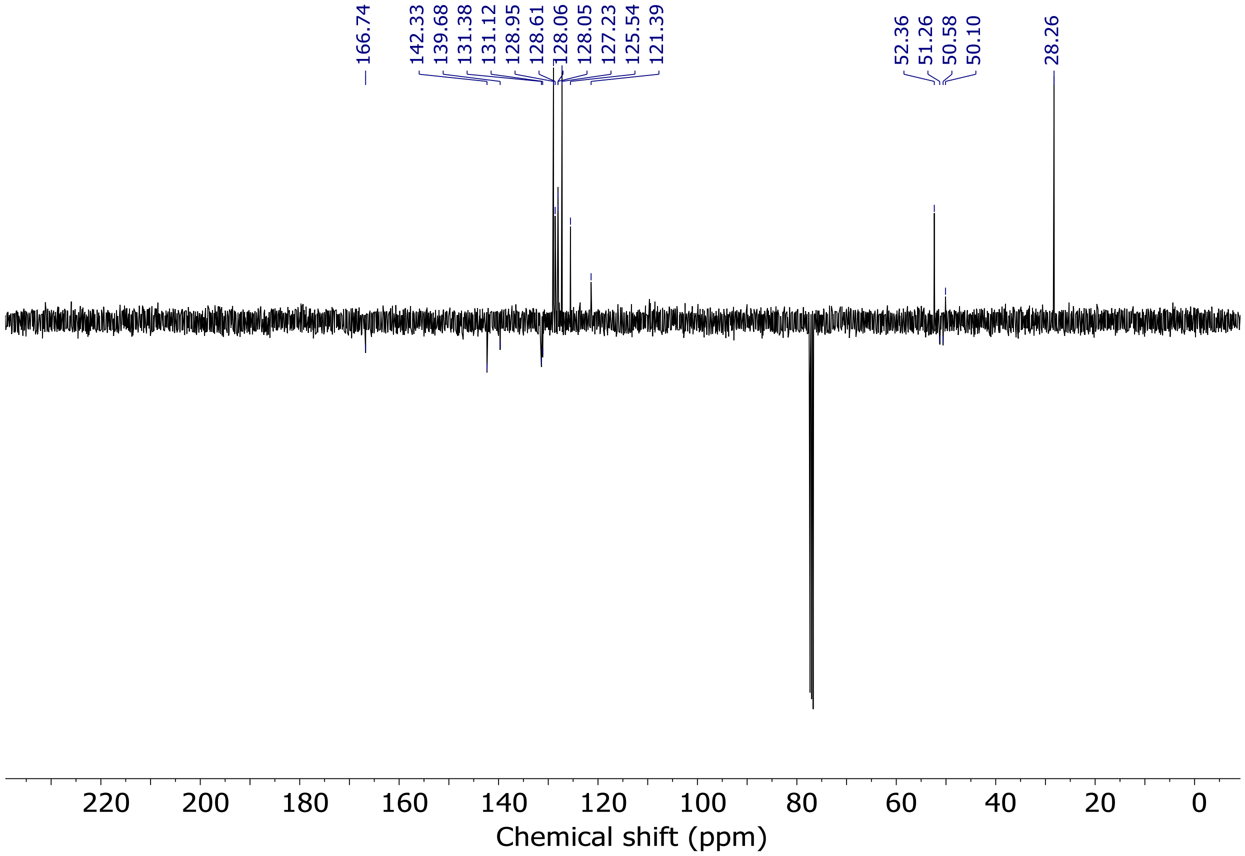

Figure S58: JMOD NMR ($CDCl_3$, 101 MHz) of (*S*)-**S6**.

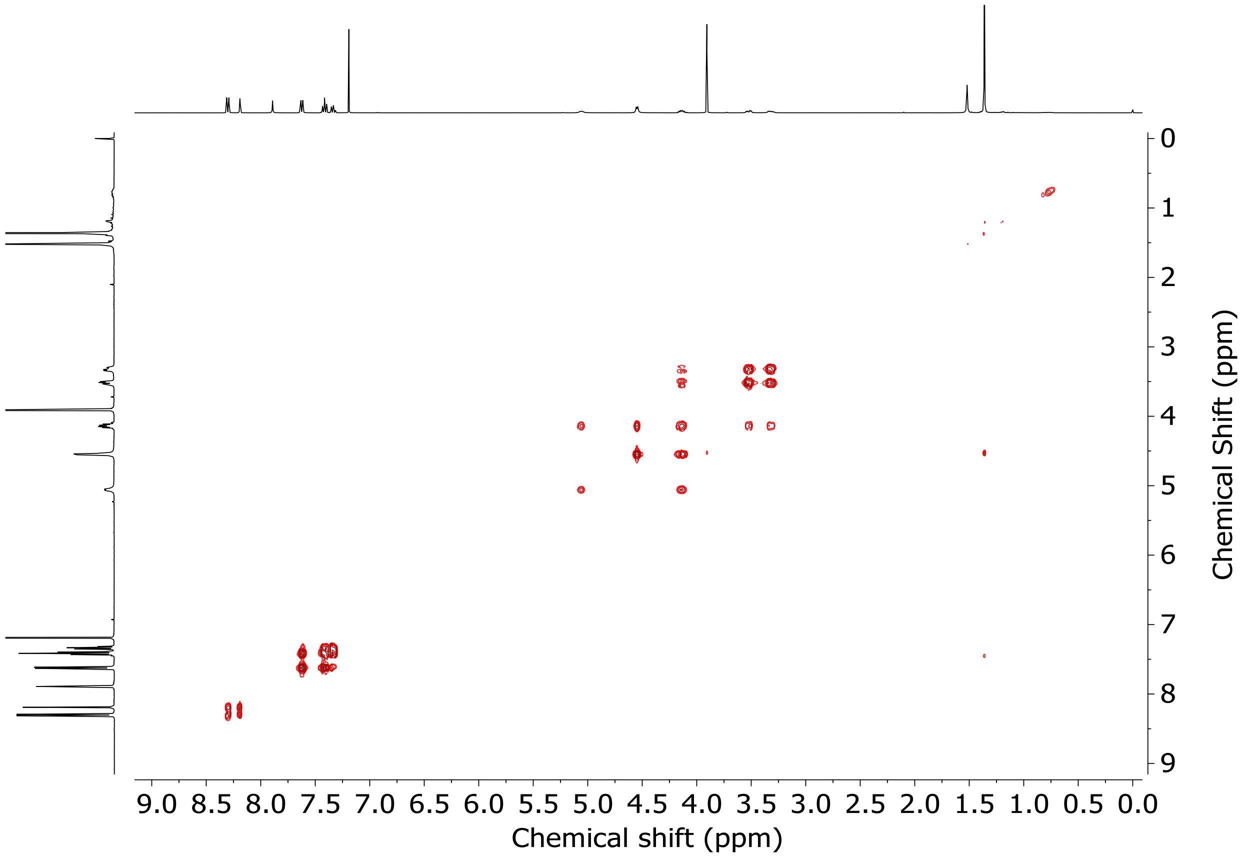

Figure S59: COSY NMR ($CDCl_3$) of (*S*)-**S6**.

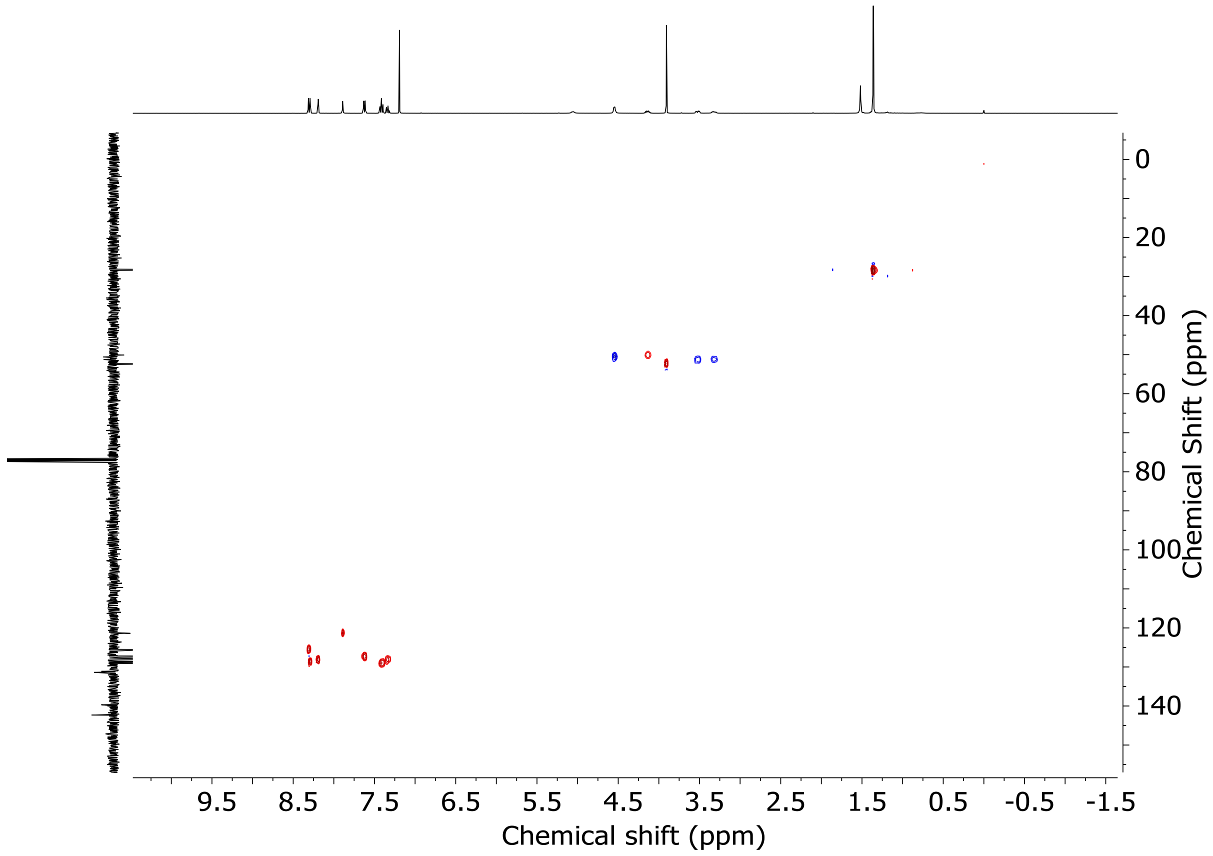

Figure S60: HSQC NMR ($CDCl_3$) of (*S*)-**S6**.

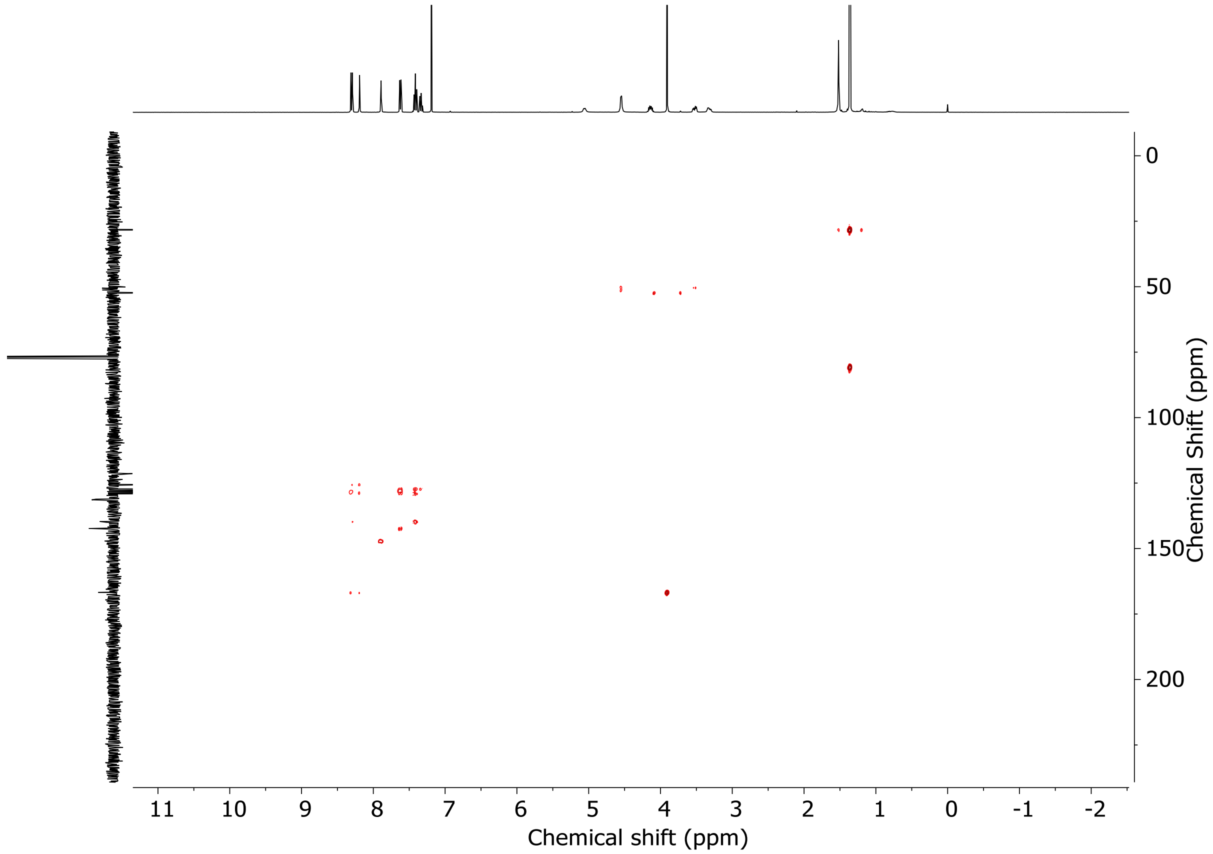

Figure S61: HMBC NMR ($CDCl_3$) of (*S*)-**S6**.

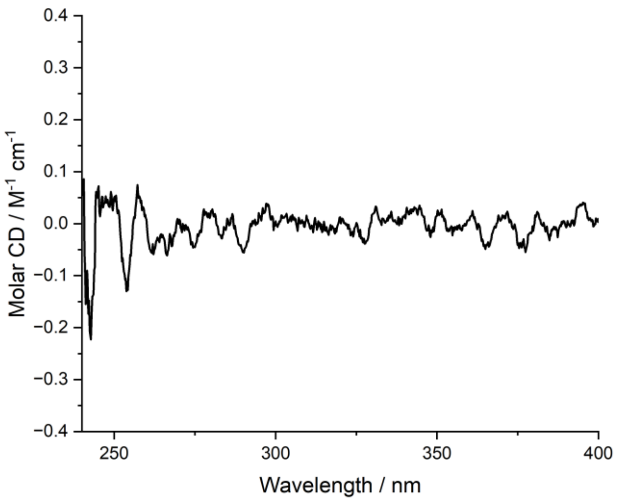

Figure S62: Circular dichroism spectra of (*S*)-**S6** (63.5 µM) at 293 K in $CHCl_3$. No measurable CD response was observed so the $[\alpha]_D$ of (*S*)-**S6** was measured.

1.7 Rotaxanes (E_m,S_{co-c})-10 and (Z_m,S_{co-c})-10 from (*S*)-S6

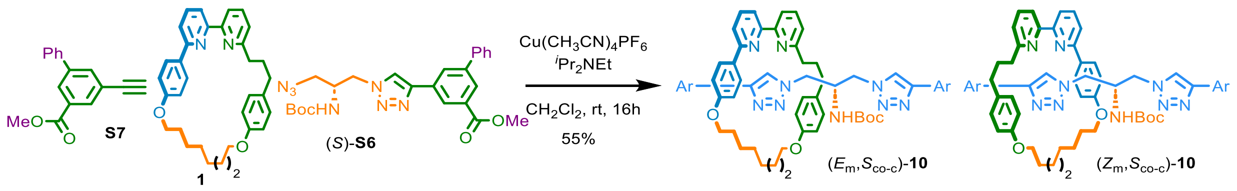

In a CEM vial were added (*S*)-**S6** (9.0 mg, 41.7 µmol), **S7** (9.7 mg, 41.7 µmol), **1** (20.0 mg, 38.0 µmol) and [$Cu(CH_3CN)_4PF_6$] (13.6 mg, 36.5 µmol). The vial was sealed and purged with N_2, then CH_2Cl_2 was added (1.0 mL), followed by ^{i}Pr_2NEt (13.3 µL, 75.9 µmol). The solution was stirred at rt for 16 h. MeOH (2 mL) and KCN as a solid (24.7 mg, 0.38 mmol) were added and the resulting mixture was stirred vigorously until complete decolouration. The crude mixture was diluted with CH_2Cl_2 (5 mL) and washed with H_2O (5 mL) then EDTA-NH_3 (5 mL), with separation of aqueous and organic phases. The combined aqueous phase was then extracted with CH_2Cl_2 (3 x 5 mL) and the combined organic extracts were washed with brine (10 mL), dried ($MgSO_4$) and concentrated *in vacuo* to give a sample containing **10** as a mixture of diastereomers (58 : 42 *dr*, Figure S64).

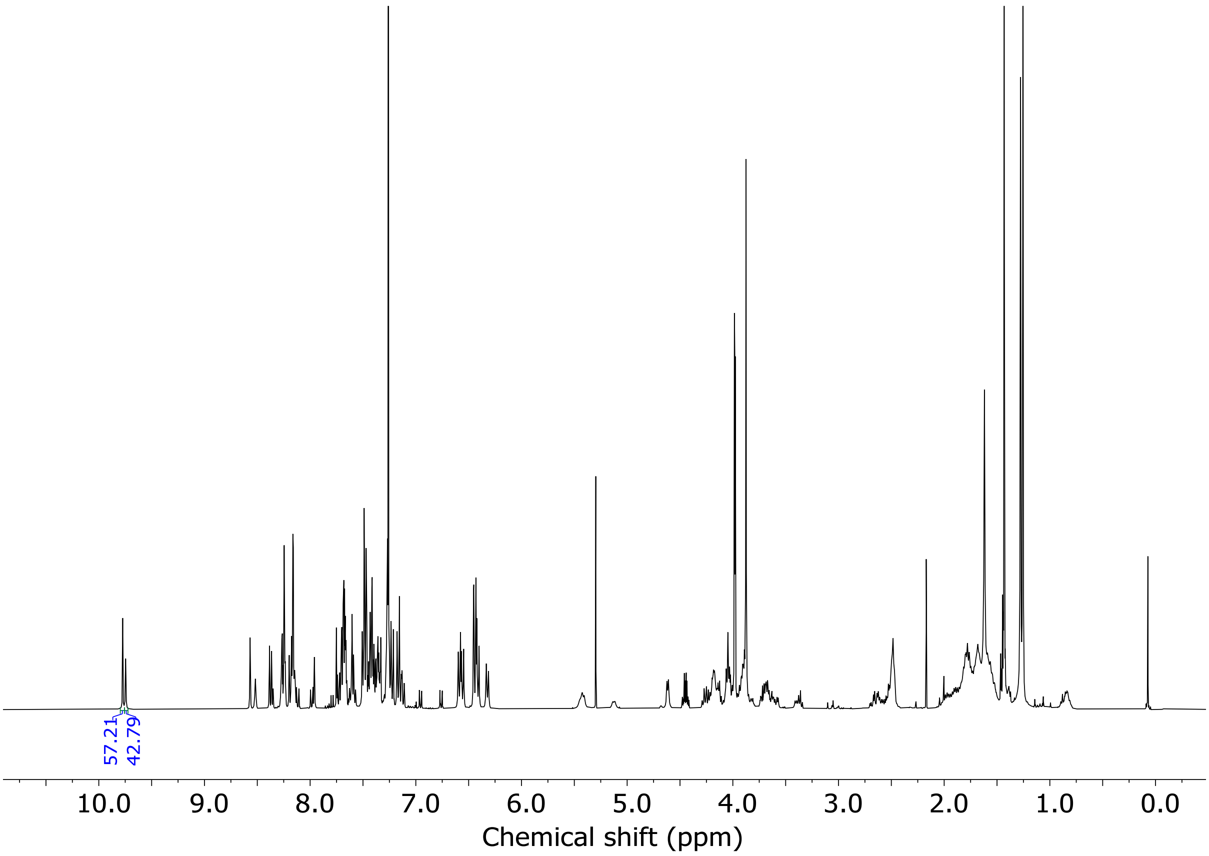

Figure S63: ^{1}H NMR ($CDCl_3$, 400 MHz) of (E_m)-**10** and (Z_m)-**10** prior to chromatography (57 : 43 *dr*).

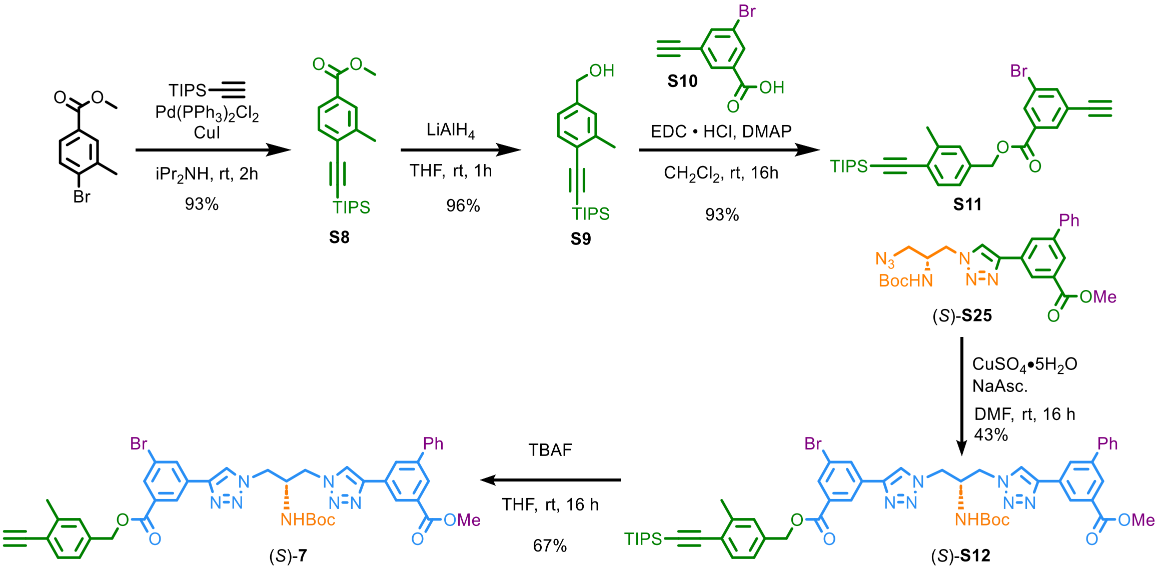

Scheme S2. Synthetic route to shuttle alkyne (*S*)-**7**.

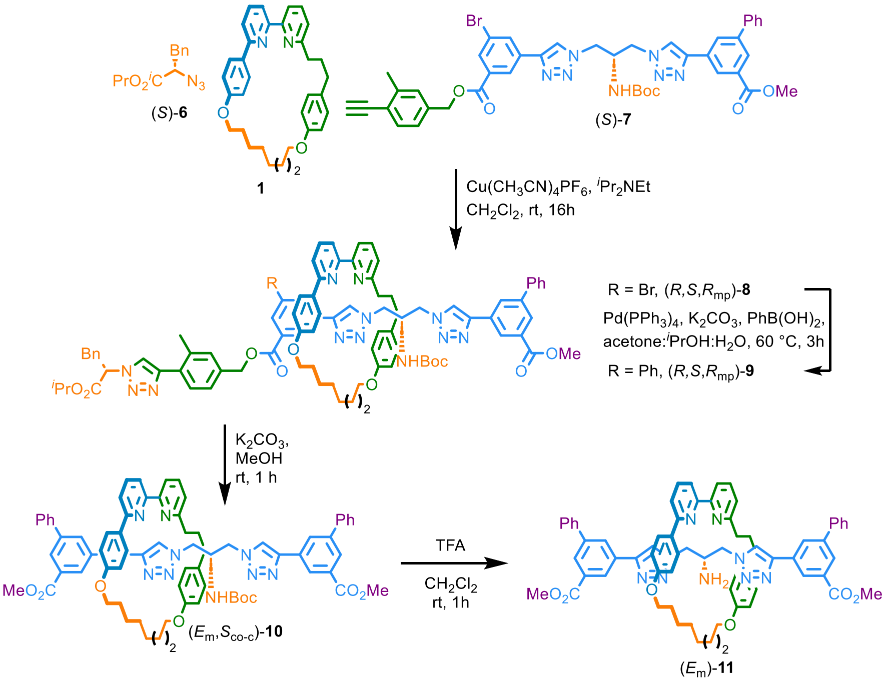

Scheme S3. Synthetic route to diastereoenriched rotaxanes **11** *via* a shuttling approach. Synthesis of (E_m)-**11** shown.

TIPS alkyne S8

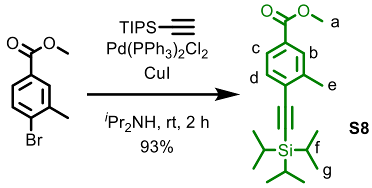

Methyl 4-bromo-3-methylbenzoate (2.5 g, 10.9 mmol), $PdCl_2(PPh_3)_2$ (387 mg, 0.546 mmol), and CuI (214 mg, 1.09 mmol) were suspended in a mixture of $^{i}Pr_2NH$ (8 ml, 54.6 mmol) and THF (25 ml), before the reaction vessel was purged with nitrogen. Ethynyltri*iso*propylsilane (3ml, 13.1 mmol) was then added to the vessel before the reaction was heated at 80 °C overnight. The reaction mixture was allowed to cool, filtrated through celite®, washed with Et_2O, and dried *in vacuo*. Chromatography (petrol-EtOAc 0→5%) gave **S8** (3.347 g, 93%) as a brown oil.

^{1}H NMR (400 MHz, $CDCl_3$) δ: 7.88 (dq, *J* = 1.7, 0.7, 1H, H_b), 7.78 (ddq, *J* = 8.0 1.6, 0.6, 1H, H_c), 7.49 (app. dt, 8.0, 0.4, 1H, H_d), 3.91 (s, 3H, H_a), 2.50 (s, 3H, H_e), 1.16-1.11 (m, 21H, H_f, H_g)

^{13}C NMR (101 MHz, $CDCl_3$) δ: 166.8, 140.7, 132.3, 130.3, 129.4, 128.0, 126.6, 104.9, 98.3, 52.2, 20.9, 18.7, 11.3.

HR-ESI-MS (+ve) *m/z* = 330.2090 $[M+H]^+$ (calc. 330.2081 *m/z* for $C_{20}H_{30}O_2Si$);

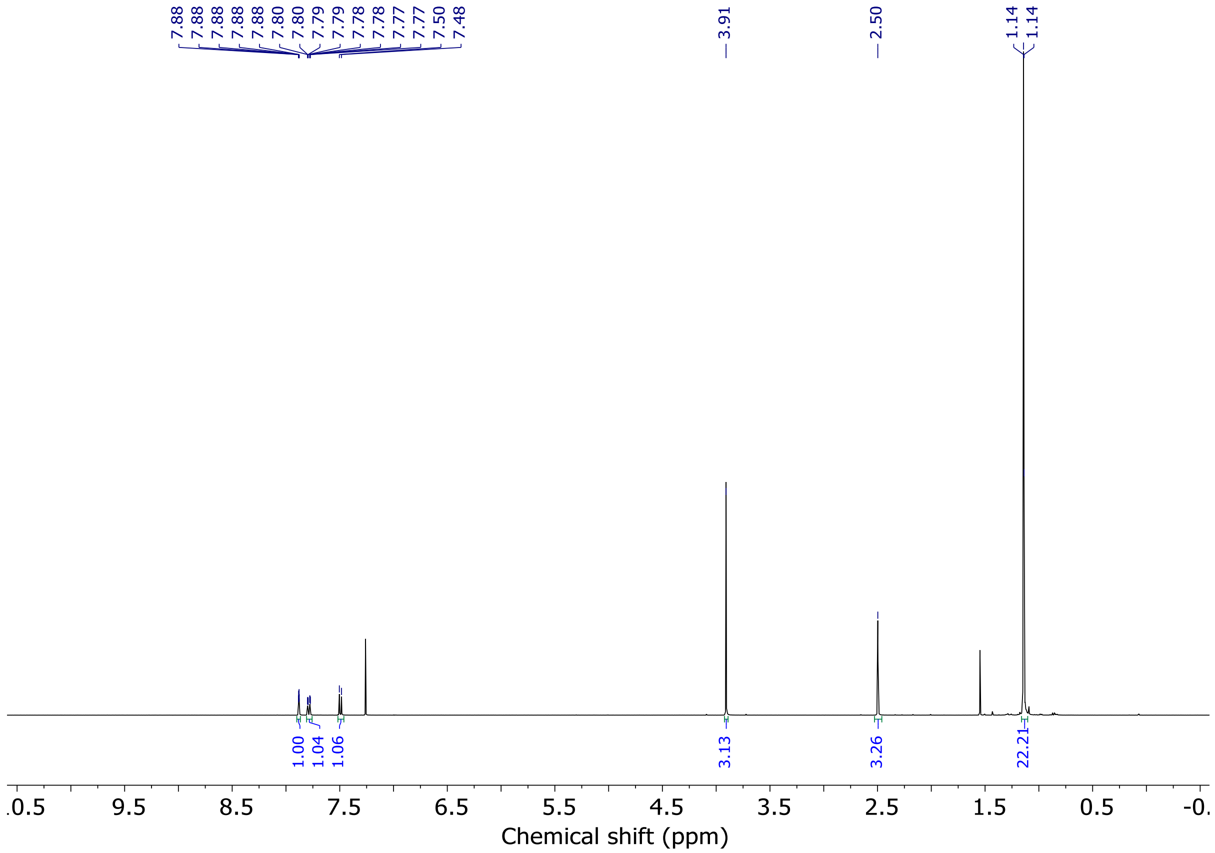

Figure S64: ^{1}H NMR ($CDCl_3$, 400 MHz) of **S8**.

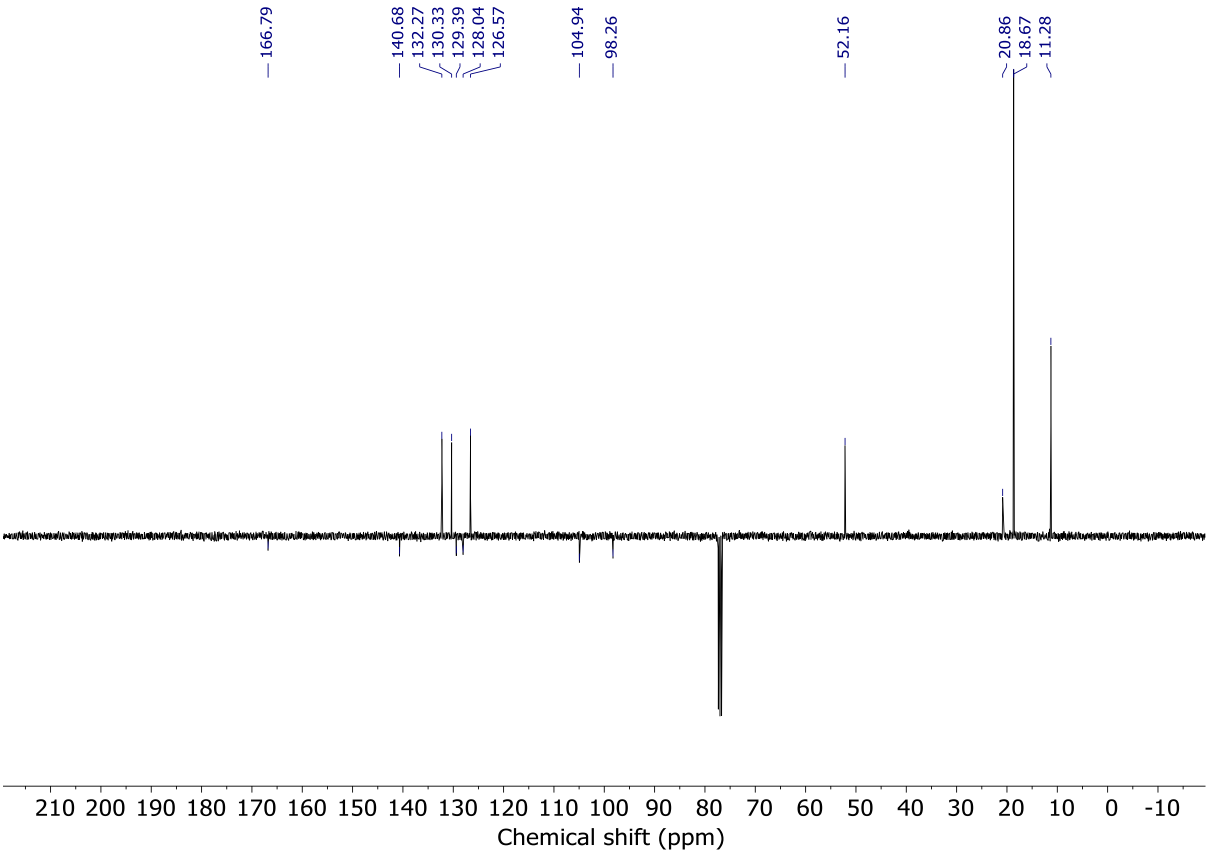

Figure S65: JMOD NMR ($CDCl_3$, 101 MHz) of **S8**.

TIPS alkyne S9

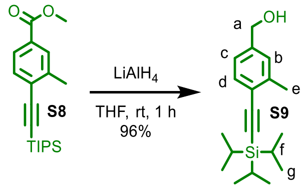

$LiAlH_4$ (695 mg, 18.2 mmol) was suspended in THF (30 ml) and was cooled to 0 °C. **S8** (3.0 g, 9.08 mmol) was added and the reaction mixture was stirred at rt. for 30 mins. The reaction was then quenched using a 1 M HCl/EtOAc/ice mixture to destroy excess $LiAlH_4$. The organic layer was then extracted using EtOAc (100 ml). The combined organic layers were dried ($MgSO_4$), and solvents removed *in vacuo*. Chromatography (petrol-EtOAc 0→10%) gave the product **S9** (2.628 g, 96%) as a yellow oil.

^{1}H NMR (400 MHz, $CDCl_3$) δ: 7.44 (d, *J* = 7.9, 1H, H_d), 7.20 (app. 8plet, *J* = 0.6, 1H, H_b), 7.12 (ddq, *J* = 7.9, 1.8, 0.6, 1H, H_c) 4.65 (d, *J* = 5.9, 2H, H_a), 2.47 (s, 3H, H_e), 1.68 (t, *J* = 6.0, 1H, OH), 1.14 (app. s, 21H, H_f, H_g)

^{13}C NMR (101 MHz, $CDCl_3$) δ: 141.0, 132.6, 127.9, 123.9, 122.7, 105.6, 94.6, 65.1, 20.9, 18.7, 11.3.

HR-ESI-MS (+ve) *m/z* = 302.2066 $[M+H]^+$ (calc. 302.2053 *m/z* for $C_{19}H_{30}OSi$).

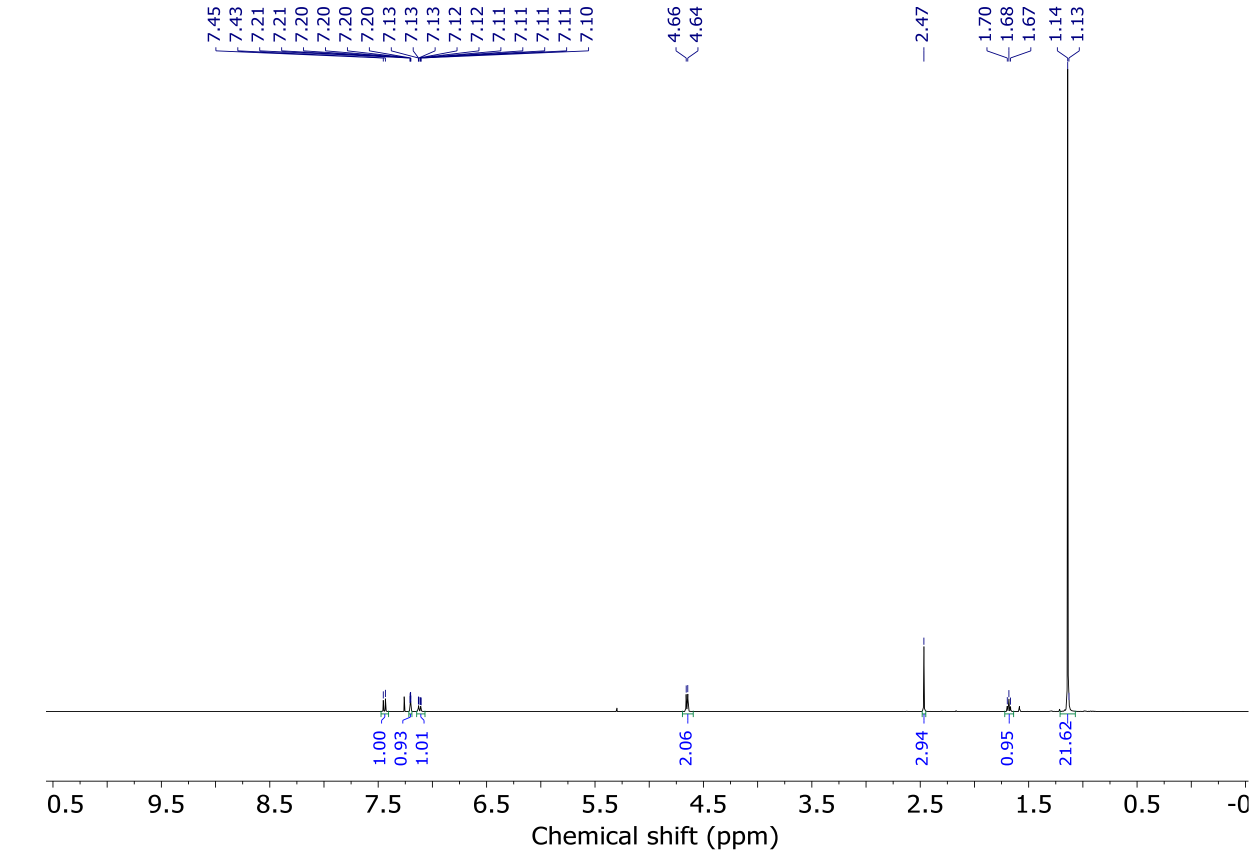

Figure S66: ^1^H NMR ($CDCl_3$, 400 MHz) of **S9**.

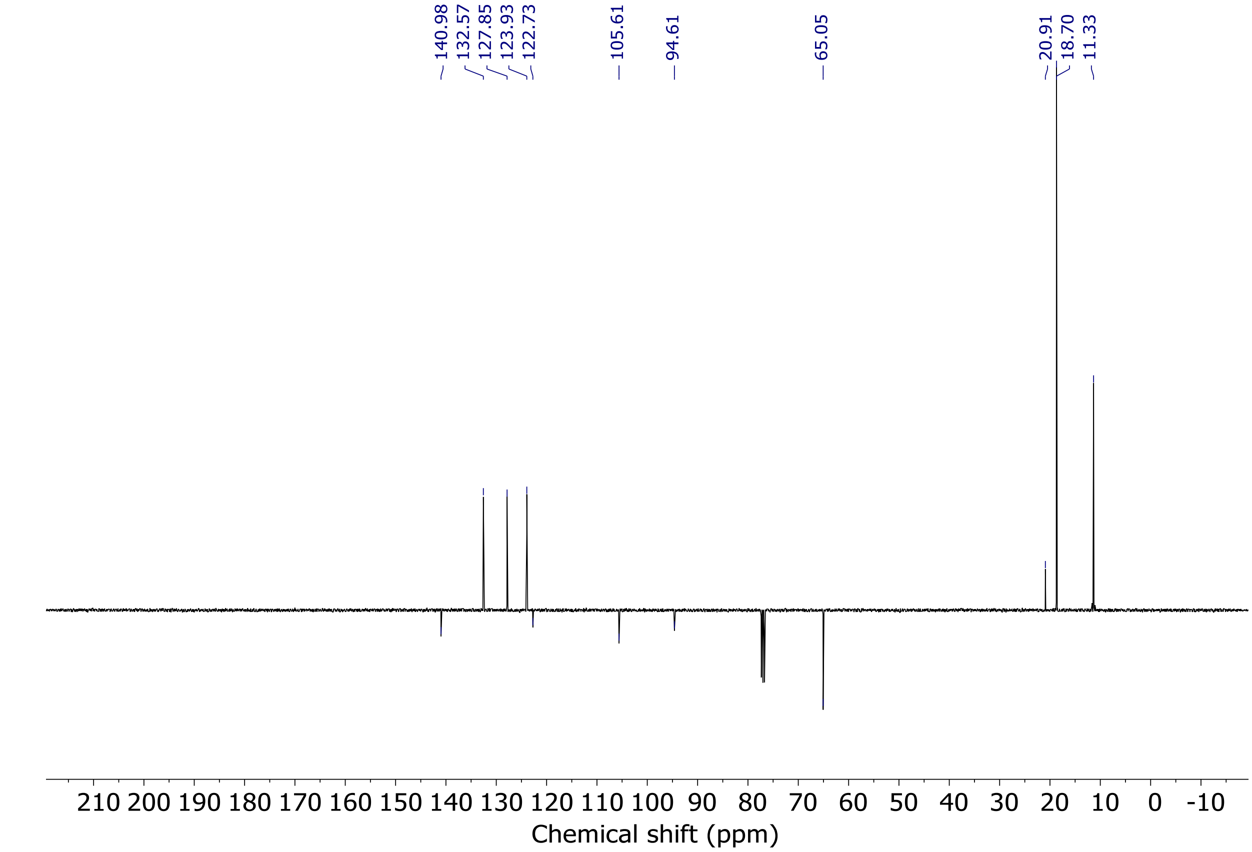

Figure S67: JMOD NMR ($CDCl_3$, 101 MHz) of **S9**.

TIPS alkyne S11

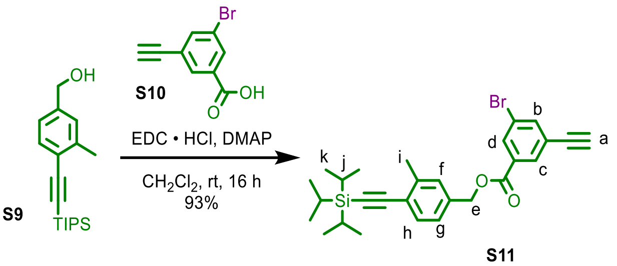

To a solution of **S9** (2.5 g, 7.6 mmol), **S10** (1.8 g, 8.4 mmol), DMAP (92.9 mg, 0.76 mmol) and CH_2Cl_2 (100 mL) was added EDC · HCl (3.0 g, 15.2 mmol). The reaction mixture was stirred at rt for 16 h. H_2O (50 mL) was added, then the aqueous and organic phases were separated, and the aqueous phase was then extracted with CH_2Cl_2 (3 x 50 mL). The combined organic extracts were washed with brine (100 mL), dried ($MgSO_4$) and concentrated *in vacuo*. Chromatography (petrol-EtOAc 0→5%) gave **S11** as a white solid (3.6 g, 93%).

^{1}H NMR (400 MHz, $CDCl_3$) δ: 8.15 (t, *J* = 1.6, 1H, H_d), 8.08 (t, *J* = 1.5, 1H, H_c), 7.79 (t, *J* = 1.6, 1H, H_b), 7.48 (d, *J* = 7.9, 1H, H_h), 7.28-7.24 (m, 1H, H_f superimposed with $CHCl_3$), 7.20 (d, *H* = 8.1, 1H, H_g) 5.22 (s, 2H, H_e), 3.09 (s, 1H, H_a), 2.40 (s, 3H, H_i), 1.06 (app. s, 21H, H_f, H_g)

^{13}C NMR (101 MHz, $CDCl_3$) δ: 164.3, 141.0, 138.9, 135.3, 132.9, 132.7, 132.0, 131.9, 129.5, 125.6, 124.4, 123.8, 122.2, 105.3, 95.5, 81.1, 79.6, 79.0, 67.1, 20.9, 18.7, 11.3.

HR-ESI-MS (+ve) *m/z* = 509.5614 $[M+H]^+$ (calc. 509.5609 *m/z* for $C_{28}H_{33}BrO_2Si$).

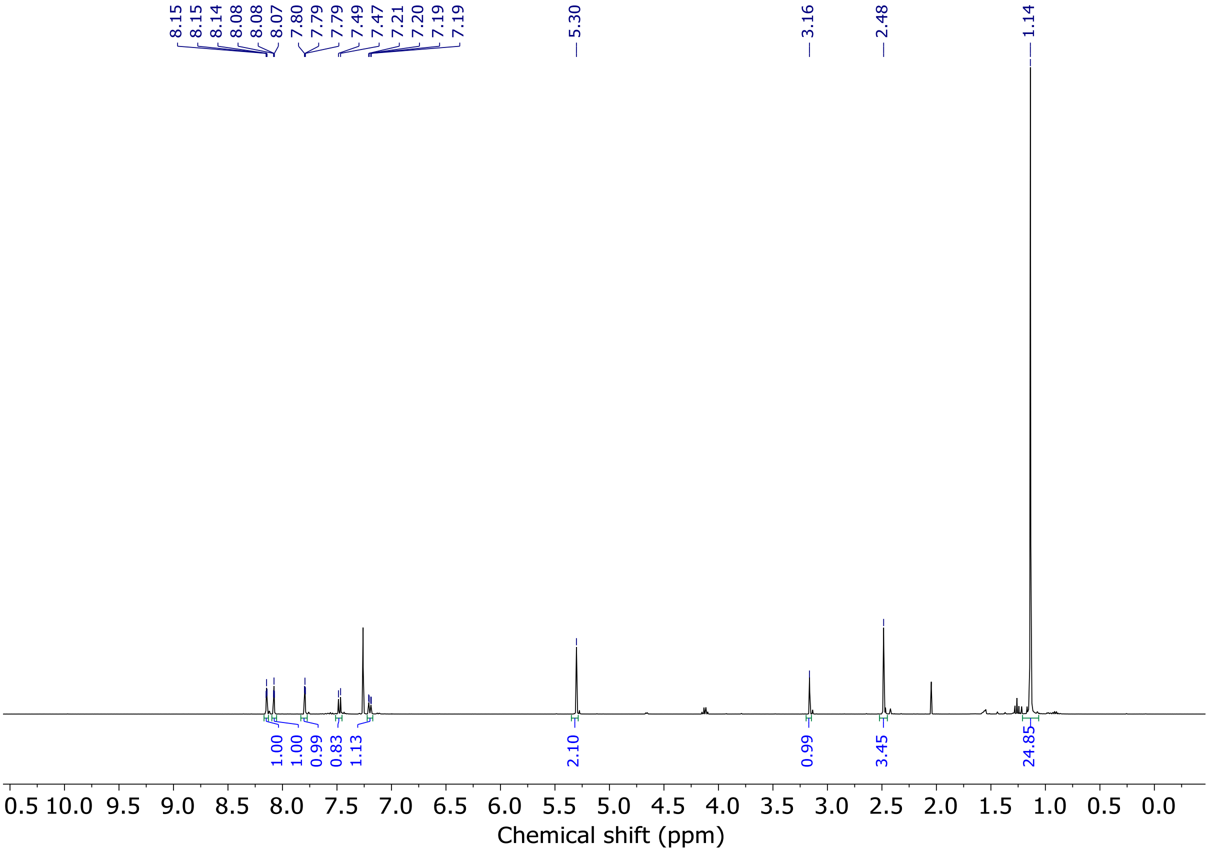

Figure S68: ^{1}H NMR ($CDCl_3$, 400 MHz) of **S11**.

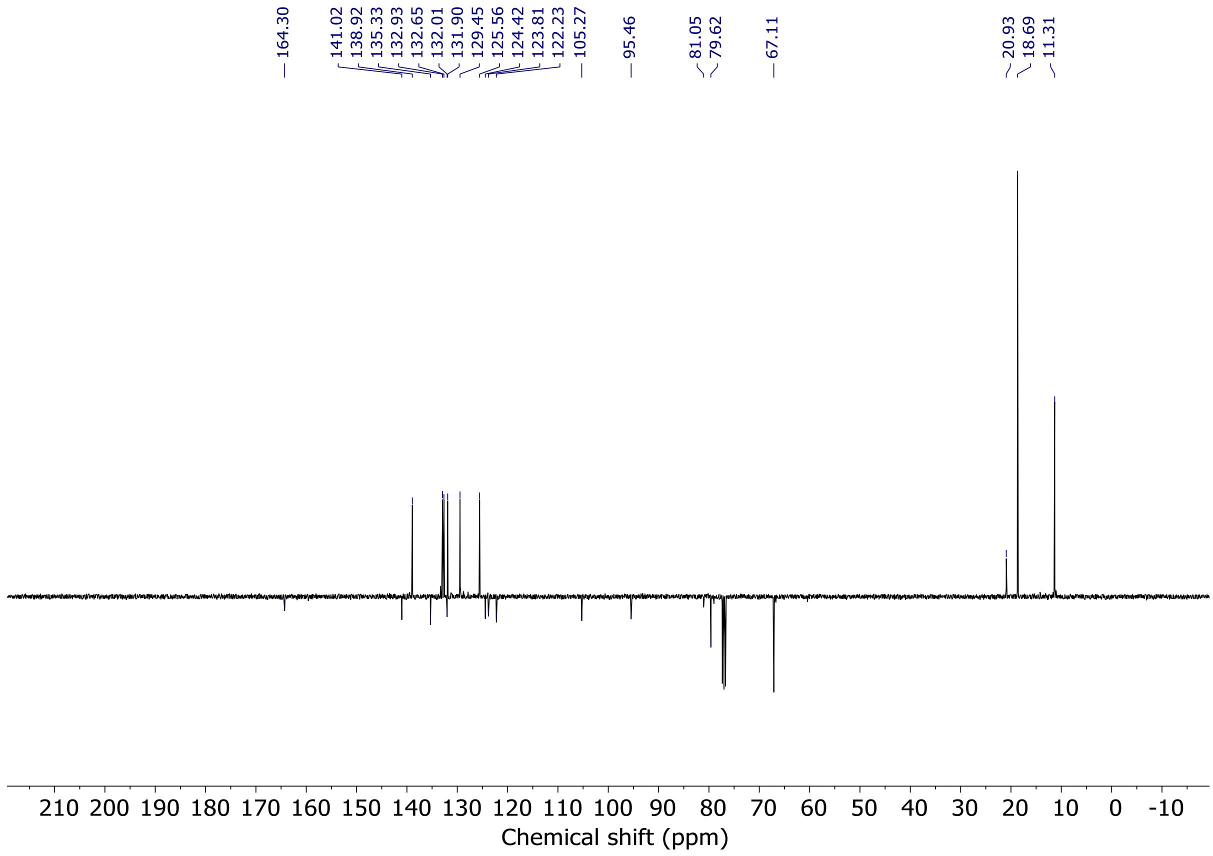

Figure S69: JMOD NMR ($CDCl_3$, 101 MHz) of **S11**.

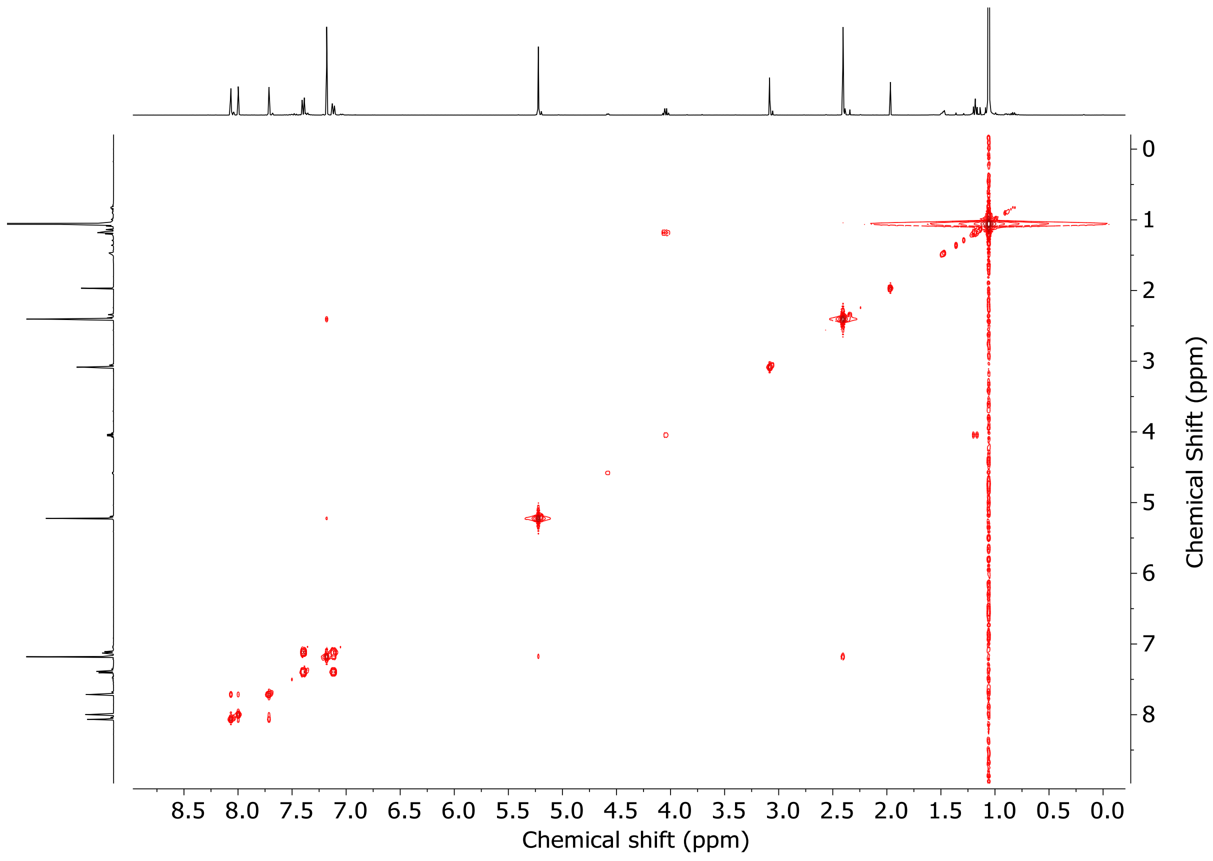

Figure S70: COSY NMR ($CDCl_3$) of **S11**.

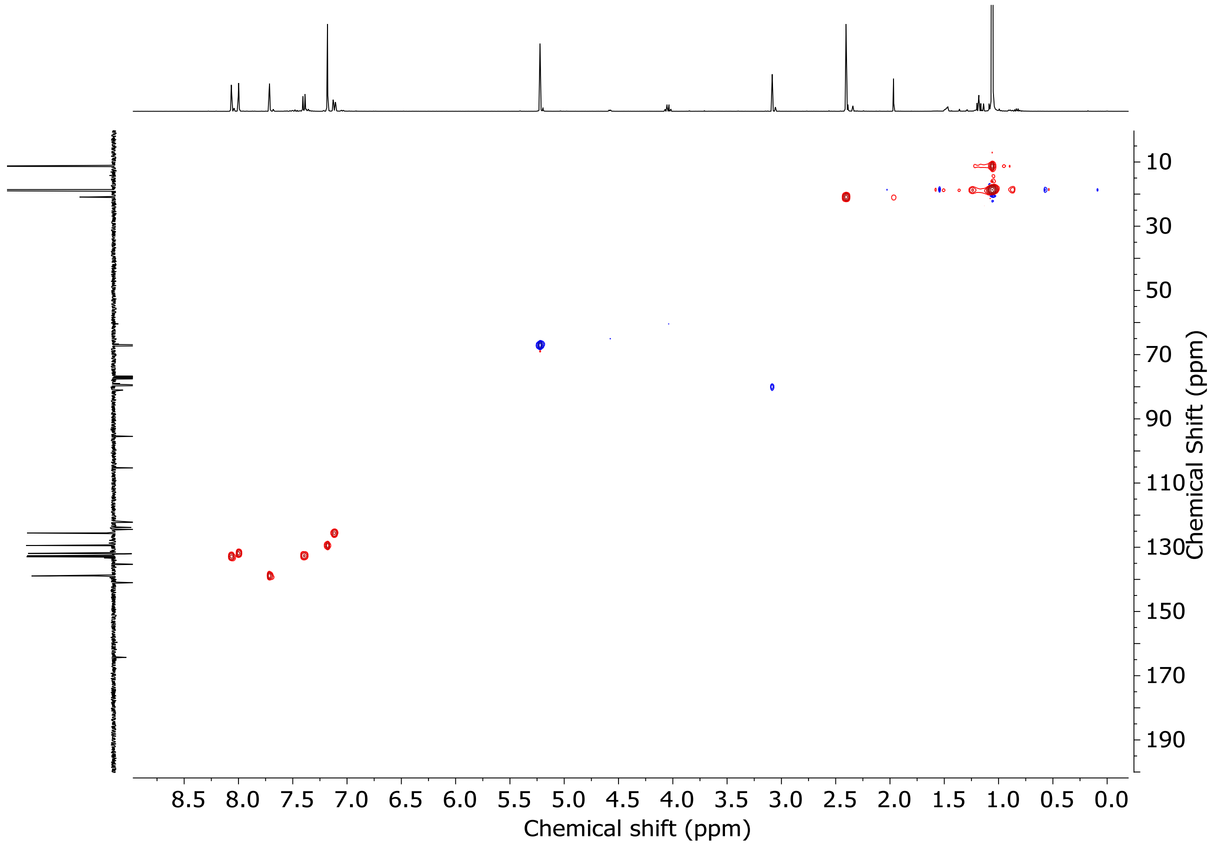

Figure S71: HSQC NMR ($CDCl_3$) of **S11**.

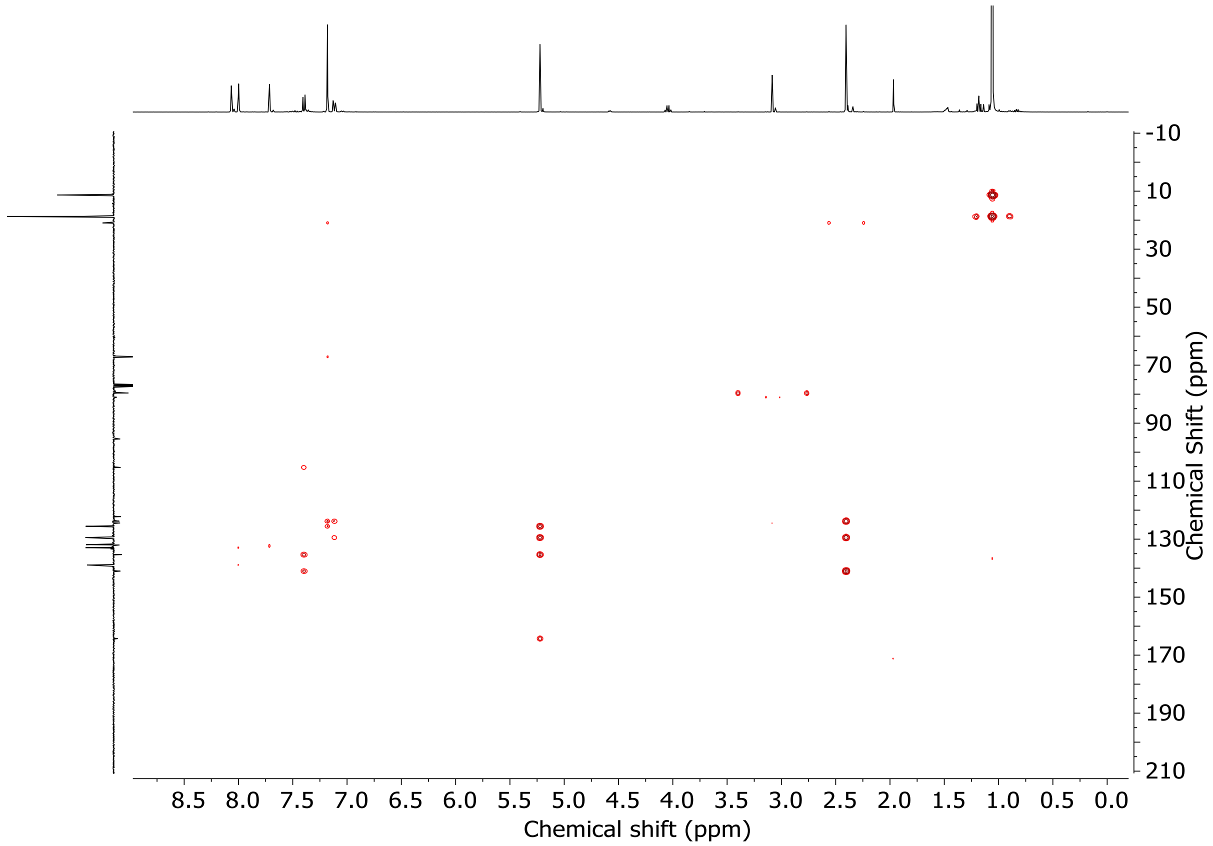

Figure S72: HMBC NMR ($CDCl_3$) of **S11**.

Alkyne precursor (*S*)-S12

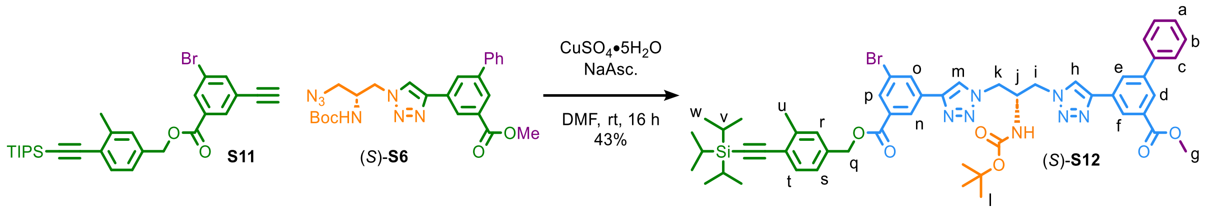

A suspension of (*S*)-**S6** (130 mg, 0.27 mmol), **S11** (138.7 mg, 0.27 mmol), $CuSO_4 \cdot 5\ H_2O$ (4.3 mg, 0.027 mmol) and sodium ascorbate (8.3 mg, 0.041 mmol) in DMF (3 mL) is degassed by bubbling N_2 for 10 min. The solution turns brown, and it is stirred at rt for 16 h. The reaction mixture is poured into a NH_3-EDTA (10 mL) then extracted with EtOAc (3 x 20 mL). The organic phases were washed with a 5% LiCl solution (50 mL), washed with brine (20 mL), dried ($MgSO_4$) and concentrated *in vacuo*. Chromatography (petrol-EtOAc 0→50%) gave (*S*)-**S12** as a white foam (116.0 mg, 43%).

^{1}H NMR (400 MHz, CDCl$_3$) δ: 8.33 (app. s, 1H, H_f), 8.29 (s, 1H, H_n or H_p), 8.27 (app. s, 1H, H_e), 8.21 (app. s, 1H, H_d), 8.19 (s, 1H, H_h) 8,18-8.15 (m, 2H, H_o, H_m), 8.07 (H_n or H_p), 7.68-7.62 (m, 2H, H_b), 7.51-7.43 (m, 3H, H_c, H_t), 7.42-7.36 (m, 1H, H_a) 7.28 (bs, 1H, H_r), 7.14 (s, 1H, H_s) 5.98 (d, *J* = 7.4, 1H, NH), 5.31 (s, 2H, H_q), 4.76-4.44 (m, 5H, H_i, H_j, H_k) 3.96 (s, 3H, H_g), 2.48 (s, 3H, H_u), 1.42 (s, 9H, H_l), 1.13 (m, 21H, H_v, H_w)

^{13}C NMR (101 MHz, CDCl$_3$) δ: 166.6, 164.7, 155.3, 147.1, 145.7, 142.2, 141.0, 139.5, 135.5, 132.7, 132.7, 132.4, 132.3, 132.2, 131.3, 129.6, 128.9, 128.4, 128.1, 127.2, 125.7, 125.4, 125.3, 123.8, 123.1, 122.4, 122.1, 105.3, 95.4, 80.9, 67.1, 52.4, 51.2, 50.1, 50.0, 40.9, 33.9, 33.8, 28.3, 23.9, 20.9, 18.7, 11.3.

HR-ESI-MS (+ve) *m/z* = 1008.3 $[M+Na]^+$ for isotopic pattern see Figure S79

$[\alpha]_D^{23}$-2.3 (c 0.72, $CHCl_3$)

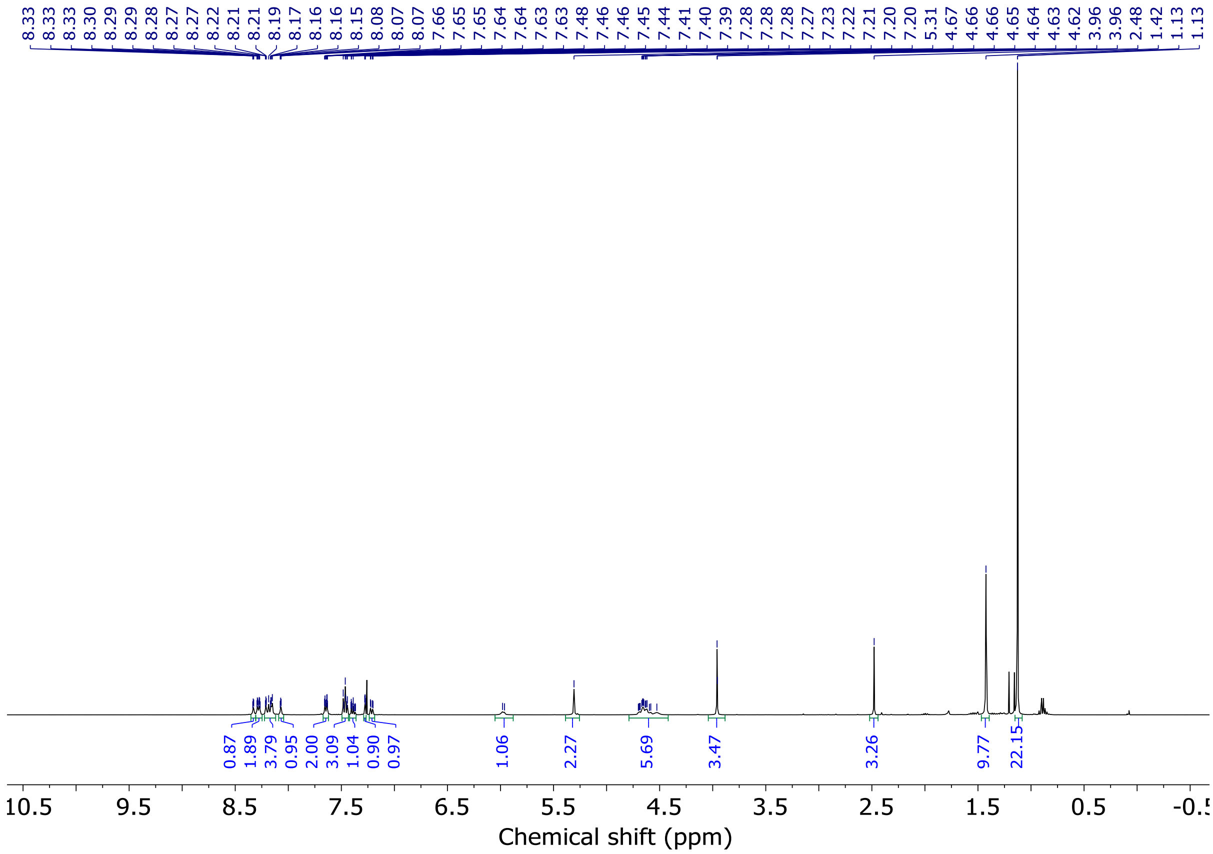

Figure S73: ^{1}H NMR ($CDCl_3$, 400 MHz) of (*S*)-**S12**.

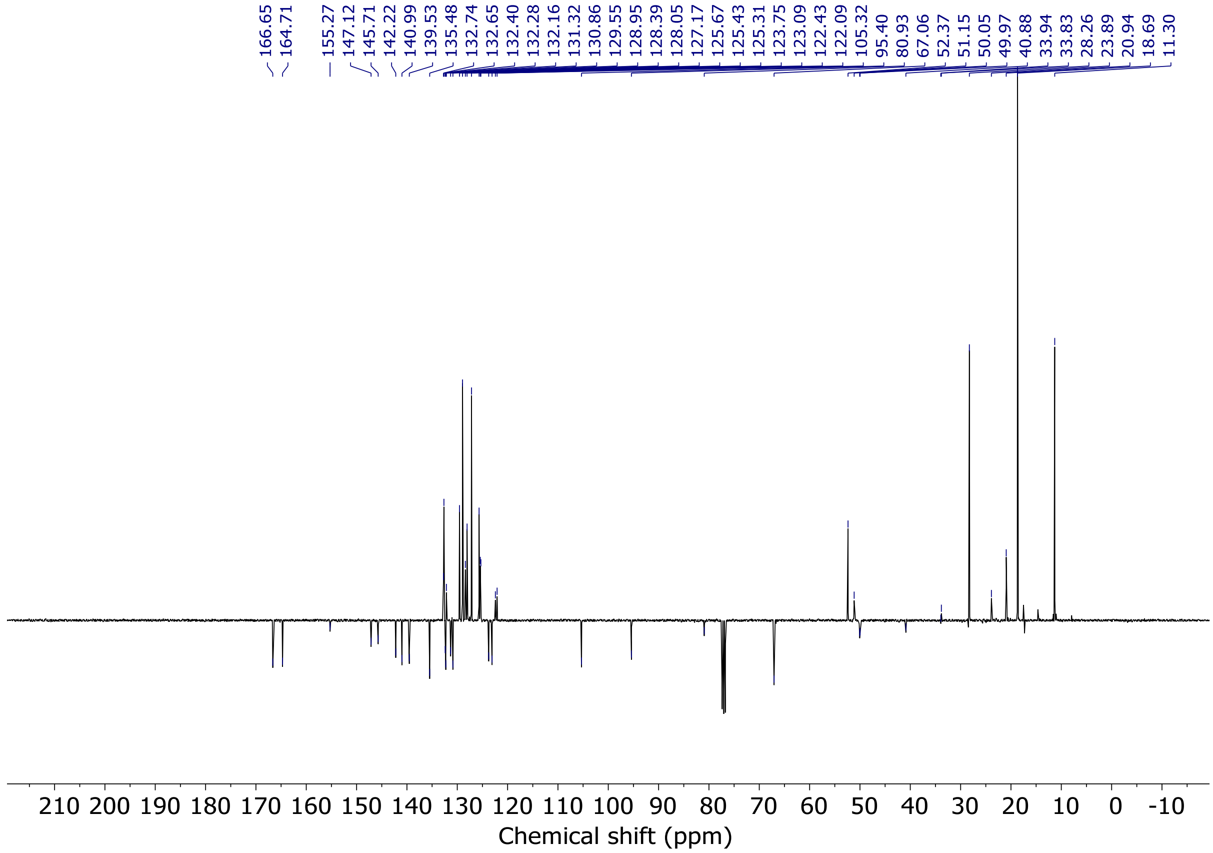

Figure S74: JMOD NMR ($CDCl_3$, 101 MHz) of (*S*)-**S12**.

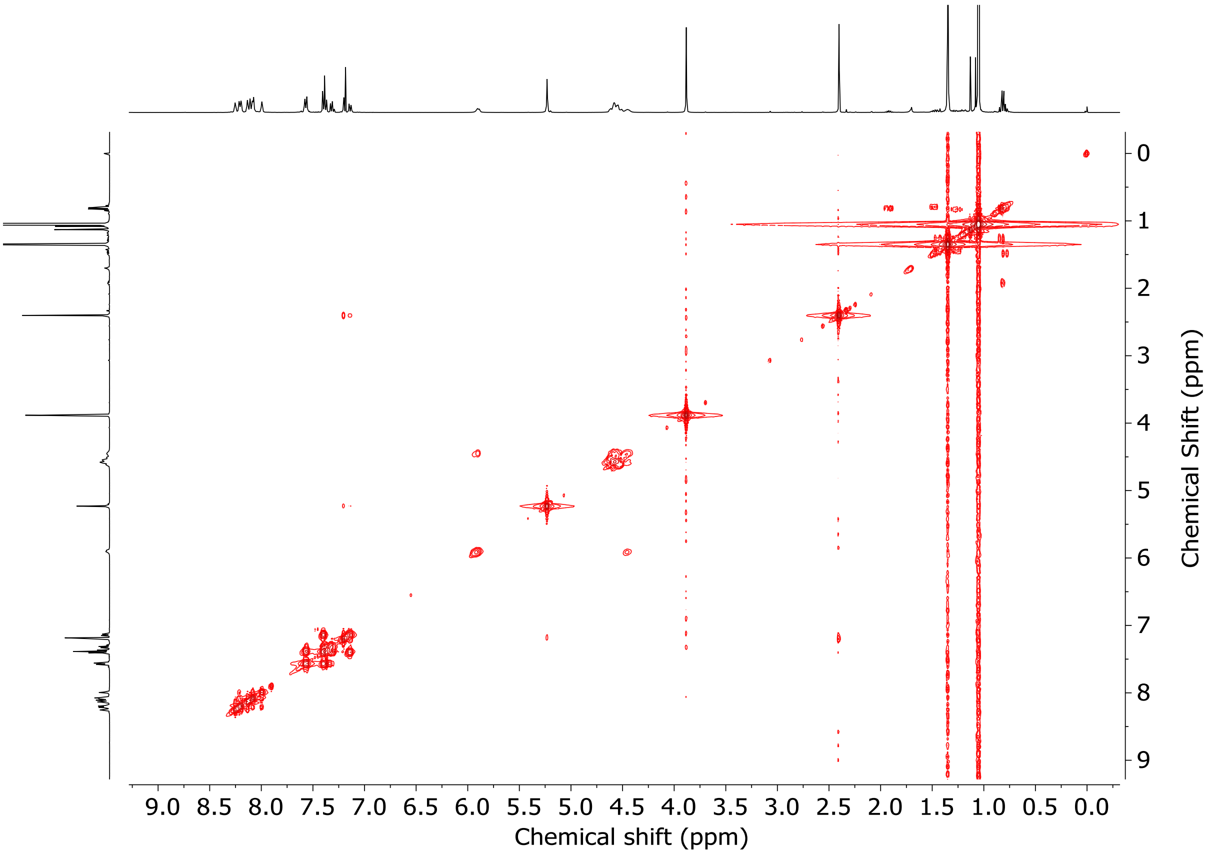

Figure S75: COSY NMR ($CDCl_3$) of (*S*)-**S12**.

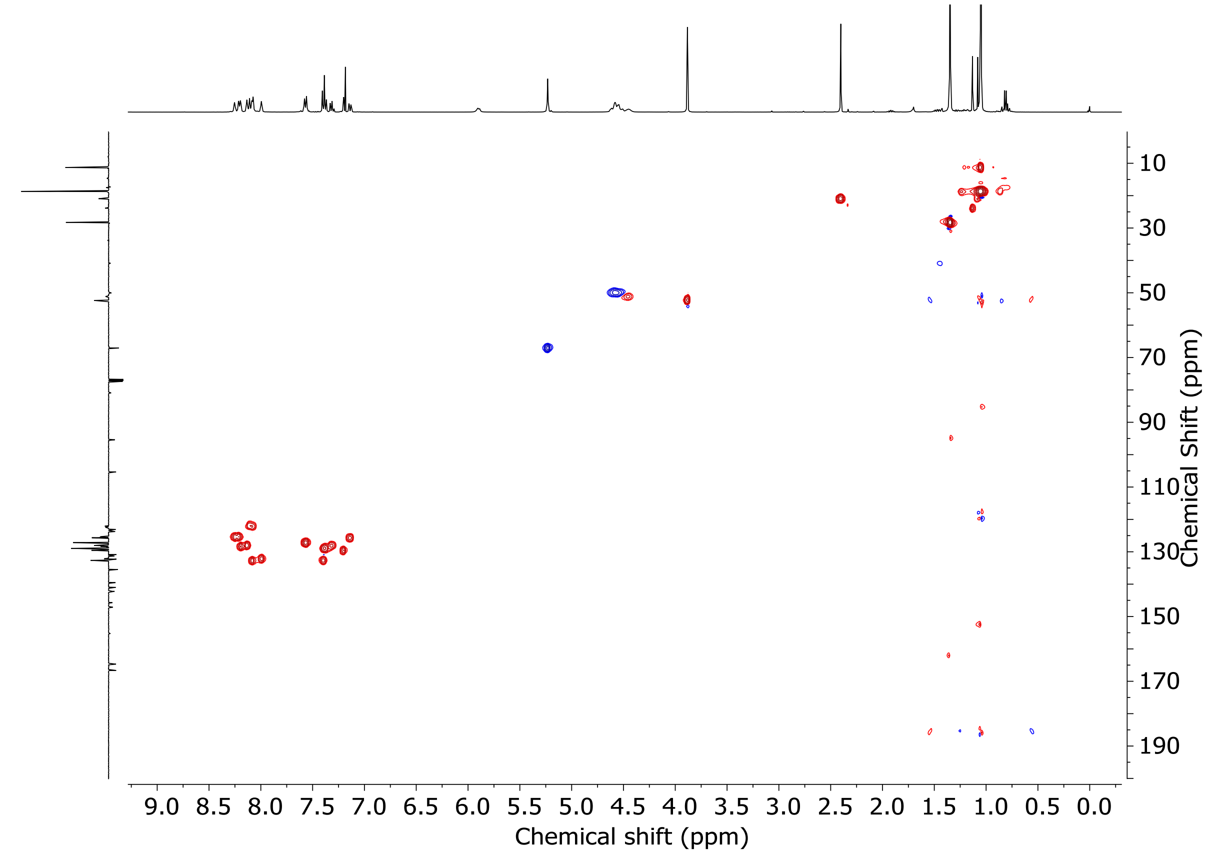

Figure S76: HSQC NMR ($CDCl_3$) of (*S*)-**S12**.

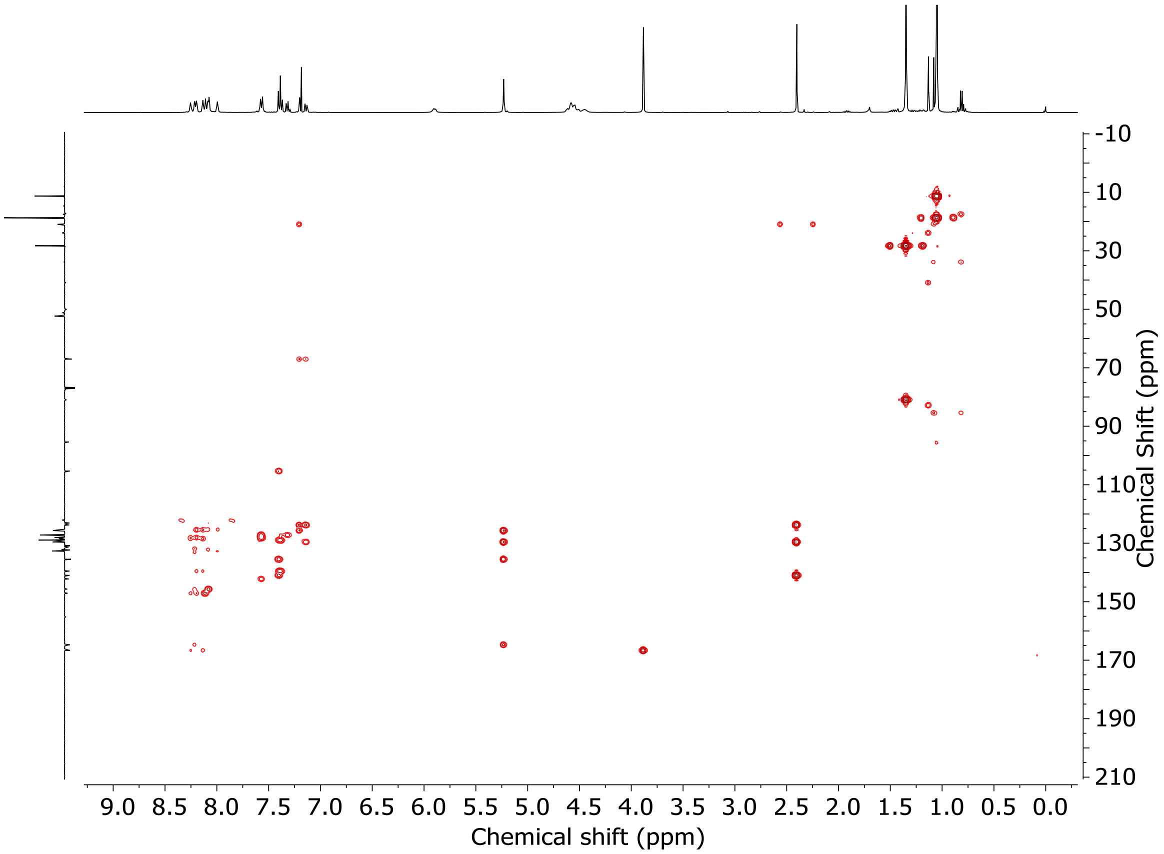

Figure S77: HMBC NMR ($CDCl_3$) of (*S*)-**S12**.

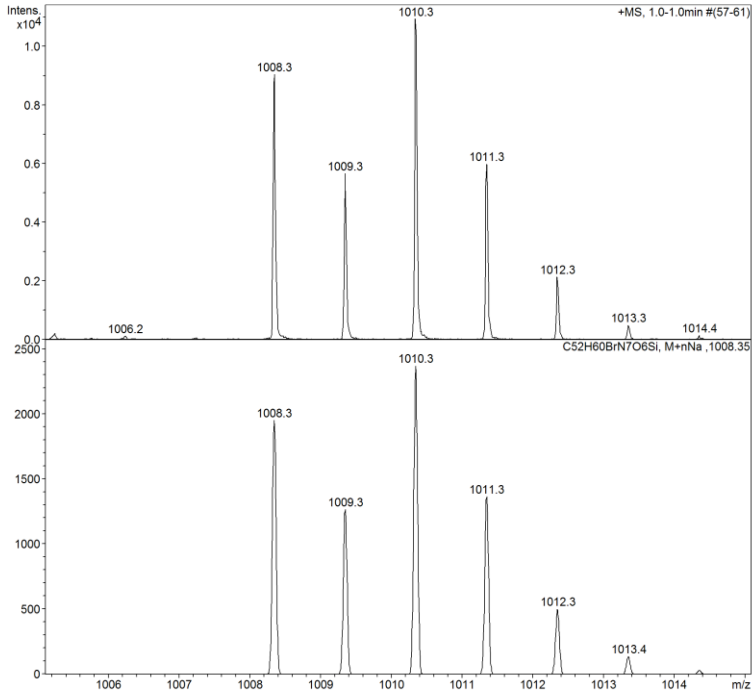

Figure S78: Calculated (top) and observed (bottom) isotopic patterns for (*S*)-**S12**.

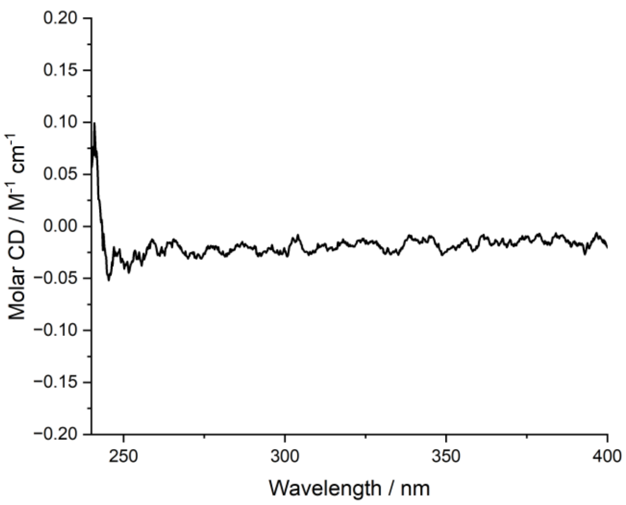

Figure S79: Circular dichroism spectra of (*S*)-**S12** (63.5 µM) at 293 K in $CHCl_3$. No measurable CD response was observed so the $[\alpha]_D$ of (*S*)-**S12** was measured.

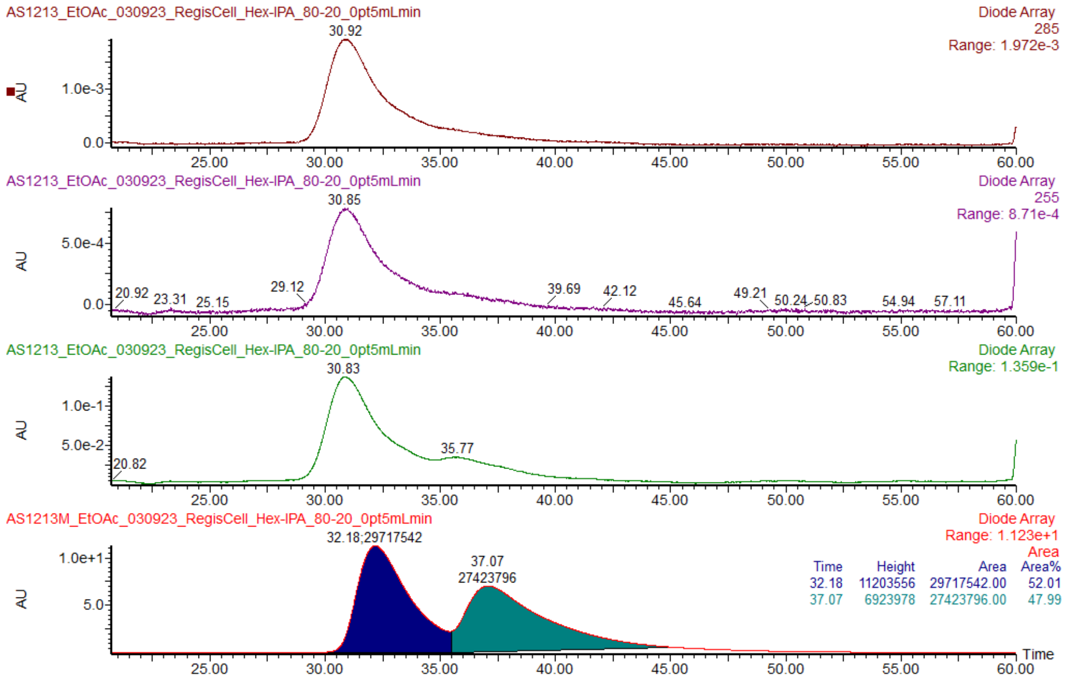

Figure S80: CSP-HPLC of (S)-**S12** (loaded in EtOAc) with 285 and 255 nm traces. RegisCell, *n*-hexane-^{i}PrOH 80 : 20, flowrate 0.5 mLmin^{-1}. The minor signal observed at ~35.77 min is not consistent with the minor enantiomer; varying the wavelength of detection yielded a different relative intensity vs the major enantiomer. This impurity could not be detected by ^{1}H NMR and could not be completely removed from the sample. On this basis, the enantiopurity of (*S*)-**S12** is estimated to be >99% ee. (bottom) *rac*-**S12**, (*S*)-**S12** (32.18 min, 29717542, 52.01%), (*R*)-**S12** (37.07 min, 27423796, 47.99%).

1.8 Alkyne (*S*)-13

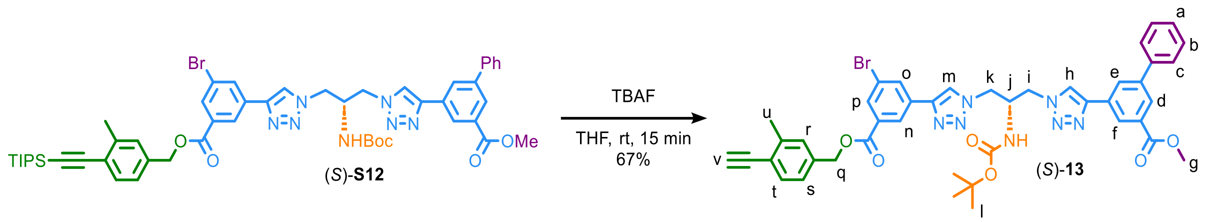

To a solution of (*S*)-**S12** (110 mg, 0.11 mmol) in THF (2 mL) was added a 1M solution of TBAF in THF (0.22 mL). The solution is stirred at rt for 15 min. The reaction mixture was partitioned between H_2O (10 mL) and EtOAc (10 mL), the phases separated, and the organic layer extracted with EtOAc (2 x 10 mL). The collected organic fractions were then washed with brine (10 mL), then dried ($MgSO_4$). The solvent was removed *in vacuo*. Chromatography (petrol-EtOAC 0→50%) gave (*S*)-**13** as a white foam (61.2 mg, 67%).

^{1}H NMR (400 MHz, $CDCl_3$) δ: 8.39 (t, *J* = 1.6, 1H, H_f), 8.36 (t, *J* = 1.5, 1H, H_n or H_p), 8.33 (t, *J* =1.7, 1H, H_e), 8.26 (t, *J* = 2.0, 1H, H_d), 8.23 (t, *J* = 1.6, H, H_n or H_p) 8.18-8.10 (m, 3H, H_h, H_m, H_o), 7.72-7.66 (m, 2H, H_a) ,7.53-7.45 (m, 3H, H_b, H_t) 7.44-7.38 (m, 1H, H_a), 7.29 (app. sept., *J* = 0.6, 1H, H_r), 7.25-7.21 (m, 1H, H_s), 5.83 (d, *J* = 7.7, 1H, NH), 5.34 (s, 2H, H_q), 4.70-4.50 (m, 4H, H_i, H_k), 4.45 (app. quint., *J* = 5.2, 1H, H_j), 3.98 (s, 3H, H_g), 3.29 (s, 1H, H_v), 2.47 (s, 3H, H_u), 1.45 (s, 9H, H_l).

^{13}C NMR (101 MHz, $CDCl_3$) δ: 166.7, 164.7, 155.2, 147.3, 145.8, 142.3, 141.3, 139.6, 132.9, 132.9, 132.5, 132.3, 132.3, 131.5, 130.8, 129.5, 129.0, 128.5, 128.2, 128.1, 127.2, 125.6, 125.5, 125.4, 123.2, 122.4, 122.0, 82.1, 81.5, 81.1, 66.9, 52.4, 51.2, 49.9, 49.8, 28.3, 23.8, 20.5

HR-ESI-MS (+ve) *m/z* = 830.2290 $[M+H]^+$ (calc. 830.2296 *m/z* for $C_{43}H_{40}BrN_7O_6$);

$[a]_D^{23}$-8.5 (c 0.80, $CHCl_3$);

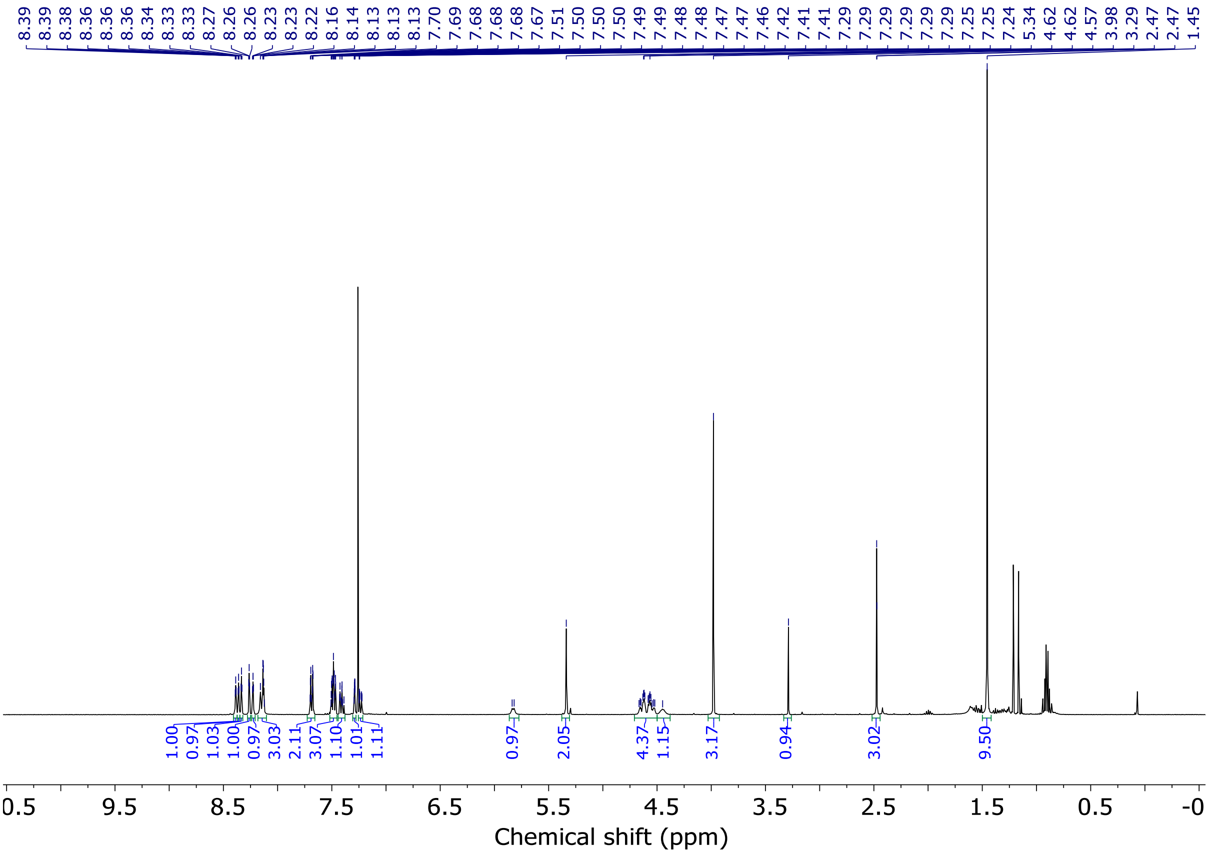

Figure S81: ^{1}H NMR ($CDCl_3$, 400 MHz) of (*S*)-**13**.

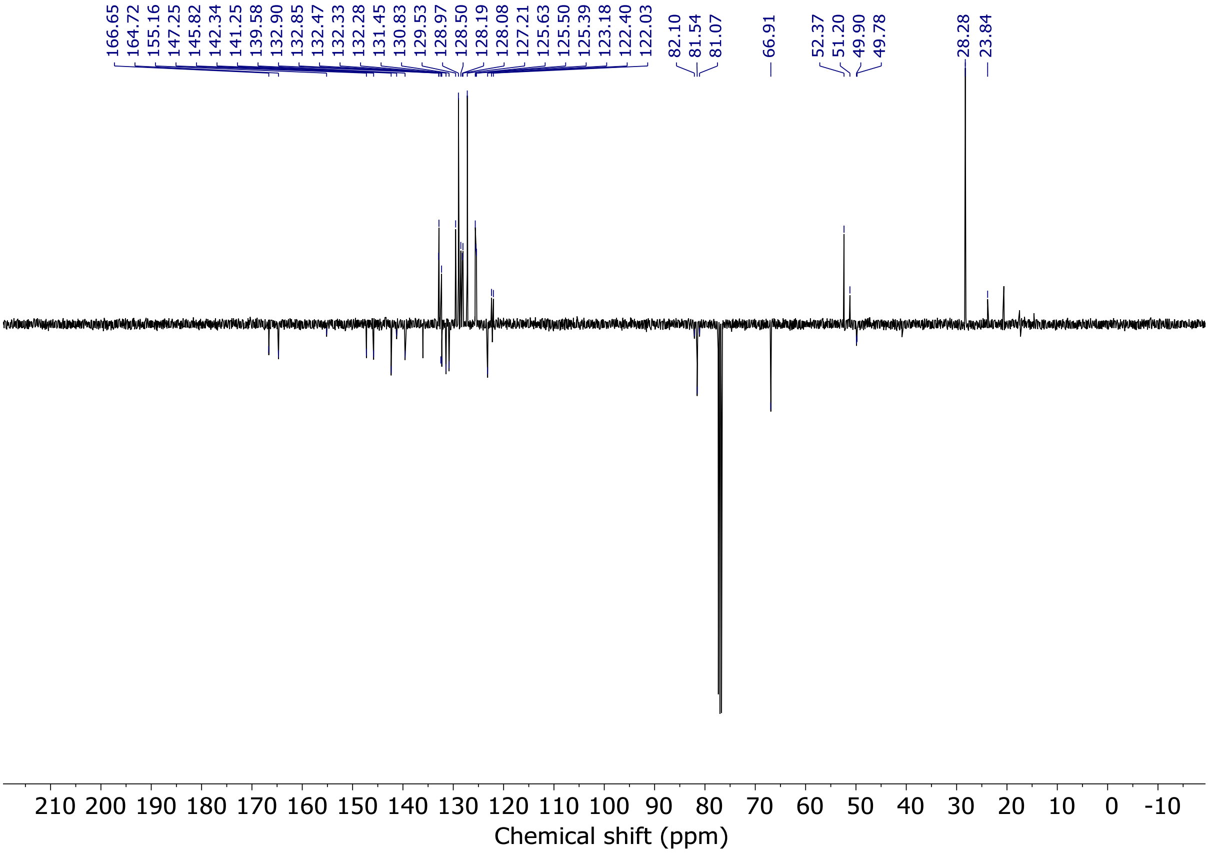

Figure S82: JMOD NMR ($CDCl_3$, 101 MHz) of (*S*)-**13**.

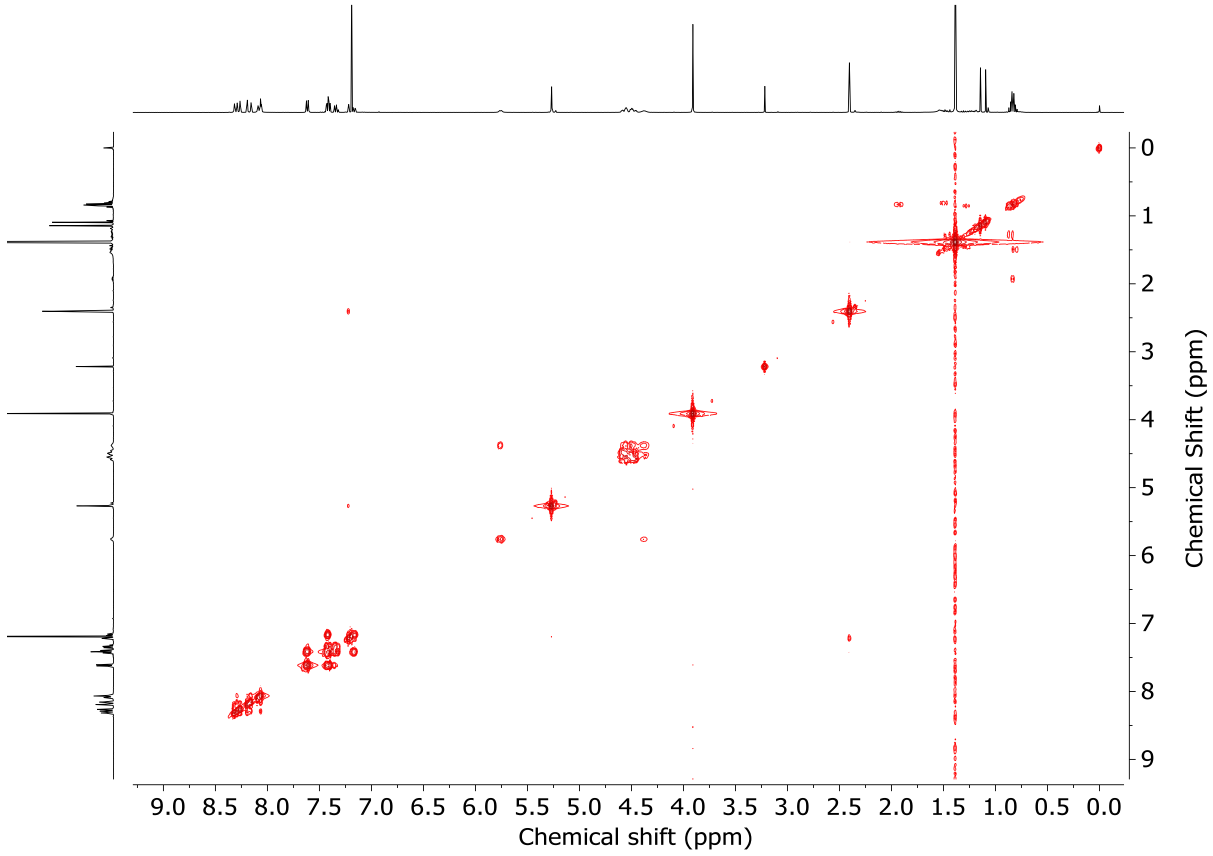

Figure S83: COSY NMR ($CDCl_3$) of (*S*)-**13**.

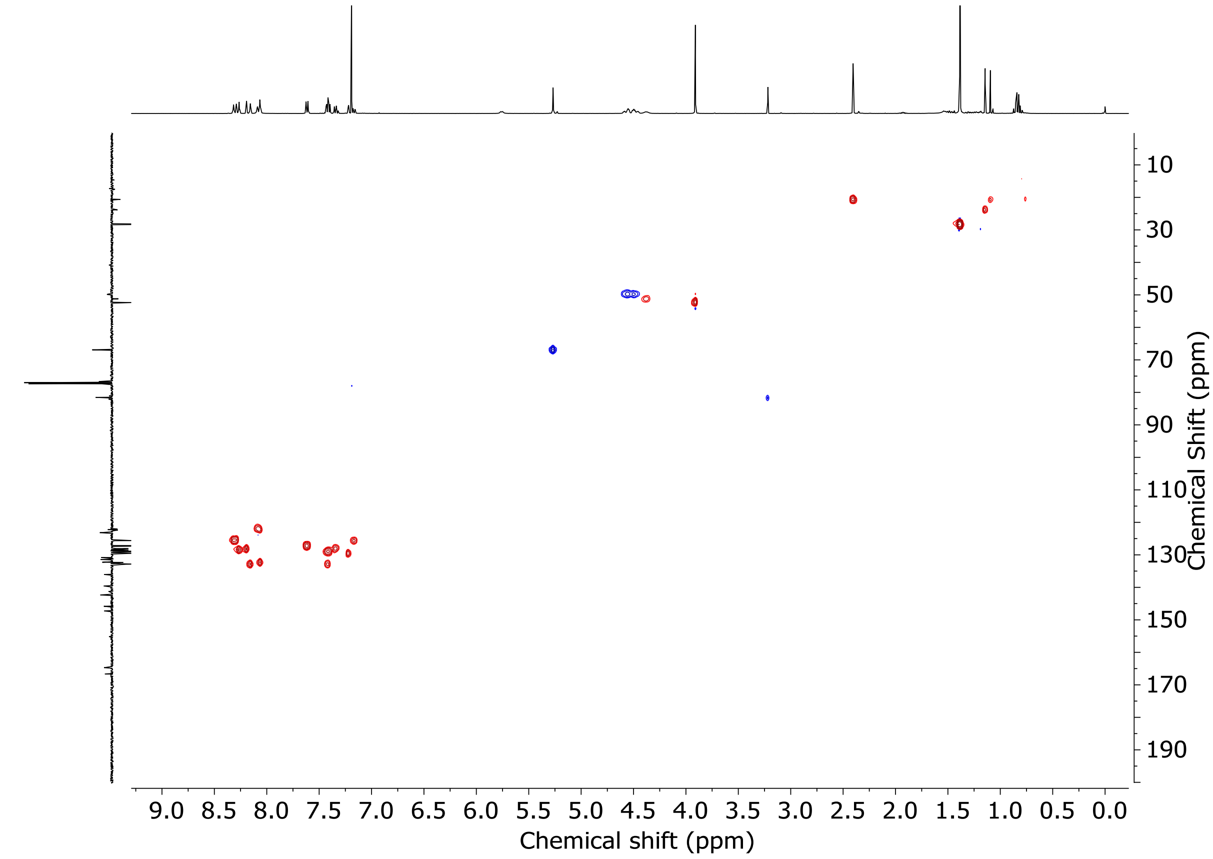

Figure S84: HSQC NMR ($CDCl_3$) of (*S*)-**13**.

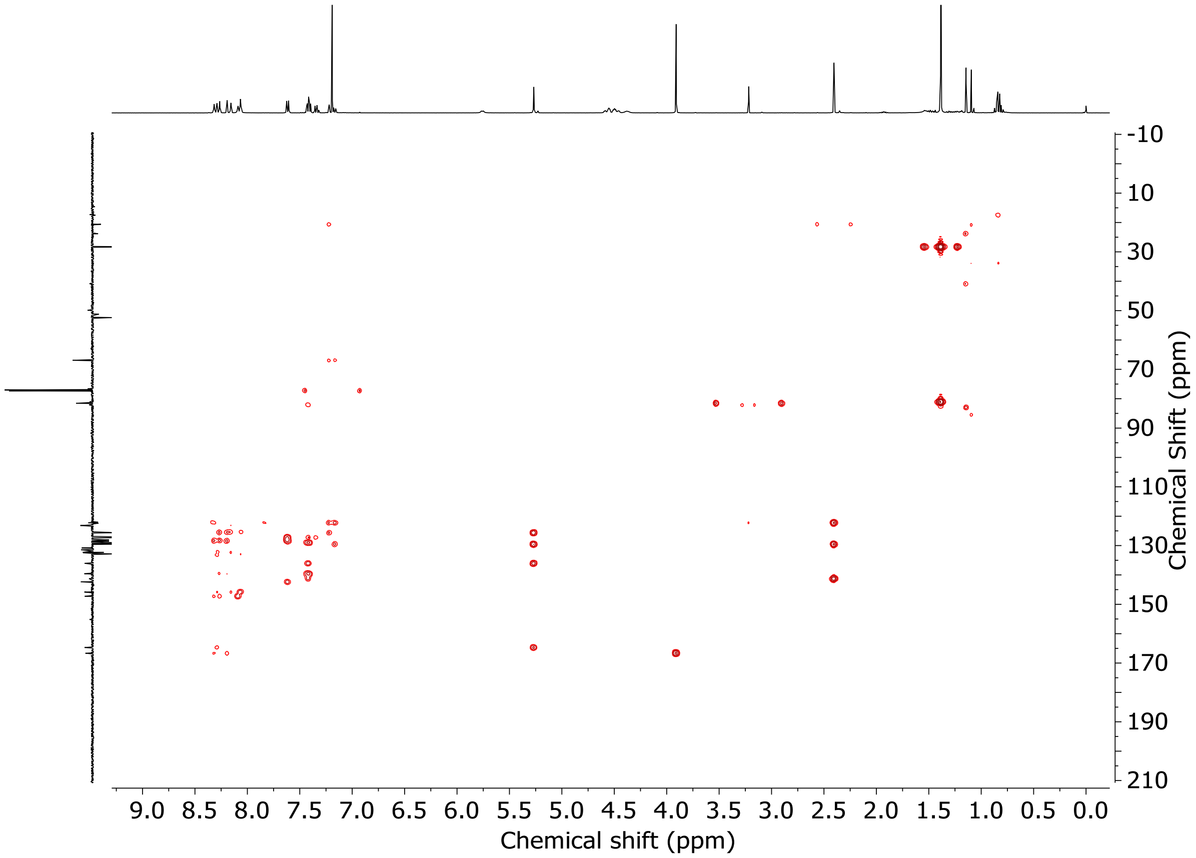

Figure S85: HMBC NMR ($CDCl_3$) of (*S*)-**13**.

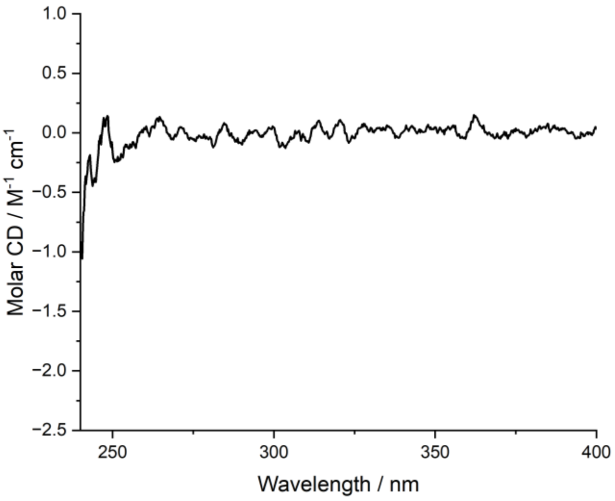

Figure S86: Circular dichroism spectra of (*S*)-**13** (63.5 µM) at 293 K in $CHCl_3$. No measurable CD response was observed so the $[\boldsymbol{\alpha}]_D$ of (*S*)-**13** was measured.

1.9 Rotaxane (E_m,S_{co-c})-10

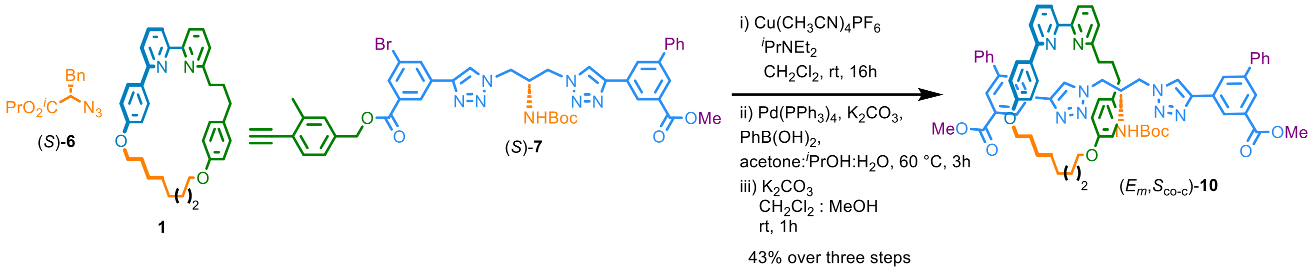

A dry sealed vessel was charged with (*S*)-**7** (50 mg, 60.2 µmol), (*S*)-**6** (14 mg, 60.0 µmol), **1** (27 mg, 55.0 µmol), $[Cu(MeCN)_4]PF_6$ (20.3 mg, 54.5 µmol), $^iPr_2NEt$ (39.2 µL, 0.21 mmol), and CH_2Cl_2 (5 mL). The reaction mixture was stirred at rt for 16 h. The solution was diluted with CH_2Cl_2 (5 mL), then EDTA-NH_3 (5 mL) was added. The solution was vigorously stirred until complete decolouration. The aqueous and organic phases were separated, and the aqueous phase was then extracted with CH_2Cl_2 (3 x 10 mL). The combined organic extracts were washed with brine (10 mL), dried ($MgSO_4$) and concentrated *in vacuo* to give a sample containing a mixture of diastereomers (97 : 3 *dr*, Figure S88). The residue was used in the next step without further purification. The crude reaction mixture, $PhB(OH)_2$ (25.5 mg, 0.2 mmol), K_2CO_3 (29 mg, 0.2 mmol), and $Pd(PPh_3)_4$ (6 mg, 51.9 µmol) were dissolved in solvents mixture acetone:$^iPrOH:H_2O$ (2:1:1, 4 mL) (degassed) and stir for 3 h at 60 °C. After colling down to room temperature, the solution was diluted with CH_2Cl_2 (5 mL). The aqueous and organic phases were separated, and the aqueous phase was then extracted with CH_2Cl_2 (3 x 5 mL). The combined organic extracts were washed with brine (5 mL), dried over $MgSO_4$ and concentrated in vacuo and used without further purification. Then dissolved in MeOH:CH_2Cl_2 (1:1, 10 mL) and K_2CO_3 (30 mg, 0.2 mmol) was added and stir for 1h. Solvents were removed, and the crude was purified via silica-gel chromatography (petrol : EtOAc 7:4, 0→40%), gave (E_m,S_{co-c})-**10** as a colourless oil (27.1 mg, 43% over three steps, 92% *de*).

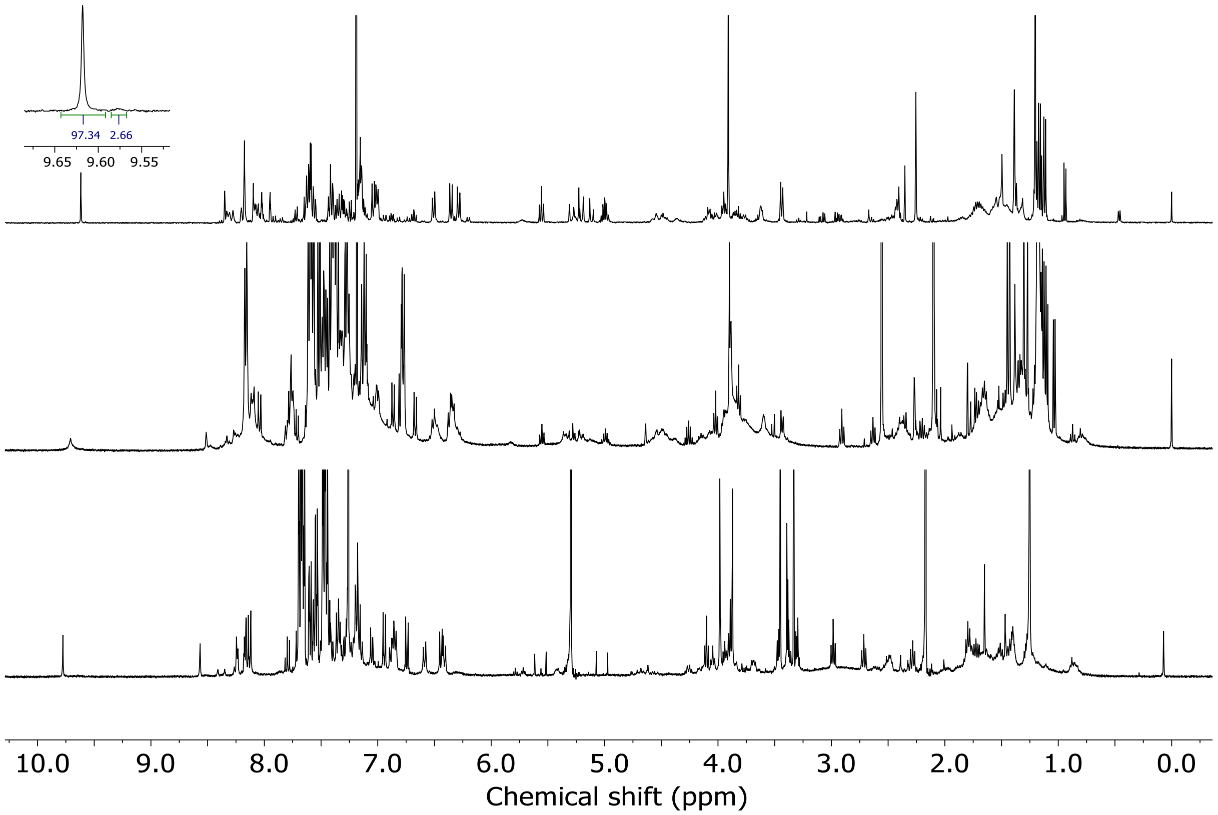

Figure S87: ^{1}H NMR ($CDCl_3$, 400 MHz) of steps towards (E_m,S_{co-c})-**10** prior to chromatography; first step (top), second step (middle) and third step (bottom).

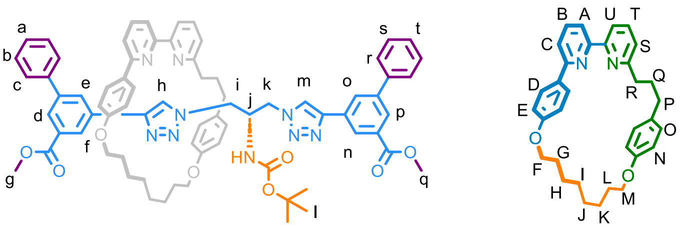

^{1}H NMR (500 MHz, CDCl₃) δ: δ: 9.77 (s, 1H, H_h), 8.57 (t, *J* = 1.6, 1H, H_d), 8.25 (t, *J* = 1.6, 1H, H_n), 8.24 (t, *J* = 1.7, 1.1H, H_p), 8.18 (t, *J* = 1.7, 1H, H_o), 8.16 (t, *J* = 1.6 Hz, 1H, H_f), 7.69-7.66 (m, 4H, H_B, H_m H_r or H_s), 7.59 (t, *J* = 7.9, 2H, H_e ,H_T), 7.51-7.41 (m, 6H, H_A, H_C, H_r or H_s, H_t, H_U), 7.35-7.33 (m, 2H, H_b or H_c), 7.29-7.25 (m, 3H, H_a, H_b or H_c, superimposed with $CDCl_3$), 7.16 (t, *J* = 8.7 Hz, 2H, H_D), 7.12 (d, *J* = 7.8 Hz, 1H, H_S), 6.59 (d, *J* = 8.5, 2H, H_O), 6.44 (d, *J* = 8.9, 2H, H_E), 6.41 (d, *J* = 8.4 Hz, 2H, H_N), 5.41 (d, *J* = 6.1, 1H, NH), 4.28-4.10 (m, 3H, H_k, H_M), 4.08-4.00 (m, 5H, H_F, H_q), 3.99-3.79 (m, 6H, H_g, H_i, H_j, $H_{M`}$), 3.69 (m, 1H, $H_{i`}$), 2.61 (td, *J* = 13.7, 4.4, 1H, H_R), 2.57-2.44 (m, 3H, H_P, $H_{R`}$), 2.03-1.48 (m, 12H, H_G, H_H, H_I, H_J, H_K, H_L, H_Q, superimposed with H_2O), 1.26 (s, 9H, H_l).

^{13}C NMR (126 MHz, CDCl₃) δ: 167.1, 166.7, 162.9, 159.2, 158.8, 157.7, 157.01, 157.0, 146.4, 146.0, 142.1, 141.2, 139.7, 139.7, 137.3, 137.0, 132.5, 132.1, 131.5, 131.3, 131.2, 130.9, 129.0, 128.9, 128.6, 128.4, 128.0, 127.7, 127.4, 127.2, 126.8, 125.5, 125.3, 122.4, 121.2, 120.4, 120.3, 120.2, 114.6, 113.8, 67.7, 66.5, 52.3, 52.1, 50.2, 49.9, 37.2, 34.9, 31.9, 29.7, 29.2, 28.5, 28.5, 28.4, 28.1, 25.8, 25.5.

HR-ESI-MS (+ve) *m/z* = 1206.6 $[M+H]^+$ for isotopic pattern see Figure S94

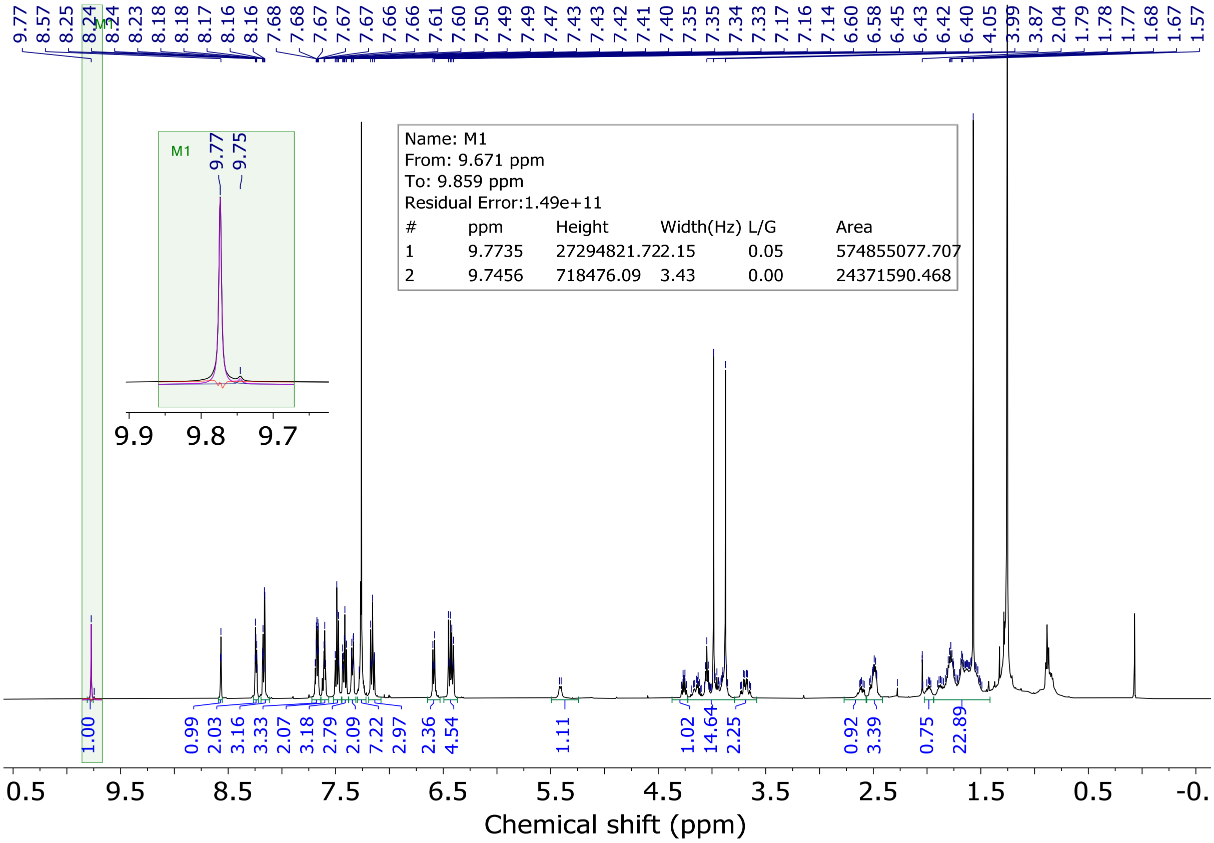

Figure S88: ^{1}H NMR ($CDCl_3$, 500 MHz) of (E_m,$S_{co\text{-}c}$)-**10** (96 : 4 *dr*).

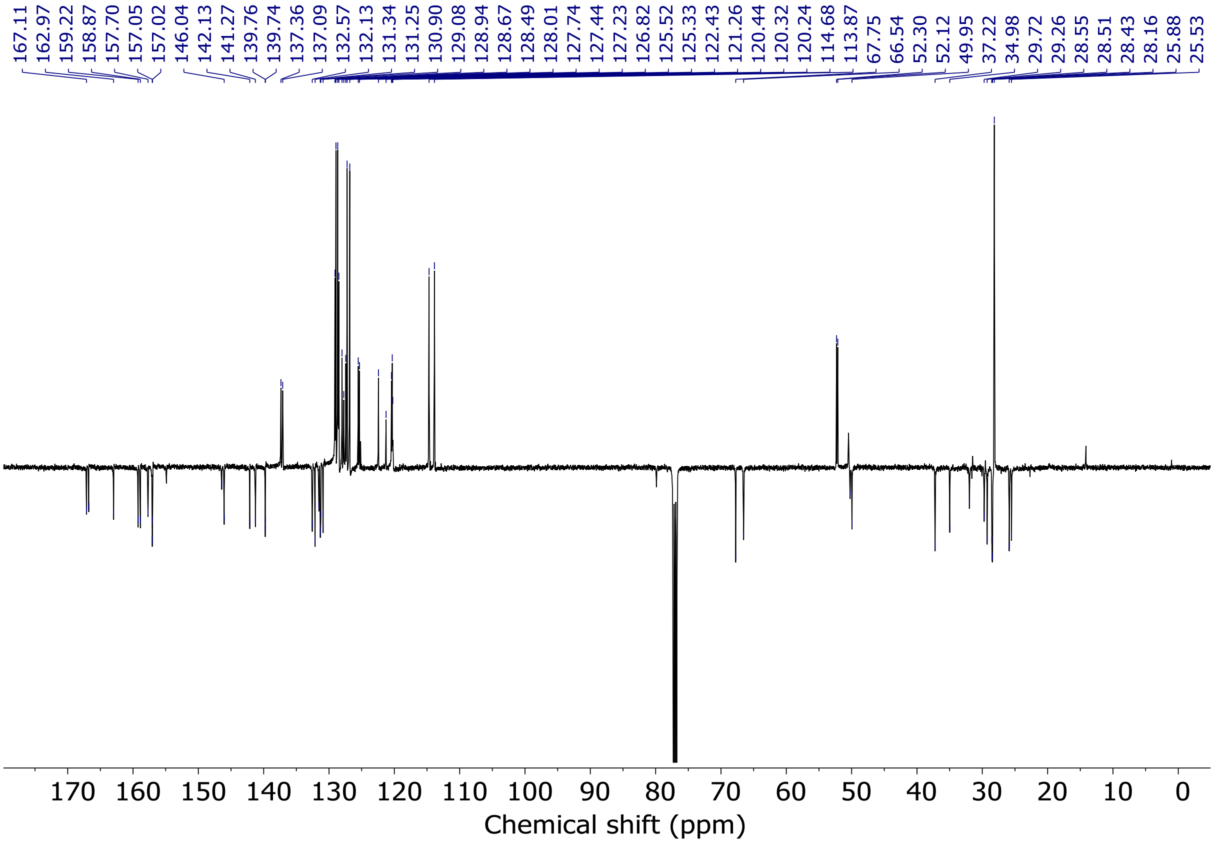

Figure S89: JMOD NMR ($CDCl_3$, 126 MHz) of (E_m,$S_{co\text{-}c}$)-**10** (96 : 4 *dr*).

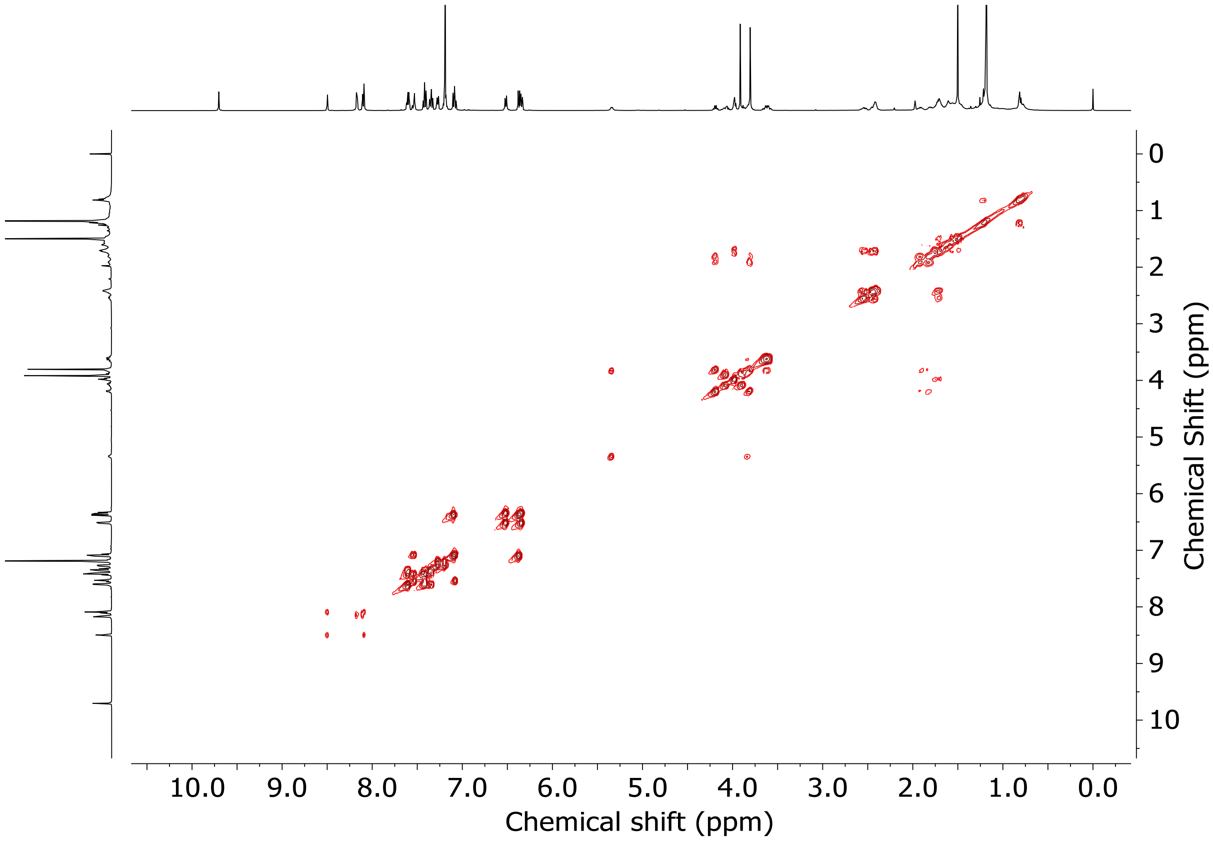

Figure S90: COSY NMR ($CDCl_3$) of (E_m,$S_{co\text{-}c}$)-**10** (96 : 4 *dr*).

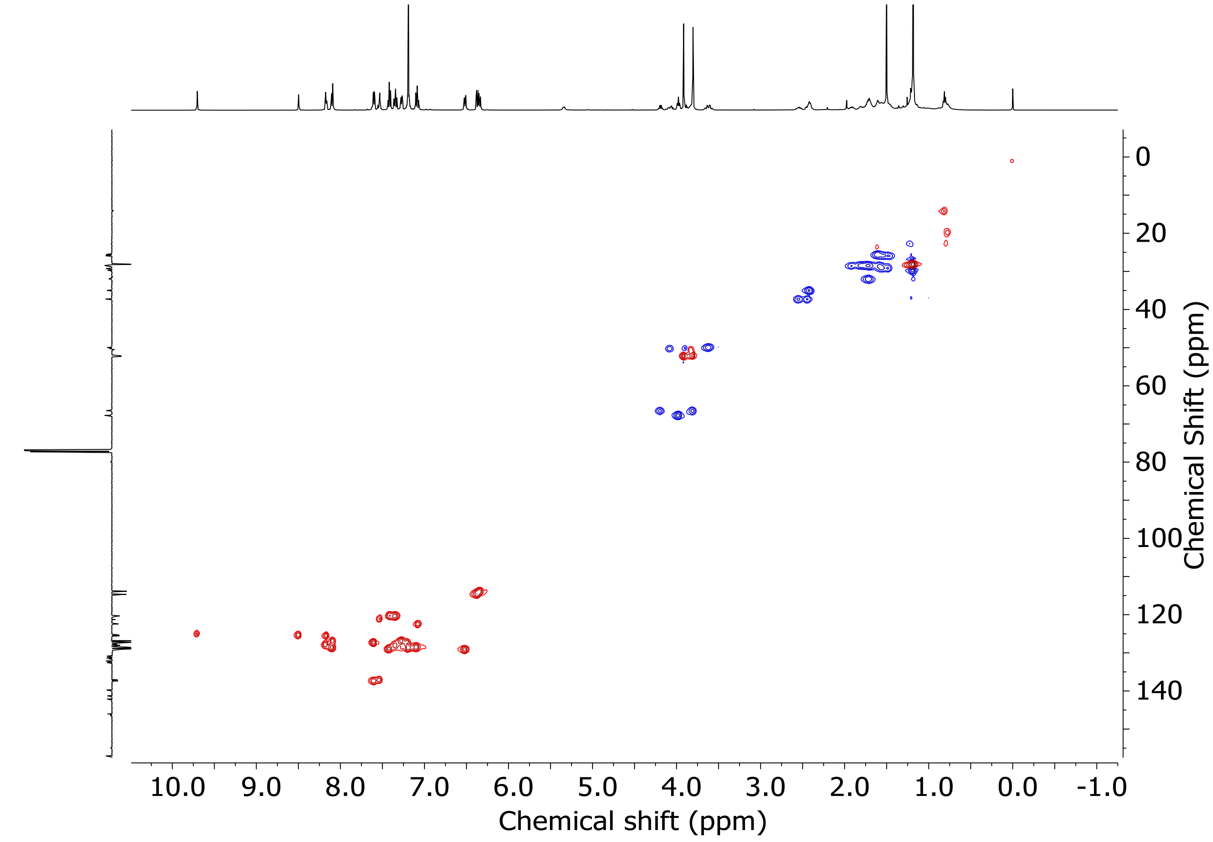

Figure S91: HSQC NMR ($CDCl_3$) of (E_m,$S_{co\text{-}c}$)-**10** (96 : 4 *dr*).

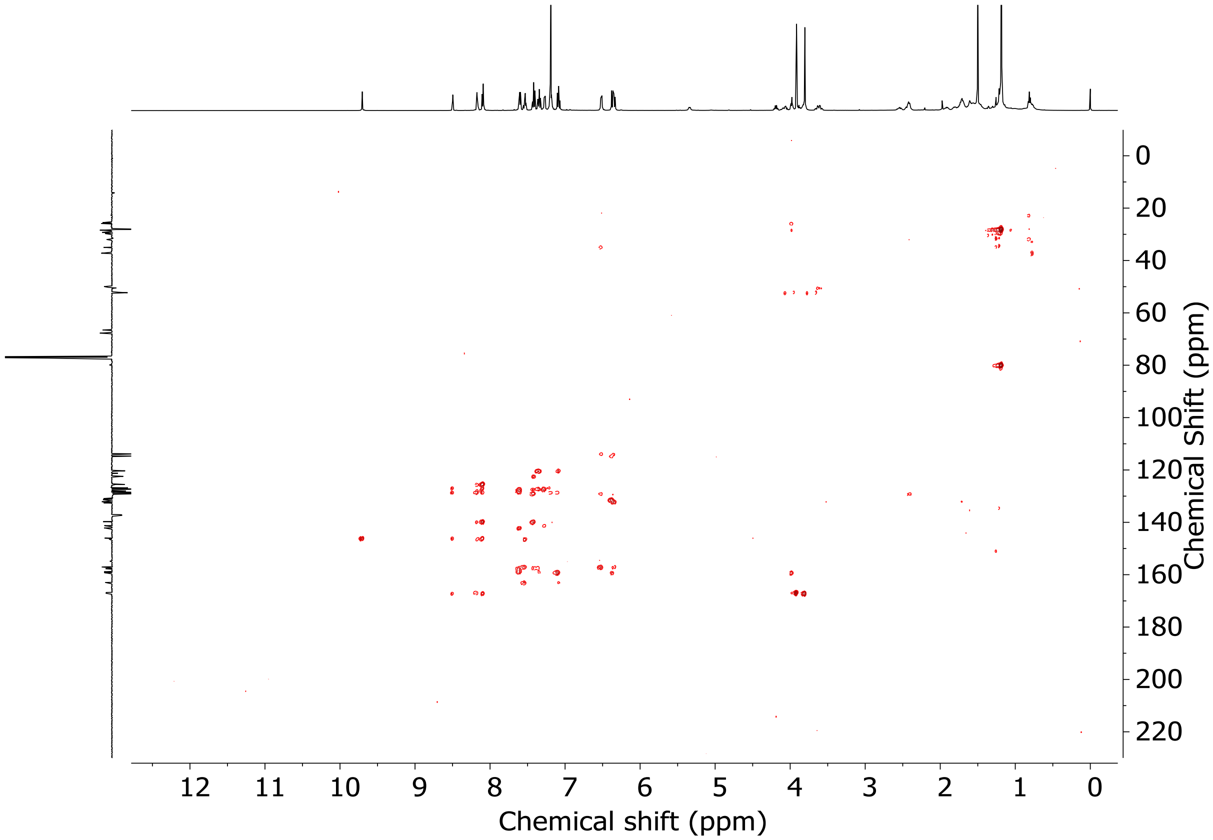

Figure S92: HMBC NMR ($CDCl_3$) of (E_m,$S_{co\text{-}c}$)-**10** (96 : 4 *dr*).

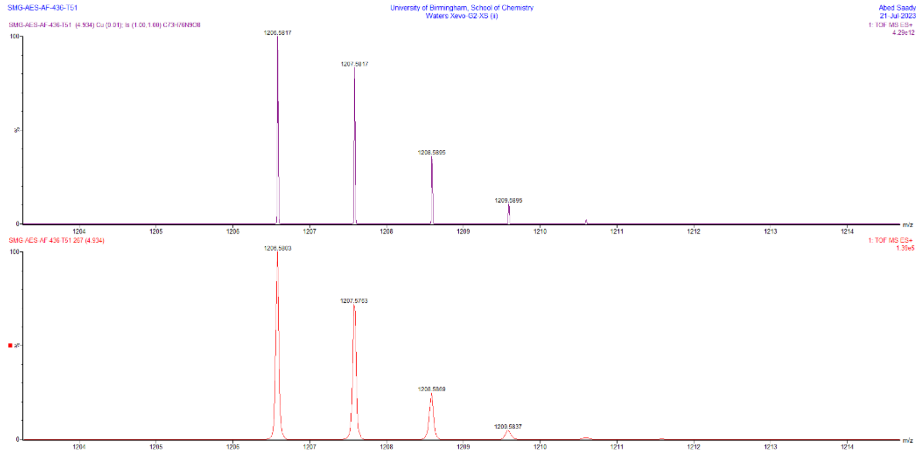

Figure S93: Calculated (top) and observed (bottom) isotopic patterns for (E_m,$S_{co\text{-}c}$)-**10**.

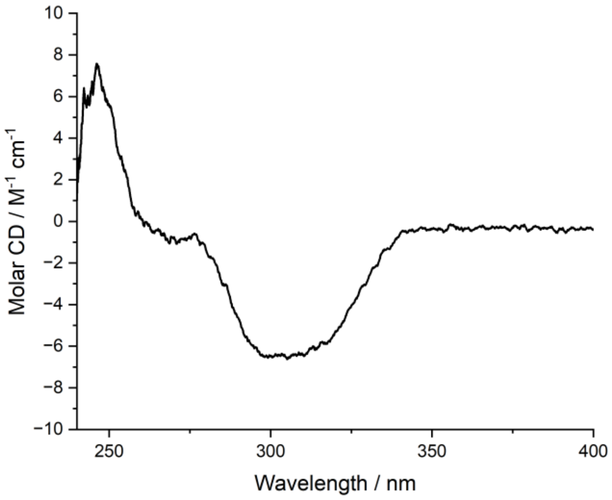

Figure S94: Circular dichroism spectra of (E_m,$S_{co\text{-}c}$)-**10** (63.5 µM) at 293 K in $CHCl_3$

1.10 Rotaxane (E_m)-11

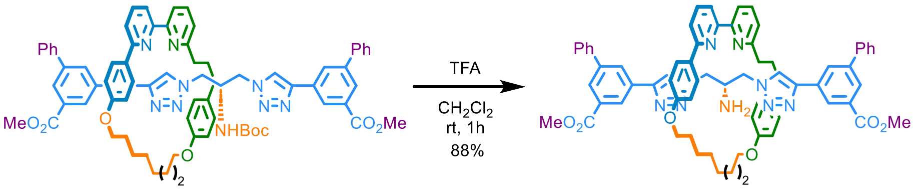

(E_m,$S_{co\text{-}c}$)-**10** (92% *de*) (E_m)-**11** (94% *de*)

A vessel was charged with rotaxane (E_m,$S_{co\text{-}c}$)-**10** (15.0 mg, 0.012 mmol, 92% *de*), TFA (21.4 µL, 0.18 mmol), and CH_2Cl_2 (1 mL). The reaction mixture was stirred at rt for 1 h. CH_2Cl_2 (10 mL) was added, washed with sat. $NaHCO_3$ solution (10 mL), brine (10 mL), and the combined organic extracts were dried ($MgSO_4$) and concentrated *in vacuo*. The residue was purified by column chromatography (*n*-hexane-EtOAc 0→40%) gave rotaxane (E_m)-**11** as a white foam (12.1 mg, 88%, 94% *de*).

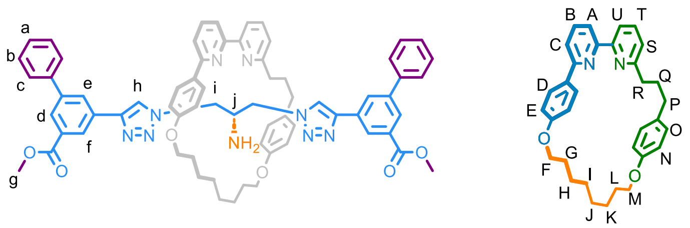

^{1}H NMR (500 MHz, $CDCl_3$) δ: δ: 8.73 (s, 2H, H_h), 8.40 (t, J = 1.7, 2H, H_f), 8.20 (t, J = 1.7, 2H, H_d), 8.14 (t, J = 1.7, 2H, H_e), 7.62 (app t, J = 7.8, 1H, H_B),7.56-7.50 (m, 5H, H_b or H_c, H_T), 7.42-7.33 (m, 9H, H_a, H_b or H_c, H_A, H_C, H_U), 7.25 (d, J = 8.6, 2H, H_E), 7.07 (d, J = 7.8, 1H, H_S), 6.64 (d, J = 8.4, 2H, H_N), 6.55 (d, J = 8.6, 2H, H_D), 6.52 (d, J = 8.4, 2H, H_O), 4.14-4.05 (m, 5H, H_F, H_M), 3.93 (s, 6H, H_g), 3.87 (dd, J = 14.4, 4.2, 2H, H_i), 3.42-3.35 (m, 3H, $H_{i`}$, NH), 2.86 (t, J = 5.3, 2H, H_R), 2.64 (app. sept. J = 3.5, 1H, H_j), 2.52-2.38 (m, 2H, H_P) 1.91 (t, J = 5.4, 2H, H_L), 1.80-1.43 (m, 12H, H_G, H_H, H_I, H_J, H_K, H_Q, superimposed with H_2O)

^{13}C NMR (125 MHz, CDCl$_3$) δ: 167.3, 163.3, 159.6, 159.4, 158.1, 157.6, 157.4, 146.6, 142.3, 140.2, 137.9, 137.5, 132.9, 132.5, 132.1, 131.7, 129.8, 129.3, 128.8, 128.2, 127.8, 127.5, 125.6, 125.2, 124.9, 124.8, 123.9, 123.3, 122.9, 121.0, 120.9, 120.7, 119.6, 119.5, 119.3, 115.4, 114.5, 67.9, 67.0, 65.1, 54.5, 52.7, 51.0, 37.6, 37.0, 35.4, 33.1, 32.4, 32.0, 30.8, 30.6, 30.1, 28.8, 26.4, 26.1.

HR-ESI-MS (+ve) *m/z* = 1106.5 [M+H]$^+$ for isotopic pattern see Figure S101

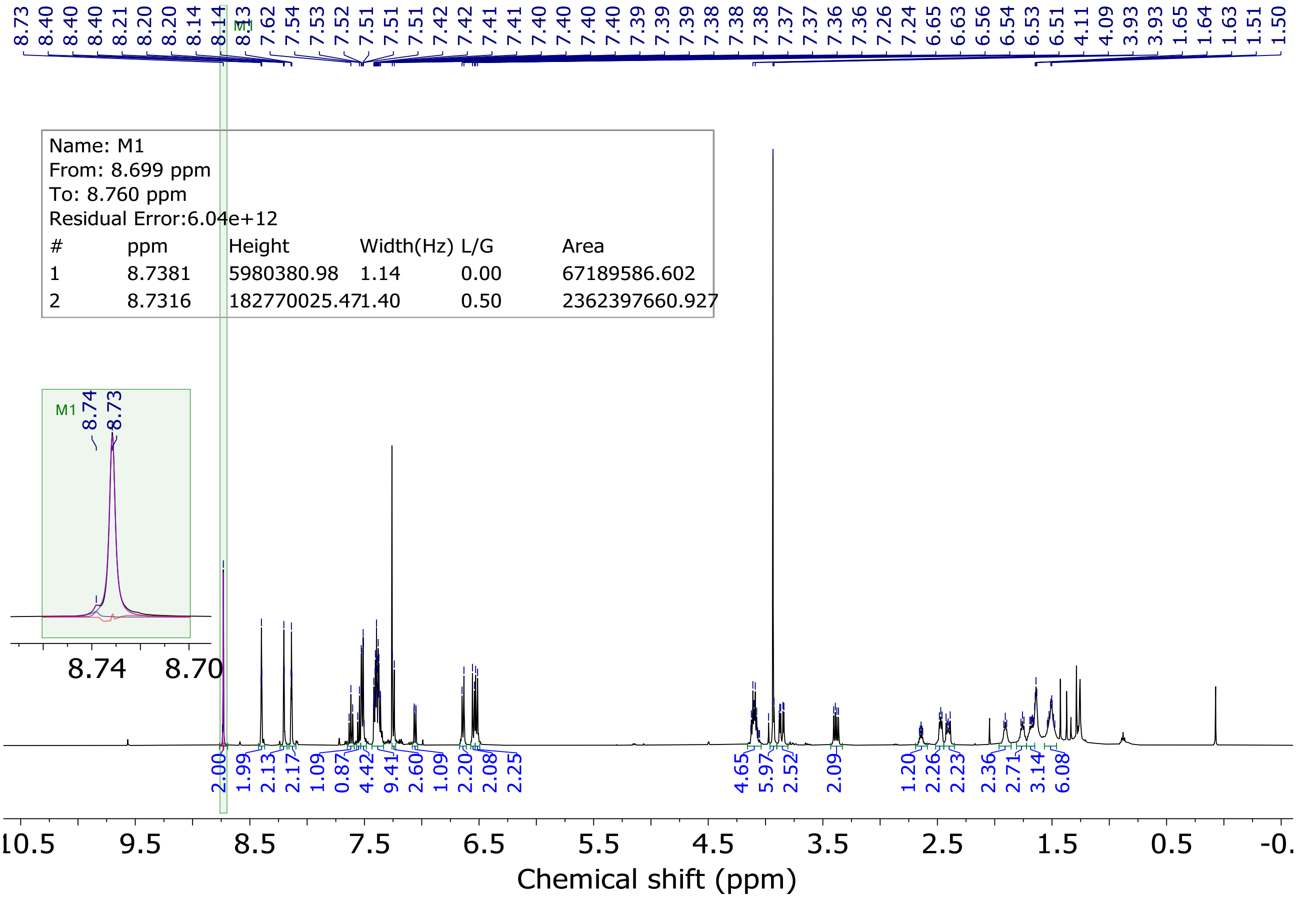

Figure S95: ^{1}H NMR (CDCl$_3$, 500 MHz) of (E_m)-**11** (97: 3 *dr*).

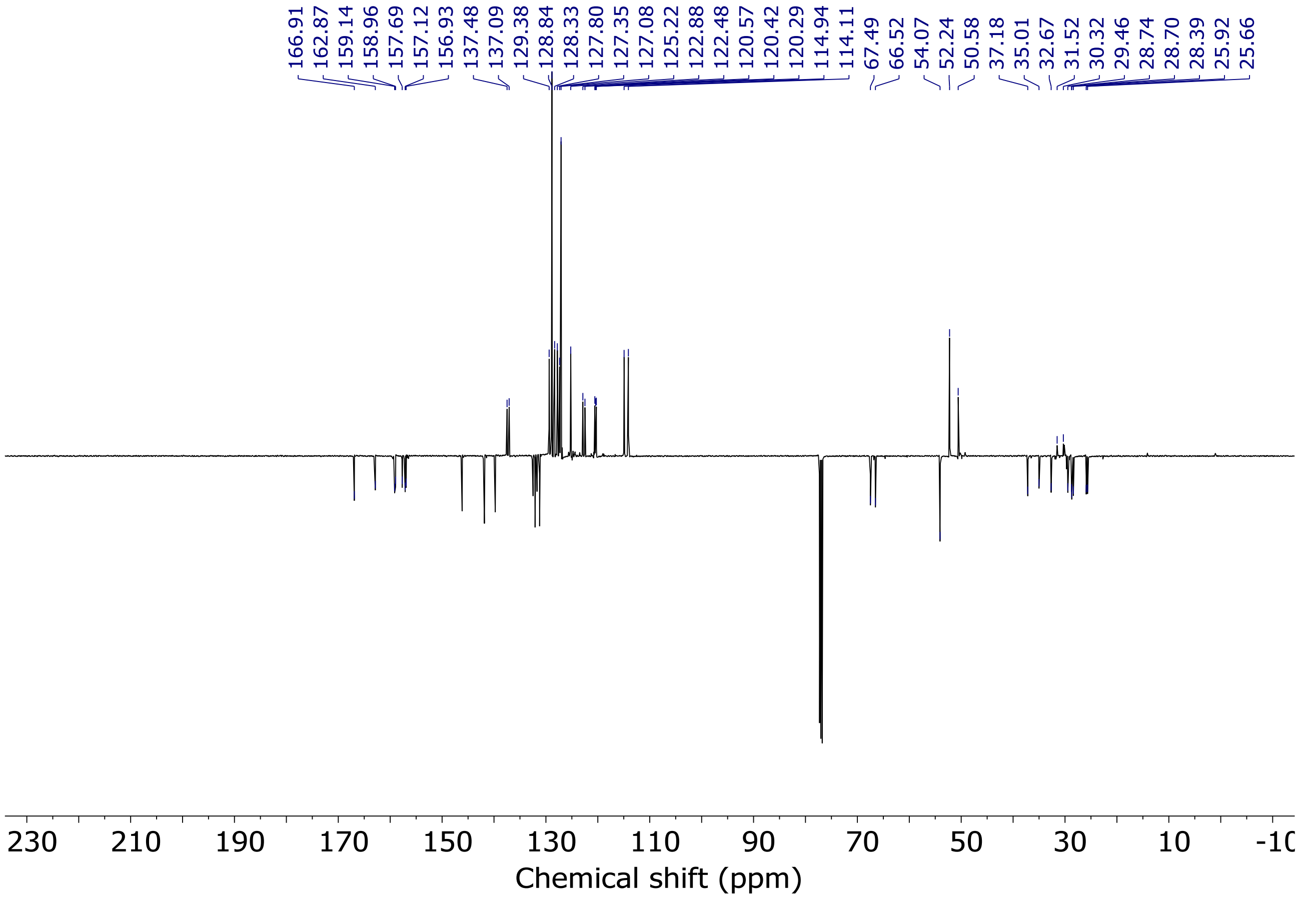

Figure S96: JMOD NMR ($CDCl_3$, 126 MHz) of (E_m)-**11** (97: 3 *dr*).

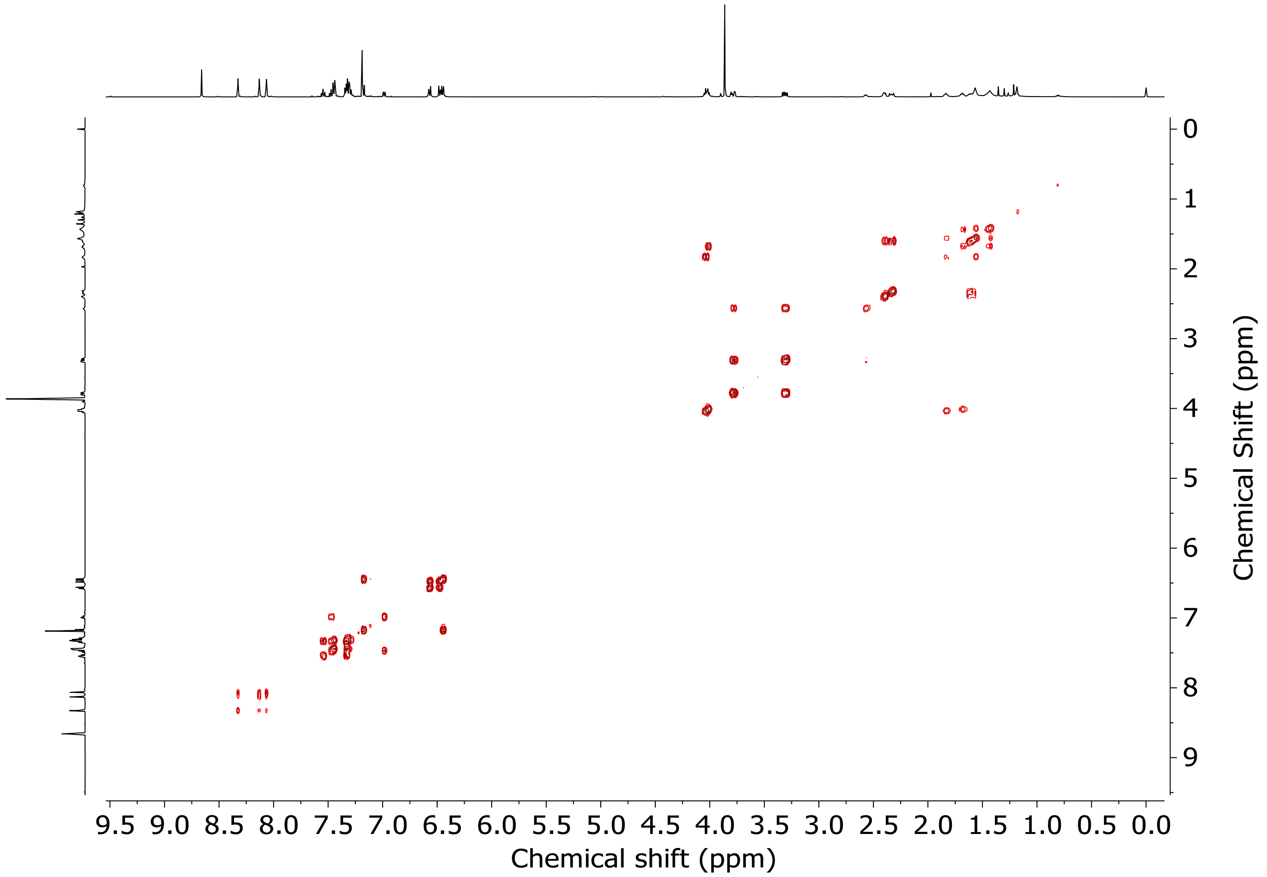

Figure S97: COSY NMR ($CDCl_3$) of (E_m)-**11** (97: 3 *dr*).

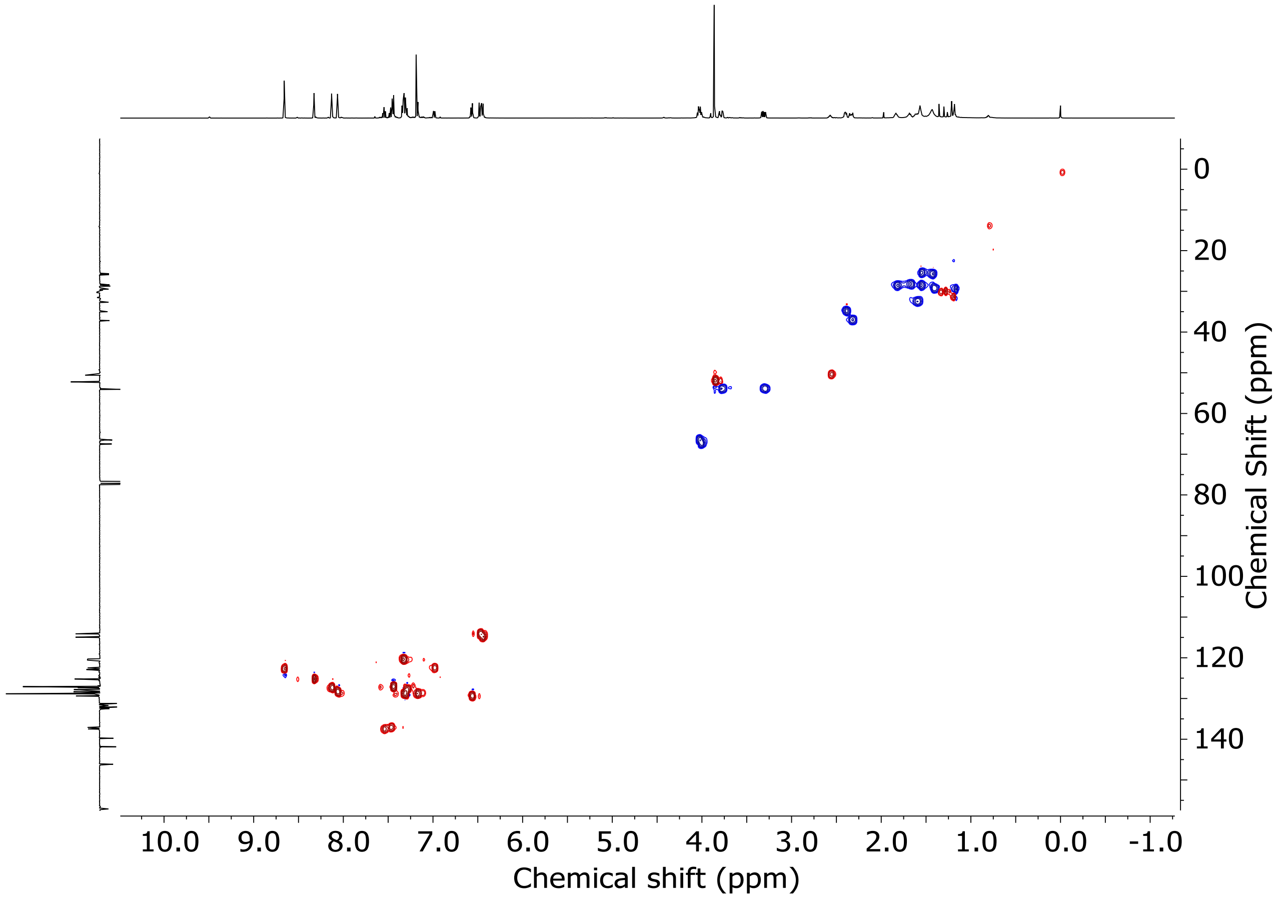

Figure S98: HSQC NMR ($CDCl_3$) of (E_m)-**11** (97: 3 *dr*).

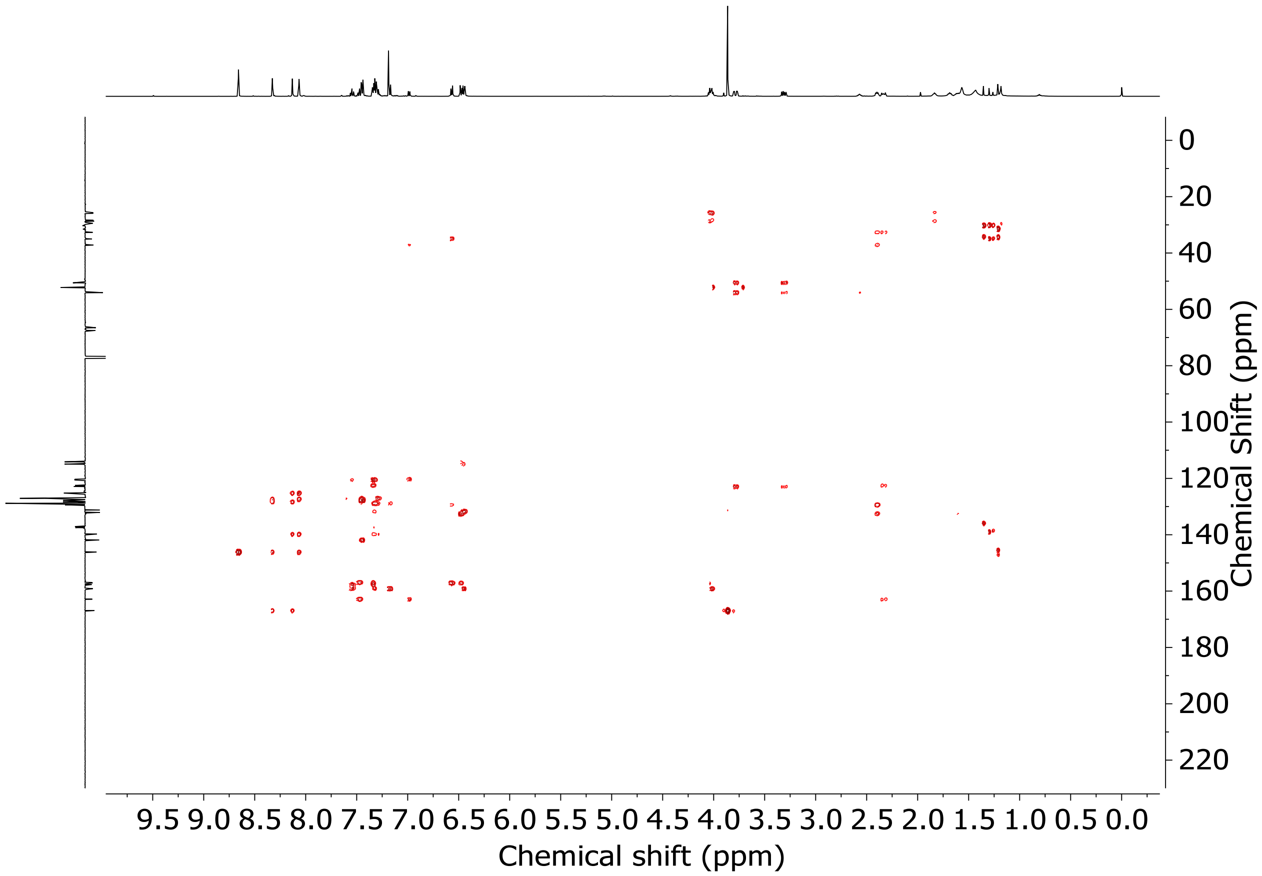

Figure S99: HMBC NMR ($CDCl_3$) of (E_m)-**11** (97: 3 *dr*).

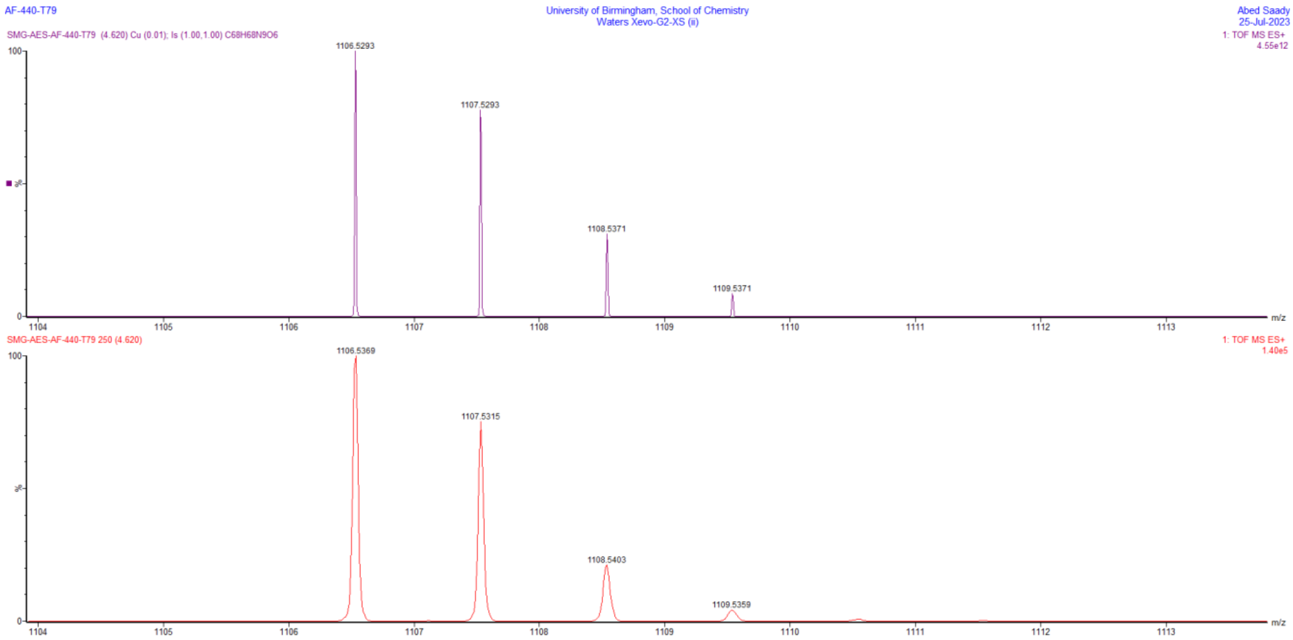

Figure S100: Calculated (top) and observed (bottom) isotopic patterns for (E_m)-**11**.

1.11 Rotaxane ($(Z_m,S_{co\text{-}c})$-10)

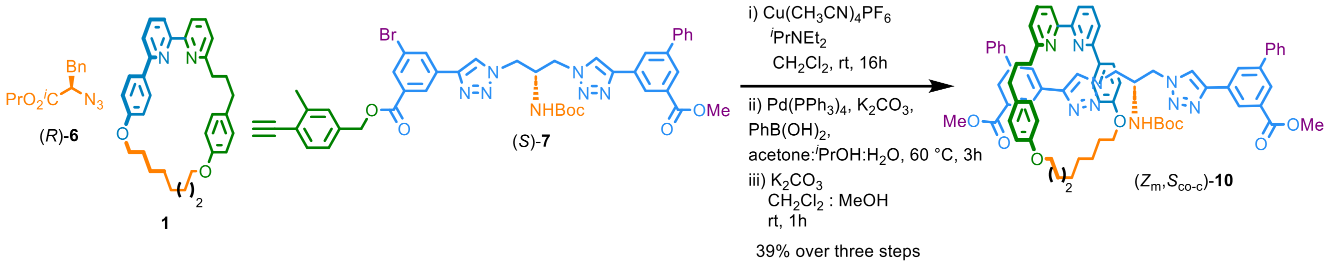

A dry sealed vessel was charged with (*S*)-**7** (50 mg, 60.2 µmol), (*R*)-**6** (14 mg, 60.0 µmol), **1** (27 mg, 55.0 µmol), $[Cu(MeCN)_4]PF_6$ (20.3 mg, 54.5 µmol), $^iPr_2NEt$ (39.2 µL, 0.21 mmol), and anhydrous CH_2Cl_2 (5 mL). The reaction mixture was stirred at rt for 16 h. The solution was diluted with CH_2Cl_2 (5 mL), then EDTA-NH_3 (5 mL) was added. The solution was vigorously stirred until complete decolouration. The aqueous and organic phases were separated, and the aqueous phase was then extracted with CH_2Cl_2 (3 x 10 mL). The combined organic extracts were washed with brine (10 mL), dried ($MgSO_4$) and concentrated *in vacuo* to give a sample containing a mixture of diastereomers (2 : 98 *dr*, Figure S102). The residue was used in the next step without further purification. The crude reaction mixture, $PhB(OH)_2$ (25.5 mg, 0.2 mmol), K_2CO_3 (29 mg, 0.2 mmol), and $Pd(PPh)_4$ (6 mg, 51.9 µmol) were dissolved in solvents mixture acetone:^iPrOH:H_2O (2:1:1, 4 mL) (degassed) and stir for 3 h at 60 °C. After cooling to rt, the solution was diluted with CH_2Cl_2 (5 mL). The aqueous and organic phases were separated, and the aqueous phase was then extracted with CH_2Cl_2 (3 x 5 mL). The combined organic extracts were washed with brine (5 mL), dried ($MgSO_4$) and concentrated *in vacuo,* then dissolved in MeOH:CH_2Cl_2 (1:1, 10 mL) and K_2CO_3 (30 mg, 0.2 mmol) was added. The suspension was stirred at rt for 1 h, then solvents were removed *in vacuo*. Chromatography (petrol-EtOAc 0→30%) gave $(Z_m,S_{co\text{-}c})$-**10** as a colourless oil (25.8 mg, 39% over three steps, 94% *de*).

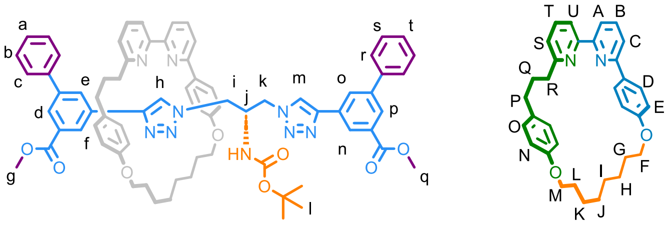

^{1}H NMR (500 MHz, $CDCl_3$) δ: 9.75 (s, 1H, H_h), 8.52 (bs, 1H, H_d), 8.27 (t, *J* = 1.6, 1H, H_n), 8.25 (t, *J* = 1.7, 1.1H, H_p), 8.20 (t, *J* = 1.7, 1H, H_o), 8.16 (t, *J* = 1.6 Hz, 1H, H_f), 8.14 (bs, 1H, H_e), 7.75 (s, 1H, H_m), 7.73-7.65 (m, 3H, H_B, H_r or H_s), 7.59 (t, *J* = 7.9, 1H, H_T), 7.53-7.39 (m, 6H, H_A, H_C, H_r or H_s, H_t, H_U), 7.39-7.35 (m, 2H, H_b or H_c), 7.29-7.25 (m, 3H, H_a, H_b or H_c, superimposed with $CDCl_3$), 7.22 (d, *J* = 8.7 Hz, 2H, H_D), 7.12 (d, *J* = 7.8 Hz, 1H, H_S), 6.56 (d, *J* = 8.5, 2H, H_O), 6.44 (d, *J* = 8.9, 2H, H_E), 6.32 (d, *J* = 8.4 Hz, 2H, H_N), 5.44 (d, *J* = 6.1, 1H, NH), 4.26-4.10 (m, 3H, H_k, H_M), 4.10-3.95 (m, 5H, H_F, H_q), 3.94-3.77 (m, 6H, H_g, H_i, H_j, $H_{M'}$), 3.64 (dd, *J* = 14.4, 4.1, 1H, $H_{i'}$), 2.66 (td, *J* = 13.7, 4.4, 1H, H_R), 2.57-2.44 (m, 3H, H_P, $H_{R'}$), 2.01-1.48 (m, 12H, H_G, H_H, H_I, H_J, H_K, H_L, H_Q, superimposed with H_2O), 1.28 (s, 9H, H_l)

^{13}C NMR (126 MHz, CDCl$_3$) δ: 167.6, 167.3, 163.4, 159.7, 159.2, 158.0, 157.6, 157.5, 146.9, 146.3, 142.6, 141.7, 140.3, 140.2, 137.8, 137.5, 133.1, 132.5, 132.0, 131.8, 131.7, 131.3, 129.4, 129.1, 129.1, 129.0, 128.9, 128.5, 128.2, 127.9, 127.7, 127.3, 127.3, 126.0, 125.8, 122.7, 121.8, 120.9, 120.8, 120.6, 115.0, 114.2, 97.8, 80.5, 77.5, 77.2, 68.2, 66.9, 52.8, 52.6, 51.5, 50.8, 50.1, 37.7, 35.4, 29.6, 28.9, 28.9, 28.8, 28.6, 26.4, 25.9.

HR-ESI-MS (+ve) *m/z* = 1206.6 [M+H]$^+$, for isotopic pattern seeFigure S108

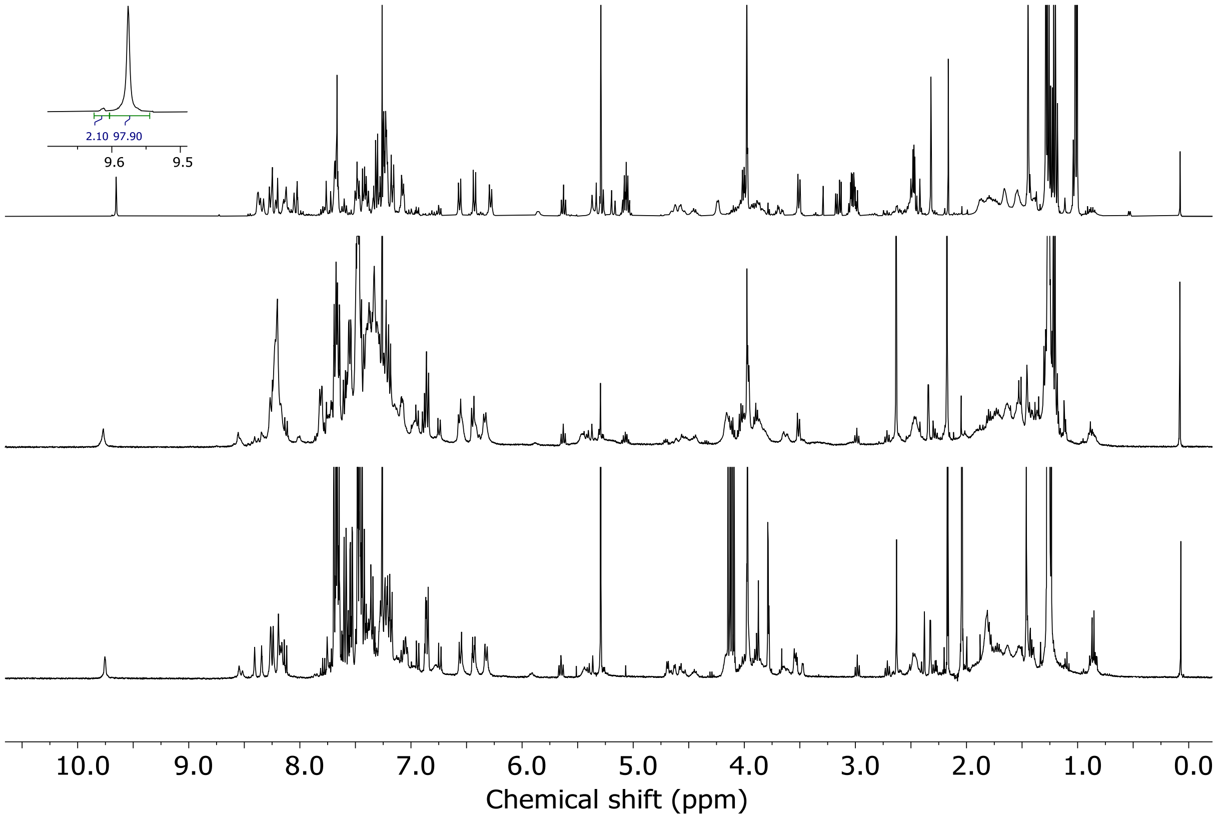

Figure S101: ^{1}H NMR (CDCl$_3$, 400 MHz) of steps towards (Z_m,S_{co-c})-**10** prior to chromatography; first step (top), second step (middle) and third step (bottom).

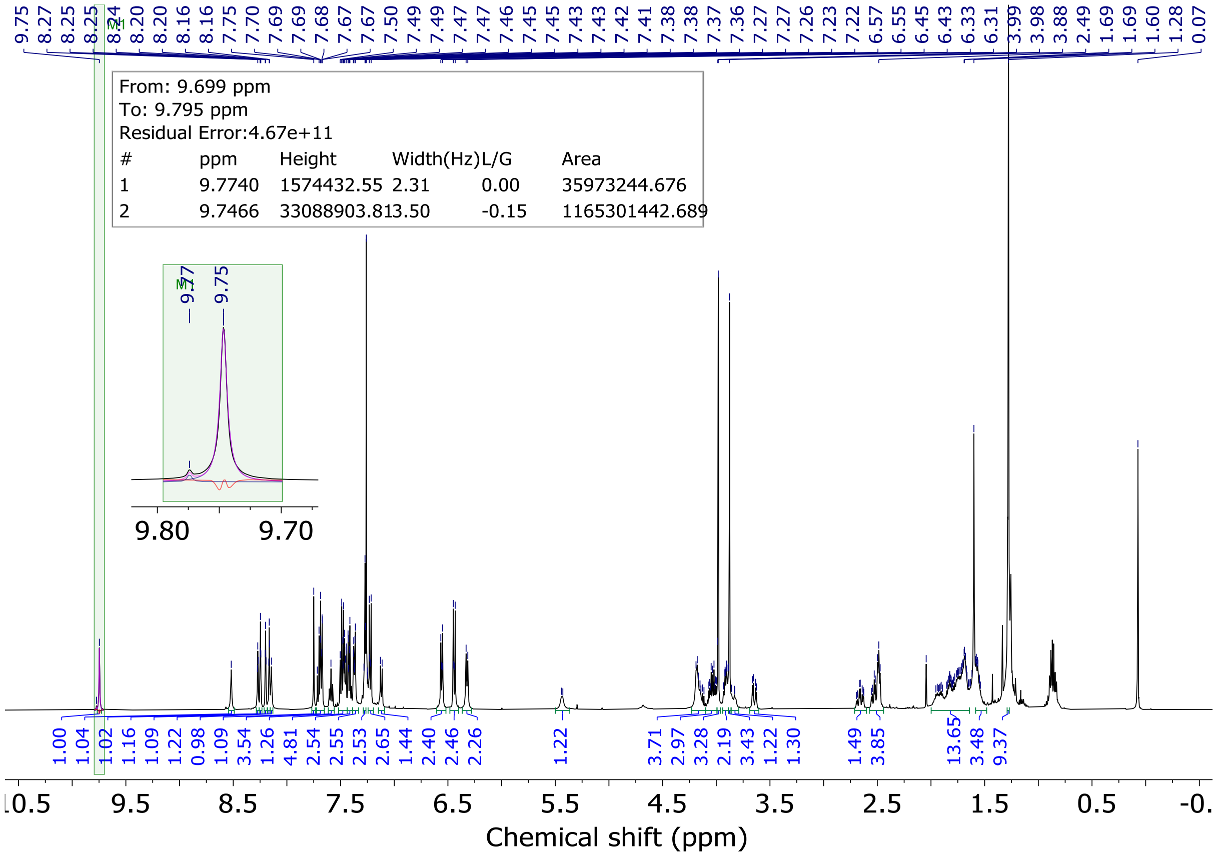

Figure S102: ^{1}H NMR ($CDCl_3$, 500 MHz) of (Z_m,$S_{co\text{-}c}$)-**10** (97 : 3 *dr*).

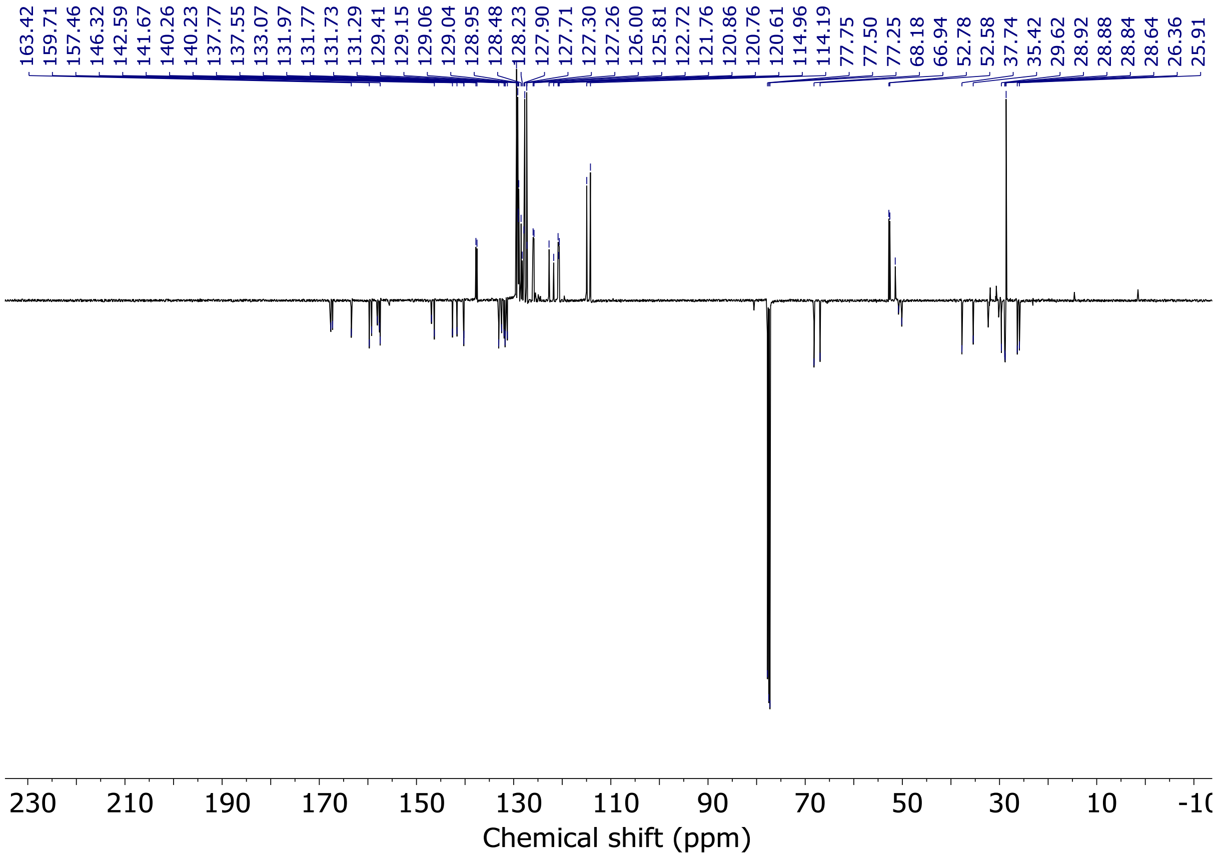

Figure S103: JMOD NMR ($CDCl_3$, 126 MHz) of (Z_m,$S_{co\text{-}c}$)-**10** (97 : 3 *dr*).

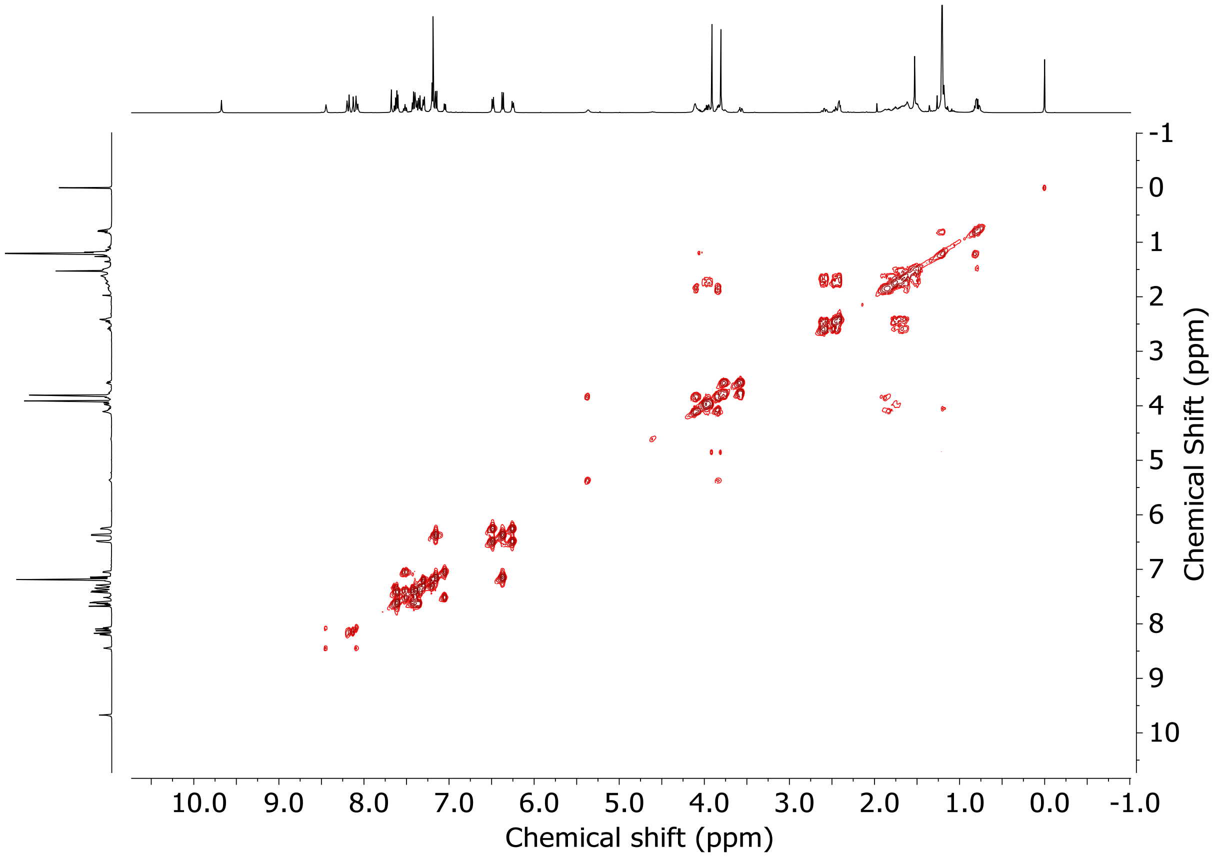

Figure S104: COSY NMR ($CDCl_3$) of (Z_m,$S_{co\text{-}c}$)-**10** (97 : 3 *dr*).

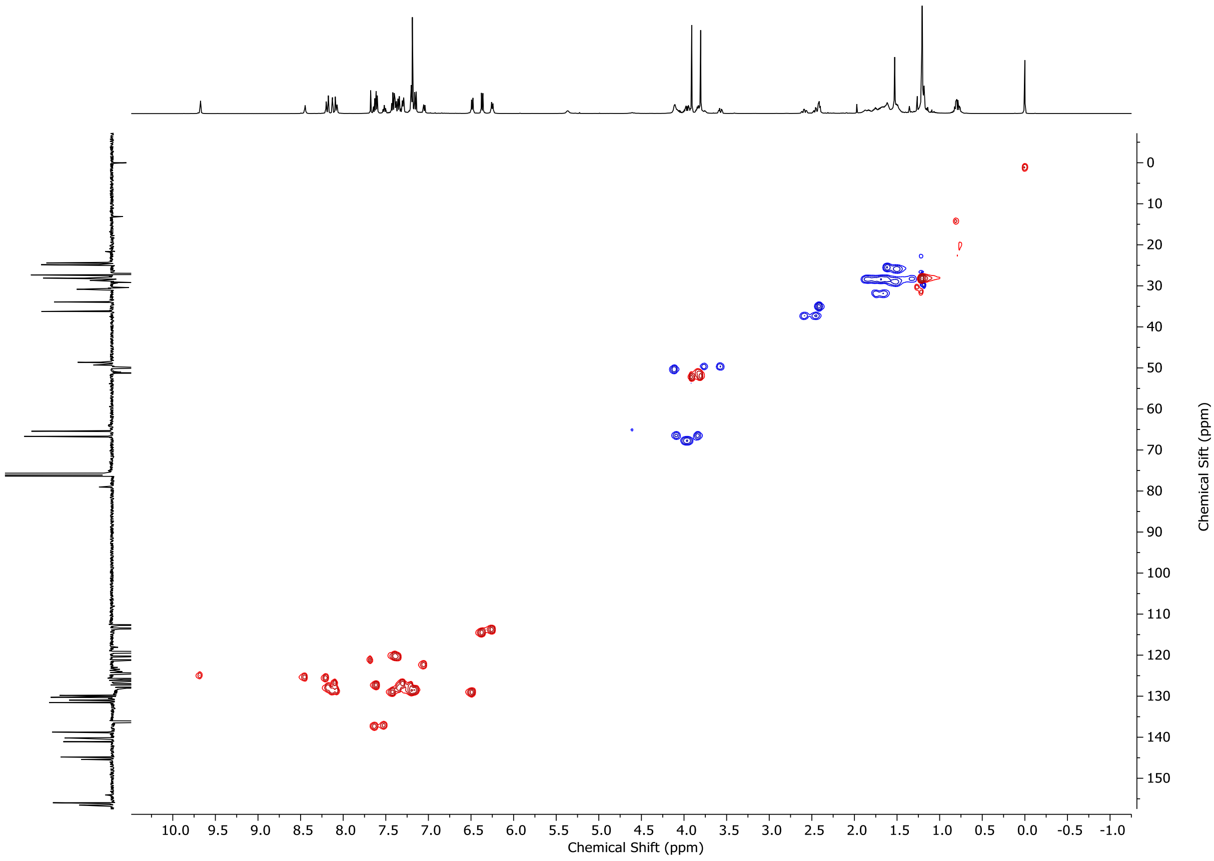

Figure S105: HSQC NMR ($CDCl_3$) of (Z_m,$S_{co\text{-}c}$)-**10** (97 : 3 *dr*).

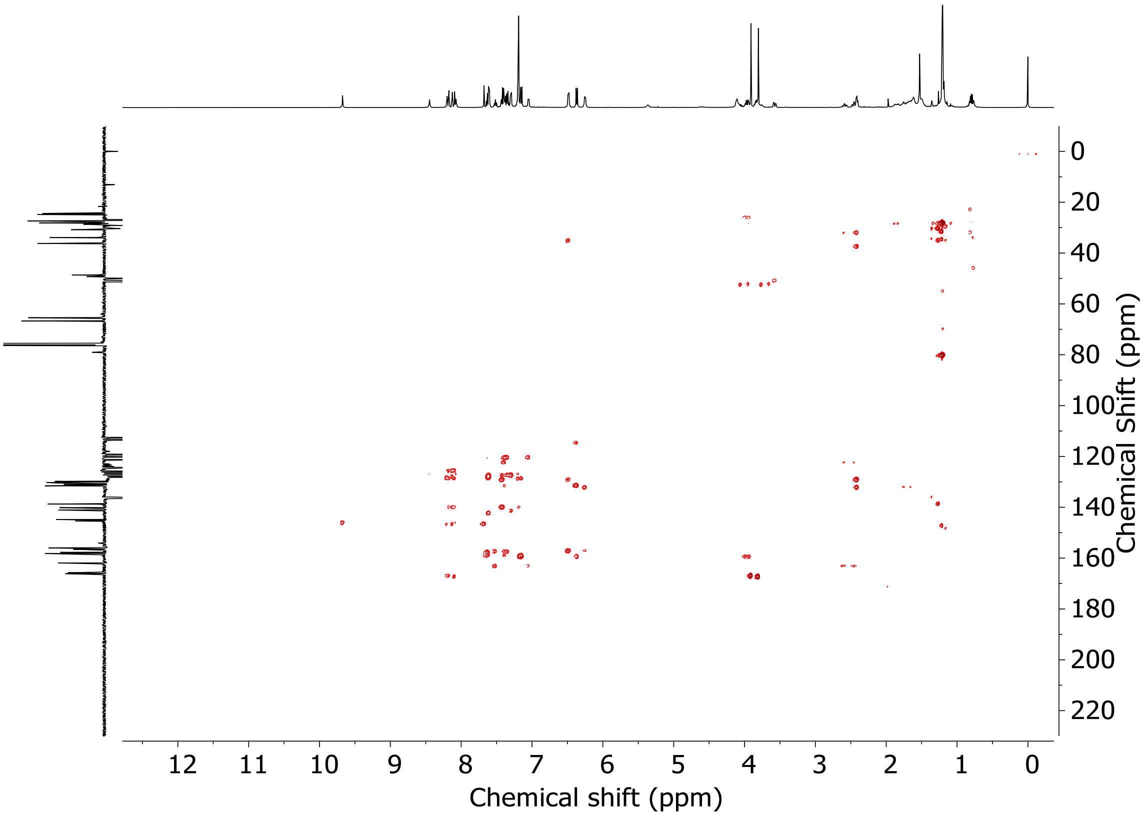

Figure S106: HMBC NMR ($CDCl_3$) of (Z_m,$S_{co\text{-}c}$)-**10** (97 : 3 *dr*).

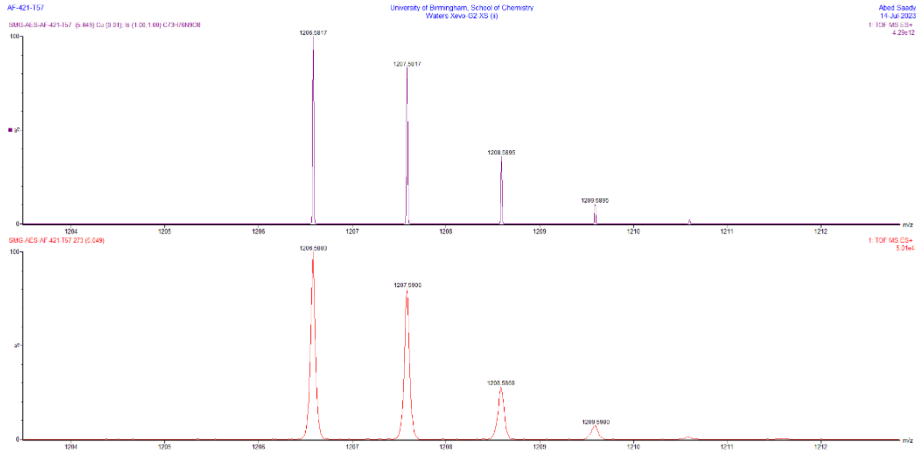

Figure S107: Calculated (top) and observed (bottom) isotopic patterns for (Z_m,$S_{co\text{-}c}$)-**10**.

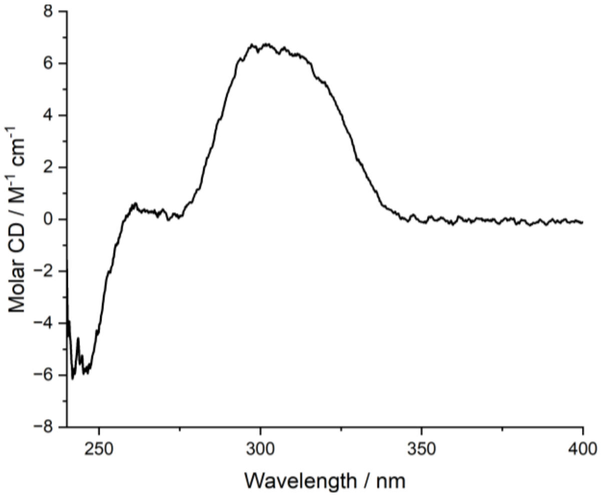

Figure S108: Circular dichroism spectra of (Z_m,$S_{co\text{-}c}$)-**10**. (63.5 µM) at 293 K in $CHCl_3$

1.12 Rotaxane (Z_m)-11

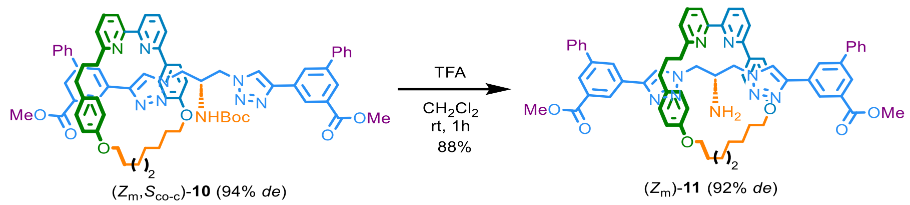

A vessel was charged with rotaxane (Z_m,S_{co-c})-**10** (15.0 mg, 0.012 mmol), TFA (21.4 µL, 0.18 mmol), and CH_2Cl_2 (1 mL). The reaction mixture was stirred at rt for 1 h. CH_2Cl_2 (10 mL) was added, washed with sat. $NaHCO_3$ solution (10 mL), brine (10 mL), and the combined organic extracts were dried ($MgSO_4$) and concentrated *in vacuo*. Chromatography (*n*-hexane-EtOAc 0→40%) gave rotaxane (Z_m)-**11** as a white foam (12.1 mg, 88%, 92% *de*).

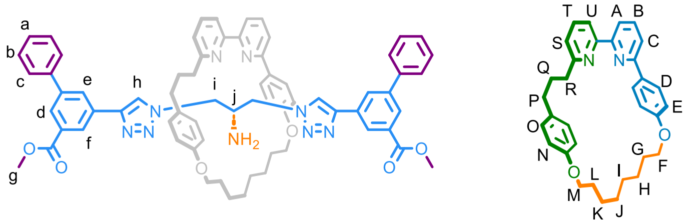

^{1}H NMR (500 MHz, CDCl$_3$) δ: 8.74 (s, 2H, H_h), 8.38 (t, J = 1.7, 2H, H_f), 8.19 (t, J = 1.7, 2H, H_d), 8.13 (t, J = 1.7, 2H, H_e), 7.60 (app t, J = 7.8, 1H, H_B),7.57-7.50 (m, 5H, H_b or H_c, H_T), 7.43-7.33 (m, 9H, H_a, H_b or H_c, H_A, H_C, H_U), 7.07 (d, J = 7.8, 1H, H_S), 7.21 (d, J = 8.6, 2H, H_E), 6.65 (d, J = 8.4, 2H, H_N), 6.56 (d, J = 8.6, 2H, H_D), 6.50 (d, J = 8.4, 2H, H_O), 4.10 (t, J = 6.1, 2H, H_F), 4.05 (t, J = 6.0, 2H, H_M), 3.93 (s, 6H, H_g), 3.77 (dd, J = 14.4, 4.2, 2H, H_i), 3.66-3.59 (m, 3H, $H_{i'}$, NH), 2.99 (app. sept. J = 3.5, 1H, H_j), 2.49 (t, J = 5.3, 2H, H_R), 2.46-2.40 (m, 2H, H_P) 1.87 (t, J = 5.4, 2H, H_L), 1.83-1.43 (m, 12H, H_G, H_H, H_I, H_J, H_K, H_Q, superimposed with H_2O)

^{13}C NMR (125 MHz, CDCl$_3$) δ: 166.9, 162.8, 159.1, 159.1, 157.7, 157.1, 157.0, 146.1, 141.8, 139.8, 137.4, 137.1, 132.4, 132.1, 131.8, 131.1, 129.4, 128.8, 128.7, 128.4, 127.8, 127.3, 127.1, 125.3, 122.8, 122.5, 120.5, 120.5, 120.3, 114.9, 114.0, 67.5, 66.4, 53.8, 52.2, 50.2, 37.1, 35.0, 32.5, 29.5, 28.7, 28.6, 28.5, 25.9, 25.6.

HR-ESI-MS (+ve) m/z = 1106.5 [M+H]$^+$, for isotopic pattern see Figure S115

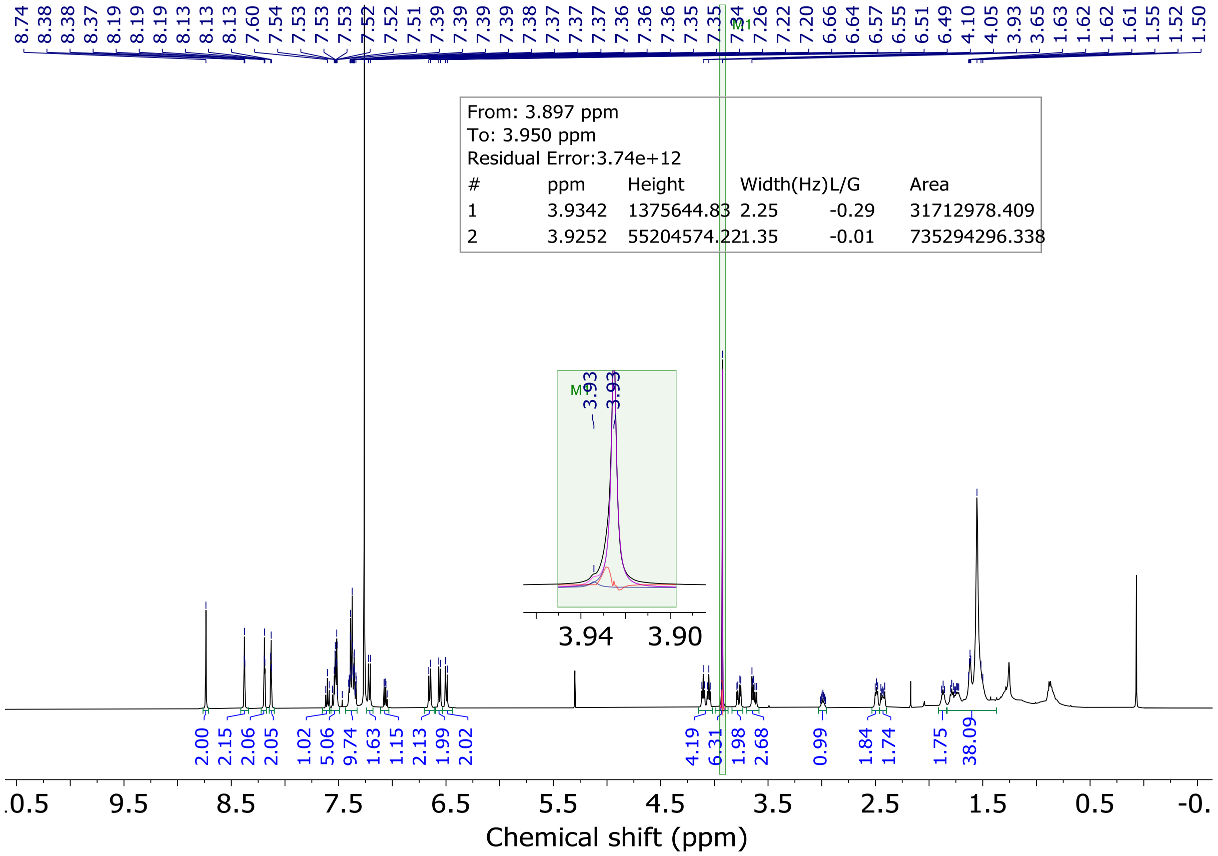

Figure S109: ^{1}H NMR ($CDCl_3$, 500 MHz) of (Z_m)-**11** (96 :4 *dr*).

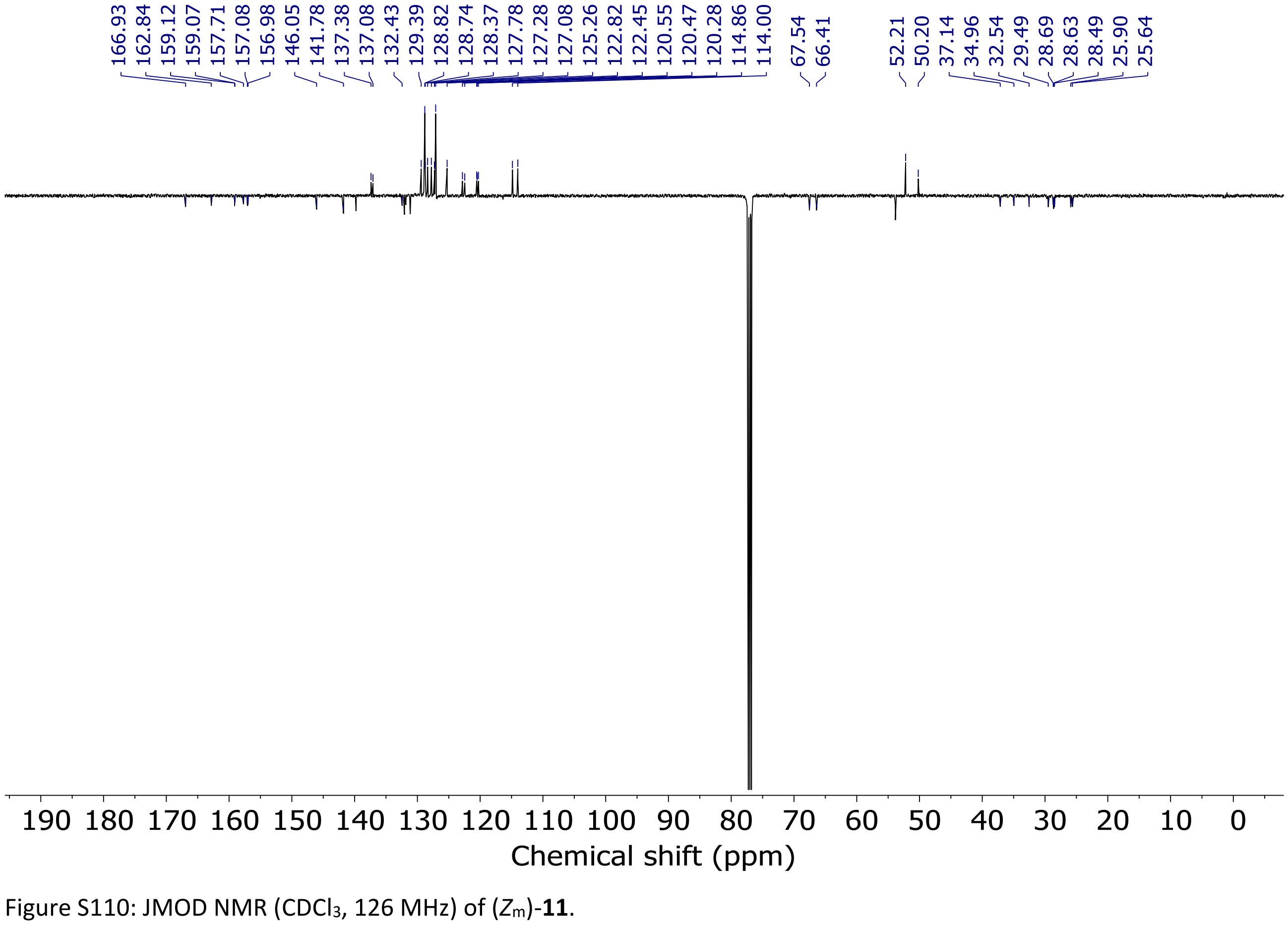

Figure S110: JMOD NMR ($CDCl_3$, 126 MHz) of (Z_m)-**11**.

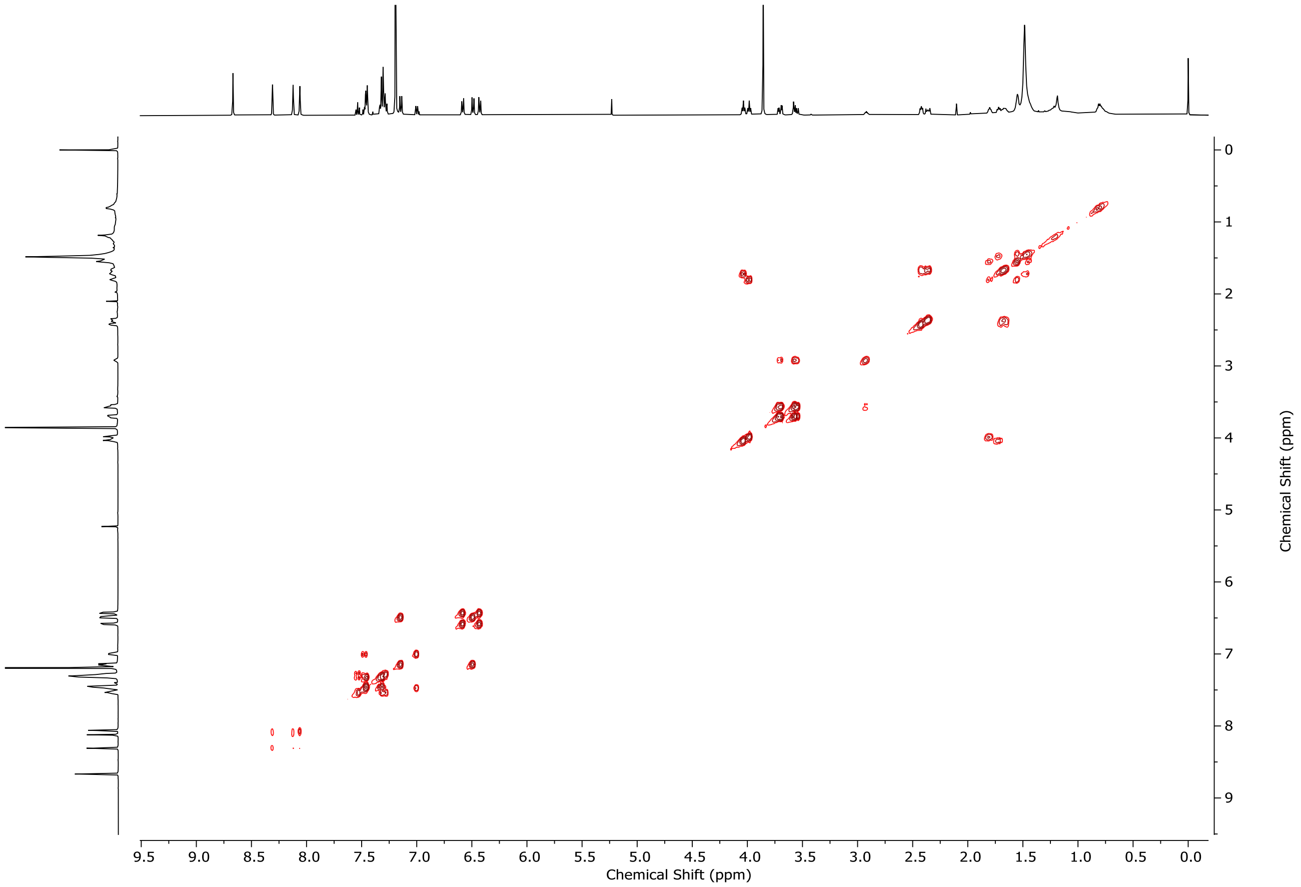

Figure S111: COSY NMR ($CDCl_3$) of (Z_m)-**11**.

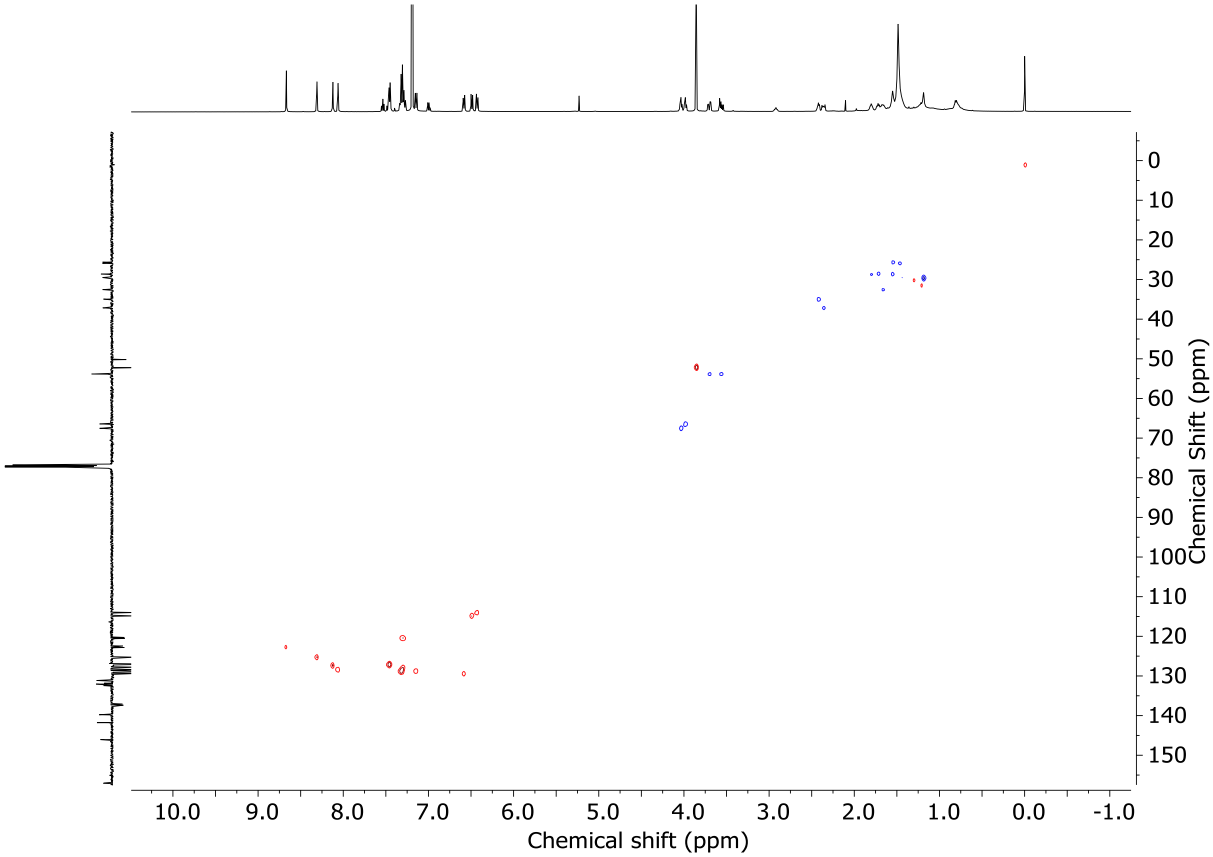

Figure S112: HSQC NMR ($CDCl_3$) of (Z_m)-**11**.

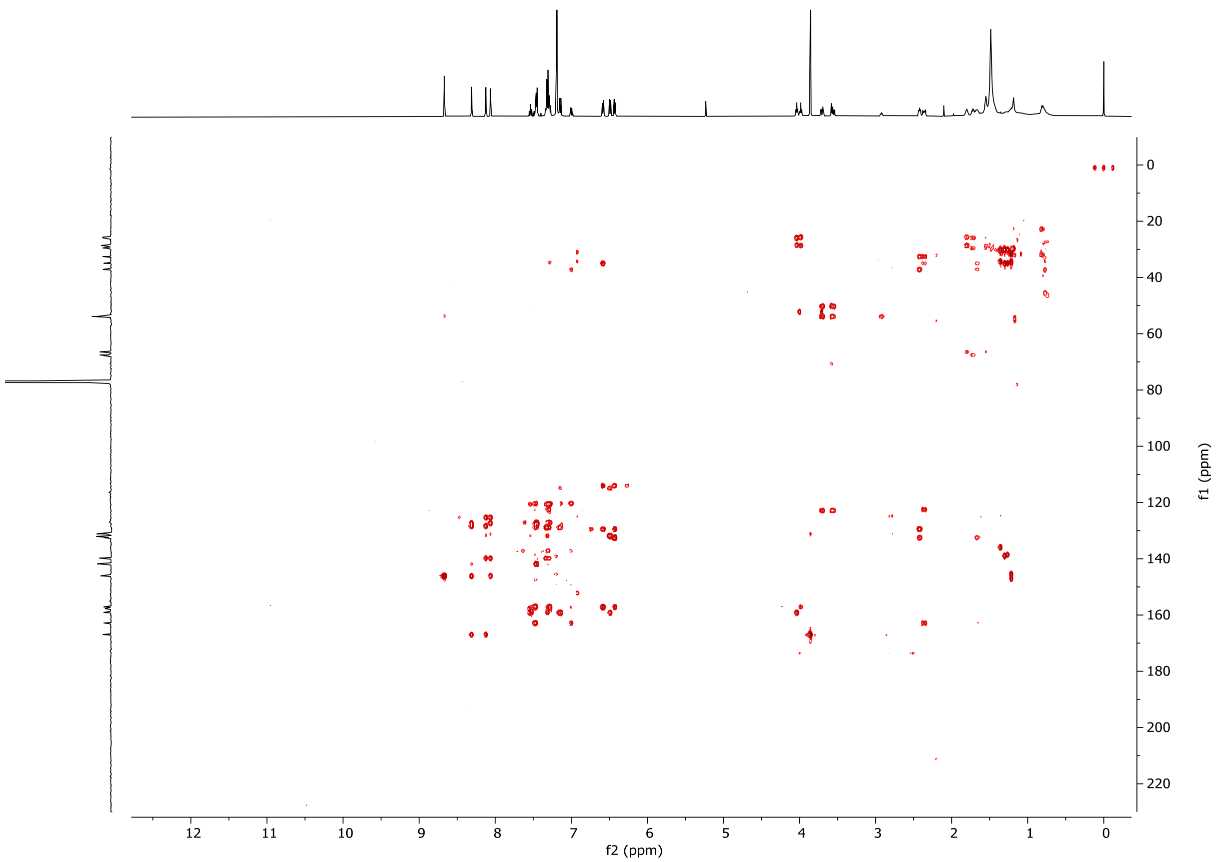

Figure S113: HMBC NMR ($CDCl_3$) of (Z_m)-**11**.

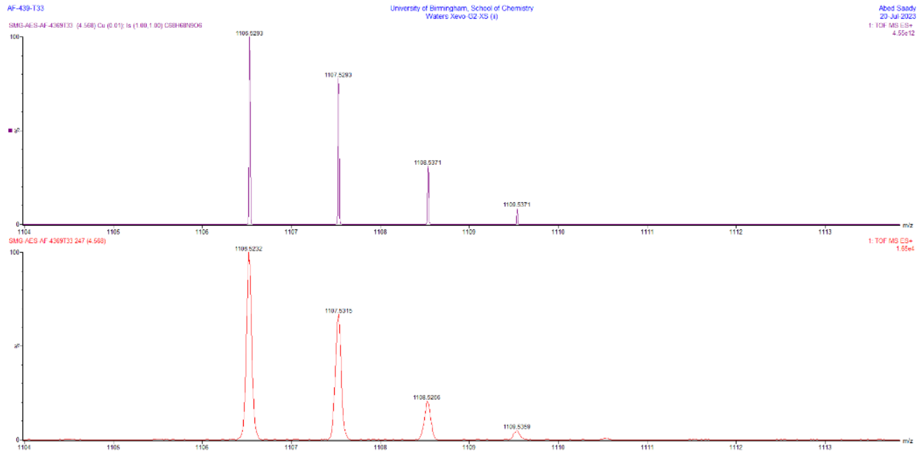

Figure S114: Calculated (top) and observed (bottom) isotopic patterns for (Z_m)-**11**.

6. Absolute Stereochemistry of Type 2 Mechanical Geometric Isomers

Methods to assign the absolute stereochemistry of interlocked molecules are still in development. However, we have previously proposed methods for the assignment of absolute stereochemistry in mechanically planar chiral catenanes and rotaxanes, which are based on oriented covalent sub-units[7] and mechanically axially chiral catenanes and rotaxanes, which are based on facially dissymmetric components.[4] In all cases we make use of Cahn-Ingold-Prelog (CIP)-derived atom priorities to unambiguously assign vectors associated the bilateral dissymmetry of the covalent sub-components. Here we extend these rules to mechanical geometric isomers. In all cases, the stereochemical assignment is achieved by considering the relative orientation of the vectors associated with the individual components. Those vectors can be identified by a step-by-step approach based on the rules outlined below.

A particular challenge with assigning the absolute stereochemistry of the non-canonical type 2 mechanical geometric isomers of rotaxanes is that the outcome depends on the relative orientation of the axle within the cavity of the oriented macrocycle (c.f., deciding which orientation to observe a covalent stereocenter to assign the stereolabel). To ensure that the notional interconversion of type **1**, type **2** and catenane mechanical geometric isomers occurs with retention of stereolabel, we propose that the axle be oriented with the in-plane substituents of the prochiral center pointing towards the observer. We note that this contradicts our previous proposal for the assignment of the related mechanical axial stereogenic unit of rotaxanes and for this reason we have decided to revise our guidelines on the latter.[8] Based on this, the following rules can be used for the absolute stereochemistry of type **II** mechanically geometric isomers of rotaxanes:

Step 1: Identify the highest priority atom in the oriented macrocycle using the CIP priority rules and label it as "**A**".

Step 2: Moving outward from **A** in spheres, as per the CIP method for assigning covalent stereogenic centers, determine the highest priority atom (CIP) that can be used to define an orientation of the axle (typically a ligand of **A**) and label it as "**B**". The orientation of the macrocycle is defined by the vector **A**→**B**, which, where relevant, passes through the intervening atoms (i.e., follows the bonds).

Step 3: In the axle, identify the highest priority prochiral group (or stereogenic unit in *meso* structures) using the CIP priority of the central atom. Identify the highest priority out of plane ligand of the identified prochiral center, again using CIP priority, and label it as "**C**"; label the lower priority group "**D**".

Step 4: View the assembly with the in-plane substituents of the prochiral unit pointing towards the observer.

Step 5: If the vectors **A**→**B** and **C**→**D** point toward the same direction, the molecule will be labelled as (Z_m). If the vectors point toward opposite directions, the molecule will be labelled as (E_m).

Worked example – type 2 rotaxane 11

Step 1: The atom with the highest priority in the macrocycle according to the CIP rules is the phenolic ether oxygen in the blue fragment highlighted in the figure below, which is labelled as **A.**

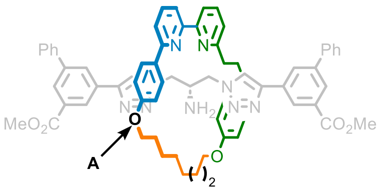

Step 2: Exploring outward from **A**, the atom with the highest CIP priority that defines an orientation of the macrocycle is the quaternary carbon of the aromatic ring. This is labelled as **B.**

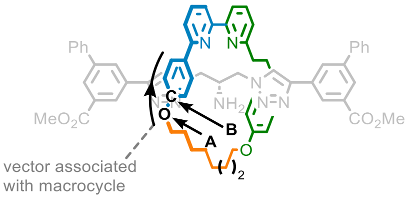

Step 3: Considering the prochiral center, the ligand with the highest CIP priority is the amine nitrogen and the lowest is the hydrogen, they are labelled **C** and **D** respectively.

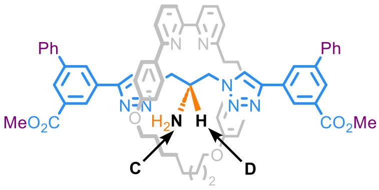

Step 4: Rotate the axle such that the in-plane methylene substituents of the prochiral center point towards the observer.

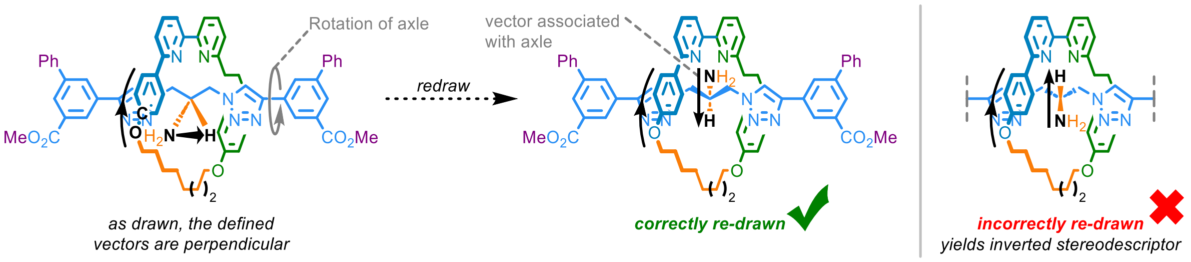

Step 5: View the relative orientations of the vectors **A**→**B** and **C**→**D**. As they point towards opposite directions, the stereoisomer of rotaxane **11** shown is labelled as (E_m)-**11.**

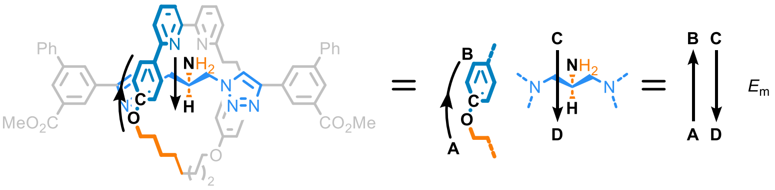

7. Rotaxanes 4, 5, 10 and 11 – three possible sets of stereodescriptors

The separable diastereomers of rotaxanes **4** and **10**, and the co-conformational diastereomers of rotaxanes **5** and **11**, can be fully described with three different sets of stereolabels. This can be readily demonstrated by considering simple structural changes to rotaxane **4** (Figure S116); i. if the prochiral stereocenter is magically flattened (**4`**) the only remaining stereochemistry is co-conformational mechanically planar chiral; ii. if the macrocycle is allowed to occupy the mirror plane associated with the prochiral unit (**4``**) the only stereochemistry arises from a type II mechanical geometric stereogenic unit; iii. if the macrocycle is symmetrized (**4```**) only a co-conformational covalent stereogenic unit remains.

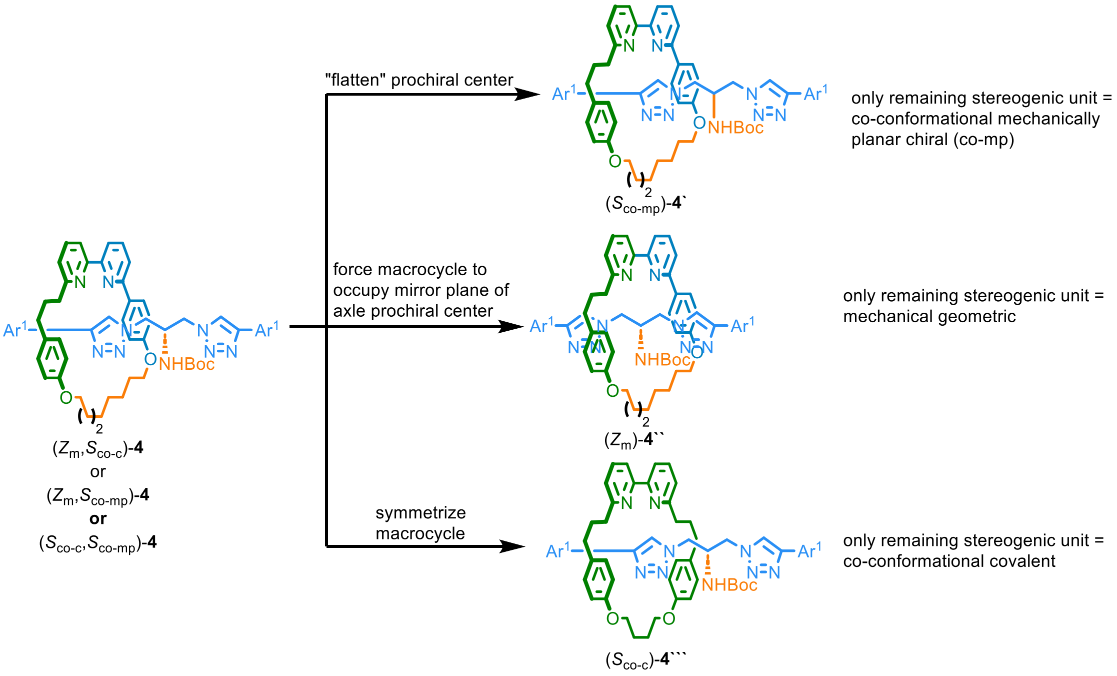

Figure S115: Notional changes to the structure of rotaxane **4** that highlight the different stereogenic units present

Thus, it could be said that rotaxane **4** is simultaneously co-conformationally mechanically planar chiral, displays mechanical geometric type II isomerism and contains a co-conformational covalent stereogenic center. However, if two of these stereogenic units are defined the third is automatically specified and thus the third label is redundant.

In such situations the choice of the two stereolabels to use is somewhat arbitrary but we prefer the geometric + co-conformational covalent description because i. it highlights the form of stereochemistry that does not depend on the co-conformation of the components (the E_m stereodecriptor is co-conformationally invariant, whereas the $S_{co\text{-}mp}$ and $S_{co\text{-}c}$ descriptors are not – the notional shuttling of the macrocycle from one of the axle to the other inverts these labels); ii. the $S_{co\text{-}c}$ stereolabel is more intuitively obvious than the $S_{co\text{-}mp}$ stereolabel and highlights the desymmetrization of the axle.

8. References

1. Pigorsch, A.; Köckerling, M., The Crystallization of Extended Niobium-Cluster Framework Compounds: A Novel Approach Using Ionic Liquids. *Cryst. Growth Des.* **2016**, *16* (8), 4240.

2. de Juan, A.; Lozano, D.; Heard, A. W.; Jinks, M. A.; Suarez, J. M.; Tizzard, G. J.; Goldup, S. M., A chiral interlocking auxiliary strategy for the synthesis of mechanically planar chiral rotaxanes. *Nat. Chem.* **2022**, *14* (2), 179.

3. Ogi, S.; Ikeda, T.; Wakabayashi, R.; Shinkai, S.; Takeuchi, M., A bevel-gear-shaped rotor bearing a double-decker porphyrin complex. *Chem. Eur. J.* **2010**, *16* (28), 8285.

4. Maynard, J. R. J.; Gallagher, P.; Lozano, D.; Butler, P.; Goldup, S. M., Mechanically axially chiral catenanes and noncanonical mechanically axially chiral rotaxanes. *Nat. Chem.* **2022**, *14* (9), 1038.

5. Akae, Y.; Sogawa, H.; Takata, T., Effective Synthesis and Modification of α-Cyclodextrin-Based [3]Rotaxanes Enabling Versatile Molecular Design. *Eur. J. Org. Chem.* **2019**, *2019* (22), 3605.

6. Jinks, M. A.; de Juan, A.; Denis, M.; Fletcher, C. J.; Galli, M.; Jamieson, E. M. G.; Modicom, F.; Zhang, Z.; Goldup, S. M., Stereoselective Synthesis of Mechanically Planar Chiral Rotaxanes. *Angew. Chem. Int. Ed.* **2018**, *57* (45), 14806.

7. Jamieson, E. M. G.; Modicom, F.; Goldup, S. M., Chirality in rotaxanes and catenanes. *Chem. Soc. Rev.* **2018**, *47* (14), 5266.

8. Gallagher, P. R.; Savoini, A.; Saady, A.; Maynard, J. R. J.; Butler, P. V. W.; Tizzard, G. J.; Goldup, S. M., Facial Selectivity in Mechanical Bond Formation: Axially Chiral Enantiomers and Geometric Isomers from a Simple Prochiral Macrocycle. *J. Am. Chem. Soc.* **2024**, 10.1021/jacs.3c14329.
